# Manganese(I)-Catalyzed Enantioselective Alkylation To Access P-Stereogenic Phosphines

**DOI:** 10.1021/jacs.4c16130

**Published:** 2025-01-17

**Authors:** Bin Wan, Marta Castiñeira Reis, Tizian-Frank Ramspoth, Syuzanna R. Harutyunyan

**Affiliations:** † Stratingh Institute for Chemistry, University of Groningen, Nijenborgh 4, 9747 AG Groningen, The Netherlands; ‡ Centro Singular de Investigación en Química Biolóxica e Materiais Moleculares (CIQUS), Universidade de Santiago de Compostela, C/Jenaro de la Fuente s/n, Campus Vida, Santiago de Compostela 15782, Spain

## Abstract

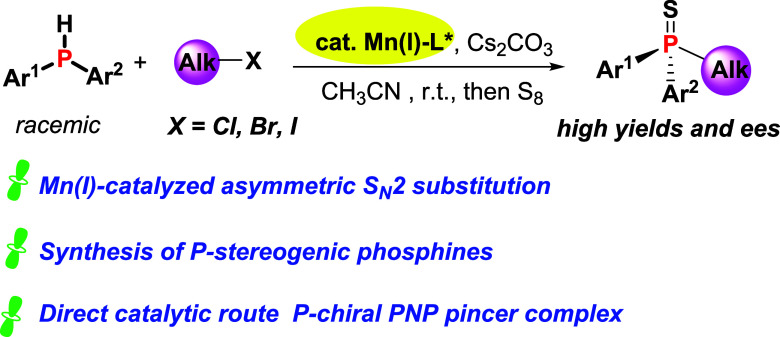

This work introduces a novel Mn(I)-catalyzed enantioselective alkylation methodology that efficiently produces a wide array of P-chiral phosphines with outstanding yields and enantioselectivities. Notably, the exceptional reactivity of Mn(I) complexes in these reactions is demonstrated by their effective catalysis with both typically reactive alkyl iodides and bromides, as well as with less reactive alkyl chlorides. This approach broadens the accessibility to various P-chiral phosphines and simplifies the synthesis of chiral tridentate pincer phosphines to a concise 1–2 step process, contrary to conventional, labor-intensive multistep procedures. Importantly, the development significantly expands the applicability of earth-abundant Mn(I)-based complexes beyond their recently established roles in catalytic hydrogenative and conjugate addition reactions, emphasizing the catalytic potential of Mn(I) complexes as a viable alternative to noble metal chemistry and, in some cases, even surpassing their performance.

## Introduction

Homogeneous catalysis is central to modern organic chemistry, driving efficient transformations across diverse applications.^1^ This often relies on noble transition metals paired with (chiral) phosphine ligands^2^; however, their high costs and limited availability, along with challenges in chiral ligand synthesis, motivate the search for sustainable alternatives. Consequently, earth-abundant metals like iron and manganese have emerged as attractive candidates^3^ for environmentally friendly catalysis that reduces reliance on precious metals while advancing synthetic methods.

Recent advances, particularly in hydrogenation reactions historically dominated by noble metals, have highlighted the potential of manganese.^4^ In 2016, groundbreaking research by Milstein^4a^ and Beller^4b^ showed that Mn(I) catalysts could perform (de)hydrogenation reactions, later extended by Clarke^4c^ and Beller^4d^ to enantioselective variants and asymmetric hydrogenations of heteroaromatics.^5^ These findings suggest that manganese can be a viable alternative to precious metals in terms of both sustainability and reactivity. Previously, manganese was mainly associated with high-valent oxidation chemistry.^6^ Its recent use in low-valent complexes, particularly in hydrogenative processes with pincer and nonpincer ligands, marks a significant shift.^4,5,7^ These complexes enable a range of transformations via Mn–H species formation.

**Scheme 1:**
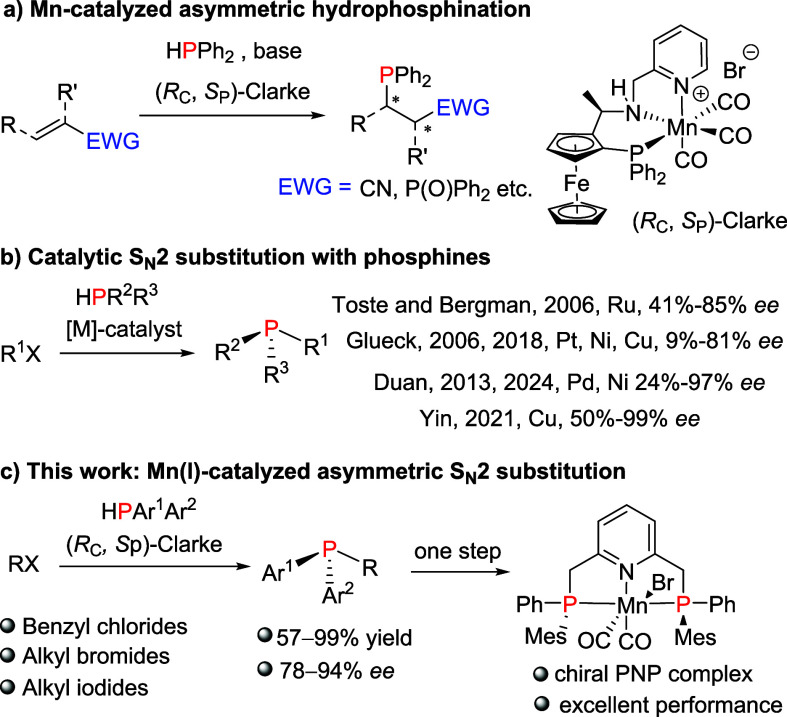
State-of-the-Art and This Work

Our group has recently extended the utility of Mn(I) complexes by demonstrating their ability to activate H–P bonds, achieving enantioselective conjugate additions to various Michael acceptors (Scheme 1a).^8^

These findings underscore the untapped potential of manganese for more sustainable chemistry, emphasizing the need to further explore its catalytic reactivity beyond asymmetric addition reactions.

Building on this, we wondered whether the nucleophilicity of the Mn-phosphido complex could facilitate S_N_2 substitutions with alkyl halides. The catalytic asymmetric S_N_2 alkylation of secondary phosphines shows promise for producing P-stereogenic phosphines, which play a crucial role in asymmetric transformations. Despite recent advances, efficient synthetic methods for P-chiral compounds remain limited, creating a demand for further development in this area.^9^ In 2006, Toste and Bergman’s Ru-iPr-PHOX complex achieved moderate enantioselectivity with benzyl and ethyl chlorides, marking an important milestone.^10^ They later adapted this to a mixed-ligand Ru system for alkyl bromides.^11^ Concurrent work by Glueck^12^ and Duan^13^ developed platinum- and palladium-based systems, respectively. Despite their success, these noble metal systems are limited in scope and often rely on costly metals. Recognizing that these transformations should not be restricted to noble metals, Glueck proposed that base metals could be viable.^14^ Later studies confirmed this with Cu^15^ and Ni^16^ complexes for related reactions. Recently, Yin’s group developed a highly enantioselective Cu-catalyzed method that achieves a broad scope of P-stereogenic products (Scheme 1b),^15b^ while Duan’s Ni-catalyzed approach using primary phosphines further underscores the potential of earth-abundant metals in phosphorus chemistry.^16b^

With these advances in mind, we investigated the potential of chiral Mn(I) complexes as catalysts for enabling the S_N_2 alkylation of secondary phosphines for producing P-stereogenic phosphines (Scheme 1c).

## Results and Discussion

At the outset of our investigation, we selected HPPhMes (**1a**) and benzyl chloride (**2a**) as the model substrates. For catalyzing this transformation, we opted for the (*R*c, *S*p)-Clarke Mn(I) complex, a catalyst utilized in our prior research. Additionally, we decided to quench reactions with S_8_ to safeguard the phosphine products for easier characterization. Initially, toluene served as the solvent, with *t*PenOK as the base, following our previous hydrophosphination conditions for Michael acceptors. However, we observed only 10% of the desired product (**2′a**) under these conditions, which, in addition, was racemic. Notably, the reaction did not proceed in the absence of the catalyst or a base, confirming the indispensability of the Clarke catalyst. Through careful optimization, inspired by both our previous work and reports by Yin on Cu-based systems,^15b^ we identified cesium carbonate (Cs_2_CO_3_) and acetonitrile (CH_3_CN) as the optimal base and solvent, allowing the reaction to be performed at room temperature in 2 h. Under these conditions, the Mn(I)-based catalyst exhibited high efficiency, yielding product **2**′**a** in 90% isolated yield and 90% *ee* (Table 1, entry 1).

**Table 1:**
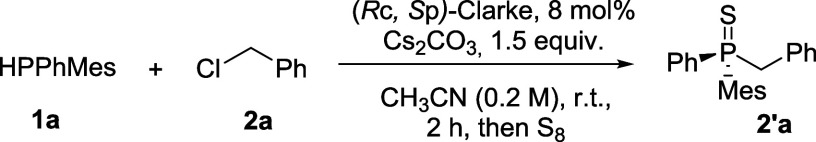
Optimization of the Reaction Conditions^a^

entry	variations	yield 2′a [%]^b^	*ee* 2′a [%]^c^
1^d^	none	90	90
2	Barton’s base	91	84
3	K_2_CO_3_	70	90
4	*i*PrOH	85	92
5	THF	18	80
6	CHCl_3_	0	-
7	toluene	3	-
8^e^	0 °C	76	95
9	16 h	92	81

a Reaction conditions: 0.1 mmol **1a**, 0.12 mmol **2a**, 1.5 equiv; base in 0.5 mL solvent.

b Determined by ^1^H NMR spectroscopy of the reaction crude mixture using 1,3,5-trimethoxybenzene as an internal standard.

c Determined via the chiral SFC system.

d 0.2 mmol of **1a**, 0.24 mmol of **2a**, 0.24 mmol of CS_2_CO_3_, in 1 mL of CH_3_CN, isolated yield.

e Reaction time: 36 h.

We explored alternative bases and found that 2-*tert*-butyl-1,1,3,3-tetramethylguanidine (Barton’s base) resulted in slightly lower enantioselectivity (entry 2), while potassium carbonate (K_2_CO_3_) yielded optimal asymmetric induction but with a lower overall yield (entry 3) due to the remaining substrate. Our optimization studies revealed that relatively weak bases, such as carbonates, are optimal for this system (entries 1 and 3), whereas stronger bases lead to either catalyst decomposition or lower enantioselectivity (entry 2). Notably, solvents capable of solubilizing the base, such as CH_3_CN and *i*PrOH, proved equally effective (entries 1 and 4), providing both a high yield and excellent enantioselectivity. Conversely, solvents like THF, CHCl_3_, and toluene were suboptimal (entries 5–7). Lowering the reaction temperature to 0 °C improved the enantioselectivity to 95% *ee* but with a decreased yield (entry 8 vs entry 1), likely due to configurational stability issues of the phosphine product. This is confirmed by the fact that prolonged reaction times led to decreased enantiomeric purity (entry 9), suggesting racemization of the P(III)-chiral product at room temperature and therefore improvement of the enantioselectivity of the process at lower temperatures. Based on these studies, we identified the optimal reaction conditions: CH_3_CN as the solvent, Cs_2_CO_3_ as the base, 8 mol % Clarke catalyst, and a 2 h reaction time.

**Scheme 2:**
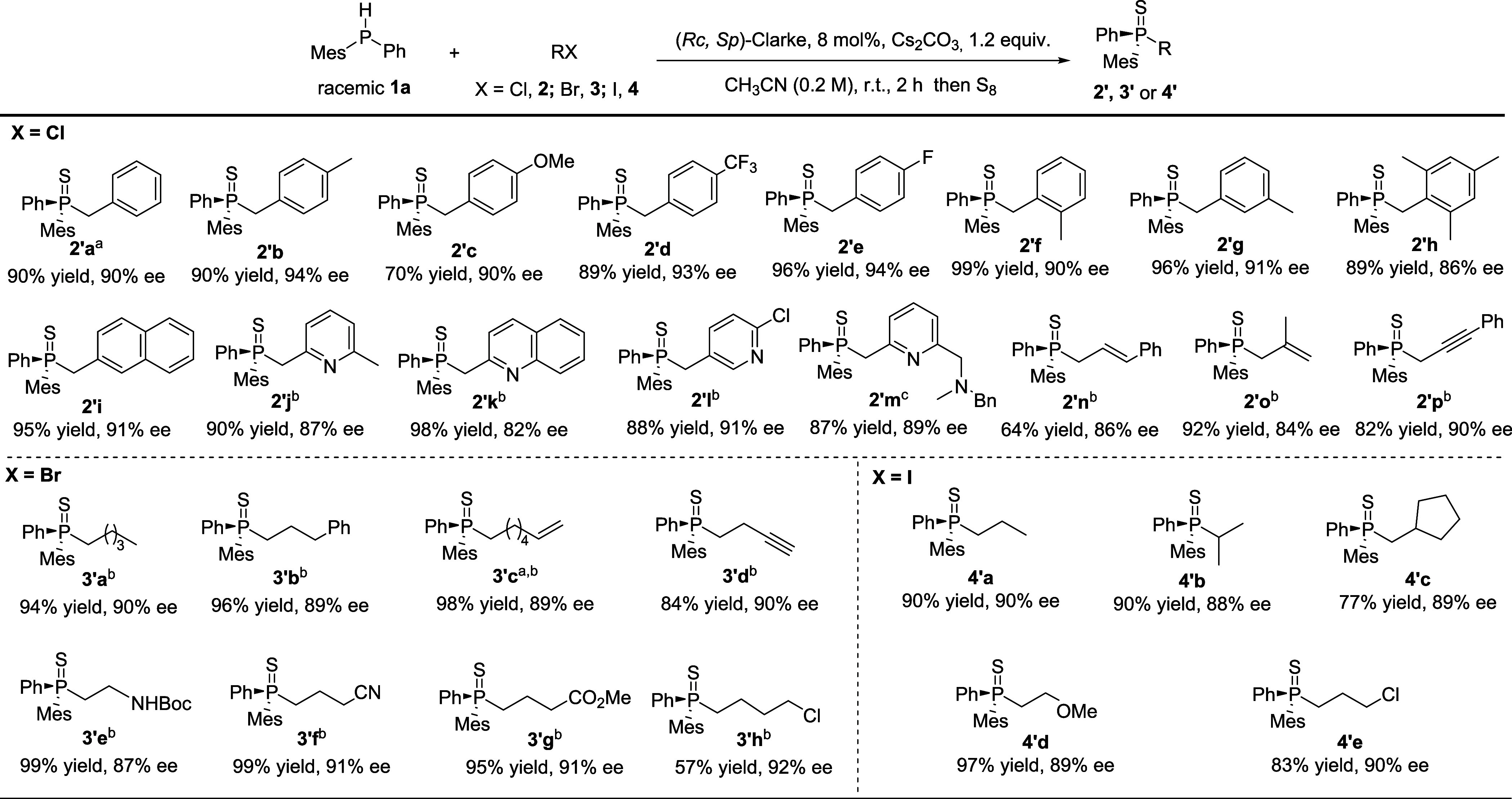
Scope of Organohalides The absolute configurations of **2**′**a** and **3′c** were identified by single-crystal X-ray crystallography (for details, see the SI). 0.1 mmol of **1**, 0.12 mmol of **2** and **3**, and 0.15 mmol of Cs_2_CO_3_ in 0.5 mL of CH_3_CN. 0.1 mmol of **1**, 0.10 mmolof **2m**, and 0.15 mmol of Cs_2_CO_3_ in 0.5 mL of CH_3_CN. Reaction conditions: 0.2 mmol of **1** and 0.24 mmol of **2, 4**, and Cs_2_CO_3_ in 1 mL of CH_3_CN.

After establishing the optimal reaction conditions for the Mn(I)-catalyzed enantioselective S_N_2 substitution of benzyl chloride by diphenylphosphines, we moved to explore the substrate scope (Scheme 2). The results revealed that both aryl groups bearing an electron-donating substituent and those bearing an electron-withdrawing substituent in the para-position were well tolerated in this reaction, as indicated by the reaction outcomes delivering products **2′b–2**′**e**. The steric hindrance at the aryl groups of compounds **2**′**f–2**′**i** minimally impacted both yield and enantioselectivity. Importantly, the catalytic system is compatible with pyridine and quinolone groups, yielding the desired products with high yields and enantiopurities (**2**′**j** and **2**′**k**). Noteworthy is that these substrates, previously incompatible with Cu-based catalytic systems,^14b^ allow two-step access to chiral pincer-like ligand structures, which would otherwise require multistep synthesis. Additionally, our system tolerates not only unsubstituted pyridine but also more decorated analogues (**2**′**l** and **2**′**m**). In addition to benzyl chlorides, we examined other activated chlorides (allyl and propargyl) as electrophilic partners in this reaction. Both substrates were converted fully, affording the corresponding products (**2**′**n–2**′**p**) with high enantiomeric excess (*ee*).

While these findings were promising, we wondered whether our manganese-based catalytic system could handle more challenging, less reactive alkyl halides. As a first test, we turned to alkyl bromides (**3**), which were not compatible with the previously reported Cu-based methodology that required highly activated iodide analogues for alkylation.^14^ We were pleased to observe that alkyl bromides are readily converted by our catalytic system, also demonstrating remarkable tolerance to various substituents, yielding enantioenriched products (**3′a–3′h**). Given the potential applications of this chemistry, especially as ligands for homogeneous catalysis, it is intriguing to consider the compatibility of functional groups that could enhance the binding to the metal. In this context, we successfully isolated product **3′e**, derived from an alkyl bromide bearing a Boc-protected amine **3**. The corresponding unprotected product could potentially serve as a chiral bidentate ligand for similar applications. Our catalytic system shows excellent compatibility with alkyl bromides featuring electron-withdrawing groups, delivering products (**3′f–3h**) with high *ee*. We also explored the effectiveness of alkyl iodides as electrophiles in the reaction. The substrate 1-iodohexane yielded the corresponding product in 85% yield and 92% *ee* (see the SI), similar to results observed with 1-bromohexane **3a**.

Moving on to alkyl iodide substrates (**4**), we noticed that the electronic properties of their substituents did not compromise the high levels of enantioselectivity and reaction efficiency either, as their corresponding products (**4**′**a–4**′**e**) were all obtained with high yields and *ee*.

**Scheme 3:**
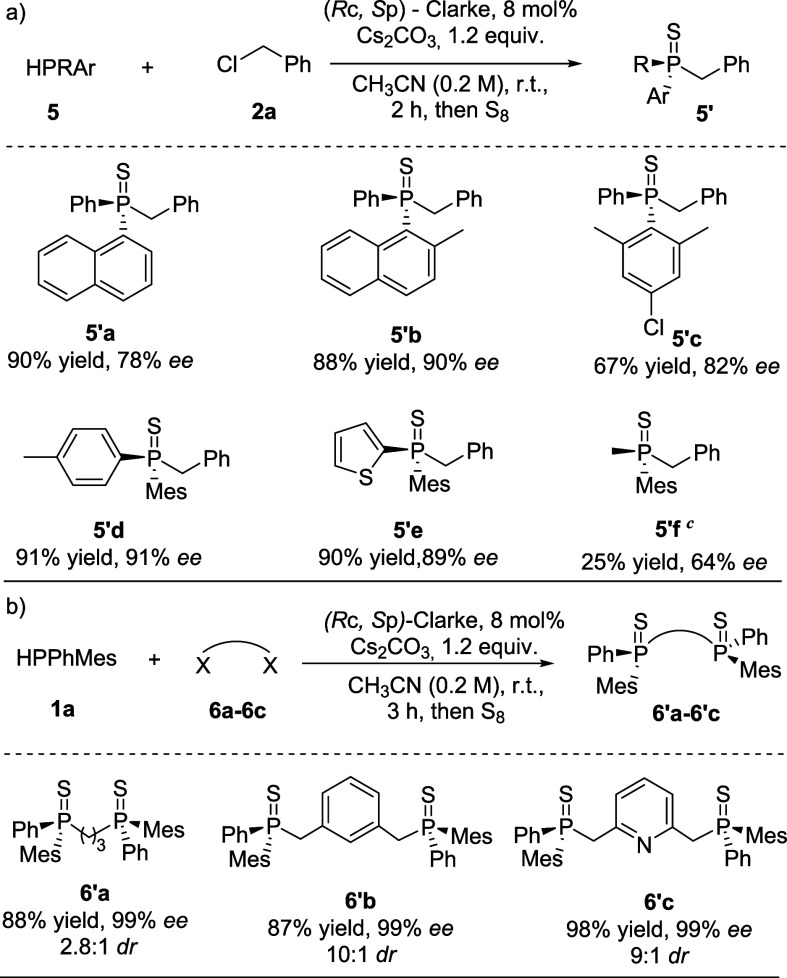
Scope of Phosphines and Dihalides, Reaction conditions for (a) 0.1 mmol of **5**, 0.12 mmol of **2a**, 0.15 mmol of Cs_2_CO_3_ in 0.5 mL of CH_3_CN. Reaction conditions for (b) 0.2 mmol of **1a**; 0.1 mmol of **6a**–**6c** (for **6a** X = Br; for **6b** and **6c** X = Cl), 0.24 mmol of Cs_2_CO_3_ in 1 mL of CH_3_CN. 0.1 mmol of **5f**, 0.2 mmol of **2a**, 0.2 mmol of Cs_2_CO_3_, 0.2 mmol of DBU, 10 mol% (*R*_c_, *S*_p_)-Clarke in 0.5 mL of CH_3_CN for 16 h.

Next, we explored the effect of the aryl group on the phosphorus atom (Scheme 3a). Utilizing less hindered aryl groups in the phosphine moiety or substituting one of the methyl groups of 1a with a chloride led to a modest reduction in the enantioselectivity of the corresponding products (**5′a–5′e**). Interestingly, an alkyl group was also tolerated in this reaction (**5′f**).

Recognizing the significance of bidentate and tridentate ligands in homogeneous catalysis, we pursued the synthesis of chiral diphosphine ligands (Scheme 3b). Notably, diphosphine products **6**′**a** and **6**′**b** were synthesized using 1,3-dibromopropane 6a and 1,3-bis(chloromethyl)benzene **6b**, respectively. More significantly, the reaction with heteroaromatic 2,6-bis(chloromethyl)pyridine **6c** yielded the corresponding product **6**′**c** with 98% yield and 99% *ee*, enabling the straightforward synthesis of a pincer-type tridentate chiral PNP ligand in a single step.

**Scheme 4:**
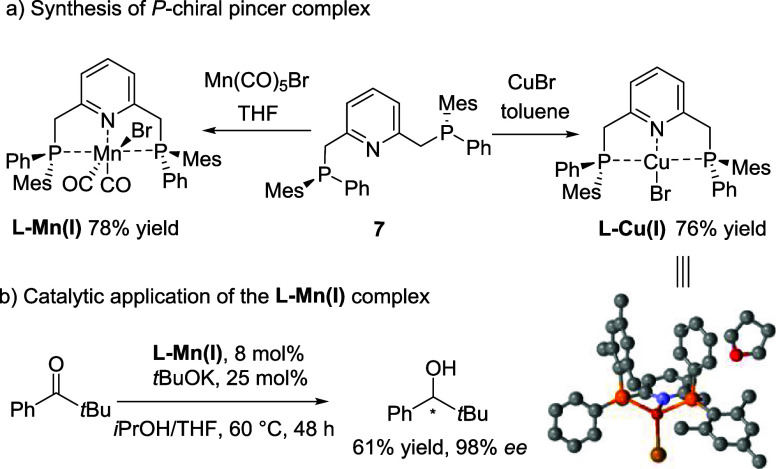
Applications of the Methodology

To demonstrate the utility of this development, upon completion of the reaction between **1a** and **6c**, we quenched the reaction with the corresponding metal salts (instead of elemental sulfur) to form the corresponding Mn(I) and Cu(I) complexes with the unprotected diphosphine **7** (formed prior to **6**′**c**). The resulting Mn(I) and Cu(I) complexes were generated and isolated in good yields (Scheme 4a). We then tested the newly synthesized chiral Mn(I) complex in the asymmetric transfer hydrogenation of an aryl ketone and were pleased to observe its outstanding performance, achieving the corresponding product with a 98% *ee* (Scheme 4b).

The mechanism of this transformation likely follows the S_N_2 pathway, as proposed in previous reports of metal-catalyzed reactions between phosphines and organohalides.^10−16^

**Scheme 5:**
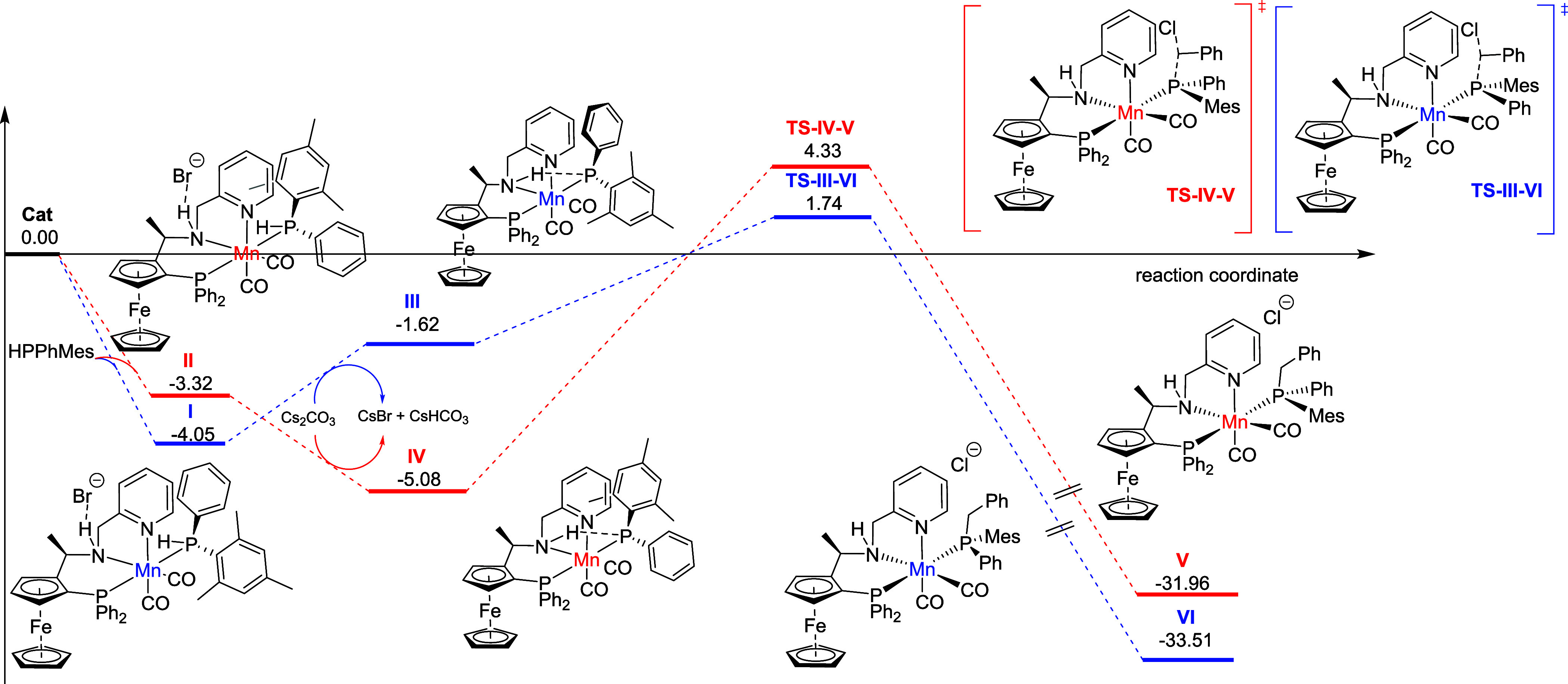
Mechanistic Studies Calculations were performed at the PCM^18^ (acetonitrile)/B3LYP-D3/def2svpp^19^ computational level using the Gaussian 16 program,^20^ and the thermochemistry was obtained at 1 atm and 298 K.

The high reactivity and enantioselectivity observed in the reaction between **1a** and **2a** (yield 85%, *ee* 90%) in the presence of the radical scavenger BHT strongly support a nonradical mechanism.^17^ Furthermore, the reaction’s high efficiency with linear organohalides and the lack of reactivity with bulky substrates like *t*BuCl and *t*BuI further support an S_N_2 pathway. A key question is at what stage does enantiodiscrimination occur within this Mn(I)-based catalytic system and which Mn(I) species are responsible? In our earlier studies on conjugate additions, we proposed that a Mn-phosphido complex forms when diarylphosphine interacts with a chiral Mn(I) complex (Clarke’s catalyst).^8a^ This hypothesis may also extend to the current reaction, where a similar Mn-phosphido complex is formed but now yields two interconverting diastereomeric species that can undergo S_N_2 substitution with halides at different rates. Consequently, enantiodiscrimination may occur either during the formation of the Mn(I)-phosphido complex or in the subsequent substitution step. Our initial attempts to monitor speciation and the formation of diastereoisomers during this reaction using ^31^P NMR were unsuccessful due to the formation of several broad peaks at every stage of the reaction, which complicated structural assignment. Therefore, we turned to molecular modeling (Scheme 5). Our computational analysis began with examining the coordination of the phosphine ligand to Clarke’s catalyst, which revealed the formation of two diastereomeric species, **I** and **II**. In the presence of a base, these species can undergo deprotonation, yielding species **III** and **IV**. Notably, although **I** and **II** differ by only 0.73 kcal/mol, the energy gap between **III** and **IV** increases significantly to 3.46 kcal/mol. This shift is attributed to the disruption of a π–π interaction between the pyridine moiety and the phenyl or mesyl groups present in **I** and **II**, which is replaced in **IV** by a stabilizing *CH*–π interaction between the pyridine and phenyl groups (see the SI). With species **III** and **IV** established, we found that the addition of alkyl halide to **III** is both kinetically and thermodynamically favored, exhibiting an energy barrier of 3.36 kcal/mol and a substantial energy release of 35.25 kcal/mol.

This preference results from the orientation of the phenyl group, which optimally accommodates the electrophilic carbon center and enables a stabilizing π–π interaction between the phosphine’s phenyl group (in the manganese complex) and the phenyl group of the organic halide. This interaction is absent in the diastereomeric transition state (**TS–IV–V**). Additionally, in **IV**, the phosphorus atom’s lone pair engages strongly with the amino group’s hydrogen, reducing its nucleophilicity compared to the phosphorus in **III** (see the SI). This interaction further obstructs substrate accommodation, leading to a higher energy penalty. Our findings suggest that the stereodiscrimination step aligns with the alkyl halide addition stage, where differential interactions dictate the preference for the reaction to occur through species **III**.

## Conclusions

In conclusion, this paper introduces a novel Mn(I)-catalyzed enantioselective alkylation methodology that efficiently produces P-chiral phosphines with excellent yields and enantioselectivities, demonstrating the versatility and potential of Mn(I) complexes. This approach enables the synthesis of chiral tridentate pincer phosphines in a streamlined 1**–**2 step process, significantly expanding the utility of earth-abundant Mn(I) complexes and cementing them as a viable alternative to noble metal catalysts in various synthetic applications.

## Supporting Information Available

Experimental procedures, characterization data, and NMR spectra (PDF)
