# Supplementary material for: Manganese(I)-Catalyzed Enantioselective Alkylation To Access P-Stereogenic Phosphines
Source: J Am Chem Soc. 2025 Jan 17;147(4):3740–6. doi: 10.1021/jacs.4c16130 (PMC11783530; doi:10.1021/jacs.4c16130)
Supplement: Supplementary file 1 — ja4c16130_si_001.pdf [file ja4c16130_si_001.pdf]

Supporting information

Manganese(I)-Catalyzed Enantioselective alkylation to access P-stereogenic phosphines

Bin Wan, Marta Castiñeira Reis, Tizian-Frank Ramspoth , and Syuzanna R. Harutyunyan*

Stratingh Institute for Chemistry, University of Groningen, Nijenborgh 4, 9747 AG, Groningen (The Netherlands)

*Corresponding author. E-mail: s.harutyunyan@rug.nl

Table of Contents

1. General experimental information

2. General computational details

3. Synthesis of substrates

4. General procedure for the substitution reaction

5. Computational mechanistic studies

6. Determination of the absolute configuration

7. Characterization of the products

8. Application of the methodology: Synthesis and characterization of copper complex and manganese complex

9. References

10. NMR spectra

11. Cartesian coordinates and energy reports

1. General experimental information

All reactions using oxygen- and/or moisture-sensitive materials were carried out with anhydrous and deoxygenated solvents under a nitrogen atmosphere using oven dried glassware and standard Schlenk techniques. Reactions were monitored by 1H NMR and 31P NMR. Purification of the products was performed by chromatography using Merck 60 Å 230-400 mesh silica gel. NMR data was collected on Varian VXR400 (^{1}H at 400 MHz; ^{13}C at 100.58 MHz; ^{19}F at 376 MHz; ^{31}P at 162MHz) equipped with a 5 mm z-gradient broadband probe. Chemical shifts are reported in parts per million (ppm) relative to residual solvent peak ($CDCl_3$, ^{1}H: 7.26 ppm, ^{13}C: 77.16 ppm; Coupling constants are reported in Hertz. Multiplicity is reported with the usual abbreviations (s: singlet, d: doublet, br s: broad singlet, dd: doublet of doublets, t: triplet, m: multiplet). Exact mass spectra were recorded on a LTQ Orbitrap XL apparatus with ESI ionization. Enantiomeric excess (*ee*) of all compounds was determined by a Thar SFC system equipped with s chiralpak columns or Water Acquity UPC2 system with PDA detector or by Chiral HPLC analysis using a Shimadzu LC10ADVP HPLC equipped with a Shimadzu SPD-M10AVP diode array detector. Melting points were measured by BUCHI Melting Point B-454 with 10 ^{o}C/min temperature increasing gradient. The data were detected by the machine automatically and rechecked by observation from eyes. Specific rotation values were measured by SCHMIDT+HAENSCH POLARTRONIC MH8. The samples were solved into a 1 mL volumetric flask and then injected into a sample tube (*l* = 1 dm). The temperature is 20 ^{o}C and the wavelength of the light is 589.44 nm. The specific rotation $[\boldsymbol{\alpha}]_{\mathbf{D}}^{\mathbf{20}} = \frac{100\alpha}{c*l}$ and the unit of specific rotation is deg·mL·g^{-1}·dm^{-1}. The concentration c = $\frac{m*100}{1}$, and it's unit is g/100 mL.

2. General computational details

We have used the Density Functional Theory (DFT) in the Kohn-Sham formulation[1] to optimize all the stationary points presented in this manuscript. Geometries of all the stationary points were fully optimized at the B3LYP-D3/def2svpp[2] computational level. The effect of solvent (acetonitrile) was modelled using the polarizable continuum model (PCM)[3] with the default parameters implemented in the Gaussian 16 package.[4] All geometry optimizations have been performed using tight convergence criteria in the SCF and requesting

a pruned (99.590) grid to guarantee the accuracy of the reported results. Moreover, calculations were performed using the default parameters implemented in the Gaussian suite. The conditions of temperature and pressure used for the computations were 1 atm and 298 K, to simulate accurately the reaction conditions.

Harmonic analysis was used to establish the nature of all optimized structures as either minima or transition state structures. For all stationary points, the stability of the wave function was also confirmed.[5]

IRC calculations[6] were conducted for transition states to ensure their connectivity with the expected reactants and products. The stability of the wave function was analysed for all the presented stationary points.

The visualization of the reported structures was performed using MOLDEN. The representation of the structures here presented were generated using ChemDraw.

3. Synthesis of substrates

3.1 Synthesis of phosphines

1[7] and **5**[7] [8] are known compounds.

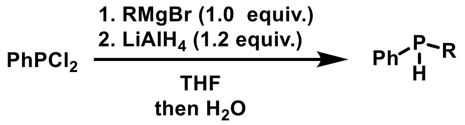

General preparation for HPPhR (**1a, 5a-5c**): A dried 100 mL Schlenk tube equipped with a magnetic stirring bar was charged with $PhPCl_2$ (1.7 mL, 12.5 mmol, 1.0 equiv.) and THF (20 mL) under N_2 atmosphere. After the mixture was cooled to -78 °C, Grignard reagent RMgBr (12.5 mmol, 1.0 equiv, in 12.5 mL THF) was added dropwise. Then the reaction mixture was stirred at -78 °C for 2 hours. After allowing mixture to warm up to 0 °C, $LiAlH_4$ (15 mL, 1.2 equiv, 1M in THF) was added dropwise. Then the resulting reaction mixture was stirred at room temperature for 2 hours. After that, degassed H_2O (8 mL) was added dropwise (Note: careful). Then the mixture was dried with $MgSO_4$ and filtered to give a clear solution. The solids were washed with anhydrous Et_2O (20 mL) for three times. The organic layers were combined and evaporated quickly under reduced pressure to give crude product. The crude was purified by distillation under reduced pressure giving the pure secondary phosphine. These phosphines were stored in a glovebox (to prevent oxidation) for further use.

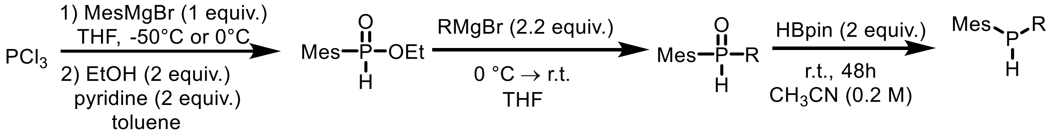

General preparation for HPMesR (5d-5f):

Step 1: Synthesis of ethyl mesitylphosphinate: A dried 100 mL Schlenk tube equipped with a magnetic stirring bar was charge with PCl_3 (0.87 ml, 10 mmol, 1 equiv.) and THF (10 mL) under N2 atmosphere. After the mixture was cooled to -50 °C (0 °C is also fine), Grignard reagent MesMgBr (10 mmol in 10 mL THF, 1equiv.) was added dropwise. Then the reaction mixture was stirred at -50 °C (0 °C is also fine) for 2-4 hours. After removing the solvent under vacuum, EtOH (20 mmol, 1.16 mL, 2 equiv.), pyridine (20 mmol, 1.61 mL, 2 equiv.) and toluene were added under air. The reaction mixture was stirred at room temperature overnight. Water (20 mL) was then added and the aqueous phase was extracted with EtOAc. The organic fraction

was dried over $MgSO_4$, concentrated under vacuum and purified via column chromatography to afford mesitylphosphinate.

Step 2: Synthesis of secondary phosphine oxides: A dried 100 mL Schlenk tube equipped with a magnetic stirring bar was charged the corresponding Grignard reagent (2.2 equiv.) in THF and cooled to 0 °C, mesitylphosphinate (5 mmol, 1 equiv.) in THF (5 mL) was added dropwise. The reaction was stirred at 0 °C for 4 hours then quenched with saturated aqueous NH_4Cl solution. The aqueous phase was extracted with EtOAc. The organic fraction was dried over $MgSO_4$, concentrated under vacuum and purified vai column chromatography to afford the desired secondary phosphine oxide.

Step 3: Reduction of secondary phosphine oxide: A 4 mL vial equipped with stirring bar was charged the secondary phosphine oxide (0.2 mmol, 1 equiv.). After transferring the vial into the glove box, HBpin (2 equiv.) and CH_3CN (1 mL, 0.2 M) were added under N2. The reaction mixture was stirred at room temperature in the glove box for 48-96 hours until the phosphine oxide was fully converted. Then solution was used directly without purification. (Note: for mesityl(methyl)phosphine oxide, 0.1 mL isopropanol was added to quench the excess HBpin)

3.2 Synthesis of (alkyl)halides

Halides **2a**-**2l**, **2n**, **2o**, **3a**-**3h** and **4a**-**4e** are commercially available.

2m was synthesized according to the literature procedure A[9].

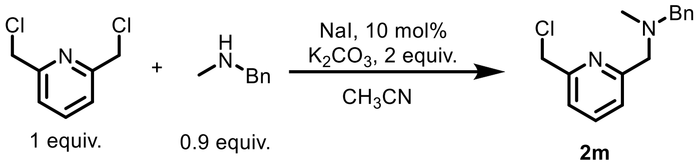

Procedure A: A 100 mL round flask equipped with a magnetic stirring bar was charged with 2,6-bis(chloromethyl)pyridine (1 equiv.), NaI (10 mol%), K_2CO_3 (2 equiv.) and CH_3CN (0.4 M) under air. After the mixture was cooled with ice bath, N-methyl-1-phenylmethanamine (0.9 equiv.) was added dropwise. Then the reaction mixture was stirred under room temperature for 16 h. The resulting mixture was filtered through a filter paper and the filtrate was concentrated under vacuum. The crude was purified by silica gel chromatography (pentane/ ethyl acetate = 5:1).

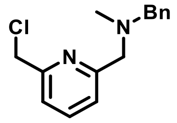

N-benzyl-1-(6-(chloromethyl)pyridin-2-yl)-N-methylmethanamine (2m)

210 mg, 30% yield, yellow liquid.

^{1}H NMR (400 MHz, $CDCl_3$) δ 7.70 (t, *J* = 7.7 Hz, 1H), 7.50 (d, *J* = 7.8 Hz, 1H), 7.42 – 7.21 (m, 6H), 4.66 (s, 2H), 3.72 (s, 2H), 3.62 (s, 2H), 2.27 (s, 3H).

^{13}C NMR (101 MHz, CDCl3) δ 155.95, 137.62, 129.20, 128.44, 127.32, 122.45, 121.20, 63.06, 62.07, 46.96, 42.56.

HRMS (ESI, m/Z): calcd. for $C_{15}H_{18}ClN_2^+$ $[M+H]^+$: 263.1124, found: 263.1117.

Note: This compound should be stored under the low temperature and inert atmosphere.

4. General procedure for the S_N2 reaction

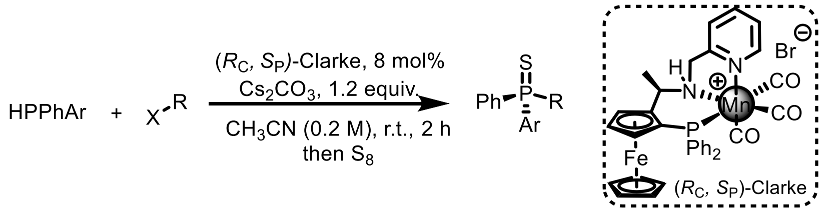

A 4 ml vial equipped with a magnetic stirring bar was charged with (R_C, S_P)-Clarke catalyst (11.6 mg, 0.016 mmol, 0.08 equiv.) and Cs_2CO_3 (78 mg, 0.24 mmol, 1.2 equiv.). Then the vial was transferred to the glove box. HPPhAr (0.2 mmol, 1.0 equiv.), halide (0.24 mmol, 1.2 equiv.) and Anhydrous CH_3CN (1 mL) were added into the vial. The resulting mixture was stirred at room temperature for 2 hours. Then the reaction mixture was subjected to a flush column to remove the base and the catalyst. After removing the solvent under reduced pressure, the residue was dissolved in $CDCl_3$ and protected with S_8 (14 mg, stirred for 30- 60 minutes). The reaction progress was monitored via 31P-NMR spectroscopy, upon completion, the crude was purified by a silica gel column chromatography (pentane/diethyl ether = 50/1-10/1) to give the desired product.

The racemic products were obtained through the same procedure with racemic Clarke catalyst.

5. Computational mechanistic studies

In order to elucidate the origin of the enantiodiscrimination, we resorted to molecular modelling. We started our computational investigation by exploring the two main conformers of the complex resulting from the complexation of the Clarke catalyst with the phosphine, before and after base deprotonation (see **Figure S1**). We have found that while both conformations are only 0.73 kcal/mol separated in energy when the phosphine is protonated, this difference in energy gets aggravated when they become deprotonated (3.46 kcal/mol). Note that while when they are protonated, at **I** and **II,** the pyridine moiety stablishes a π-π interaction with the phenyl or mesyl groups, when deprotonation occurs this interaction is lost in the case of **IV**. This conformer (**IV**) evolves to establish a stabilizing CH- π interaction between the pyridine and the phenyl group.

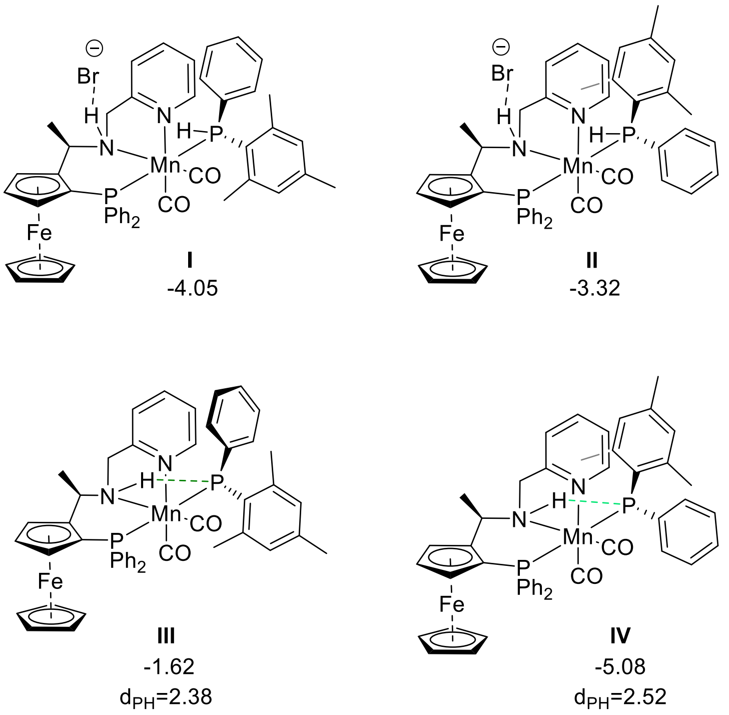

Figure S1. Analysis of the conformers of **I** to **IV**. d_{PH} denotes the bond distance between the phosphorous and hydrogen atoms.

Having identified the most stable form of the phosphide-Mn complex, we moved to explore the addition of the alkyl halide to these complexes. We have found that the addition of the alkyl halide to **III** is kinetically and thermodynamically preferred, with an energy barrier of 3.36 kcal/mol and an energy release of 35.25 kcal/mol. This preference is caused by the orientation of the phenyl group that allows for a better accommodation of the electrophile core and a stabilizing π-π interaction between the phenyl groups of the phosphine (at the manganese complex) and the phenyl group of the organic halide. This interaction cannot be established at the diastereomeric transition state (**TS-IV-V**) and, more importantly, at **IV** the lone pair of the phosphorous atom has a strong interaction with the hydrogen of the amino group, this interaction turns it overall into a less nucleophilic center wrt the phosphorous group at **III**, and it difficult the accommodation of the substrate, resulting in a greater energy penalization (Figure S2).

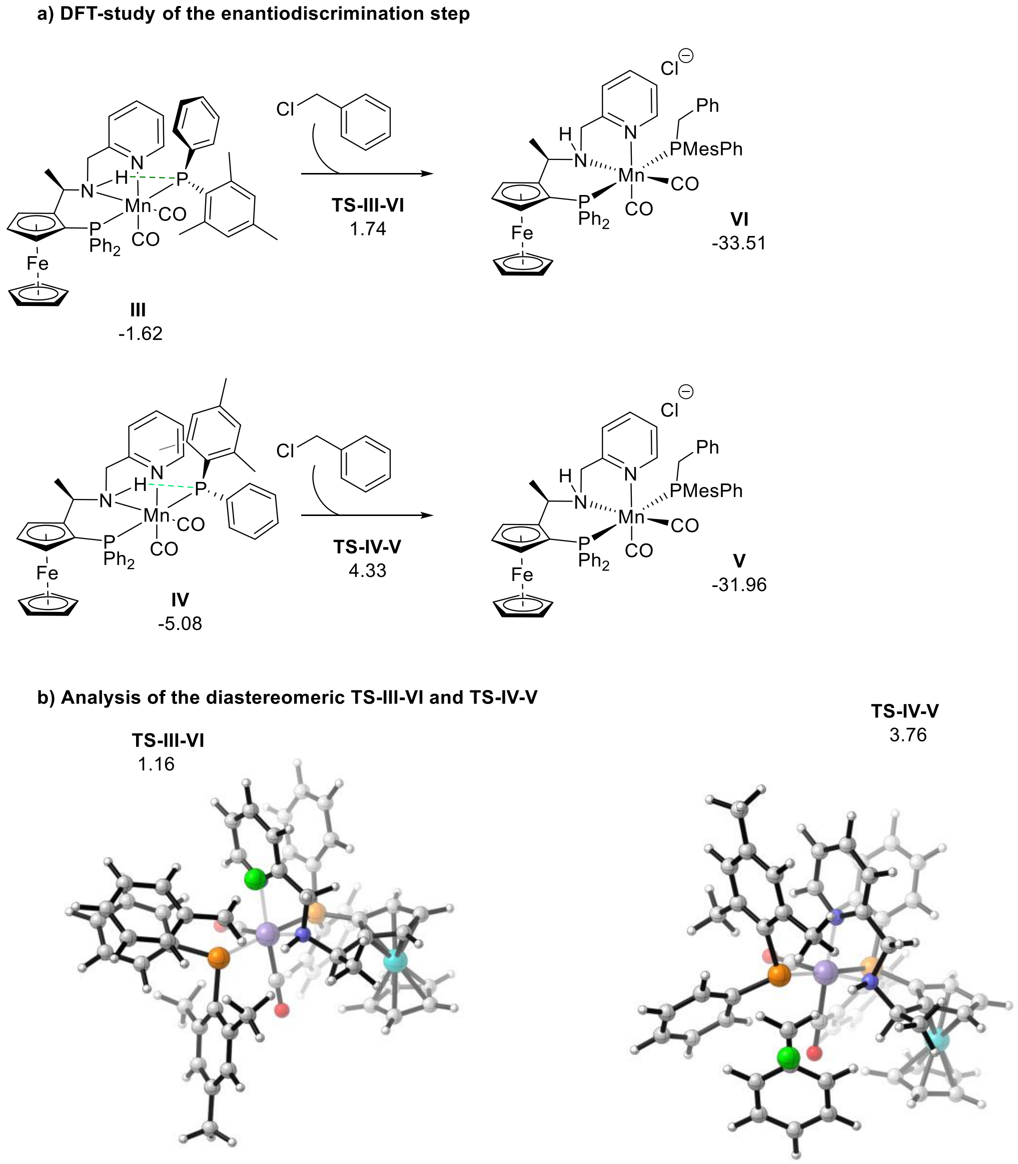

Figure S2. Analysis of the a) enantiodiscrimination step and b) diastereomeric transition states **TS-III-VI** and **TS-IV-V**.

6. Determination of the absolute configuration

The absolute configuration of **2'a** and **3'c** was determined by X-ray crystallographic analysis. The absolute configuration of other compounds was assigned by analogy.

Compound **2'a** (around 50 mg) was dissolved in 2 mL vial with a mixture of diethyl ether/pentane. The single crystal was grown by slow evaporation of solvents at room temperature. CCDC 2357463 contains the supplementary crystallographic data of **2'a**.

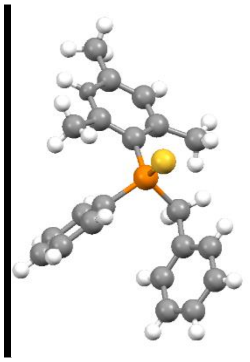

Table 1. Crystal data and structure refinement for CCDC 2357463

chem. formula	C22H23PS
Mr	350.43
cryst syst.	orthorhombic
color, habit	colourless, plate
size (mm)	0.452 x 0.255 x 0.087
space group	P 21 21 21
a (Å)	7.7591(9)
b (Å)	15.4236(17)
c (Å)	16.3734(18)
α, deg	90
β, deg	90

γ, deg	90
V (Å3)	1959.5(4)
Z	4
ρcalc, g.cm-3	1.188
μ(Cu Kα), cm-1	2.213
F(000)	744
temp (K)	273(2)
θ range (deg)	3.937 - 72.334
data collected (h,k,l)	-9:9, -19:19, -20:20
no. of rflns collected	46649
no. of indpndt reflns	3877
observed reflns (Fo ⩾ 2 σ(Fo))	3801
R(F) (%)	2.54
wR(F2) (%)	7.09
GooF	1.052
Weighting a,b	0.0504, 0.1880
params refined	221
restraints	0
min, max resid dens	-0.158, 0.173
Flack x	0.047(4)

Note: Disorder in the benzyl part of the crystal structure was detected but splitting of the affected atoms did not improve the model and was therefore discarded.

Compound **3’c** (around 30 mg) was dissolved in 2 mL vial by diethyl ether/pentane. The single crystal was grown by slow evaporation of solvents at room temperature. CCDC 2357464 contains the supplementary crystallographic data of **3’c**.

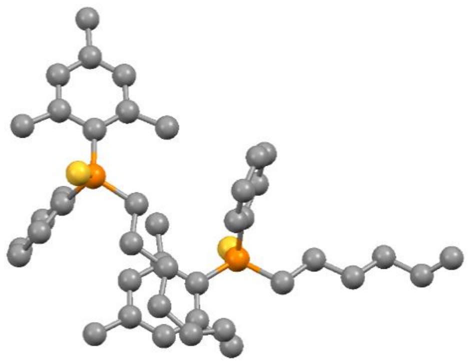

Table 2. Crystal data and structure refinement for CCDC 2357464

chem. formula	C21H27PS
Mr	342.45
cryst syst.	monoclinic
color, habit	colorless, block
size (mm)	0.522x0.395x0.223
space group	P 21
a (Å)	8.6267(8)
b (Å)	19.0245(18)
c (Å)	11.5103(12)
α, deg	90
β, deg	90.853(3)
γ, deg	90
V (Å3)	1888.8(3)

Z	4
ρcalc, g.cm-3	1.204
μ(Cu Kα), cm-1	0.254
F(000)	736
temp (K)	100(2)
θ range (deg)	2.930 – 26.173
data collected (h,k,l)	-10:10, -23:23, -14:14
no. of rflns collected	22333
no. of indpndt reflns	7032
observed reflns (Fo ⩾ 2 σ(Fo))	6793
R(F) (%)	6
wR(F2) (%)	16.08
GooF	1.088
Weighting a,b	0.1030, 2.6970
params refined	421
restraints	1
min, max resid dens	-0.597, 0.918
Flack x	0.14(3)

Note: Large reflection outliers affected by the Beamstop were omitted from the refinement.

7. Characterization of the products

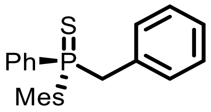

(*S*)-benzyl(mesityl)(phenyl)phosphine sulfide (2’a)

63 mg, 90% yield, white solid

^{1}H NMR (400 MHz, $CDCl_3$) δ 7.48 – 7.39 (m, 2H), 7.39 – 7.32 (m, 1H), 7.28 – 7.19 (m, 2H), 7.11 – 7.03 (m, 1H), 7.03 – 6.95 (m, 2H), 6.89 (d, *J* = 4.0 Hz, 2H), 6.84 – 6.74 (m, 2H), 4.04 (dd, *J* = 15.9, 13.0 Hz, 1H), 3.72 (dd, *J* = 13.0, 10.6 Hz, 1H), 2.36 (s, 6H), 2.30 (s, 3H).

^{13}C NMR (101 MHz, $CDCl_3$) δ 141.72 (d, *J* = 10.1 Hz), 141.00 (d, *J* = 2.9 Hz), 134.03 (d, *J* = 80.0 Hz), 131.52 (d, *J* = 11.1 Hz), 131.29 (d, *J* = 7.4 Hz), 130.88 (d, *J* = 6.2 Hz), 130.80 (d, *J* = 1.9 Hz), 130.75 (d, *J* = 3.0 Hz), 129.42 (d, *J* = 80.5 Hz), 128.07 (d, *J* = 12.4 Hz), 127.48 (d, *J* = 3.4 Hz), 126.73 (d, *J* = 4.1 Hz), 45.78 (d, *J* = 47.1 Hz), 24.51 (d, *J* = 4.7 Hz), 21.01 (d, *J* = 1.3 Hz).

^{31}P NMR (162 MHz, $CDCl_3$) δ 43.26.

HRMS (ESI, m/Z): calcd. for $C_{22}H_{24}PS^+$ $[M+H]^+$: 351.1331, found:351.1326.

Melting point: 125.5 ^oC

Specific rotation: $[\alpha]_D^{20}$= -155.31 (c = 2.821 g/100mL, $CHCl_3$).

SFC: Chiralcel OD, CO_2/MeOH with gradient from 97% to 50% CO_2 in 4.5 min, 1.8 mL/min., 40 ^oC, detection at 221 nm. Retention time: 2.56 min. (major) and 2.81min. (minor). 90% *ee.*

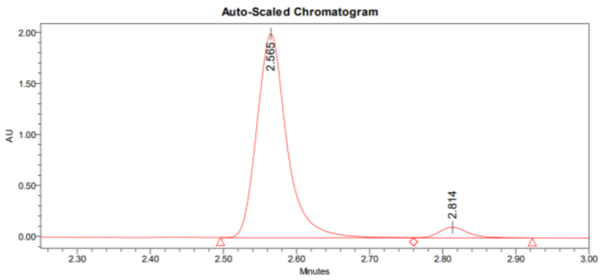

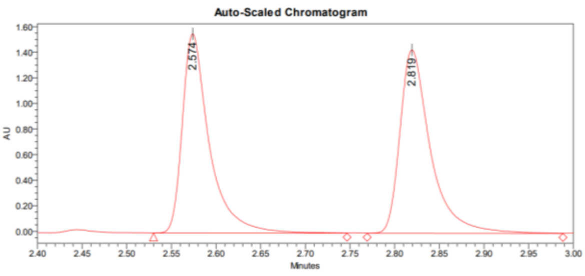

Peak Results

	Name	RT	Area	% Area	Height
1		2.565	5665689	95.14	1998148
2		2.814	289653	4.86	104369

Peak Results

	Name	RT	Area	% Area	Height
1		2.574	3287378	49.11	1558174
2		2.819	3406184	50.89	1434069

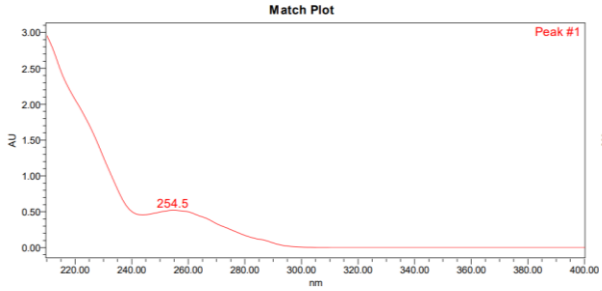

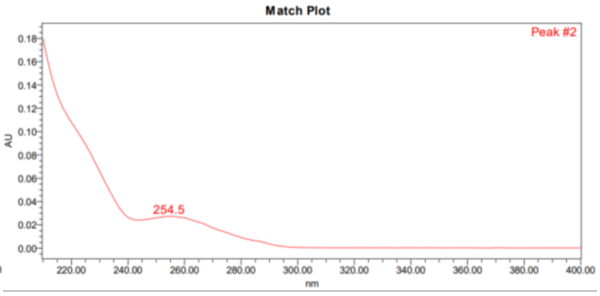

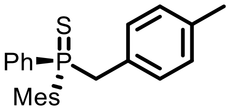

(*S*)-mesityl(4-methylbenzyl)(phenyl)phosphine sulfide (2’b)

65.2 mg, 90% yield, white solid

^{1}H NMR (400 MHz, $CDCl_3$) δ 7.51 – 7.41 (m, 2H), 7.41 – 7.32 (m, 1H), 7.31 – 7.20 (m, 2H), 6.88 (d, *J* = 3.9 Hz, 2H), 6.81 (d, *J* = 7.8 Hz, 2H), 6.64 (dd, *J* = 8.1, 2.7 Hz, 2H), 4.00 (dd, *J* = 15.7, 13.0 Hz, 1H), 3.70 (dd, *J* = 13.0, 10.4 Hz, 1H), 2.36 (d, *J* = 1.1 Hz, 6H), 2.30 (s, 3H), 2.21 (d, *J* = 2.6 Hz, 3H).

^{13}C NMR (101 MHz, $CDCl_3$) δ 141.68 (d, *J* = 10.0 Hz), 140.90 (d, *J* = 2.8 Hz), 136.31 (d, *J* = 4.3 Hz), 134.12 (d, *J* = 80.0 Hz), 131.47 (d, *J* = 11.1 Hz), 130.93 (d, *J* = 9.9 Hz), 130.68 (d, *J* = 5.2 Hz), 130.67, 129.48 (d, *J* = 79.7 Hz), 128.20 (d, *J* = 3.5 Hz), 128.03 (d, *J* = 12.2 Hz), 127.95(d, *J* = 8.0 Hz), 45.26 (d, *J* = 47.1 Hz), 24.50 (d, *J* = 4.8 Hz), 21.12 (d, *J* = 1.6 Hz), 20.98 (d, *J* = 1.5 Hz).

^{31}P NMR (162 MHz, $CDCl_3$) δ 43.23.

HRMS (ESI, m/Z): calcd. for $C_{23}H_{26}PS^+$ $[M+H]^+$: 365.1487, found: 365.1483.

Melting point: 140.6 ^{o}C

Specific rotation: $[\alpha]_{\mathbf{D}}^{\mathbf{20}}$= -243.68 (c = 4.405 g/100mL, $CHCl_3$).

SFC: Chiralcel OD, CO_2/MeOH with gradient from 97% to 50% CO_2 in 4.5 min, 1.8 mL/min., 40 ^{o}C, detection at 221 nm. Retention time: 2.59 min. (major) and 2.79 min. (minor). 94% *ee*.

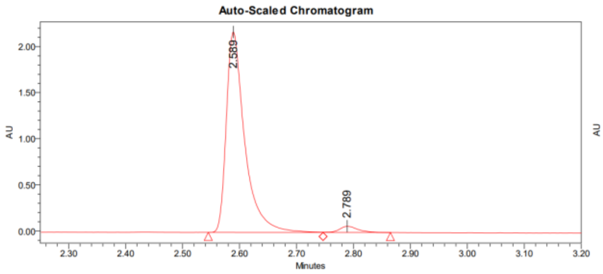

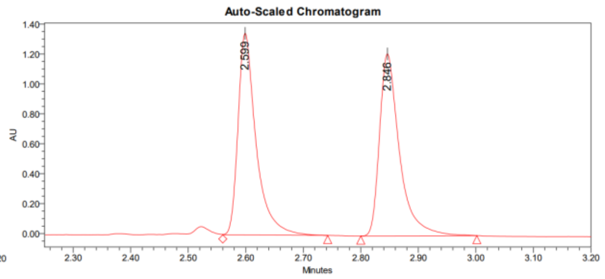

Peak Results

	Name	RT	Area	% Area	Height
1		2.589	4889263	96.96	2172928
2		2.789	153243	3.04	67094

Peak Results

	Name	RT	Area	% Area	Height
1		2.599	2940958	50.09	1348064
2		2.846	2930052	49.91	1216679

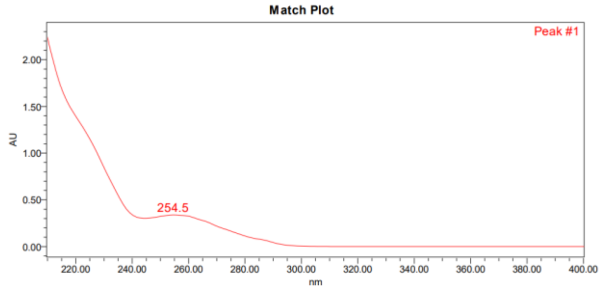

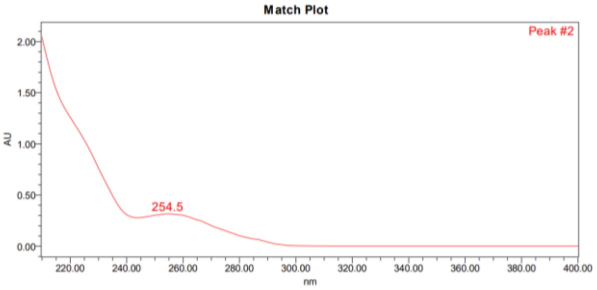

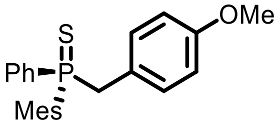

(*S*)-mesityl(4-methoxybenzyl)(phenyl)phosphine sulfide (2’c)

53.5 mg, 70% yield, white solid

^{1}H NMR (400 MHz, $CDCl_3$) δ 7.48 – 7.40 (m, 2H), 7.36 (td, *J* = 7.2, 1.7 Hz, 1H), 7.29 – 7.20 (m, 2H), 6.88 (d, *J* = 3.9 Hz, 2H), 6.71 – 6.62 (m, 2H), 6.58 – 6.50 (m, 2H), 3.97 (dd, *J* = 15.3, 13.1 Hz, 1H), 3.73 – 3.62 (m, 4H), 2.35 (s, 6H), 2.29 (s, 3H).

^{13}C NMR (101 MHz, $CDCl_3$) δ 158.56 (d, *J* = 3.9 Hz), 141.70 (d, *J* = 9.9 Hz), 140.92 (d, *J* = 2.9 Hz), 134.16 (d, *J* = 79.8 Hz), 131.79 (d, *J* = 5.4 Hz), 131.46 (d, *J* = 11.0 Hz), 130.88 (d, *J* = 9.7 Hz), 130.70 (d, *J* = 3.0 Hz), 129.46 (d, *J* = 79.0 Hz), 128.07 (d, *J* = 12.2 Hz), 123.03 (d, *J* = 7.5 Hz), 112.97 (d, *J* = 3.3 Hz), 55.20, 44.90 (d, *J* = 47.3 Hz), 24.50 (d, *J* = 4.5 Hz), 20.98 (d, *J* = 1.4 Hz).

^{31}P NMR (162 MHz, $CDCl_3$) δ 43.42.

HRMS (ESI, m/Z): calcd. for $C_{23}H_{26}OPS^+$ $[M+H]^+$: 381.1436, found: 381.1432.

Melting point: 168.3 °C

Specific rotation: $[\alpha]_D^{20}$= -235.24 (c = 2.821 g/100mL, $CHCl_3$).

SFC: Chiralcel OD, CO_2/MeOH with gradient from 97% to 50% CO_2 in 4.5 min, 1.8 mL/min., 40 °C, detection at 221 nm. Retention time: 2.63 min. (minor) and 2.96 min. (major). 90% *ee*.

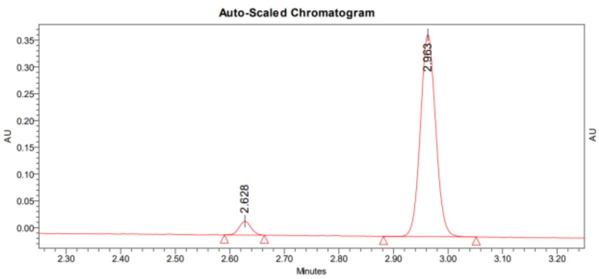

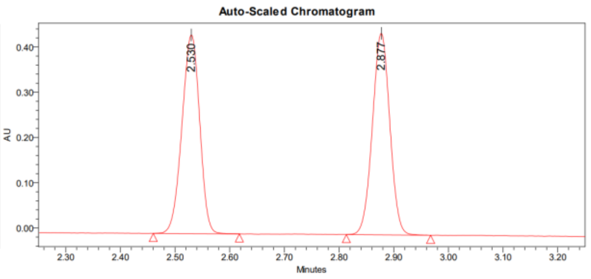

Peak Results

	Name	RT	Area	% Area	Height
1		2.628	39328	5.08	25757
2		2.963	735316	94.92	377133

Peak Results

	Name	RT	Area	% Area	Height
1		2.530	988796	50.01	438991
2		2.877	988223	49.99	445331

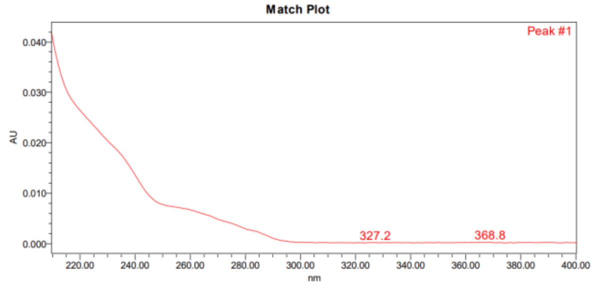

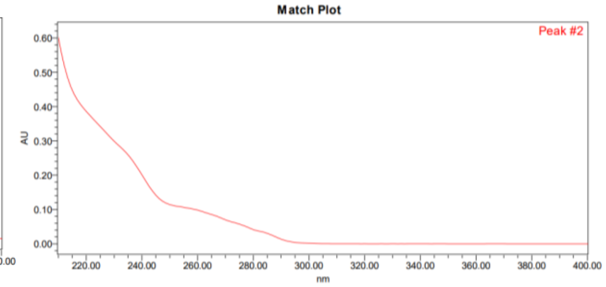

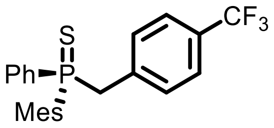

(*S*)-mesityl(phenyl)(4-(trifluoromethyl)benzyl)phosphine sulfide (2'd)

74.7 mg, 89% yield, white solid

^{1}H NMR (400 MHz, $CDCl_3$) δ 7.48 – 7.33 (m, 3H), 7.29 – 7.20 (m, 4H), 6.92 – 6.84 (m, 4H), 4.07 (dd, *J* = 16.2, 12.8 Hz, 1H), 3.75 (dd, *J* = 12.8, 10.9 Hz, 1H), 2.35 (s, 6H), 2.30 (s, 3H).

^{13}C NMR (101 MHz, $CDCl_3$) δ 141.66 (d, *J* = 10.2 Hz), 141.27 (d, *J* = 3.0 Hz), 135.59 (d, *J* = 7.8 Hz), 133.64 (d, *J* = 80.2 Hz), 131.59 (d, *J* = 11.3 Hz), 131.05 (d, *J* = 3.0 Hz), 130.98 (d, *J* = 5.4 Hz), 130.72 (d, *J* = 9.9 Hz), 128.90 (d, *J* = 81.8 Hz), 128.90 (dd, *J* = 32.4, 3.9 Hz), 128.23 (d, *J* = 12.3 Hz), 124.27 (dd, *J* = 270.4 Hz, 1.6Hz), 124.19 (q, *J* = 3.8 Hz), 45.66 (d, *J* = 46.6 Hz), 24.44 (d, *J* = 4.9 Hz), 20.98 (d, *J* = 1.5 Hz).

^{31}P NMR (162 MHz, $CDCl_3$) δ 42.83 (q, *J* = 2.8 Hz).

^{19}F NMR (376 MHz, $CDCl_3$) δ -62.55.

HRMS (ESI, m/Z): calcd. for $C_{23}H_{23}F_3PS^+$ $[M+H]^+$: 419.1205, found: 419.1198.

Melting point: 181.8 °C

Specific rotation: $[\alpha]_D^{20}$= -136.20 (c = 4.445 g/100mL, $CHCl_3$).

SFC Chiralcel OD, CO_2/MeOH with gradient from 97% to 50% CO_2 in 4.5 min, 1.8 mL/min., 40 °C, detection at 221 nm. Retention time: 2.08 min. (major) and 2.81min. (minor). 93% *ee*.

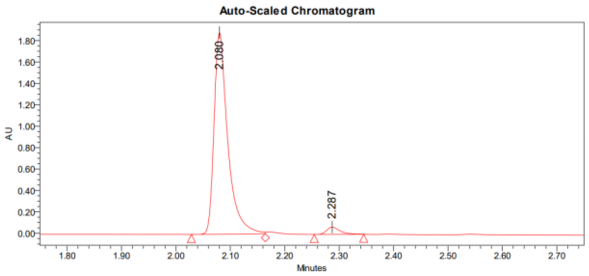

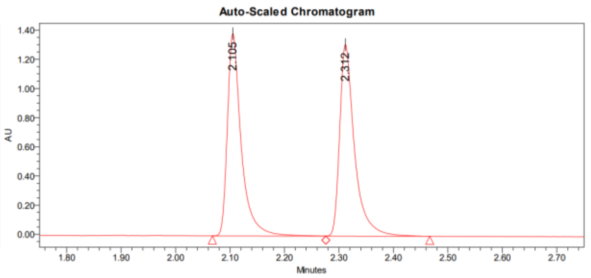

Peak Results

	Name	RT	Area	% Area	Height
1		2.080	3294580	96.63	1882317
2		2.287	115035	3.37	68392

Peak Results

	Name	RT	Area	% Area	Height
1		2.105	2449799	49.60	1388104
2		2.312	2489241	50.40	1314624

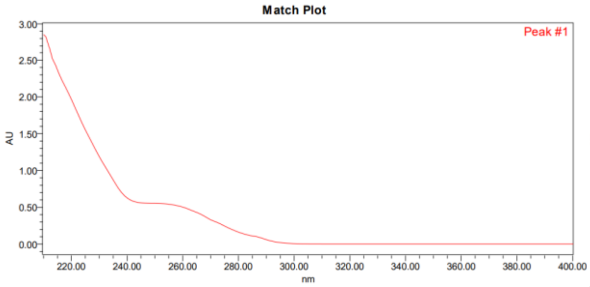

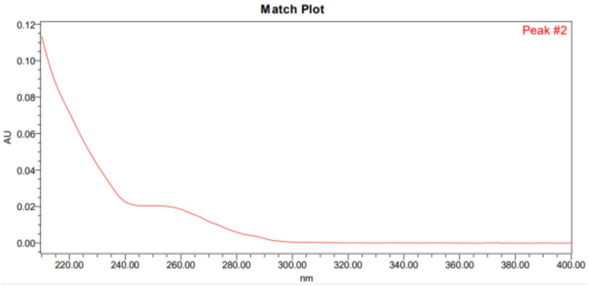

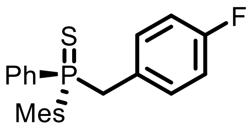

(*S*)-(4-fluorobenzyl)(mesityl)(phenyl)phosphine sulfide (2’e)

70.5 mg, 96% yield, white solid

^{1}H NMR (400 MHz, $CDCl_3$) δ 7.47 – 7.32 (m, 3H), 7.31 – 7.20 (m, 2H), 6.88 (d, *J* = 4.0 Hz, 2H), 6.78 – 6.62 (m, 4H), 3.99 (dd, *J* = 15.6, 13.1 Hz, 1H), 3.68 (dd, *J* = 13.1, 10.3 Hz, 1H), 2.35 (s, 6H), 2.29 (s, 3H).

^{13}C NMR (101 MHz, $CDCl_3$) δ 161.99 (dd, *J* = 245.5, 4.4 Hz), 141.66 (d, *J* = 10.2 Hz), 141.08 (d, *J* = 2.9 Hz), 133.88 (d, *J* = 80.0 Hz), 132.18 (dd, *J* = 8.0, 5.4 Hz), 131.51 (d, *J* = 11.1 Hz), 130.85 (d, *J* = 3.2 Hz), 130.77 (d, *J* = 9.7 Hz), 129.15 (d, *J* = 80.4 Hz), 128.16 (d, *J* = 12.2 Hz), 126.94 (dd, *J* = 7.4, 3.3 Hz), 114.33 (dd, *J* = 21.4, 3.5 Hz), 44.90 (d, *J* = 47.3 Hz), 24.45 (d, *J* = 4.8 Hz), 20.97 (d, *J* = 1.7 Hz).

^{31}P NMR (162 MHz, $CDCl_3$) δ 43.24 (d, *J* = 6.6 Hz).

^{19}F NMR (376 MHz, $CDCl_3$) δ -115.93 – -116.13 (m).

For comparison, ^{19}F NMR of starting material **2e** is provided: **^{19}F NMR** (377 MHz, $CDCl_3$) δ -110.44 – -116.81 (m).

HRMS (ESI, m/Z): calcd. for $C_{22}H_{23}FPS^+$ $[M+H]^+$: 369.1237, found: 369.1230.

Melting point: 167.4 °C

Specific rotation: $[\alpha]_D^{20}$= -201.56 (c = 5.653 g/100mL, $CHCl_3$).

SFC Chiralcel OD, CO_2/MeOH with gradient from 97% to 50% CO_2 in 4.5 min, 1.8 mL/min., 40 °C, detection at 221 nm. Retention time: 2.30 min. (major) and 2.49 min. (minor). 94% *ee*.

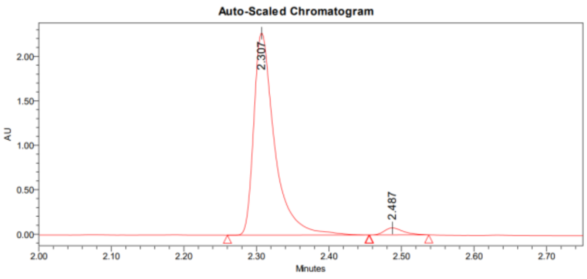

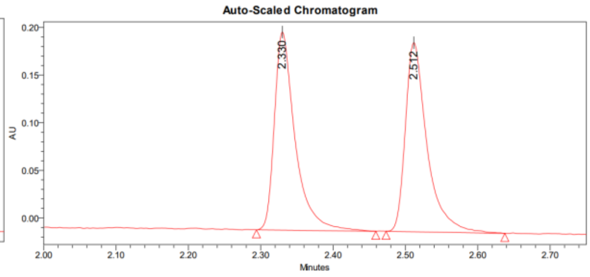

Peak Results

	Name	RT	Area	% Area	Height
1		2.307	4551882	96.93	2273680
2		2.487	144395	3.07	78792

Peak Results

	Name	RT	Area	% Area	Height
1		2.330	414632	50.07	207885
2		2.512	413405	49.93	198456

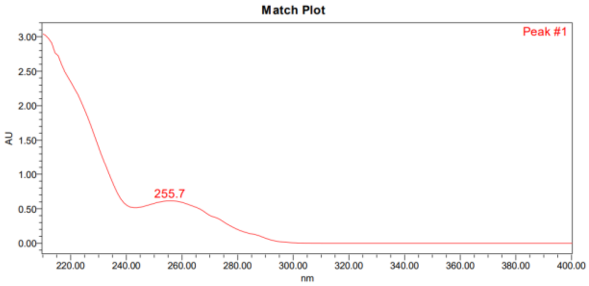

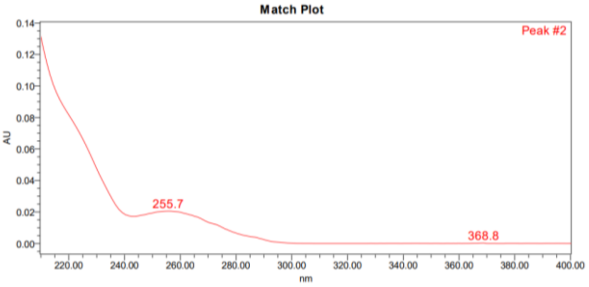

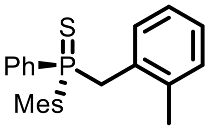

(*S*)-mesityl(2-methylbenzyl)(phenyl)phosphine sulfide (2’f)

71.1 mg, 99% yield, white solid

^{1}H NMR (400 MHz, $CDCl_3$) δ 7.57 – 7.46 (m, 2H), 7.45 – 7.35 (m, 1H), 7.32 – 7.22 (m, 2H), 7.04 – 6.99 (m, 1H), 6.96 (d, *J* = 7.4 Hz, 1H), 6.90 (d, *J* = 4.0 Hz, 2H), 6.84 (t, *J* = 7.4 Hz, 1H), 6.70 (ddd, *J* = 7.7, 2.8, 1.3 Hz, 1H), 4.19 (dd, *J* = 15.9, 13.5 Hz, 1H), 3.80 (dd, *J* = 13.5, 11.2 Hz, 1H), 2.38 (s, 6H), 2.31 (s, 3H), 2.10 (d, *J* = 1.5 Hz, 3H).

^{13}C NMR (101 MHz, $CDCl_3$) δ 141.74 (d, *J* = 10.2 Hz), 140.87 (d, *J* = 2.9 Hz), 138.03 (d, *J* = 6.1 Hz), 134.42 (d, *J* = 78.9 Hz), 131.61 (d, *J* = 11.1 Hz), 131.16 (d, *J* = 9.7 Hz), 131.09 (d, *J* = 9.1 Hz), 130.89 (d, *J* = 3.0 Hz), 130.20 (d, *J* = 3.3 Hz), 130.17 (d, *J* = 8.1 Hz), 129.40 (d, *J* = 79.9 Hz), 128.05 (d, *J* = 12.1 Hz), 126.99 (d, *J* = 3.8 Hz), 125.00 (d, *J* = 3.6 Hz), 41.48 (d, *J* = 47.9 Hz), 24.64 (d, *J* = 4.5 Hz), 20.98 (d, *J* = 1.5 Hz), 20.63 (d, *J* = 1.7 Hz).

^{31}P NMR (162 MHz, $CDCl_3$) δ 43.11.

HRMS (ESI, m/Z): calcd. for $C_{23}H_{26}PS^+$ $[M+H]^+$: 365.1487, found: 365.1481.

Melting point: 110.0 °C

Specific rotation: $[\alpha]_{\mathrm{D}}^{20}$= -162.36 (c = 3.735 g/100mL, $CHCl_3$).

SFC Chiralcel OD, CO_2/MeOH with gradient from 97% to 50% CO_2 in 4.5 min, 1.8 mL/min., 40 °C, detection at 221 nm. Retention time: 2.51 min. (major) and 2.75 min. (minor). 90% *ee*.

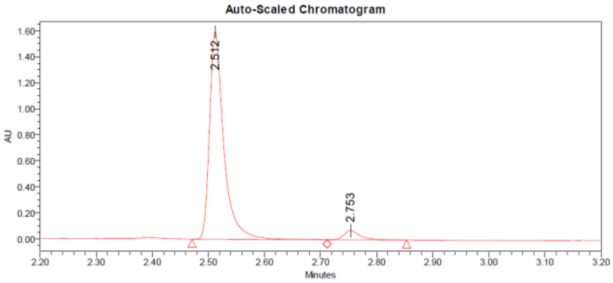

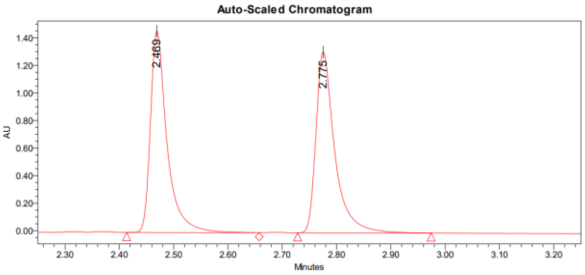

Peak Results

	Name	RT	Area	% Area	Height
1		2.512	2825535	95.20	1595507
2		2.753	142323	4.80	70383

Peak Results

	Name	RT	Area	% Area	Height
1		2.469	3103883	49.54	1461661
2		2.775	3160928	50.46	1312520

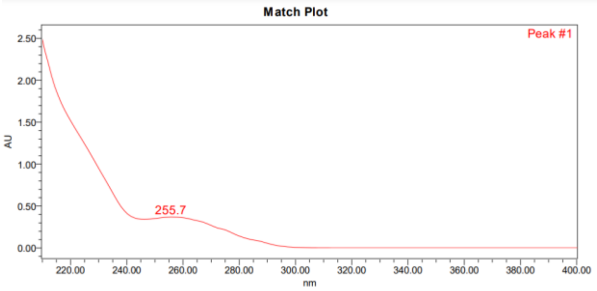

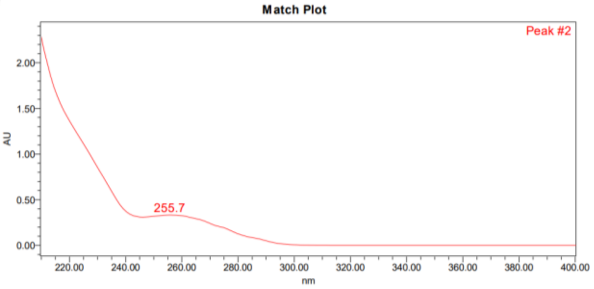

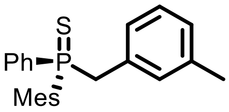

(*S*)-mesityl(3-methylbenzyl)(phenyl)phosphine sulfide (2’g)

70 mg, 96% yield, white solid

^{1}H NMR (400 MHz, $CDCl_3$) δ 7.49 – 7.40 (m, 2H), 7.37 (td, *J* = 7.3, 1.7 Hz, 1H), 6.96 – 6.84 (m, 4H), 6.69 – 6.61 (m, 1H), 6.44 (s, 1H), 3.99 (dd, *J* = 15.8, 12.9 Hz, 1H), 3.68 (dd, *J* = 12.9, 10.6 Hz, 1H), 2.37 (s, 6H), 2.30 (s, 3H), 2.09 (s, 3H).

^{13}C NMR (101 MHz, $CDCl_3$) δ 141.65 (d, *J* = 10.2 Hz), 140.90 (d, *J* = 2.9 Hz), 136.87 (d, *J* = 3.6 Hz), 134.15 (d, *J* = 80.0 Hz), 131.68 (d, *J* = 5.5 Hz), 131.46 (d, *J* = 11.1 Hz), 131.02, 130.89 (d, *J* = 9.9 Hz), 130.63 (d, *J* = 3.1 Hz), 129.41 (d, *J* = 80.2 Hz), 127.93, 127.93 (d, *J* = 12.3 Hz), 127.37 (d, *J* = 4.0 Hz), 127.26 (d, *J* = 3.5 Hz), 45.75 (d, *J* = 47.0 Hz), 24.48 (d, *J* = 4.8 Hz), 21.17, 20.96 (d, *J* = 1.4 Hz).

^{31}P NMR (162 MHz, $CDCl_3$) δ 43.28.

HRMS (ESI, m/Z): calcd. for $C_{23}H_{26}PS^+$ $[M+H]^+$: 365.1487, found: 365.1482.

Melting point: 118.5 °C

Specific rotation: $[\alpha]_D^{20}$= -187.87 (c = 1.006 g/100mL, $CHCl_3$).

SFC Chiralcel OD, CO_2/MeOH with gradient from 97% to 50% CO_2 in 4.5 min, 1.8 mL/min., 40 °C, detection at 221 nm. Retention time: 2.46 min. (major) and 2.70 min. (minor). 91% *ee*.

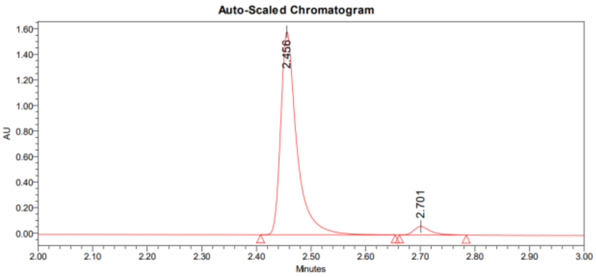

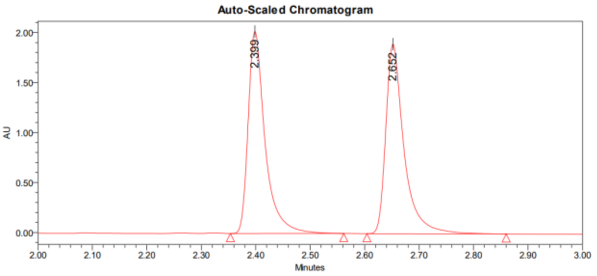

Peak Results

	Name	RT	Area	% Area	Height
1		2.456	3262603	95.85	1589523
2		2.701	141271	4.15	65918

Peak Results

	Name	RT	Area	% Area	Height
1		2.399	4361845	49.75	2020816
2		2.652	4406334	50.25	1896097

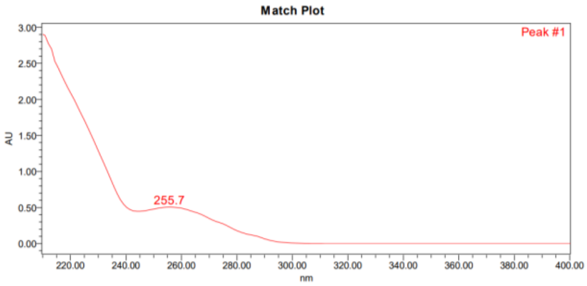

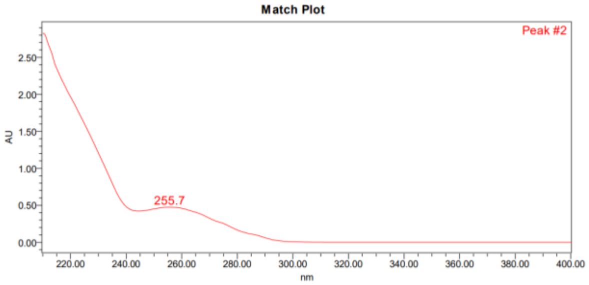

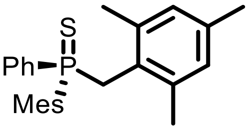

(*S*)-mesityl(phenyl)(2,4,6-trimethylbenzyl)phosphine sulfide (2’h)

67 mg, 85% yield, sticky white solid

^{1}H NMR (400 MHz, $CDCl_3$) δ 7.63 – 7.53 (m, 2H), 7.42 – 7.32 (m, 1H), 7.29 – 7.20 (m, 2H), 6.89 (d, *J* = 3.9 Hz, 2H), 6.66 (s, 2H), 4.21 – 4.13 (m, 2H), 2.38 (s, 6H), 2.30 (s, 3H), 2.18 (d, *J* = 2.7 Hz, 3H), 1.99 (s, 6H).

^{13}C NMR (101 MHz, $CDCl_3$) δ 142.03 (d, *J* = 10.0 Hz), 140.72 (d, *J* = 3.0 Hz), 138.20 (d, *J* = 5.5 Hz), 136.24 (d, *J* = 4.4 Hz), 135.56 (d, *J* = 78.1 Hz), 131.72 (d, *J* = 11.0 Hz), 131.11 (d, *J* = 9.9 Hz),

130.71 (d, J = 3.0 Hz), 129.84 (d, J = 77.1 Hz), 129.00 (d, J = 3.7 Hz), 128.72 (d, J = 81.8 Hz), 128.03 (d, J = 12.0 Hz), 127.30 (d, J = 8.2 Hz), 38.95 (d, J = 47.5 Hz), 24.92 (d, J = 4.4 Hz), 21.47 (d, J = 1.8 Hz), 20.94 (d, J = 1.5 Hz), 20.88 (d, J = 1.5 Hz).

^{31}P NMR (162 MHz, $CDCl_3$) δ 41.48.

HRMS (ESI, m/Z): calcd. for $C_{25}H_{30}PS^+$ $[M+H]^+$: 393.1800, found: 393.1795.

Melting point: cannot be measured

Specific rotation: $[\alpha]_D^{20}$= -81.29 (c = 3.885 g/100mL, $CHCl_3$).

SFC Chiralcel OD, CO_2/MeOH with gradient from 97% to 50% CO_2 in 4.5 min, 1.8 mL/min., 40 ^oC, detection at 221 nm. Retention time: 2.61 min. (major) and 2.84 min. (minor). 86% *ee*.

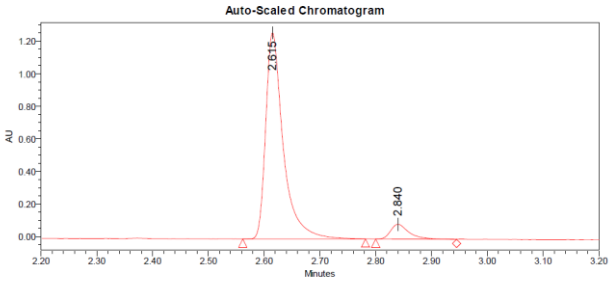

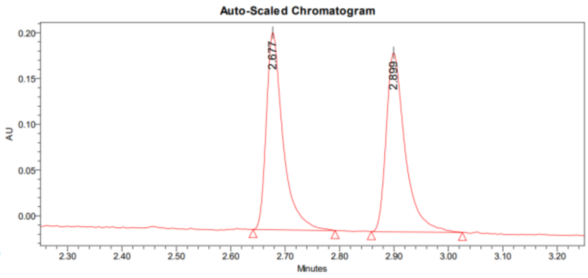

Peak Results

	Name	RT	Area	% Area	Height
1		2.615	2765227	92.97	1265558
2		2.840	209047	7.03	91303

Peak Results

	Name	RT	Area	% Area	Height
1		2.677	459239	49.33	215386
2		2.899	471645	50.67	195449

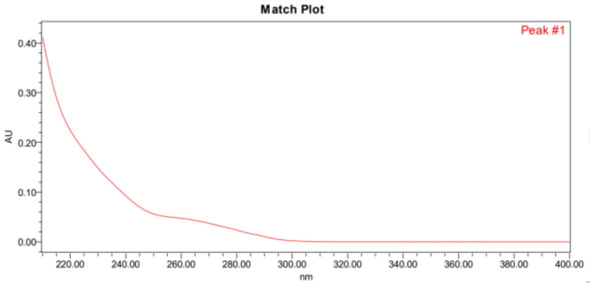

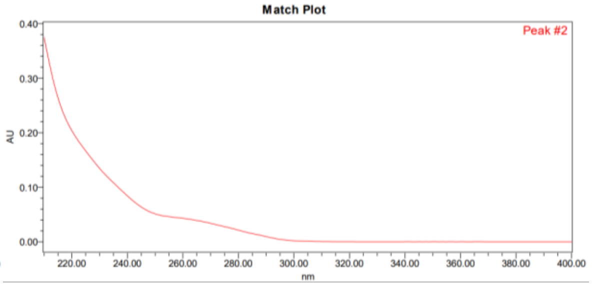

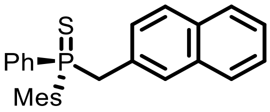

(*S*)-mesityl(naphthalen-2-ylmethyl)(phenyl)phosphine sulfide (2'i)

79.4 mg, 95% yield, white solid

^{1}H NMR (400 MHz, $CDCl_3$) δ 7.74 – 7.65 (m, 1H), 7.57 – 7.29 (m, 7H), 7.23 – 7.12 (m, 3H), 6.96 – 6.87 (m, 3H), 4.21 (dd, *J* = 15.9, 12.9 Hz, 1H), 3.88 (dd, *J* = 12.9, 10.6 Hz, 1H), 2.40 (s, 6H), 2.32 (s, 3H).

^{13}C NMR (101 MHz, $CDCl_3$) δ 141.71 (d, *J* = 10.1 Hz), 141.02 (d, *J* = 2.9 Hz), 134.00 (d, *J* = 80.0 Hz), 132.84 (d, *J* = 3.7 Hz), 132.16 (d, *J* = 2.9 Hz), 131.53 (d, *J* = 11.2 Hz), 130.91 (d, *J* = 9.9 Hz), 130.77 (d, *J* = 3.0 Hz), 129.96 (d, *J* = 7.3 Hz), 129.41 (d, *J* = 80.0 Hz), 128.89 (d, *J* = 4.2 Hz), 128.78 (d, *J* = 7.8 Hz), 128.07 (d, *J* = 12.2 Hz), 127.77 (d, *J* = 1.6 Hz), 126.70 (d, *J* = 2.9 Hz), 125.70, 125.64 (d, *J* = 1.9 Hz), 45.98 (d, *J* = 46.8 Hz), 24.53 (d, *J* = 4.8 Hz), 21.00 (d, *J* = 1.5 Hz).

^{31}P NMR (162 MHz, $CDCl_3$) δ 43.27.

HRMS (ESI, m/Z): calcd. for $C_{26}H_{26}PS^{+}$ $[M+H]^{+}$: 401.1487, found: 401.1484.

Melting point: 142.4 °C

Specific rotation: $[\alpha]_{D}^{20}$= -236.44(c = 1.001 g/100mL, CH_2Cl_2).

SFC Chiralcel OD, CO_2/MeOH with gradient from 97% to 50% CO_2 in 4.5 min, 1.8 mL/min., 40 °C, detection at 221 nm. Retention time: 3.57 min. (major) and 3.79 min. (minor). 91% *ee*.

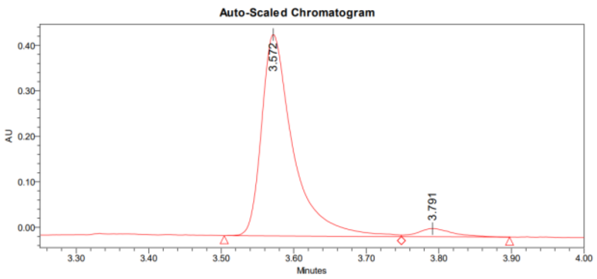

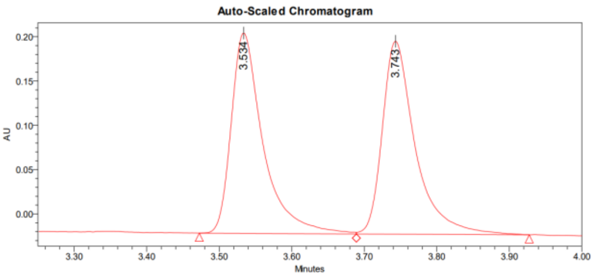

Peak Results

	Name	RT	Area	% Area	Height
1		3.572	1338279	95.64	442672
2		3.791	60971	4.36	17891

Peak Results

	Name	RT	Area	% Area	Height
1		3.534	691035	50.09	226225
2		3.743	688441	49.91	217686

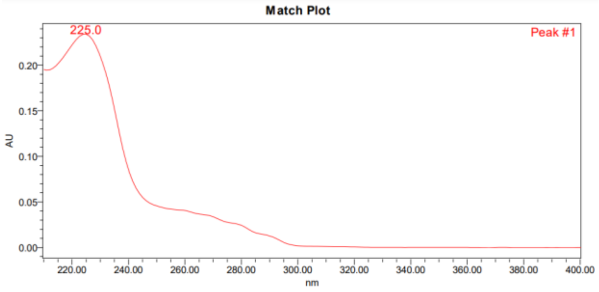

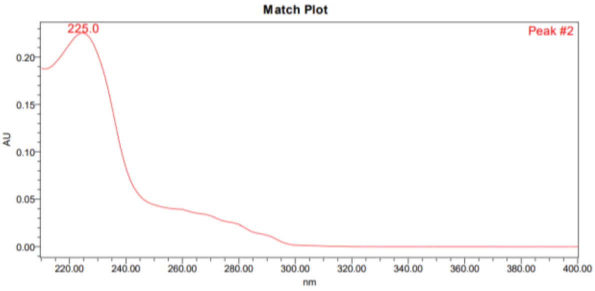

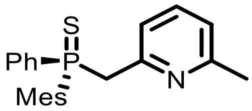

(*S*)-mesityl((6-methylpyridin-2-yl)methyl)(phenyl)phosphine sulfide (2’j)

66 mg, 90% yield, sticky white solid

^{1}H NMR (400 MHz, $CDCl_3$) δ 7.48 – 7.38 (m, 2H), 7.37 – 7.23 (m, 3H), 7.17 (td, *J* = 7.7, 2.9 Hz, 2H), 6.85 (d, *J* = 4.1 Hz, 2H), 6.75 (dt, *J* = 6.9, 2.2 Hz, 1H), 4.18 – 4.02 (m, 2H), 2.33 (s, 6H), 2.27 (s, 3H), 2.05 (s, 3H).

^{13}C NMR (101 MHz, $CDCl_3$) δ 157.23 (d, *J* = 2.6 Hz), 151.44 (d, *J* = 6.8 Hz), 141.60 (d, *J* = 10.2 Hz), 140.98 (d, *J* = 2.9 Hz), 135.70 (d, *J* = 3.1 Hz), 133.38 (d, *J* = 80.7 Hz), 131.36 (d, *J* = 11.1 Hz), 130.83 (d, *J* = 10.2 Hz), 130.44 (d, *J* = 3.1 Hz), 129.15 (d, *J* = 82.2 Hz), 127.69 (d, *J* = 12.4 Hz), 122.37 (d, *J* = 4.1 Hz), 120.99 (d, *J* = 3.5 Hz), 48.83 (d, *J* = 46.3 Hz), 24.28 (d, *J* = 5.1 Hz), 23.82, 20.97 (d, *J* = 1.5 Hz).

^{31}P NMR (162 MHz, $CDCl_3$) δ 42.41.

HRMS (ESI, m/Z): calcd. for $C_{22}H_{25}NPS^{+}$ $[M+H]^{+}$: 366.1440, found: 366.1429.

Melting point: cannot be measured

Specific rotation: $[\alpha]_{\mathrm{D}}^{20}$= -62.3 (c = 4.490 g/100mL, $CHCl_3$).

SFC Chiralcel OD, CO_2/MeOH with gradient from 97% to 50% CO_2 in 4.5 min, 1.8 mL/min., 40 ^{o}C, detection at 221 nm. Retention time: 2.17 min. (major) and 2.27 min. (minor). 87% *ee*.

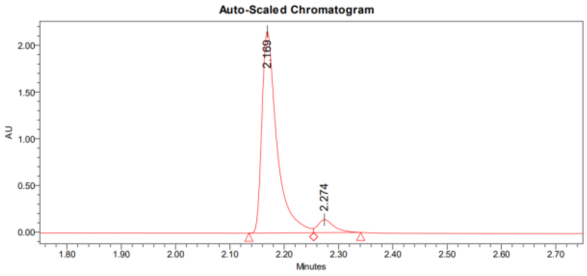

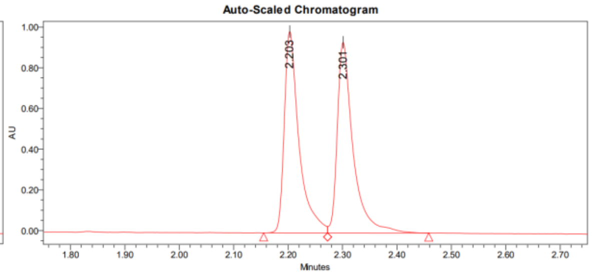

Peak Results

	Name	RT	Area	% Area	Height
1		2.169	4142282	93.46	2159352
2		2.274	289880	6.54	139466

Peak Results

	Name	RT	Area	% Area	Height
1		2.203	1861667	49.63	989669
2		2.301	1889544	50.37	936756

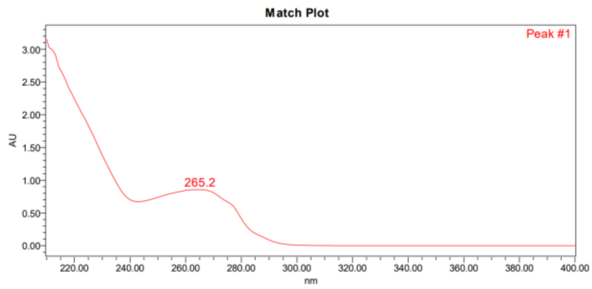

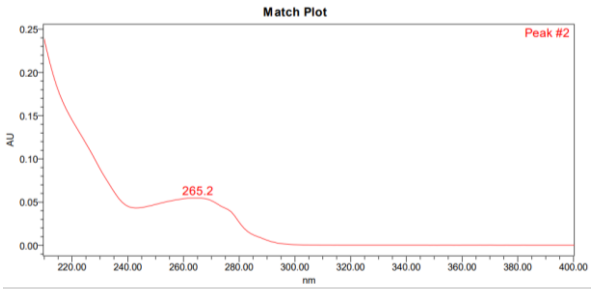

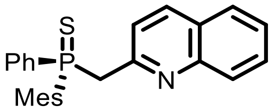

(*S*)-mesityl(phenyl)(quinolin-2-ylmethyl)phosphine sulfide (2’k)

39.4 mg, 98% yield, white solid

^{1}H NMR (400 MHz, $CDCl_3$) δ 7.91 (d, *J* = 8.4 Hz, 1H), 7.67 (dd, *J* = 7.8, 1.3 Hz, 1H), 7.62 – 7.35 (m, 6H), 7.20 – 7.02 (m, 3H), 6.88 (d, *J* = 4.2 Hz, 2H), 4.45 – 4.26 (m, 2H), 2.38 (s, 6H), 2.29 (s, 3H).

^{13}C NMR (101 MHz, $CDCl_3$) δ 153.38 (d, *J* = 6.6 Hz), 147.54 (d, *J* = 2.4 Hz), 141.68 (d, *J* = 10.4 Hz), 141.14 (d, *J* = 2.9 Hz), 135.24 (d, *J* = 2.3 Hz), 133.19 (d, *J* = 80.7 Hz), 131.48 (d, *J* = 11.4 Hz), 130.86 (d, *J* = 10.2 Hz), 130.68 (d, *J* = 3.1 Hz), 129.20 (d, *J* = 82.6 Hz), 129.08, 128.67, 127.87 (d, *J* = 12.5 Hz), 127.49 (d, *J* = 1.6 Hz), 126.68 (d, *J* = 2.3 Hz), 126.10 (d, *J* = 1.5 Hz), 123.34 (d, *J* = 2.7 Hz), 49.67 (d, *J* = 46.1 Hz), 24.35 (d, *J* = 4.9 Hz), 21.02 (d, *J* = 1.5 Hz).

^{31}P NMR (162 MHz, $CDCl_3$) δ 41.66.

HRMS (ESI, m/Z): calcd. for $C_{25}H_{25}NPS^+$ $[M+H]^+$: 402.1440, found: 402.1429.

Melting point: 64.6 ^{o}C

Specific rotation: $[\alpha]_D^{20}$= +4.20 (c = 2.811 g/100mL, $CHCl_3$).

SFC Chiralcel OD, CO_2/MeOH with gradient from 97% to 50% CO_2 in 4.5 min, 1.8 mL/min., 40 ^{o}C, detection at 221 nm. Retention time: 2.80 min. (major) and 2.99 min. (minor). 82% *ee*.

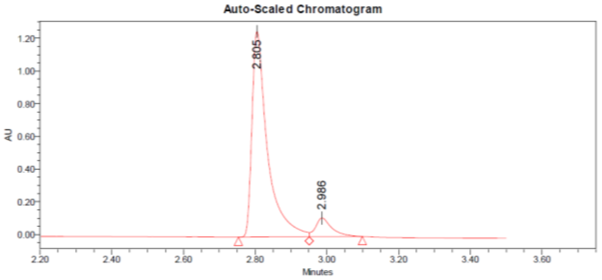

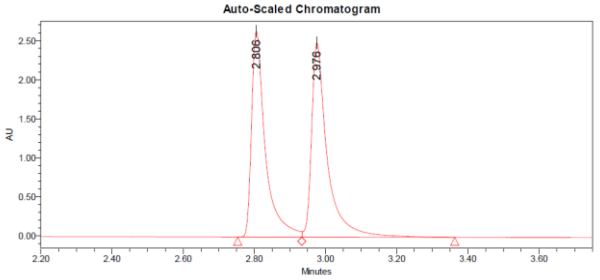

Peak Results

	Name	RT	Area	% Area	Height
1		2.805	3765302	91.19	1257991
2		2.986	363855	8.81	115124

Peak Results

	Name	RT	Area	% Area	Height
1		2.806	7111290	48.25	2636145
2		2.976	7628557	51.75	2489731

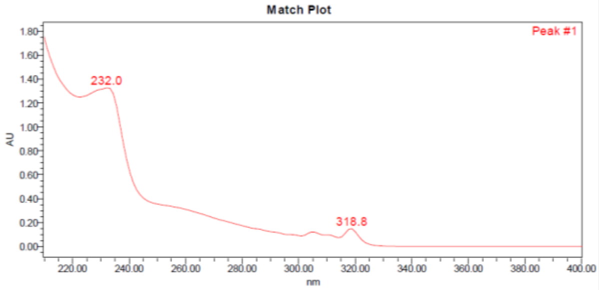

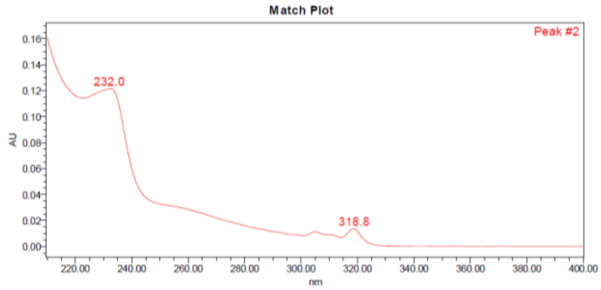

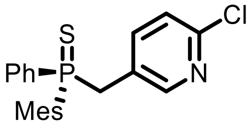

(S)-((6-chloropyridin-3-yl)methyl)(mesityl)(phenyl)phosphine sulfide (2'l)

33.8 mg, 88% yield, white solid

^{1}H NMR (400 MHz, $CDCl_3$) δ 7.46 – 7.39 (m, 4H), 7.37 – 7.27 (m, 3H), 7.04 (d, J = 8.2 Hz, 1H), 6.89 (d, J = 4.1 Hz, 2H), 3.95 (dd, J = 15.5, 13.0 Hz, 1H), 3.66 (dd, J = 13.0, 10.5 Hz, 1H), 2.35 (s, 6H), 2.30 (s, 3H).

^{13}C NMR (101 MHz, $CDCl_3$) δ 150.66 (d, J = 5.9 Hz), 149.99 (d, J = 4.4 Hz), 141.68 (d, J = 10.3 Hz), 141.49 (d, J = 2.9 Hz), 140.75 (d, J = 4.4 Hz), 133.27 (d, J = 80.3 Hz), 131.64 (d, J = 11.3 Hz), 131.55 (d, J = 3.0 Hz), 130.67 (d, J = 9.9 Hz), 128.55 (d, J = 12.4 Hz), 128.45 (d, J = 82.1 Hz), 126.53 (d, J = 7.1 Hz), 122.98 (d, J = 3.2 Hz), 42.34 (d, J = 47.2 Hz), 24.44 (d, J = 5.0 Hz), 21.02 (d, J = 1.6 Hz).

^{31}P NMR (162 MHz, $CDCl_3$) δ 42.73.

HRMS (ESI, m/Z): calcd. for $C_{21}H_{22}ClNPS^{+}$ $[M+H]^{+}$: 386.0894, found: 386.0890.

Melting point: 65.6 °C

Specific rotation: $[\alpha]_{\mathbf{D}}^{\mathbf{20}}$= -233.17 (c = 1.043 g/100mL, $CHCl_3$).

SFC Chiralcel OD, CO_2/MeOH with gradient from 97% to 50% CO_2 in 4.5 min, 1.8 mL/min., 40 °C, detection at 221 nm. Retention time: 2.66 min. (minor) and 2.92 min. (major). 91% *ee*.

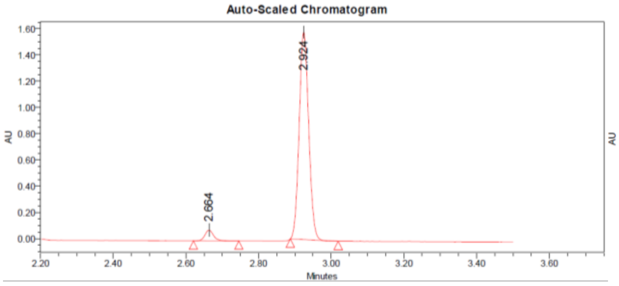

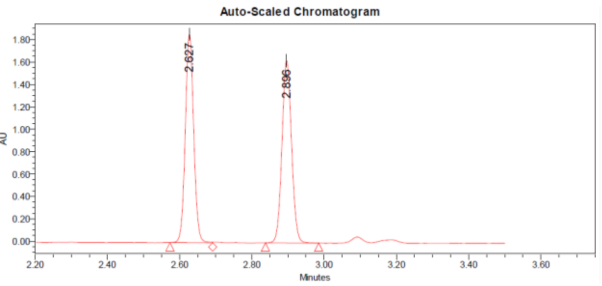

Peak Results

	Name	RT	Area	% Area	Height
1		2.664	137786	4.59	81768
2		2.924	2865516	95.41	1581594

Peak Results

	Name	RT	Area	% Area	Height
1		2.627	3003905	50.06	1863032
2		2.896	2996399	49.94	1628696

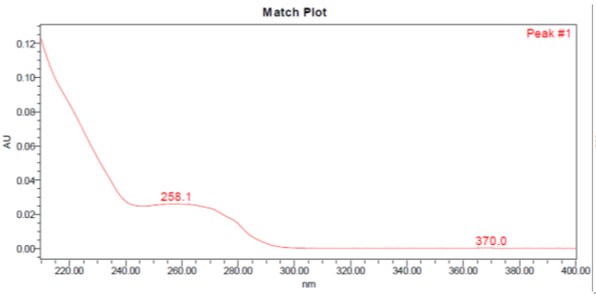

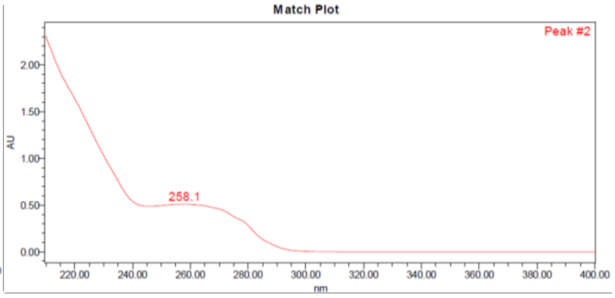

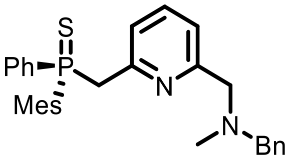

(*S*)-((6-((benzyl(methyl)amino)methyl)pyridin-2-yl)methyl)(mesityl)(phenyl)phosphine sulfide (2'm)

42.4 mg, 87% yield, sticky light yellow solid

^{1}H NMR (400 MHz, $CDCl_3$) δ 7.51 – 7.36 (m, 4H), 7.34 – 7.18 (m, 6H), 7.17-7.05 (m, 3H), 6.85 (d, J = 4.1 Hz, 2H), 4.23 – 4.07 (m, C(1)$_{SP}$^{3}-H, 2H), 3.58 – 2.97 (m, C(2 and 3)$_{SP}$^{3}-H, 4H), 2.33 (s, C(5 and 6)$_{SP}$^{3}-H, 6H), 2.28 (s, C(7)$_{SP}$^{3}-H, 3H), 2.05 (s, C(4)$_{SP}$^{3}-H, 3H).

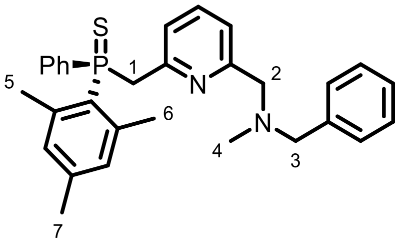

^{13}**C NMR** (101 MHz, $CDCl_3$) δ 151.56 (d, *J* = 7.1 Hz), 141.66 (d, *J* = 10.2 Hz), 141.03 (d, *J* = 2.8 Hz), 136.01 (d, *J* = 3.1 Hz), 133.43 (d, *J* = 80.6 Hz), 131.42 (d, *J* = 11.3 Hz), 130.95 (d, *J* = 10.1 Hz), 130.60 (d, *J* = 3.0 Hz), 129.26 (d, *J* = 82.8 Hz), 129.10, 128.36, 127.79 (d, *J* = 12.4 Hz), 127.23, 123.68, 120.68, 61.99, 48.64 (d, *J* = 46.7 Hz), 42.50, 30.44, 24.30 (d, *J* = 5.1 Hz), 21.00 (d, *J* = 1.5 Hz).

^{31}**P NMR** (162 MHz, $CDCl_3$) δ 42.15.

HRMS (ESI, m/Z): calcd. for $C_{30}H_{34}N_2PS^+$ [M+H]$^+$: 485.2175, found: 485.2168.

Melting point: cannot be measured, as it decomposes during heating

Specific rotation: $[\alpha]_D^{20}$= -38.63 (c = 2.511 g/100mL, $CHCl_3$).

HPLC CHIRALPAK OD-H, heptane/*i*-PrOH = 95/5, flow rate: 0.5 mL/min, 40 °C, detection at 221 nm. Retention time: 12.04 min. (major) and 16.39 min. (minor). 89% *ee*.

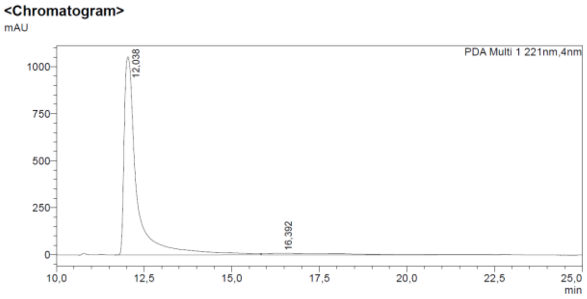

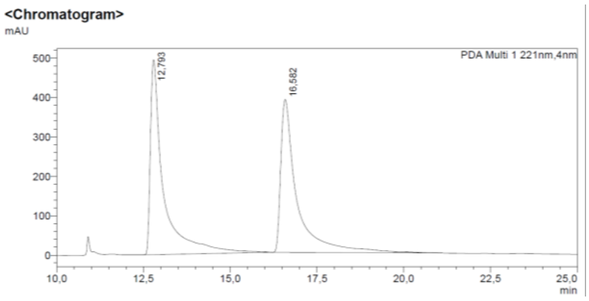

<Peak Table>

PDA Ch1 221nm

Peak#	Ret. Time	Area	Height	Area%
1	12,038	26553384	1054014	94,015
2	16,392	1690272	9412	5,985
Total		28243656	1063427	100,000

<Peak Table>

PDA Ch1 221nm

Peak#	Ret. Time	Area	Height	Area%
1	12,793	13105805	494342	50,786
2	16,582	12700238	387554	49,214
Total		25806042	881896	100,000

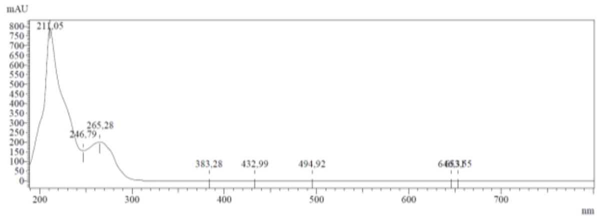

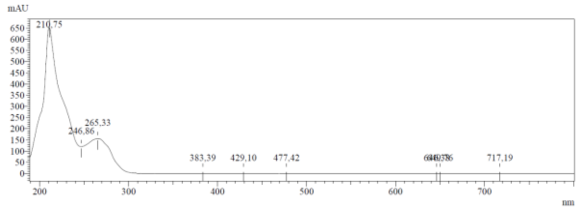

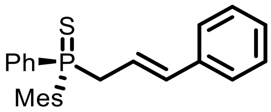

(*S*)-cinnamyl(mesityl)(phenyl)phosphine sulfide (2’n)

24.2 mg, 64% yield, sticky white solid

^{1}H NMR (400 MHz, $CDCl_3$) δ 7.78 – 7.67 (m, 2H), 7.50 – 7.34 (m, 3H), 7.28 – 7.12 (m, 3H), 7.10 – 7.03 (m, 2H), 6.87 (d, *J* = 4.0 Hz, 2H), 6.04 (dd, *J* = 15.8, 5.7 Hz, 1H), 5.76 (dtd, *J* = 15.7, 7.4, 6.2 Hz, 1H), 3.57 – 3.38 (m, 2H), 2.35 (s, 6H), 2.29 (s, 3H).

^{13}C NMR (101 MHz, $CDCl_3$) δ 141.70 (d, *J* = 10.2 Hz), 141.13 (d, *J* = 2.9 Hz), 137.02 (d, *J* = 3.9 Hz), 135.84 (d, *J* = 13.9 Hz), 134.62 (d, *J* = 79.9 Hz), 131.45 (d, *J* = 11.0 Hz), 130.98 (d, *J* = 3.1 Hz), 130.89 (d, *J* = 9.8 Hz), 128.88 (d, *J* = 79.8 Hz), 128.49(d, *J* = 1.1 Hz), 128.33 (d, *J* = 12.2 Hz), 127.55 (d, *J* = 1.4 Hz), 126.30 (d, *J* = 2.3 Hz), 119.48 (d, *J* = 9.8 Hz), 44.64 (d, *J* = 50.1 Hz), 24.20 (d, *J* = 5.1 Hz), 21.01 (d, *J* = 1.7 Hz).

^{31}P NMR (162 MHz, $CDCl_3$) δ 41.81.

HRMS (ESI, m/Z): calcd. for $C_{24}H_{26}PS^{+}$ $[M+H]^{+}$: 377.1487, found: 377.1486.

Melting point: cannot be measured

Specific rotation: $[\alpha]_{D}^{20}$= -99.28 (c = 1.386 g/100mL, $CHCl_3$).

SFC Chiralcel OJ-3, CO_2/MeOH with gradient from 97% to 50% CO_2 in 4.5 min, 1.8 mL/min., 40 °C, detection at 215 nm. Retention time: 2.69 min. (minor) and 3.08 min. (major). 85% *ee*.

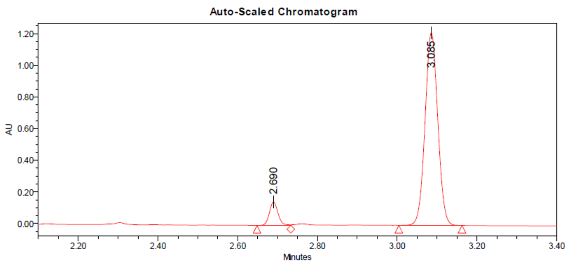

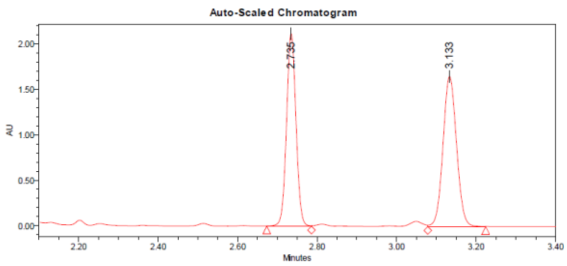

Peak Results

	Name	RT	Area	% Area	Height
1		2.690	222794	7.63	147763
2		3.085	2695505	92.37	1216932

Peak Results

	Name	RT	Area	% Area	Height
1		2.735	3492778	47.29	2116251
2		3.133	3893179	52.71	1649618

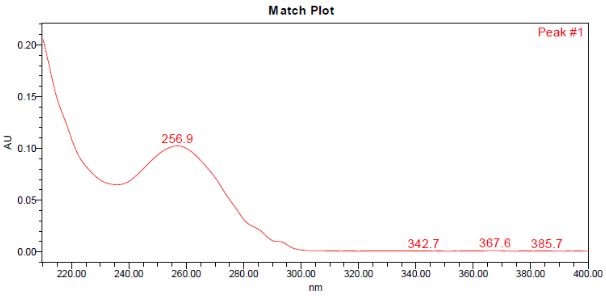

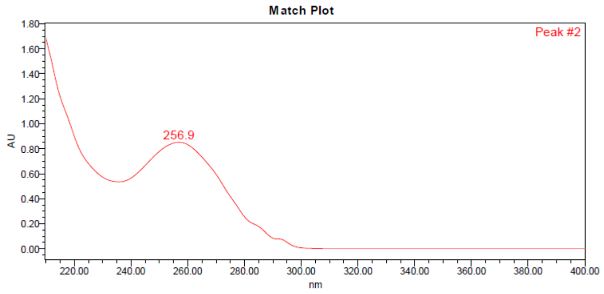

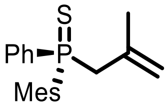

(*S*)-mesityl(2-methylallyl)(phenyl)phosphine sulfide (2’o)

29 mg, 92% yield, white solid

^{1}H NMR (400 MHz, $CDCl_3$) δ 7.80 – 7.70 (m, 2H), 7.49 – 7.35 (m, 3H), 6.84 (d, *J* = 4.0 Hz, 2H), 4.65 (d, *J* = 5.4 Hz, 1H), 4.37 (d, *J* = 5.6 Hz, 1H), 3.51 (dd, *J* = 17.5, 13.1 Hz, 1H), 3.24 (dd, *J* = 13.1, 11.5 Hz, 1H), 2.30 (s, 6H), 2.27 (s, 3H), 1.53 (d, *J* = 3.2 Hz, 3H).

^{13}C NMR (101 MHz, $CDCl_3$) δ 141.48 (d, *J* = 10.0 Hz), 140.79 (d, *J* = 2.9 Hz), 136.63 (d, *J* = 9.5 Hz), 134.36 (d, *J* = 79.2 Hz), 131.43 (d, *J* = 10.1 Hz), 131.42, 130.92 (d, *J* = 3.1 Hz), 130.00 (d, *J* = 80.6 Hz), 128.22 (d, *J* = 12.4 Hz), 117.30 (d, *J* = 11.0 Hz), 47.11 (d, *J* = 48.8 Hz), 24.75 (d, *J* = 2.1 Hz), 24.37 (d, *J* = 4.8 Hz), 20.95 (d, *J* = 1.7 Hz).

^{31}P NMR (162 MHz, $CDCl_3$) δ 40.90.

HRMS (ESI, m/Z): calcd. for $C_{19}H_{24}PS^+$ $[M+H]^+$: 315.1331, found: 315.1327.

Melting point: 95.2 ^{o}C

Specific rotation: $[\alpha]_{\mathbf{D}}^{\mathbf{20}}$= -84.54 (c = 2.569 g/100mL, $CHCl_3$).

SFC Chiralcel OD, CO_2/MeOH with gradient from 97% to 50% CO_2 in 4.5 min, 1.8 mL/min., 40 ^{o}C, detection at 221 nm. Retention time: 1.89 min. (major) and 2.03 min. (minor). 84% *ee*.

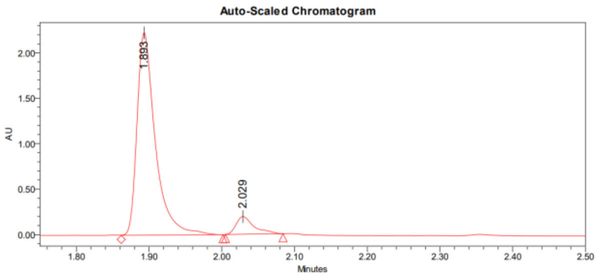

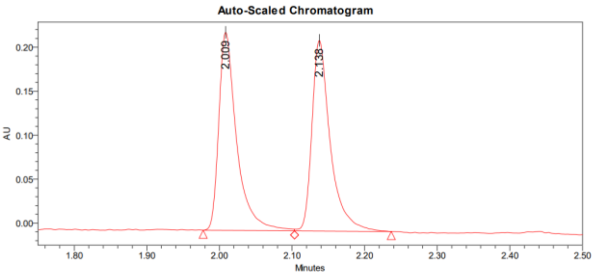

	Peak Results				
	Name	RT	Area	% Area	Height
1		1.893	3878882	92.12	2228245
2		2.029	331854	7.88	194379

	Peak Results				
	Name	RT	Area	% Area	Height
1		2.009	386163	50.29	225374
2		2.138	381716	49.71	216854

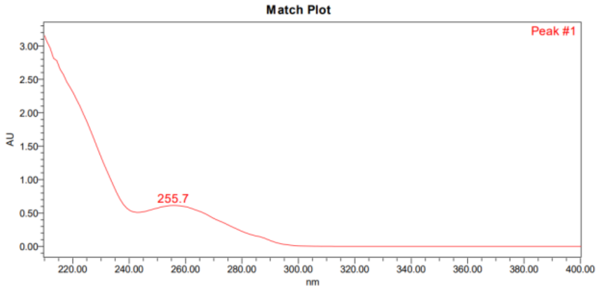

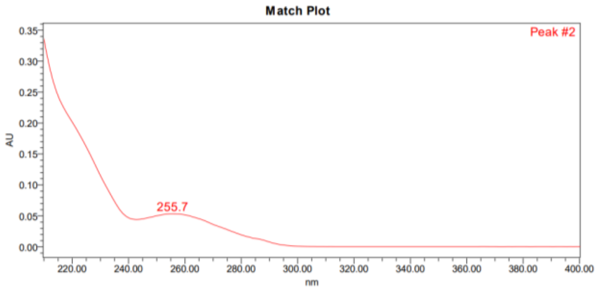

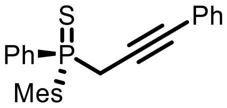

(*S*)-mesityl(phenyl)(3-phenylprop-2-yn-1-yl)phosphine sulfide (2'p)

31 mg, 82% yield, white solid

^{1}H NMR (400 MHz, $CDCl_3$) δ 8.06 – 7.95 (m, 2H), 7.59 – 7.40 (m, 3H), 7.33 – 7.12 (m, 5H), 6.88 (d, *J* = 4.3 Hz, 2H), 3.86 (dd, *J* = 17.3, 14.6 Hz, 1H), 3.29 (dd, *J* = 17.4, 15.6 Hz, 1H), 2.39 (s, 6H), 2.28 (s, 3H).

^{13}C NMR (101 MHz, $CDCl_3$) δ 142.07 (d, *J* = 10.2 Hz), 141.37 (d, *J* = 2.9 Hz), 134.86 (d, *J* = 80.6 Hz), 131.56 (d, *J* = 3.0 Hz), 131.51, 131.28 (d, *J* = 11.4 Hz), 130.85 (d, *J* = 9.9 Hz), 128.41, 128.37, 128.37 (d, *J* = 12.4 Hz), 126.78 (d, *J* = 84.8 Hz), 122.86 (d, *J* = 3.9 Hz), 84.86 (d, *J* = 8.8 Hz), 82.15 (d, *J* = 13.2 Hz), 35.35 (d, *J* = 52.1 Hz), 23.37 (d, *J* = 5.5 Hz), 21.05 (d, *J* = 1.5 Hz).

^{31}P NMR (162 MHz, $CDCl_3$) δ 39.02.

HRMS (ESI, m/Z): calcd. for $C_{24}H_{24}PS^+$ $[M+H]^+$: 375.1331, found: 375.1326.

Melting point: 53.2 ^{o}C

Specific rotation: $[\alpha]_{\mathrm{D}}^{20}$= +67.56 (c = 2.448 g/100mL, $CHCl_3$).

SFC Chiralcel OD, CO_2/MeOH with gradient from 97% to 50% CO_2 in 4.5 min, 1.8 mL/min., 40 ^{o}C, detection at 221 nm. Retention time: 2.58 min. (minor) and 2.70 min. (major). 90% *ee*.

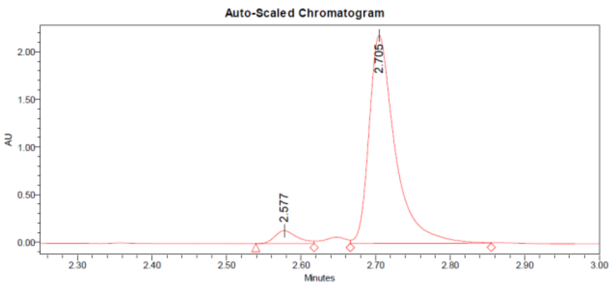

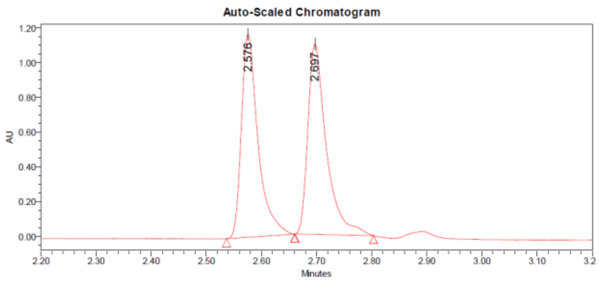

Peak Results

	Name	RT	Area	% Area	Height
1		2.577	277356	4.96	134265
2		2.705	5309047	95.04	2182419

Peak Results

	Name	RT	Area	% Area	Height
1		2.576	2440651	48.88	1167685
2		2.697	2552677	51.12	1097048

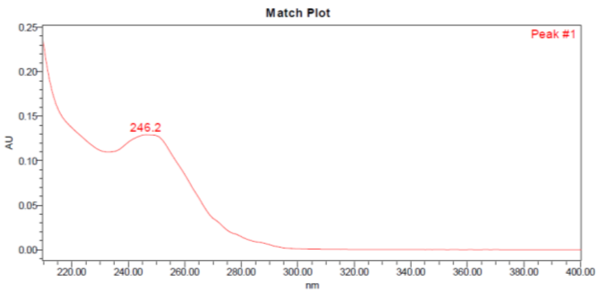

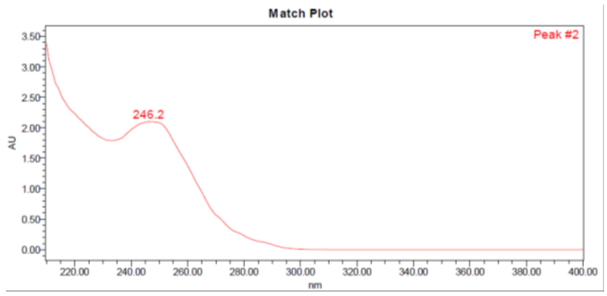

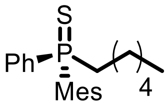

(S)-hexyl(mesityl)(phenyl)phosphine sulfide (3’a)

32.4 mg, 94% yield, sticky white solid

^{1}**H NMR** (400 MHz, Chloroform-*d*) δ 7.86 – 7.66 (m, 2H), 7.53 – 7.32 (m, 3H), 6.82 (d, *J* = 3.9 Hz, 2H), 2.69 – 2.34 (m, 2H), 2.29 (s, 6H), 2.25 (s, 3H), 1.82 – 1.57 (m, 1H), 1.38 – 1.01 (m, 6H), 0.89 – 0.63 (m, 4H).

^{13}**C NMR** (101 MHz, $CDCl_3$) δ 141.65 (d, *J* = 10.0 Hz), 140.84, 134.91 (d, *J* = 77.3 Hz), 131.40 (d, *J* = 11.0 Hz), 130.81 (d, *J* = 2.9 Hz), 130.79 (d, *J* = 10.1 Hz), 129.14 (d, *J* = 81.6 Hz), 128.43 (d, *J* = 11.9 Hz), 38.88 (d, *J* = 53.0 Hz), 31.36 (d, *J* = 1.0 Hz), 30.56 (d, *J* = 17.2 Hz), 24.11 (d, *J* = 5.1 Hz), 22.94 (d, *J* = 3.3 Hz), 22.47, 20.93 (d, *J* = 1.7 Hz), 14.03.

^{31}**P NMR** (162 MHz, Chloroform-*d*) δ 43.66.

HRMS (ESI, m/Z): calcd. for $C_{21}H_{30}PS^+$ $[M+H]^+$: 345.1800, found: 345.1796.

Melting point: cannot be measured

Specific rotation: $[\alpha]_D^{20}$= -72.84 (c = 2.850 g/100mL, $CHCl_3$).

SFC Chiralcel OD, CO_2/MeOH with gradient from 97% to 50% CO_2 in 4.5 min, 1.8 mL/min., 40 °C, detection at 221 nm. Retention time: 1.88 min. (major) and 1.99 min. (minor). 90% *ee*.

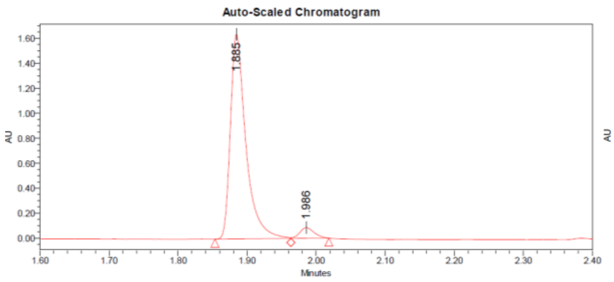

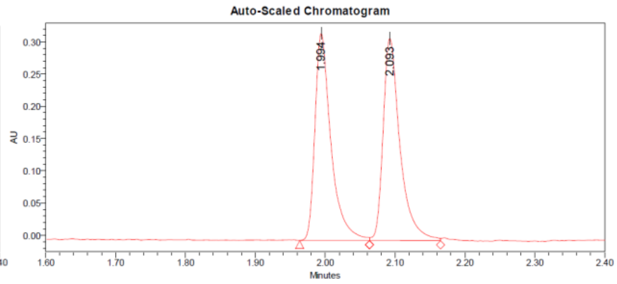

Peak Results

	Name	RT	Area	% Area	Height
1		1.885	2544153	95.38	1640247
2		1.986	123165	4.62	85464

Peak Results

	Name	RT	Area	% Area	Height
1		1.994	513756	49.49	321097
2		2.093	524354	50.51	313456

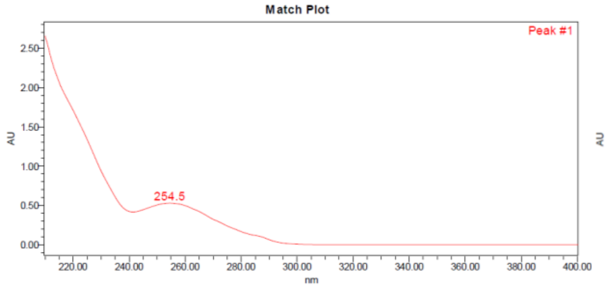

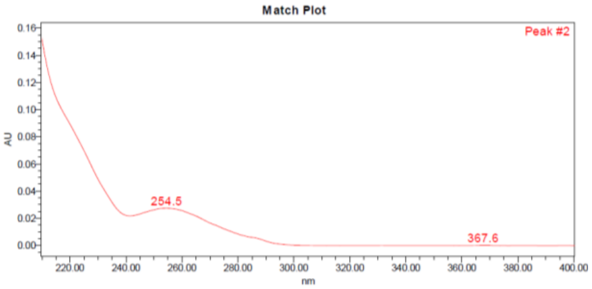

3'a from 1-iodohexane

30.0 mg, 85% yield

SFC Chiralcel OD, CO_2/MeOH with gradient from 97% to 50% CO_2 in 4.5 min, 1.8 mL/min., 40 °C, detection at 221 nm. Retention time: 1.91 min. (major) and 2.01 min. (minor). 92% *ee*.

Peak Results

	Name	RT	Area	% Area	Height
1		1.912	2579915	96.13	1593276
2		2.016	103872	3.87	61557

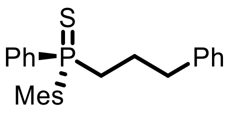

(*S*)-mesityl(phenyl)(3-phenylpropyl)phosphine sulfide (3'b)

36.2 mg, 96% yield, white solid

^{1}H NMR (400 MHz, $CDCl_3$) δ 7.77 – 7.68 (m, 2H), 7.51 – 7.44 (m, 1H), 7.44 – 7.37 (m, 2H), 7.25 – 7.18 (m, 2H), 7.18 – 7.11 (m, 1H), 7.03 – 6.94 (m, 2H), 6.81 (d, *J* = 3.9 Hz, 2H), 2.73 – 2.48 (m, 3H), 2.47 – 2.33 (m, 1H), 2.25 (s, 9H), 2.12 – 1.96 (m, 1H), 1.19 – 1.03 (m, 1H).

^{13}C NMR (101 MHz, $CDCl_3$) δ 141.66 (d, *J* = 10.2 Hz), 140.98, 140.91 (d, *J* = 2.9 Hz), 134.77 (d, *J* = 77.4 Hz), 131.43 (d, *J* = 11.2 Hz), 130.92 (d, *J* = 3.1 Hz), 130.86 (d, *J* = 10.1 Hz), 129.06 (d, *J* = 81.9 Hz), 128.60, 128.49, 128.48, 126.13, 38.06 (d, *J* = 53.6 Hz), 36.63 (d, *J* = 17.4 Hz), 24.47 (d, *J* = 2.5 Hz), 24.10 (d, *J* = 5.1 Hz), 20.95 (d, *J* = 1.5 Hz).

^{31}P NMR (162 MHz, $CDCl_3$) δ 43.44.

HRMS (ESI, m/Z): calcd. for $C_{24}H_{28}PS^+$ $[M+H]^+$: 379.1644, found: 379.1639.

Melting point: 124.0 °C

Specific rotation: $[\alpha]_D^{20}$= -67.47 (c = 3.086 g/100mL, $CHCl_3$).

SFC Chiralcel OJ-3, CO_2/MeOH with gradient from 97% to 50% CO_2 in 4.5 min, 1.8 mL/min., 40 °C, detection at 221 nm. Retention time: 2.55 min. (minor) and 2.87 min. (major). 89% *ee*.

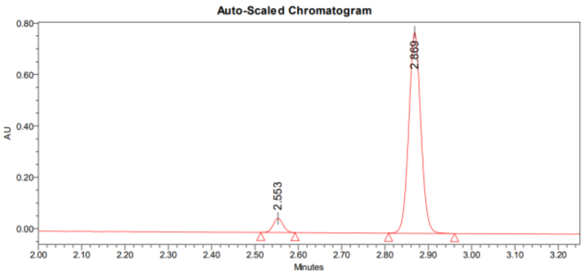

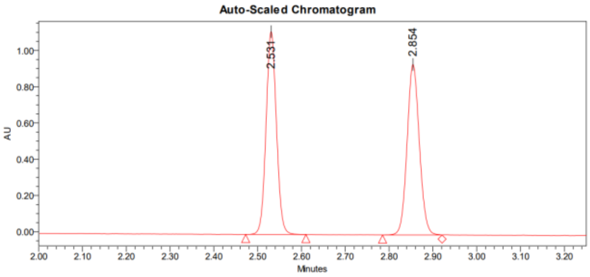

Peak Results

	Name	RT	Area	% Area	Height
1		2.553	87563	5.46	55165
2		2.869	1516122	94.54	782639

Peak Results

	Name	RT	Area	% Area	Height
1		2.531	1833968	50.01	1118776
2		2.854	1833077	49.99	940675

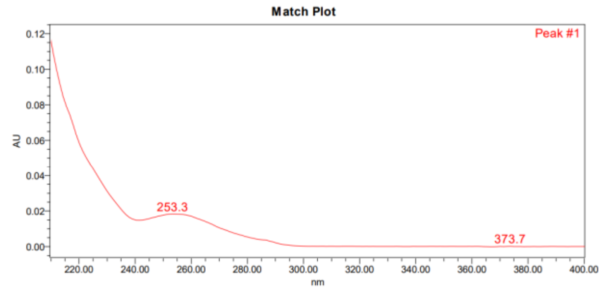

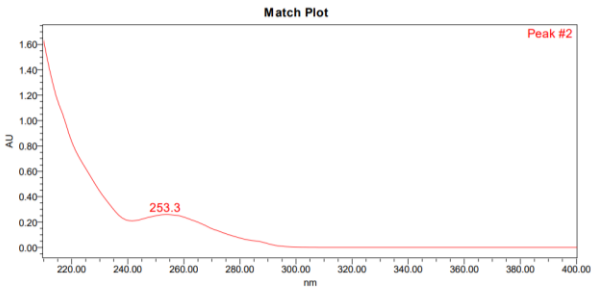

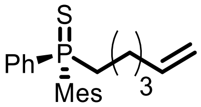

(*S*)-hex-5-en-1-yl(mesityl)(phenyl)phosphine sulfide (3’c)

33.5 mg, 98% yield, white solid

^{1}H NMR (400 MHz, $CDCl_3$) δ 7.81 – 7.70 (m, 2H), 7.52 – 7.37 (m, 3H), 6.83 (d, *J* = 4.0 Hz, 2H), 5.65 (ddt, *J* = 16.9, 10.2, 6.6 Hz, 1H), 4.93 – 4.81 (m, 2H), 2.63 – 2.36 (m, 2H), 2.29 (s, 6H), 2.26 (s, 3H), 1.92 (q, *J* = 7.0 Hz, 2H), 1.78 – 1.57 (m, 1H), 1.49 – 1.23 (m, 2H), 0.89 – 0.71 (m, 1H).

^{13}C NMR (101 MHz, $CDCl_3$) δ 141.55 (d, *J* = 10.2 Hz), 140.78 (d, *J* = 2.9 Hz), 138.10, 134.75 (d, *J* = 77.3 Hz), 131.32 (d, *J* = 11.1 Hz), 130.75 (d, *J* = 2.8 Hz), 130.69 (d, *J* = 10.1 Hz), 128.99 (d, *J* = 81.8 Hz), 128.36 (d, *J* = 12.1 Hz), 114.65, 38.59 (d, *J* = 53.2 Hz), 33.17, 29.97 (d, *J* = 17.3 Hz), 24.01 (d, *J* = 5.1 Hz), 22.44 (d, *J* = 3.2 Hz), 20.83 (d, *J* = 1.6 Hz).

^{31}P NMR (162 MHz, $CDCl_3$) δ 43.56.

HRMS (ESI, m/Z): calcd. for $C_{21}H_{28}PS^+$ $[M+H]^+$: 343.1644, found: 343.1642.

Melting point: 66.2 ^{o}C

Specific rotation: $[\alpha]_D^{20}$= -73.87 (c = 2.231 g/100mL, $CHCl_3$).

SFC Chiralcel OD, CO_2/MeOH with gradient from 97% to 50% CO_2 in 4.5 min, 1.8 mL/min., 40 ^{o}C, detection at 221 nm. Retention time: 1.86 min. (major) and 1.96 min. (minor). 89% *ee*.

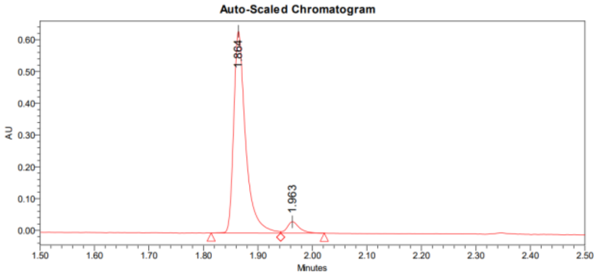

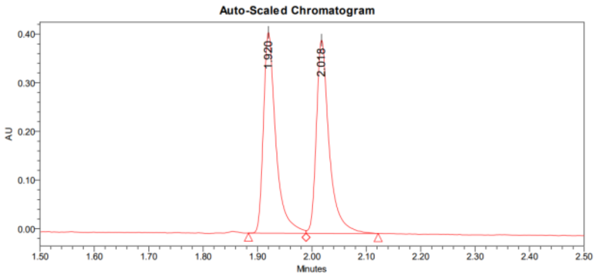

	Name	RT	Area	% Area	Height
1		1.864	964461	94.57	635101
2		1.963	55390	5.43	35699

Peak Results

	Name	RT	Area	% Area	Height
1		1.920	653723	50.14	412582
2		2.018	650176	49.86	397359

Peak Results

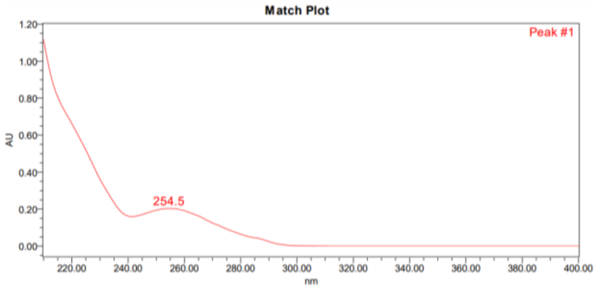

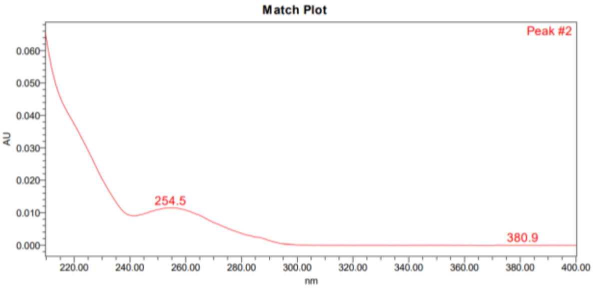

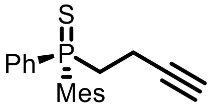

(*S*)-but-3-yn-1-yl(mesityl)(phenyl)phosphine sulfide (3'd)

26.3 mg, 84% yield, white solid

^{1}H NMR (400 MHz, $CDCl_3$) δ 7.80 – 7.70 (m, 2H), 7.54 – 7.39 (m, 3H), 6.84 (d, *J* = 4.1 Hz, 2H), 2.97 – 2.83 (m, 1H), 2.74 – 2.57 (m, 2H), 2.30 (s, 6H), 2.27 (s, 3H), 1.90 (t, *J* = 2.5 Hz, 1H), 1.67 – 1.54 (m, 1H).

^{13}C NMR (101 MHz, $CDCl_3$) δ 141.73 (d, *J* = 10.3 Hz), 141.31 (d, *J* = 2.9 Hz), 133.96 (d, *J* = 77.3 Hz), 131.55 (d, *J* = 11.3 Hz), 131.30 (d, *J* = 3.0 Hz), 130.84 (d, *J* = 10.3 Hz), 128.79 (d, *J* = 12.1 Hz), 128.36 (d, *J* = 83.8 Hz), 83.15 (d, *J* = 24.0 Hz), 69.19 (d, *J* = 2.2 Hz), 37.56 (d, *J* = 52.6 Hz), 24.16 (d, *J* = 5.4 Hz), 20.99 (d, *J* = 1.5 Hz), 12.99 (d, *J* = 1.3 Hz).

^{31}P NMR (162 MHz, $CDCl_3$) δ 41.87.

HRMS (ESI, m/Z): calcd. for $C_{19}H_{22}PS^{+}$ $[M+H]^{+}$: 313.1174, found: 313.1171.

Melting point: 107.6 °C

Specific rotation: $[\alpha]_{D}^{20}$= -38.25 (c = 1.621 g/100mL, $CHCl_3$).

SFC Chiralcel OJ-3, CO_2/MeOH with gradient from 97% to 50% CO_2 in 4.5 min, 1.8 mL/min., 40 °C, detection at 221 nm. Retention time: 1.72 min. (minor) and 1.80 min. (major). 90% *ee*.

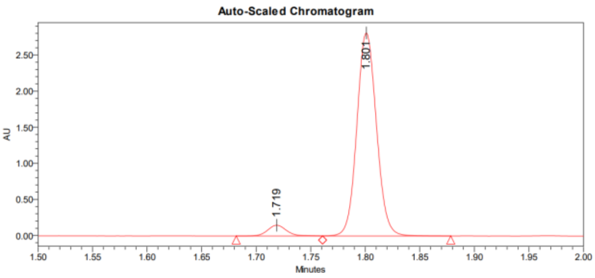

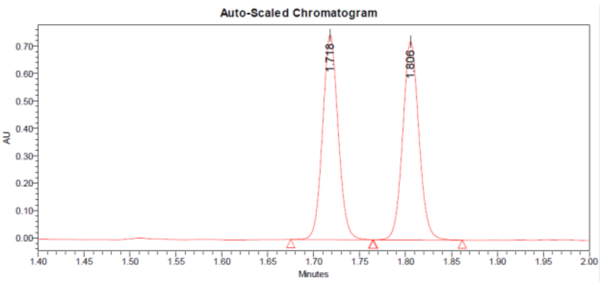

Peak Results

	Name	RT	Area	% Area	Height
1		1.719	180919	4.90	149130
2		1.801	3511566	95.10	2813624

Peak Results

	Name	RT	Area	% Area	Height
1		1.718	880731	49.82	747108
2		1.806	887210	50.18	724386

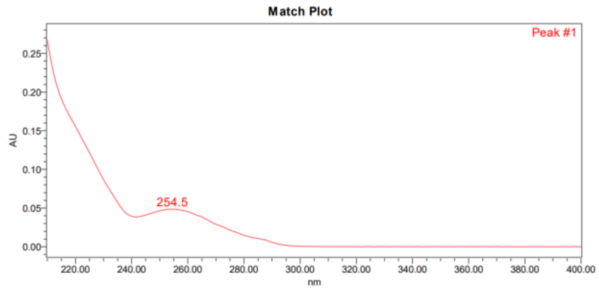

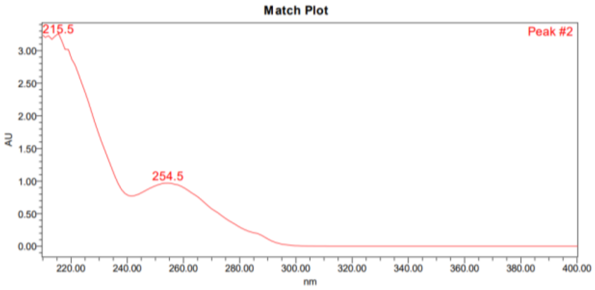

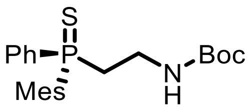

tert-butyl (*S*)-(2-(mesityl(phenyl)phosphorothioyl)ethyl)carbamate (3'e)

40.1 mg, 99% yield, white solid

^{1}H NMR (400 MHz, $CDCl_3$) δ 7.84 – 7.73 (m, 2H), 7.54 – 7.40 (m, 3H), 6.82 (d, *J* = 4.1 Hz, 2H), 5.03 (s, 1H), 3.15 – 3.11 (m, 2H), 2.90 – 2.71 (m, 2H), 2.28 (s, 6H), 2.25 (s, 3H), 1.37 (s, 9H).

^{13}C NMR (101 MHz, $CDCl_3$) δ 141.61 (d, *J* = 10.3 Hz), 141.22 (d, *J* = 2.7 Hz), 134.46 (d, *J* = 77.5 Hz), 131.53 (d, *J* = 11.2 Hz), 131.28 (d, *J* = 2.8 Hz), 130.83 (d, *J* = 10.5 Hz), 128.86 (d, *J* = 12.1 Hz), 128.63 (d, *J* = 83.8 Hz), 28.47, 24.13 (d, *J* = 5.4 Hz), 20.96 (d, *J* = 1.5 Hz).(two carbon are missing)

^{31}P NMR (162 MHz, $CDCl_3$) δ 40.46.

HRMS (ESI, m/Z): calcd. for $C_{22}H_{30}NNaO_2PS^+$ [M+Na]$^+$: 426.1627, found: 426.1621.

Melting point: 51.1 ^{o}C

Specific rotation: $[\alpha]_D^{20}$= -59.76 (c = 1.004 g/100mL, $CHCl_3$).

SFC Chiralcel OD, CO_2/MeOH with gradient from 97% to 50% CO_2 in 4.5 min, 1.8 mL/min., 40 °C, detection at 221 nm. Retention time: 2.31 min. (minor) and 2.41min. (major). 87% *ee*.

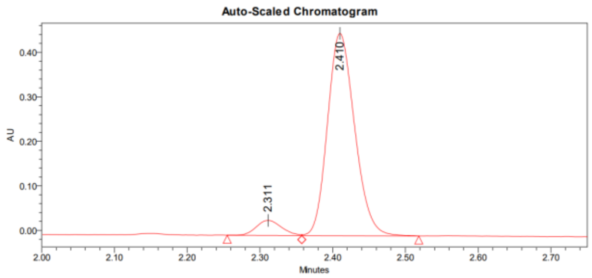

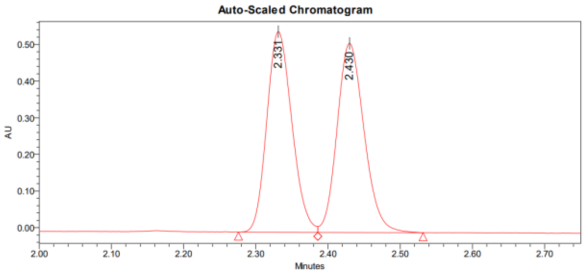

Peak Results

	Name	RT	Area	% Area	Height
1		2.311	81237	6.45	33680
2		2.410	1178651	93.55	454496

Peak Results

	Name	RT	Area	% Area	Height
1		2.331	1320338	49.58	547402
2		2.430	1342725	50.42	516046

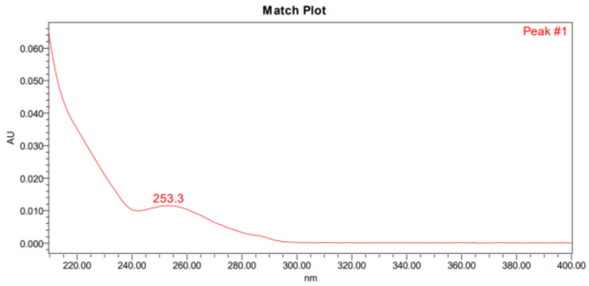

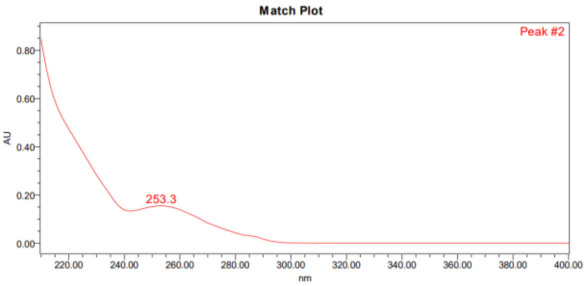

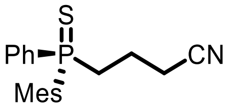

(*S*)-4-(mesityl(phenyl)phosphorothioyl)butanenitrile (3’f)

34 mg, 99% yield, sticky white solid

^{1}H NMR (400 MHz, $CDCl_3$) δ 7.82 – 7.72 (m, 2H), 7.56 – 7.41 (m, 3H), 6.85 (d, *J* = 4.1 Hz, 2H), 2.79 – 2.52 (m, 2H), 2.35 (t, *J* = 6.9 Hz, 2H), 2.29 (s, 6H), 2.27 (s, 3H), 2.14 – 1.97 (m, 1H), 1.24 – 1.08 (m, 1H).

^{13}C NMR (101 MHz, $CDCl_3$) δ 141.47 (d, *J* = 10.3 Hz), 141.25 (d, *J* = 2.9 Hz), 134.04 (d, *J* = 77.8 Hz), 131.45 (d, *J* = 11.4 Hz), 131.25 (d, *J* = 2.9 Hz), 130.66 (d, *J* = 10.3 Hz), 128.75 (d, *J* = 12.1 Hz), 128.35 (d, *J* = 82.8 Hz), 118.75, 37.10 (d, *J* = 54.7 Hz), 24.05 (d, *J* = 5.2 Hz), 20.85 (d, *J* = 1.7 Hz), 19.46, 18.14 (d, *J* = 17.7 Hz).

^{31}P NMR (162 MHz, $CDCl_3$) δ 42.36.

HRMS (ESI, m/Z): calcd. for $C_{19}H_{23}NPS^+$ $[M+H]^+$: 328.1283, found: 328.1280.

Melting point: cannot be measured

Specific rotation: $[\alpha]_{\mathrm{D}}^{20}$= -76.80 (c = 2.560 g/100mL, $CHCl_3$).

SFC Chiralcel OJ-3, CO_2/MeOH with gradient from 97% to 50% CO_2 in 4.5 min, 1.8 mL/min., 40 °C, detection at 221 nm. Retention time: 2.28 min. (minor) and 2.39 min. (major). 91% *ee*.

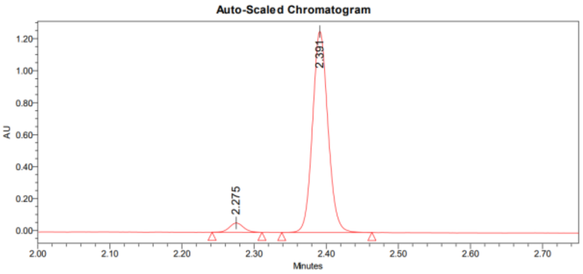

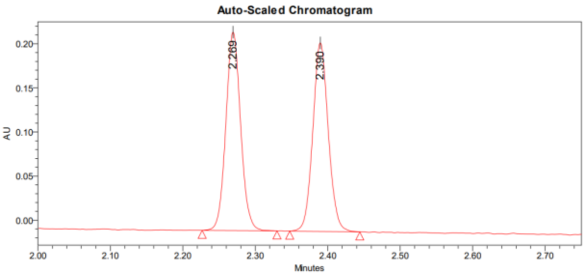

Peak Results

	Name	RT	Area	% Area	Height
1		2.275	80493	4.13	58561
2		2.391	1869873	95.87	1258431

Peak Results

	Name	RT	Area	% Area	Height
1		2.269	318739	50.11	225094
2		2.390	317292	49.89	213865

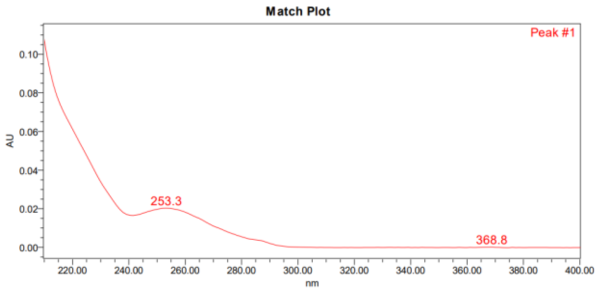

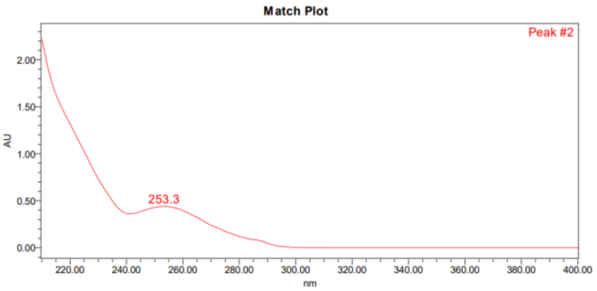

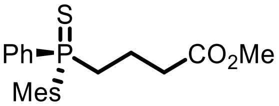

methyl (*S*)-4-(mesityl(phenyl)phosphorothioyl)butanoate (3'g)

34 mg, 95% yield, white solid

^{1}H NMR (400 MHz, $CDCl_3$) δ 7.83 – 7.72 (m, 2H), 7.53 – 7.38 (m, 3H), 6.82 (d, *J* = 4.0 Hz, 2H), 3.59 (s, 3H), 2.67 – 2.47 (m, 2H), 2.40 – 2.19 (m, 11H), 2.04 – 1.80 (m, 1H), 1.28 – 1.05 (m, 1H).

^{13}C NMR (101 MHz, $CDCl_3$) δ 173.12, 141.65 (d, *J* = 10.2 Hz), 141.02 (d, *J* = 2.9 Hz), 134.52 (d, *J* = 77.5 Hz), 131.46 (d, *J* = 11.2 Hz), 131.07 (d, *J* = 2.9 Hz), 130.87 (d, *J* = 10.2 Hz), 128.87 (d, *J* = 82.8 Hz),128.61 (d, *J* = 11.9 Hz), 51.66, 37.90 (d, *J* = 54.1 Hz), 34.63 (d, *J* = 17.5 Hz), 24.12 (d, *J* = 5.2 Hz), 20.95 (d, *J* = 1.5 Hz), 18.78 (d, *J* = 1.5 Hz).

^{31}P NMR (162 MHz, $CDCl_3$) δ 43.01.

HRMS (ESI, m/Z): calcd. for $C_{20}H_{26}O_2PS^+$ $[M+H]^+$: 361.1386, found: 361.1382.

Melting point: 69.9 °C

Specific rotation: $[\alpha]_D^{20}$= -65.83 (c = 2.558 g/100mL, $CHCl_3$).

SFC Chiralcel OJ-3, CO_2/MeOH with gradient from 97% to 50% CO_2 in 4.5 min, 1.8 mL/min., 40 °C, detection at 221 nm. Retention time: 1.95 min. (minor) and 2.00 min. (major). 91% *ee*.

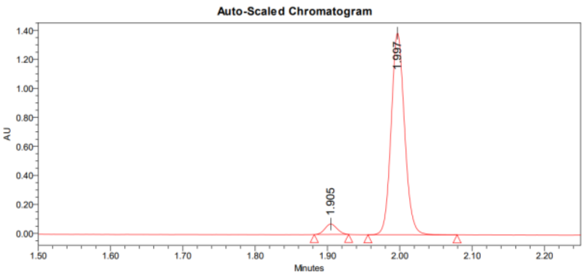

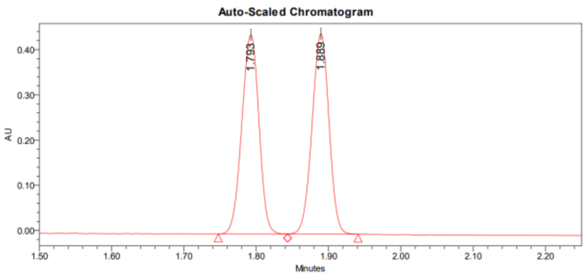

Peak Results

	Name	RT	Area	% Area	Height
1		1.905	83591	4.45	71848
2		1.997	1792878	95.55	1391005

Peak Results

	Name	RT	Area	% Area	Height
1		1.793	726674	50.10	440524
2		1.889	723684	49.90	443528

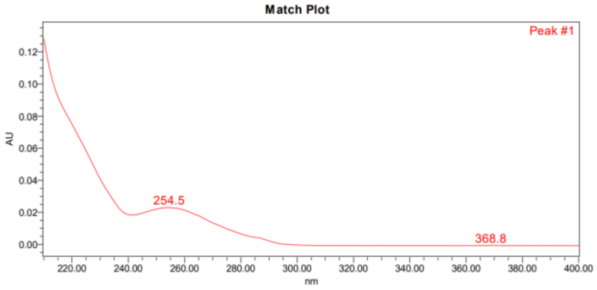

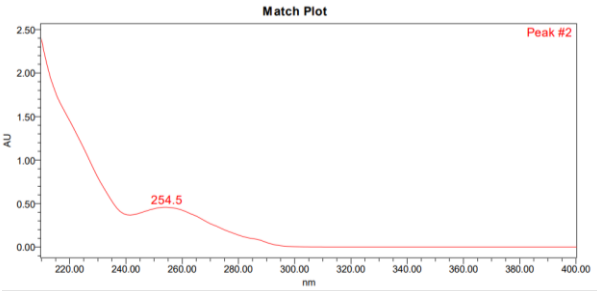

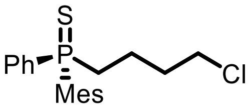

(*S*)-(4-chlorobutyl)(mesityl)(phenyl)phosphine sulfide (3'h)

20.1 mg, 57% yield, sticky white solid

^{1}H NMR (400 MHz, $CDCl_3$) δ 7.82 – 7.71 (m, 2H), 7.53 – 7.39 (m, 3H), 6.84 (d, *J* = 4.0 Hz, 2H), 3.47 – 3.32 (m, 2H), 2.66 – 2.39 (m, 2H), 2.29 (s, 6H), 2.26 (s, 3H), 1.89 – 1.64 (m, 3H), 1.06 – 0.89 (m, 1H).

^{13}C NMR (101 MHz, $CDCl_3$) δ 141.67 (d, *J* = 10.2 Hz), 134.61 (d, *J* = 77.5 Hz), 131.51 (d, *J* = 11.2 Hz), 131.07 (d, *J* = 2.9 Hz), 130.83 (d, *J* = 10.1 Hz), 128.92 (d, *J* = 82.8 Hz), 128.63 (d, *J* = 12.1 Hz), 44.18, 38.07 (d, *J* = 53.6 Hz), 33.58 (d, *J* = 17.0 Hz), 24.16 (d, *J* = 5.1 Hz), 20.98 (d, *J* = 1.5 Hz), 20.74 (d, *J* = 2.3 Hz).

^{31}P NMR (162 MHz, $CDCl_3$) δ 43.25.

HRMS (ESI, m/Z): calcd. for $C_{19}H_{25}ClPS^{+}$ $[M+H]^{+}$: 351.1098, found: 351.1092.

Melting point: cannot be measured

Specific rotation: $[\alpha]_{D}^{20}$= -72.03 (c = 1.405 g/100mL, $CHCl_3$).

SFC Chiralcel OD, CO_2/MeOH with gradient from 97% to 50% CO_2 in 4.5 min, 1.8 mL/min., 40 °C, detection at 221 nm. Retention time: 2.12 min. (major) and 2.44 min. (minor). 92% *ee*.

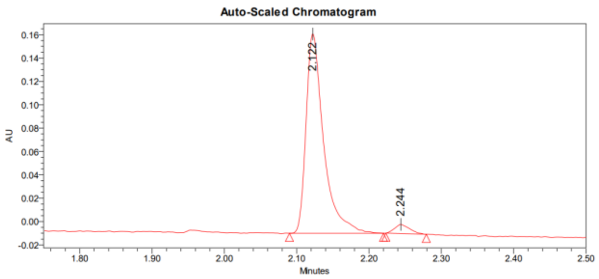

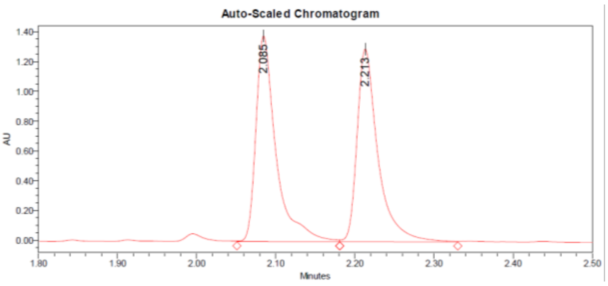

Peak Results

	Name	RT	Area	% Area	Height
1		2.122	288321	96.28	170748
2		2.244	11154	3.72	7825

Peak Results

	Name	RT	Area	% Area	Height
1		2.085	2454133	50.82	1382241
2		2.213	2374666	49.18	1294379

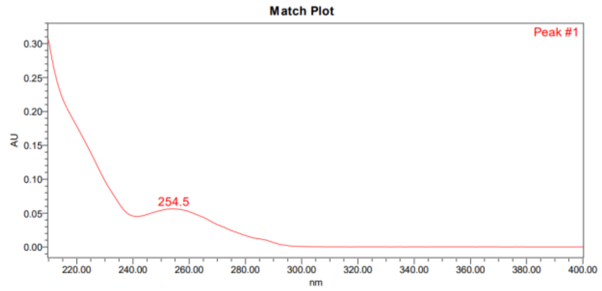

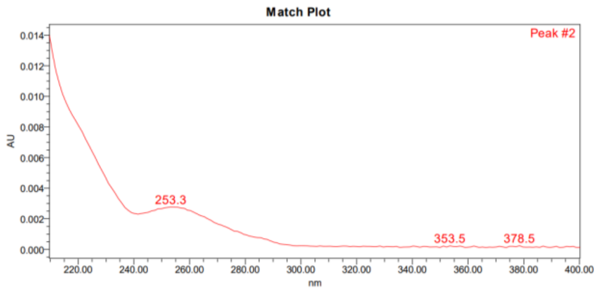

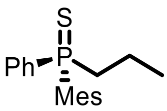

(*S*)-mesityl(phenyl)(propyl)phosphine sulfide (4'a)

54.5 mg, 90% yield, white solid

^{1}H NMR (400 MHz, Chloroform-*d*) δ 7.83 – 7.70 (m, 2H), 7.52 – 7.38 (m, 3H), 6.83 (d, *J* = 3.9 Hz, 2H), 2.62 – 2.36 (m, 2H), 2.29 (s, 6H), 2.25 (s, 3H), 1.80 – 1.58 (m, 1H), 0.94 – 0.69 (m, 4H).

^{13}C NMR (101 MHz, $CDCl_3$) δ 141.67 (d, *J* = 10.0 Hz), 140.87 (d, *J* = 2.9 Hz), 134.93 (d, *J* = 77.2 Hz), 131.41 (d, *J* = 11.3 Hz), 130.83 (d, *J* = 3.0 Hz), 130.79 (d, *J* = 10.1 Hz), 129.07 (d, *J* = 81.7 Hz), 128.46 (d, *J* = 12.1 Hz), 41.04 (d, *J* = 53.1 Hz), 24.09 (d, *J* = 5.1 Hz), 20.94 (d, *J* = 1.5 Hz), 16.78 (d, *J* = 3.0 Hz), 15.56 (d, *J* = 19.0 Hz).

^{31}P NMR (162 MHz, Chloroform-*d*) δ 43.29.

HRMS (ESI, m/Z): calcd. for $C_{18}H_{24}PS^+$ $[M+H]^+$: 303.1331, found: 303.1327.

Melting point: 87.6 ^{o}C

Specific rotation: $[\alpha]_D^{20}$= -77.14 (c = 3.770 g/100mL, $CHCl_3$).

SFC Chiralcel OD, CO_2/MeOH with gradient from 97% to 50% CO_2 in 4.5 min, 1.8 mL/min., 40 ^{o}C, detection at 221 nm. Retention time: 2.01 min. (major) and 2.08 min. (minor). 90% *ee*.

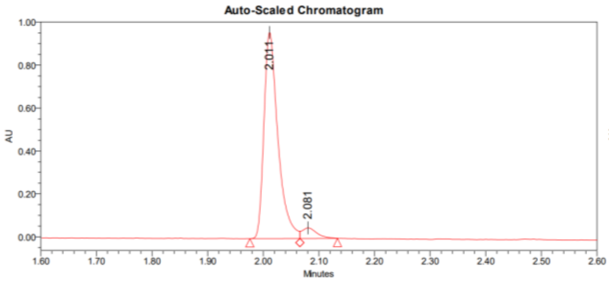

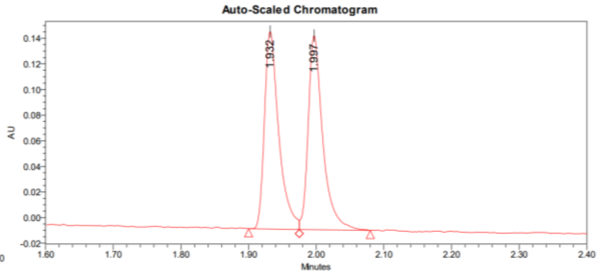

Peak Results

	Name	RT	Area	% Area	Height
1		2.011	1675441	94.81	960275
2		2.081	91692	5.19	49347

Peak Results

	Name	RT	Area	% Area	Height
1		1.932	227256	49.98	154314
2		1.997	227400	50.02	151653

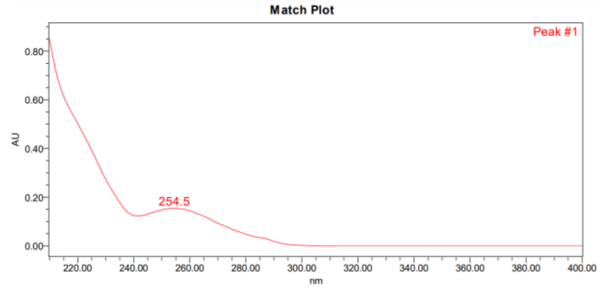

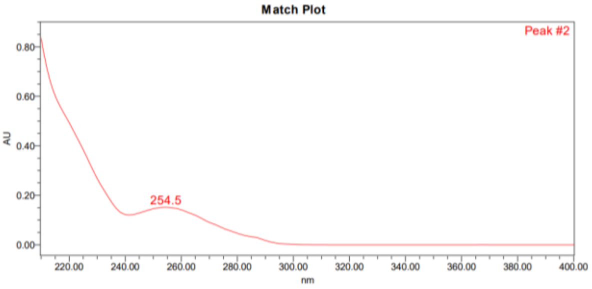

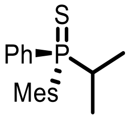

(*S*)-isopropyl(mesityl)(phenyl)phosphine sulfide (4’b)

27.1 mg, 90% yield, white solid

^{1}H NMR (400 MHz, $CDCl_3$) δ 7.83 – 7.73 (m, 2H), 7.55 – 7.38 (m, 3H), 6.85 (d, *J* = 3.9 Hz, 2H), 2.99 (dp, *J* = 13.7, 6.9 Hz, 1H), 2.28 (s, 9H), 1.10 (dd, *J* = 17.1, 6.9 Hz, 3H), 0.96 (dd, *J* = 19.5, 6.8 Hz, 3H).

^{13}C NMR (101 MHz, $CDCl_3$) δ 142.08 (d, *J* = 9.6 Hz), 140.60 (d, *J* = 2.9 Hz), 131.93 (d, *J* = 9.2 Hz), 131.49 (d, *J* = 11.0 Hz), 131.07 (d, *J* = 73.7 Hz), 131.06 (d, *J* = 2.9 Hz), 128.02 (d, *J* = 11.5 Hz), 127.65 (d, *J* = 81.8 Hz), 34.49 (d, *J* = 51.5 Hz), 23.99 (d, *J* = 4.8 Hz), 20.94 (d, *J* = 1.4 Hz), 17.82 (d, *J* = 2.8 Hz), 16.25.

^{31}P NMR (162 MHz, $CDCl_3$) δ 51.64.

HRMS (ESI, m/Z): calcd. for $C_{18}H_{24}PS^+$ $[M+H]^+$: 303.1331, found: 303.1326.

Melting point: 153.9 ^oC

Specific rotation: $[\alpha]_D^{20}$= -60.86 (c = 2.261 g/100mL, $CHCl_3$).

SFC Chiralcel OJ-3, CO_2/MeOH with gradient from 97% to 50% CO_2 in 4.5 min, 1.8 mL/min., 40 ^oC, detection at 221 nm. Retention time: 1.84 min. (minor) and 2.05 min. (major). 88% *ee*.

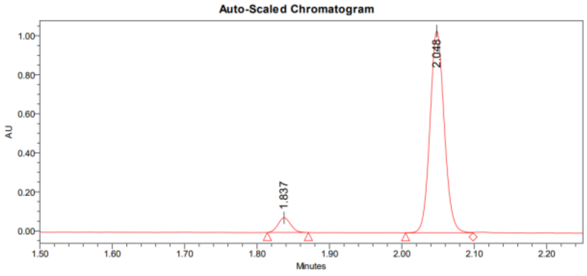

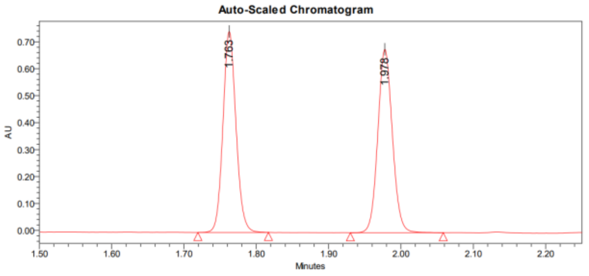

	Name	RT	Area	% Area	Height
1		1.837	91408	6.06	75138
2		2.048	1415841	93.94	1035102

Peak Results

	Name	RT	Area	% Area	Height
1		1.763	935271	49.51	745814
2		1.978	953761	50.49	681077

Peak Results

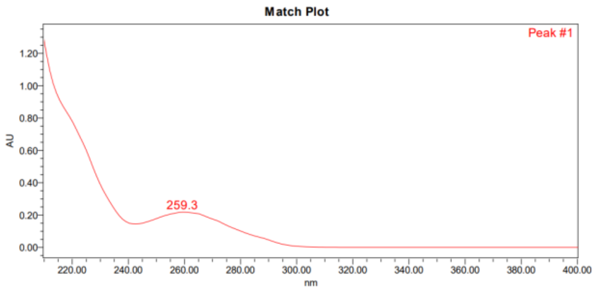

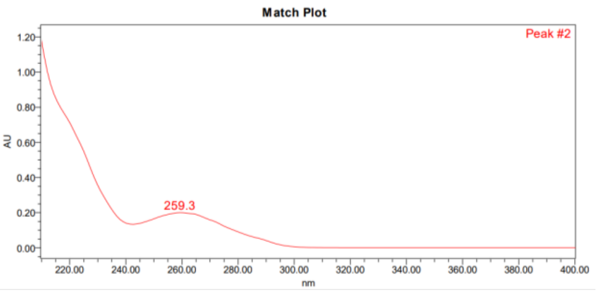

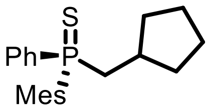

(*S*)-(cyclopentylmethyl)(mesityl)(phenyl)phosphine sulfide (4’c)

52.6 mg, 77% yield, sticky white solid

^{1}H NMR (400 MHz, Chloroform-*d*) δ 7.84 – 7.72 (m, 2H), 7.51 – 7.36 (m, 3H), 6.81 (d, J = 3.9 Hz, 2H), 2.77 (td, J = 13.9, 6.1 Hz, 1H), 2.56 (ddd, J = 13.9, 9.2, 5.8 Hz, 1H), 2.28 (s, 6H), 2.25 (s, 3H), 1.96 – 1.69 (m, 2H), 1.62 – 1.47 (m, 1H), 1.47 – 1.18 (m, 4H), 1.14 – 0.99 (m, 1H), 0.71 (dq, J = 12.5, 8.6 Hz, 1H).

^{13}C NMR (101 MHz, $CDCl_3$) δ 141.51 (d, *J* = 10.0 Hz), 140.66 (d, *J* = 2.9 Hz), 135.30 (d, *J* = 76.7 Hz), 131.43 (d, *J* = 11.0 Hz), 131.12 (d, *J* = 10.0 Hz), 130.91 (d, *J* = 2.9 Hz), 130.00 (d, *J* = 81.5 Hz), 128.48 (d, *J* = 12.1 Hz), 44.38 (d, *J* = 52.2 Hz), 35.35 (d, *J* = 3.3 Hz), 34.95 (d, *J* = 8.2 Hz), 34.42 (d, *J* = 9.2 Hz), 24.72 (d, *J* = 19.5 Hz), 24.35 (d, *J* = 4.8 Hz), 20.92 (d, *J* = 1.5 Hz).

^{31}P NMR (162 MHz, $CDCl_3$) δ 42.85.

HRMS (ESI, m/Z): calcd. for $C_{21}H_{28}PS^{+}$ $[M+H]^{+}$: 343.1644, found: 343.1638.

Melting point: cannot be measured

Specific rotation: $[\alpha]_{\mathbf{D}}^{\mathbf{20}}$= -74.78 (c = 1.741 g/100mL, $CHCl_3$).

SFC Chiralcel OD, CO_2/MeOH with gradient from 97% to 50% CO_2 in 4.5 min, 1.8 mL/min., 40 °C, detection at 221 nm. Retention time: 2.00 min. (major) and 2.15 min. (minor). 89% *ee*.

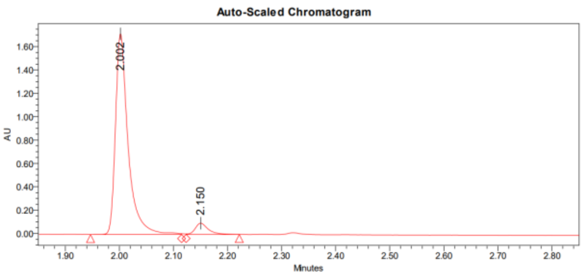

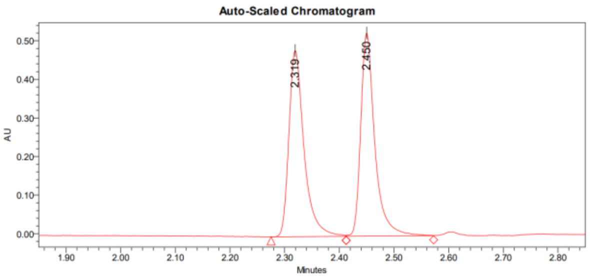

Peak Results

	Name	RT	Area	% Area	Height
1		2.002	2794221	94.49	1720497
2		2.150	162837	5.51	96724

Peak Results

	Name	RT	Area	% Area	Height
1		2.319	928197	49.64	482332
2		2.450	941734	50.36	526569

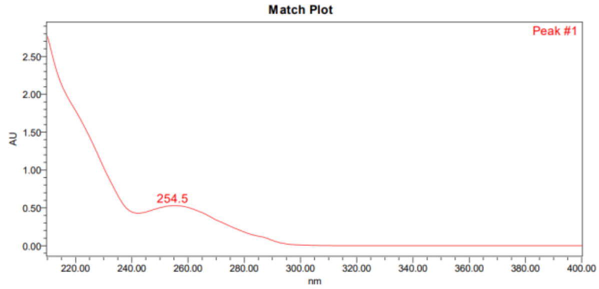

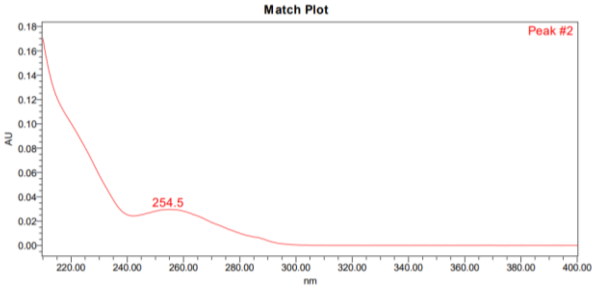

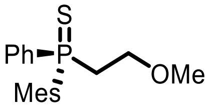

(*S*)-mesityl(2-methoxyethyl)(phenyl)phosphine sulfide (4’d)

61.6 mg, 97% yield, white solid

^{1}H NMR (400 MHz, CDCl3) δ 7.83 – 7.71 (m, 2H), 7.53 – 7.39 (m, 3H), 6.83 (d, J = 4.1 Hz, 2H), 3.72 – 3.61 (m, 1H), 3.15 (s, 3H), 3.06 – 2.85 (m, 2H), 2.83 – 2.71 (m, 1H), 2.29 (s, 6H), 2.26 (s, 3H).

^{13}C NMR (101 MHz, $CDCl_3$) δ 141.63 (d, *J* = 10.3 Hz), 141.16 (d, *J* = 2.9 Hz), 134.67 (d, *J* = 77.7 Hz), 131.52 (d, *J* = 11.4 Hz), 131.13 (d, *J* = 3.0 Hz), 130.64 (d, *J* = 10.5 Hz), 128.71 (d, *J* = 84.4 Hz), 128.66 (d, *J* = 12.2 Hz), 67.48 (d, *J* = 4.4 Hz), 58.55, 38.47 (d, *J* = 53.9 Hz), 24.05 (d, *J* = 5.5 Hz), 20.96 (d, *J* = 1.5 Hz).

^{31}P NMR (162 MHz, Chloroform-*d*) δ 37.65.

HRMS (ESI, m/Z): calcd. for $C_{18}H_{24}OPS^{+}$ $[M+H]^{+}$: 319.1280, found: 319.1276.

Melting point: 88.6 °C

Specific rotation: $[\alpha]_{\mathrm{D}}^{20}$= -84.54 (c = 4.339 g/100mL, $CHCl_3$).

SFC Chiralcel OJ-3, CO_2/MeOH with gradient from 97% to 50% CO_2 in 4.5 min, 1.8 mL/min., 40 °C, detection at 221 nm. Retention time: 1.56 min. (minor) and 1.67 min. (major). 89% *ee*.

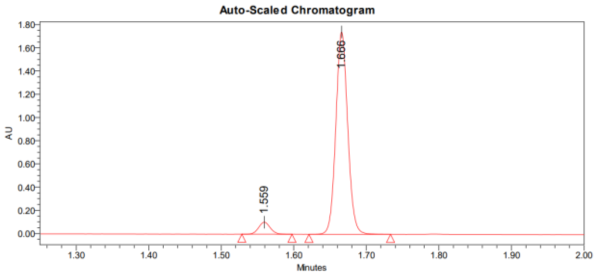

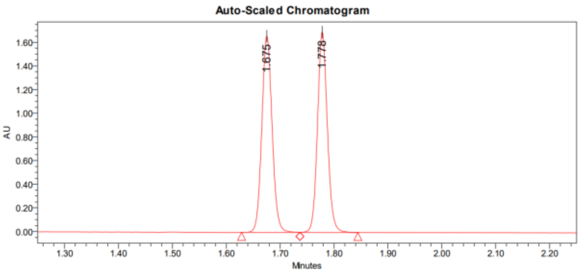

Peak Results

	Name	RT	Area	% Area	Height
1		1.559	116395	5.55	103826
2		1.666	1982591	94.45	1743692

Peak Results

	Name	RT	Area	% Area	Height
1		1.675	2205115	50.53	1660074
2		1.778	2159243	49.47	1695475

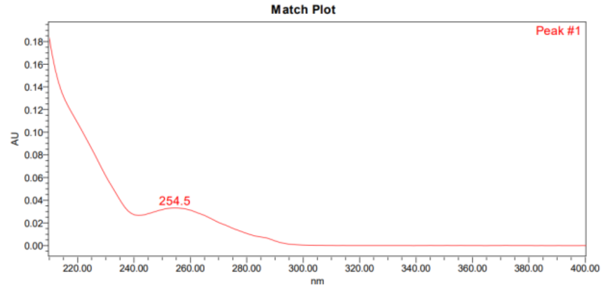

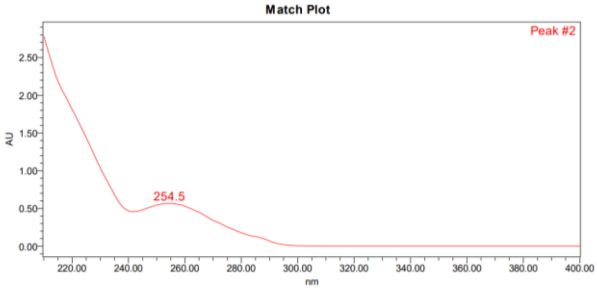

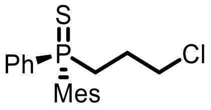

(*S*)-(3-chloropropyl)(mesityl)(phenyl)phosphine sulfide (4'e)

56 mg, 83% yield, sticky white solid

^{1}H NMR (400 MHz, Chloroform-*d*) δ 7.82 – 7.72 (m, 2H), 7.53 – 7.38 (m, 3H), 6.84 (d, *J* = 4.0 Hz, 2H), 3.48 (td, *J* = 5.8, 0.9 Hz, 2H), 2.77 – 2.57 (m, 2H), 2.30 (s, 6H), 2.26 (s, 3H), 2.24 – 2.11 (m, 1H), 1.25 – 1.12 (m, 1H).

^{13}C NMR (101 MHz, $CDCl_3$) δ 141.63 (d, *J* = 10.1 Hz), 141.14 (d, *J* = 2.9 Hz), 134.43 (d, *J* = 77.7 Hz), 131.49 (d, *J* = 11.1 Hz), 131.15 (d, *J* = 2.9 Hz), 130.79 (d, *J* = 10.1 Hz), 128.73 (d, *J* = 83.4 Hz), 128.69 (d, *J* = 11.9 Hz), 45.59 (d, *J* = 19.6 Hz), 36.17 (d, *J* = 54.9 Hz), 26.30, 24.16 (d, *J* = 5.2 Hz), 20.94.

^{31}P NMR (162 MHz, Chloroform-*d*) δ 43.06.

HRMS (ESI, m/Z): calcd. for $C_{18}H_{23}ClPS^{+}$ $[M+H]^{+}$: 337.0941, found: 337.0936.

Melting point: cannot be measured

Specific rotation: $[\alpha]_{\mathbf{D}}^{\mathbf{20}}$= -23.84 (c = 3.112 g/100mL, $CHCl_3$).

SFC Chiralcel OD, CO_2/MeOH with gradient from 97% to 50% CO_2 in 4.5 min, 1.8 mL/min., 40 ^{o}C, detection at 221 nm. Retention time: 2.21 min. (major) and 2.29 min. (minor). 90% *ee*.

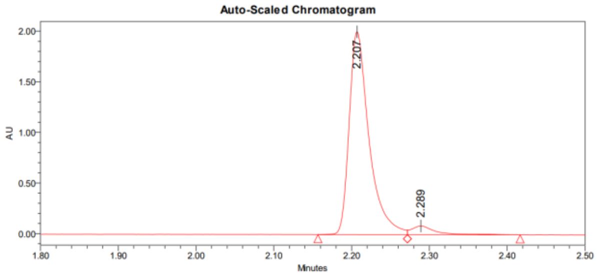

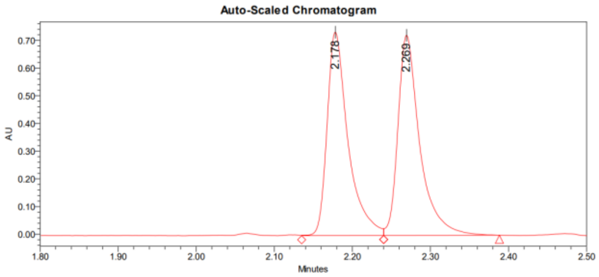

Peak Results

	Name	RT	Area	% Area	Height
1		2.207	3558741	94.94	2006293
2		2.289	189628	5.06	86485

Peak Results

	Name	RT	Area	% Area	Height
1		2.178	1370259	49.42	734582
2		2.269	1402216	50.58	722555

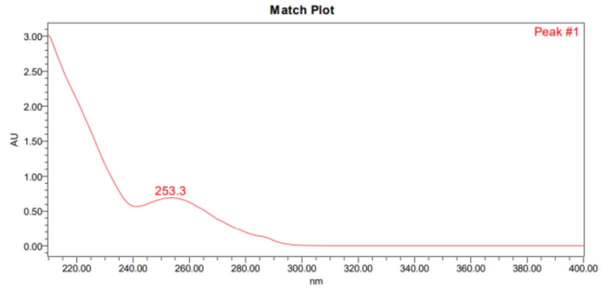

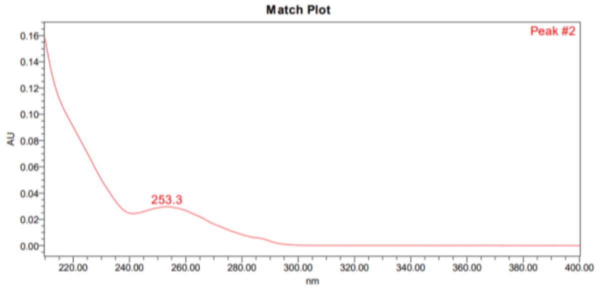

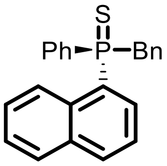

(*S*)-benzyl(naphthalen-1-yl)(phenyl)phosphine sulfide (5'a)

32.4 mg, 90% yield, white solid

^{1}H NMR (400 MHz, $CDCl_3$) δ 8.26 (d, *J* = 8.6 Hz, 1H), 8.15 (dd, *J* = 15.5, 7.2 Hz, 1H), 8.04 (d, *J* = 8.2 Hz, 1H), 7.90 (d, *J* = 8.2 Hz, 1H), 7.64 – 7.41 (m, 5H), 7.41 – 7.30 (m, 3H), 7.21 – 7.05 (m, 3H), 6.87 (dd, *J* = 7.5, 2.5 Hz, 2H), 4.16 – 3.94 (m, 2H).

^{13}C NMR (101 MHz, $CDCl_3$) δ 134.37 (d, *J* = 8.7 Hz), 133.40 (d, *J* = 3.2 Hz), 132.74 (d, *J* = 80.0 Hz), 132.28 (d, *J* = 8.0 Hz), 132.13 (d, *J* = 9.2 Hz), 131.54 (d, *J* = 3.0 Hz), 131.45 (d, *J* = 10.3 Hz), 130.80 (d, *J* = 5.2 Hz), 130.51 (d, *J* = 6.9 Hz), 129.37 (d, *J* = 1.4 Hz), 128.59 (d, *J* = 12.4 Hz), 128.53 (d, *J* = 78.8 Hz), 127.87 (d, *J* = 3.4 Hz), 127.18 (d, *J* = 3.9 Hz), 127.04 (d, *J* = 6.7 Hz), 126.98, 126.43, 124.62 (d, *J* = 13.5 Hz), 41.11 (d, *J* = 51.0 Hz).

^{31}P NMR (162 MHz, $CDCl_3$) δ 38.97.

HRMS (ESI, m/Z): calcd. for $C_{23}H_{20}PS^{+}$ $[M+H]^{+}$: 359.1018, found: 359.1014.

Melting point: 153.0 °C

Specific rotation: $[\alpha]_{D}^{20}$= -134.51 (c = 1.811 g/100mL, $CHCl_3$).

SFC Chiralcel OD, CO_2/MeOH with gradient from 97% to 50% CO_2 in 4.5 min, 1.8 mL/min., 40 °C, detection at 221 nm. Retention time: 3.55 min. (minor) and 3.70 min. (major). 78% *ee*.

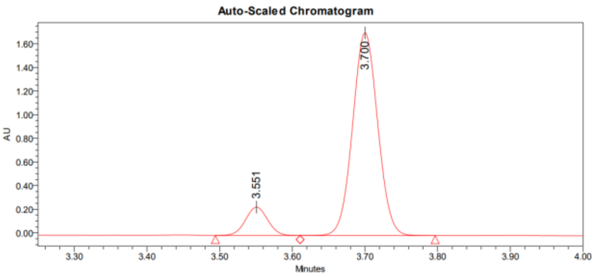

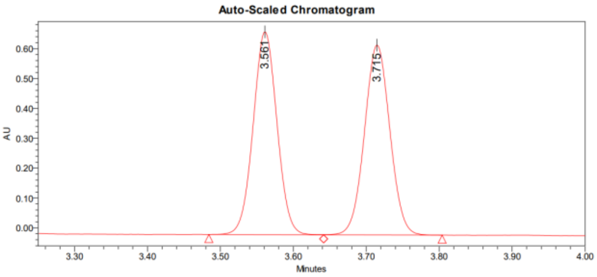

Peak Results

	Name	RT	Area	% Area	Height
1		3.551	512372	11.04	240444
2		3.700	4130569	88.96	1716794

Peak Results

	Name	RT	Area	% Area	Height
1		3.561	1542650	50.03	679258
2		3.715	1540805	49.97	635760

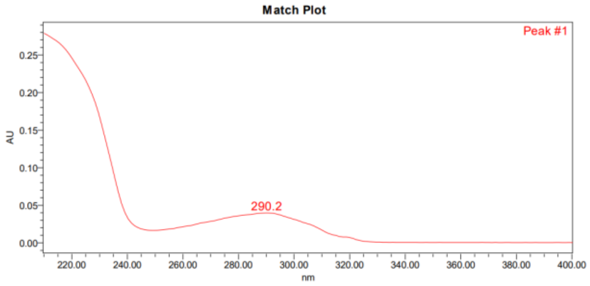

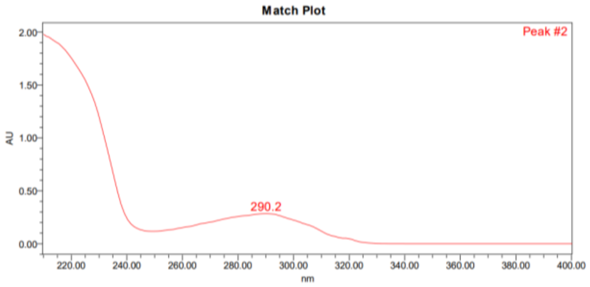

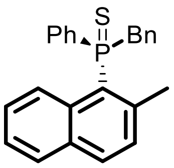

(*S*)-benzyl(2-methylnaphthalen-1-yl)(phenyl)phosphine sulfide (5'b)

30.0 mg, 88% yield, white solid

^{1}H NMR (400 MHz, $CDCl_3$) δ 8.44 (d, *J* = 8.7 Hz, 1H), 7.90 – 7.78 (m, 2H), 7.47 – 7.17 (m, 8H), 7.13 – 6.96 (m, 3H), 6.78 (dd, *J* = 7.6, 2.6 Hz, 2H), 4.28 (dd, *J* = 15.3, 13.3 Hz, 1H), 3.77 (dd, *J* = 13.3, 10.9 Hz, 1H), 2.52 (s, 3H).

^{13}C NMR (101 MHz, $CDCl_3$) δ 142.02 (d, *J* = 8.1 Hz), 133.86 (d, *J* = 80.8 Hz), 133.37 (d, *J* = 9.6 Hz), 132.74 (d, *J* = 8.9 Hz), 132.08 (d, *J* = 3.2 Hz), 131.42 (d, *J* = 7.2 Hz), 131.17 (d, *J* = 9.7 Hz), 130.86 (d, *J* = 3.0 Hz), 130.71 (d, *J* = 5.5 Hz), 130.36 (d, *J* = 12.4 Hz), 128.76 (d, *J* = 1.3 Hz), 128.11 (d, *J* = 12.5 Hz), 128.03 (d, *J* = 80.8Hz), 127.64 (d, *J* = 3.5 Hz), 127.28 (d, *J* = 7.1 Hz), 126.85 (d, *J* = 4.0 Hz), 126.09, 125.50, 45.73 (d, *J* = 48.3 Hz), 25.18 (d, *J* = 5.5 Hz).

^{31}P NMR (162 MHz, $CDCl_3$) δ 40.77.

HRMS (ESI, m/Z): calcd. for $C_{24}H_{22}PS^+$ $[M+H]^+$: 373.1174, found: 373.1172.

Melting point: 112.9 ^{o}C

Specific rotation: $[\alpha]_D^{20}$= -97.48 (c = 2.698 g/100mL, $CHCl_3$).

SFC Chiralcel OD, CO_2/MeOH with gradient from 97% to 50% CO_2 in 4.5 min, 1.8 mL/min., 40 ^{o}C, detection at 221 nm. Retention time: 2.94 min. (major) and 3.17 min. (minor). 90% *ee*.

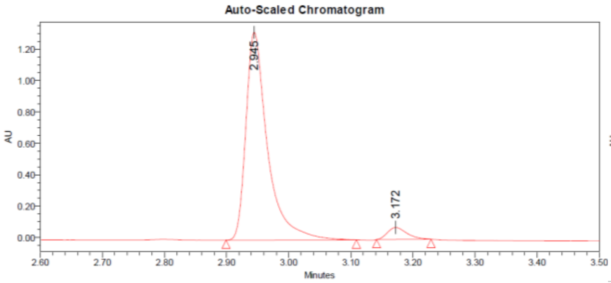

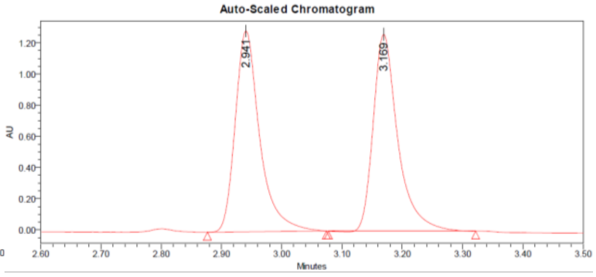

Peak Results

	Name	RT	Area	% Area	Height
1		2.945	3203126	95.11	1324353
2		3.172	164756	4.89	75729

Peak Results

	Name	RT	Area	% Area	Height
1		2.941	3641039	50.38	1288111
2		3.169	3585961	49.62	1261428

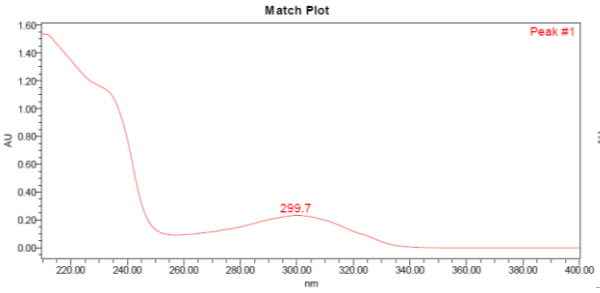

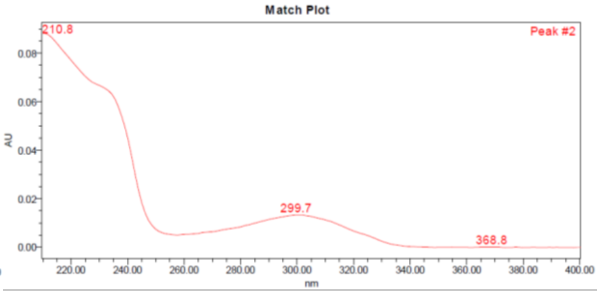

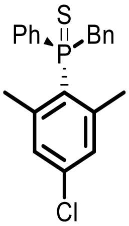

(*S*)-benzyl(4-chloro-2,6-dimethylphenyl)(phenyl)phosphine sulfide (5'c)

24.5 mg, 67% yield, white solid

^{1}H NMR (400 MHz, $CDCl_3$) δ 7.45 – 7.33 (m, 3H), 7.30 – 7.20 (m, 2H), 7.12 – 7.04 (m, 3H), 7.00 (t, *J* = 7.3 Hz, 2H), 6.80 – 6.73 (m, 2H), 4.03 (dd, *J* = 15.9, 12.9 Hz, 1H), 3.71 (dd, *J* = 13.0, 10.7 Hz, 1H), 2.37 (s, 6H).

^{13}C NMR (101 MHz, $CDCl_3$) δ 143.67 (d, *J* = 10.6 Hz), 136.72 (d, *J* = 3.3 Hz), 133.42 (d, *J* = 80.7 Hz), 131.54 (d, *J* = 79.6 Hz), 131.05 (d, *J* = 2.9 Hz), 130.84 (d, *J* = 2.9 Hz), 130.80 (d, *J* = 7.2 Hz), 130.76 (d, *J* = 7.3 Hz), 130.35 (d, *J* = 11.0 Hz), 128.25 (d, *J* = 12.5 Hz), 127.57 (d, *J* = 3.7 Hz), 126.91 (d, *J* = 4.0 Hz), 24.46 (d, *J* = 4.5 Hz).

^{31}P NMR (162 MHz, $CDCl_3$) δ 43.15.

HRMS (ESI, m/Z): calcd. for $C_{21}H_{21}ClPS^+$ $[M+H]^+$: 371.0785, found: 371.0782.

Melting point: 163.5 ^{o}C

Specific rotation: $[\alpha]_{\mathbf{D}}^{\mathbf{20}}$= -168.27 (c = 2.408 g/100mL, $CHCl_3$).

SFC Chiralcel OD, CO_2/MeOH with gradient from 97% to 50% CO_2 in 4.5 min, 1.8 mL/min., 40 ^{o}C, detection at 221 nm. Retention time: 2.60 min. (major) and 2.88 min. (minor). 82% *ee*.

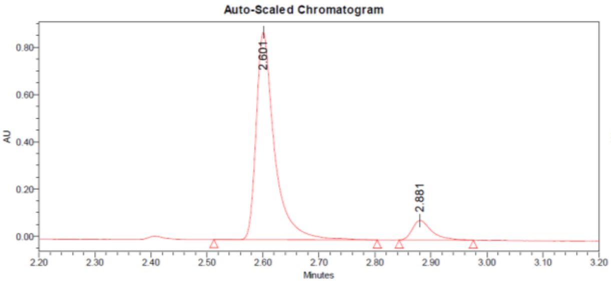

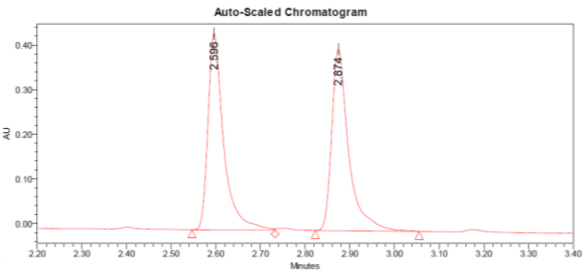

Peak Results

	Name	RT	Area	% Area	Height
1		2.601	1991964	90.92	878057
2		2.881	198876	9.08	83052

Peak Results

	Name	RT	Area	% Area	Height
1		2.596	1073105	49.97	440006
2		2.874	1074520	50.03	406621

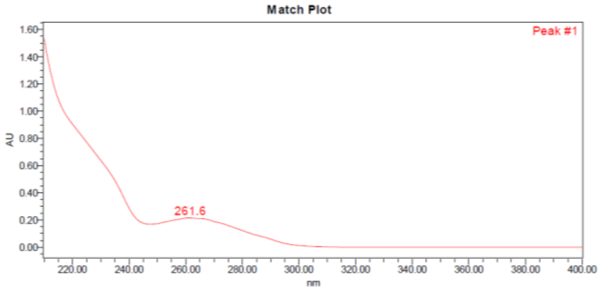

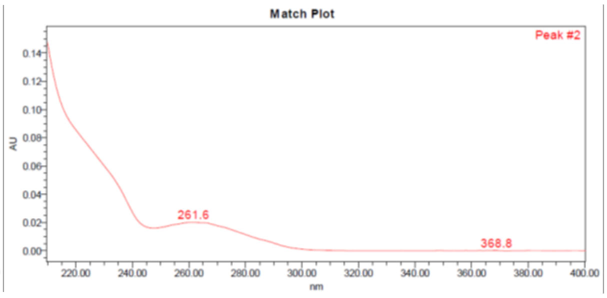

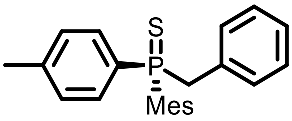

(S)-benzyl(mesityl)(p-tolyl)phosphine sulfide (5’d)

33 mg, 91% yield, white solid

^{1}H NMR (400 MHz, $CDCl_3$) δ 7.36 – 7.27 (m, 2H), 7.13 – 6.96 (m, 5H), 6.88 (d, *J* = 4.0 Hz, 2H), 6.83 – 6.75 (m, 2H), 4.02 (dd, *J* = 15.8, 13.0 Hz, 1H), 3.71 (dd, *J* = 13.0, 10.7 Hz, 1H), 2.36 (s, 6H), 2.34 (s, 3H), 2.29 (s, 3H).

^{13}C NMR (101 MHz, $CDCl_3$) δ 141.72 (d, *J* = 10.0 Hz), 141.11 (d, *J* = 3.0 Hz), 140.87 (d, *J* = 2.9 Hz), 131.50 (d, *J* = 11.2 Hz), 131.44 (d, *J* = 7.1 Hz), 130.95 (d, *J* = 7.1 Hz), 130.87 (d, *J* = 2.4 Hz), 130.65 (d, *J* = 82.8 Hz), 129.63 (d, *J* = 80.3 Hz), 128.83 (d, *J* = 12.8 Hz), 127.45 (d, *J* = 3.4 Hz), 126.69 (d, *J* = 4.0 Hz), 45.76 (d, *J* = 47.3 Hz), 24.52 (d, *J* = 4.7 Hz), 21.53 (d, *J* = 1.5 Hz), 21.00 (d, *J* = 1.4 Hz).

^{31}P NMR (162 MHz, $CDCl_3$) δ 42.94.

HRMS (ESI, m/Z): calcd. for $C_{23}H_{26}PS^+$ $[M+H]^+$: 365.1487, found: 365.1484.

Melting point: 137.1 °C

Specific rotation: $[\alpha]_D^{20}$= -226.96 (c = 2.396 g/100mL, $CHCl_3$).

SFC Chiralcel OJ-3, CO_2/MeOH with gradient from 97% to 50% CO_2 in 4.5 min, 1.8 mL/min., 40 °C, detection at 221 nm. Retention time: 2.68 min. (minor) and 2.85 min. (major). 91% *ee*.

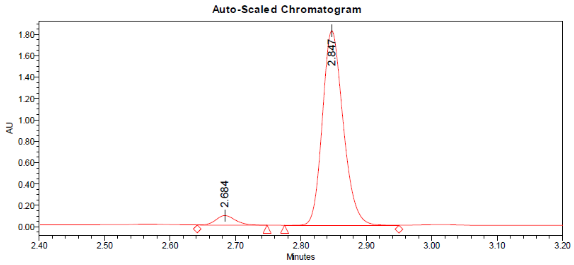

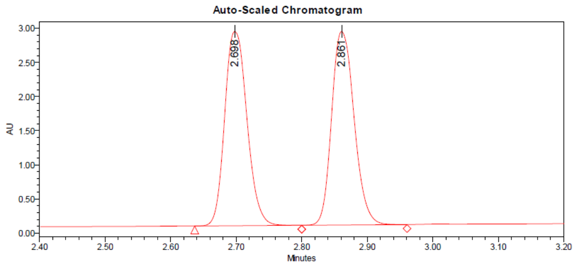

Peak Results

	Name	RT	Area	% Area	Height
1		2.684	185965	4.44	89774
2		2.847	4006015	95.56	1822472

Peak Results

	Name	RT	Area	% Area	Height
1		2.698	6728736	49.82	2843018
2		2.861	6776403	50.18	2834860

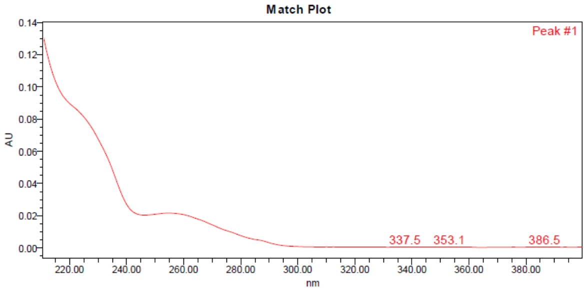

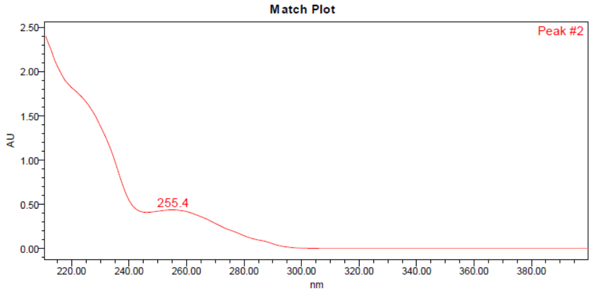

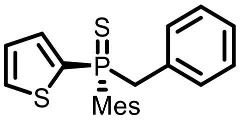

(*R*)-benzyl(mesityl)(thiophen-2-yl)phosphine sulfide(5'e)

32 mg, 90% yield, white solid

^{1}H NMR (400 MHz, $CDCl_3$) δ 7.52 (ddd, *J* = 4.9, 3.8, 1.2 Hz, 1H), 7.19 – 7.04 (m, 4H), 6.99 – 6.87 (m, 5H), 4.06 (dd, *J* = 15.2, 13.1 Hz, 1H), 3.78 (dd, *J* = 13.1, 11.2 Hz, 1H), 2.44 (s, 6H), 2.30 (s, 3H).

^{13}C NMR (101 MHz, $CDCl_3$) δ 141.74 (d, *J* = 10.7 Hz), 141.26 (d, *J* = 2.9 Hz), 137.39 (d, *J* = 87.7 Hz), 135.66 (d, *J* = 8.9 Hz), 132.94 (d, *J* = 4.4 Hz), 131.58 (d, *J* = 11.3 Hz), 131.48 (d, *J* = 7.1 Hz),

130.59 (d, *J* = 5.8 Hz), 130.03 (d, *J* = 83.0 Hz), 127.92 (d, *J* = 13.9 Hz), 127.69 (d, *J* = 3.4 Hz), 126.98 (d, *J* = 4.1 Hz), 47.23 (d, *J* = 50.6 Hz), 24.19 (d, *J* = 4.9 Hz), 21.02 (d, *J* = 1.5 Hz).

^{31}P NMR (162 MHz, cdcl$_3$) δ 34.64.

HRMS (ESI, m/Z): calcd. for $C_{20}H_{22}PS_2^+$ [M+H]$^+$: 357.0895, found: 357.0890.

Melting point: 127.3 °C

Specific rotation: $[\alpha]_D^{20}$= -129.38 (c = 1.991 g/100mL, CHCl$_3$).

SFC Chiralcel OD, CO_2/MeOH with gradient from 97% to 50% CO_2 in 4.5 min, 1.8 mL/min., 40 °C, detection at 221 nm. Retention time: 2.85 min. (major) and 3.19 min. (minor). 89% *ee*.

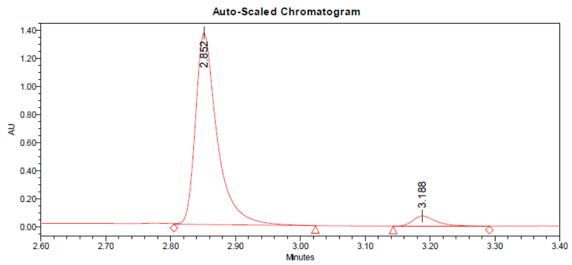

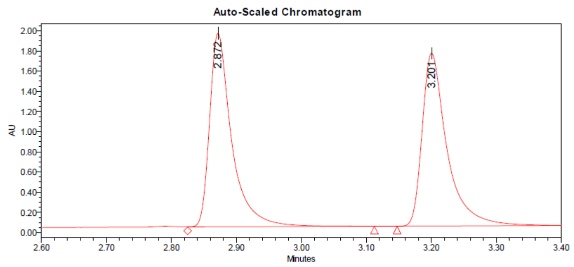

Peak Results

	Name	RT	Area	% Area	Height
1		2.852	3177103	94.57	1366435
2		3.188	182282	5.43	72537

Peak Results

	Name	RT	Area	% Area	Height
1		2.872	4545584	49.87	1918584
2		3.201	4568684	50.13	1712980

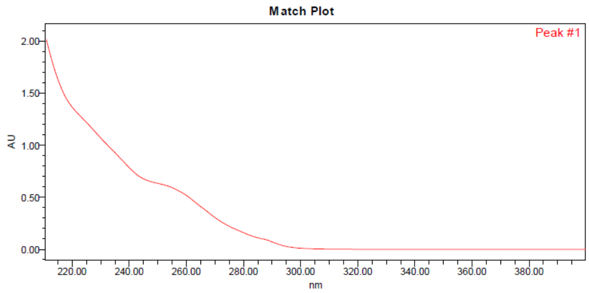

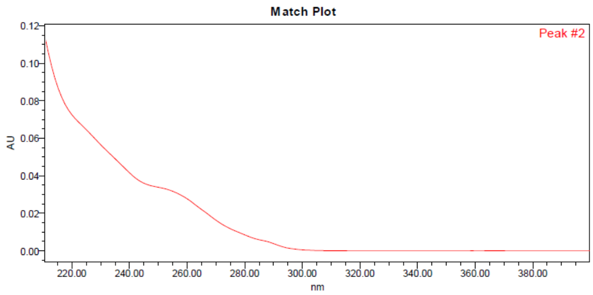

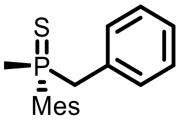

(R)-benzyl(mesityl)(methyl)phosphine sulfide(5’f)

7 mg, 25% yield, white solid

^{1}H NMR (400 MHz, $CDCl_3$) δ 7.29 – 7.16 (m, 3H), 7.02 – 6.95 (m, 2H), 6.88 (d, *J* = 3.9 Hz, 2H), 3.84 (t, *J* = 13.9 Hz, 1H), 3.51 (t, *J* = 14.6 Hz, 1H), 2.61 (s, 6H), 2.29 (s, 3H), 2.02 (d, *J* = 12.8 Hz, 3H).

^{13}C NMR (101 MHz, $CDCl_3$) δ 141.33 (d, *J* = 10.0 Hz), 141.03 (d, *J* = 3.0 Hz), 132.96 (d, *J* = 8.3 Hz), 131.84 (d, *J* = 11.2 Hz), 129.90 (d, *J* = 5.6 Hz), 128.67 (d, *J* = 3.0 Hz), 127.38 (d, *J* = 3.7 Hz), 127.17 (d, *J* = 75.8 Hz), 44.51 (d, *J* = 47.3 Hz), 25.65 (d, *J* = 56.6 Hz), 24.62 (d, *J* = 4.2 Hz), 20.97 (d, *J* = 1.6 Hz).

^{31}P NMR (162 MHz, $CDCl_3$) δ 38.98.

HRMS (ESI, m/Z): calcd. for $C_{17}H_{22}PS^{+}$ $[M+H]^{+}$: 289.1174, found: 289.1171.

Melting point: 123.2 °C

Specific rotation: $[\alpha]_{\mathbf{D}}^{\mathbf{20}}$= -52.13 (c = 0.211 g/100mL, $CHCl_3$).

SFC Chiralcel OD, CO_2/MeOH with gradient from 97% to 50% CO_2 in 4.5 min, 1.8 mL/min., 40 °C, detection at 221 nm. Retention time: 2.47 min. (major) and 2.56 min. (minor). 64% *ee*.

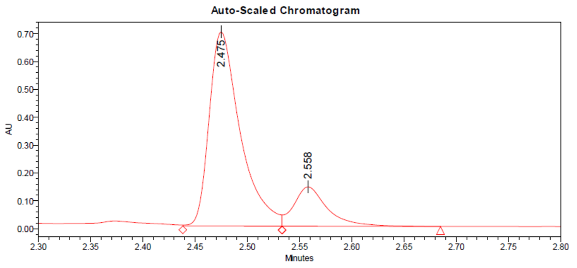

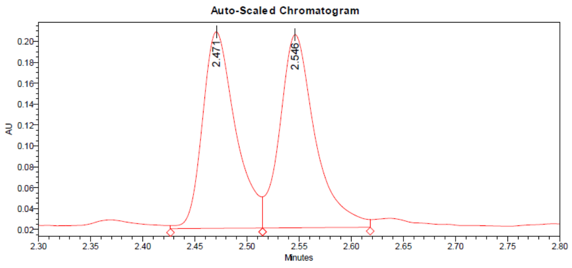

Peak Results

	Name	RT	Area	% Area	Height
1		2.475	1439731	82.06	697203
2		2.558	314819	17.94	141324

Peak Results

	Name	RT	Area	% Area	Height
1		2.471	415336	48.70	188250
2		2.546	437454	51.30	184975

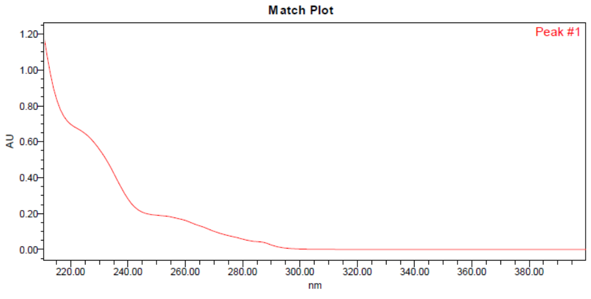

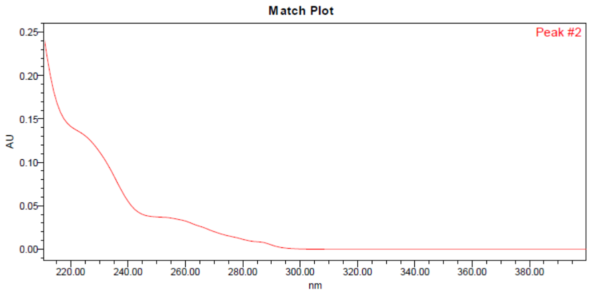

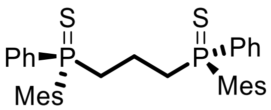

(1*S*,1'*S*)-propane-1,3-diylbis(mesityl(phenyl)phosphine sulfide) (6'a)

49.1 mg, 88% yield, white solid

^{1}H NMR (400 MHz, $CDCl_3$) δ 7.54 – 7.45 (m, 4H), 7.43 – 7.34 (m, 2H), 7.33 – 7.24 (m, 4H), 6.80 (d, *J* = 3.9 Hz, 4H), 2.85 – 2.71 (m, 2H), 2.63 – 2.48 (m, 2H), 2.29 – 2.21 (m, 18H), 1.23 – 1.04 (m, 2H).

^{13}C NMR (101 MHz, $CDCl_3$) δ 141.54 (d, *J* = 10.5 Hz), 140.97 (d, *J* = 2.9 Hz), 134.01 (d, *J* = 77.4 Hz), 131.42 (d, *J* = 11.6 Hz), 130.76 (d, *J* = 2.7 Hz), 130.65 (d, *J* = 10.5 Hz), 128.91(d, *J* = 82.8 Hz), 128.48 (d, *J* = 12.3 Hz), 39.56 (d, *J* = 13.0 Hz), 39.03 (d, *J* = 12.9 Hz), 24.11 (d, *J* = 5.3 Hz), 20.94, 17.92.

^{31}P NMR (162 MHz, $CDCl_3$) δ 42.65.

HRMS (ESI, m/Z): calcd. for $C_{33}H_{39}P_2S_2^+$ $[M+H]^+$: 561.1963, found: 561.1960.

Melting point: 199.7 ^{o}C

Specific rotation: $[\alpha]_D^{20}$= -111.11 (c = 0.108 g/100mL, $CHCl_3$).

SFC Chiralcel AS-3, CO_2/MeOH with gradient from 97% to 50% CO_2 in 4.5 min, 1.8 mL/min., 40 ^{o}C, detection at 221 nm. Retention time: 3.04 min. (minor) and 3.45 min. (major). 99% *ee*, 2.8:1 *dr*.

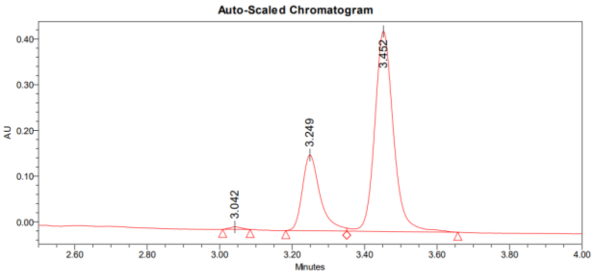

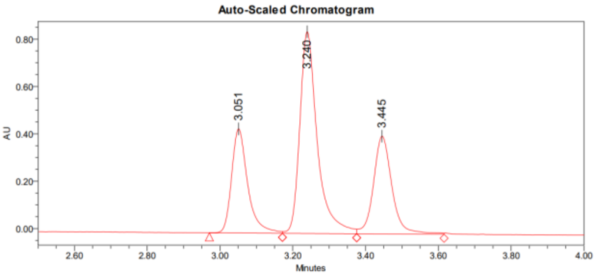

Peak Results

	Name	RT	Area	% Area	Height
1		3.042	12177	0.57	5211
2		3.249	561539	26.17	165711
3		3.452	1571931	73.26	437872

Peak Results

	Name	RT	Area	% Area	Height
1		3.051	1353349	23.95	439971
2		3.240	2848245	50.40	851924
3		3.445	1449944	25.66	412719

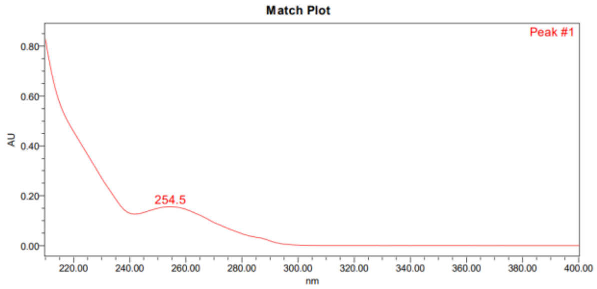

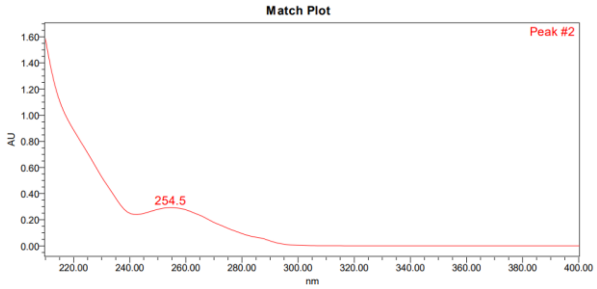

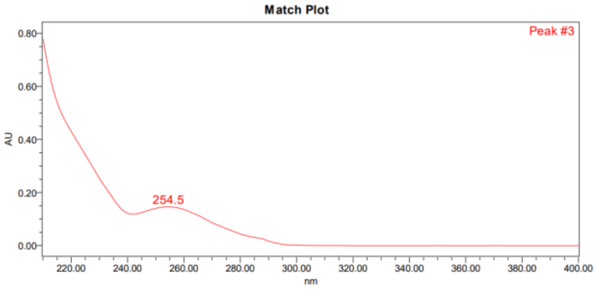

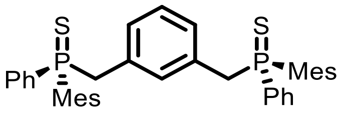

(1*S*,1'*S*)-(1,3-phenylenebis(methylene))bis(mesityl(phenyl)phosphine sulfide) (6'b)

54.4 mg, 87% yield, white solid

^{1}H NMR (400 MHz, CDCl$_3$) δ 7.43 – 7.29 (m, 6H), 7.27 – 7.17 (m, 4H), 6.86 (d, J = 3.8 Hz, 4H), 6.64 (q, J = 7.1 Hz, 1H), 6.57 – 6.49 (m, 2H), 5.99 (s, 1H), 3.85 (dd, J = 15.9, 13.1 Hz, 2H), 3.39 (dd, J = 13.1, 10.6 Hz, 2H), 2.32 – 2.26 (m, 18H).

^{13}C NMR (101 MHz, CDCl$_3$) δ 141.95 – 141.42 (m), 141.04 – 140.81 (m), 134.27 (d, *J* = 79.9 Hz), 133.61 (t, *J* = 5.7 Hz), 131.86 – 131.33 (m), 131.22 – 130.84 (m), 130.54 (t, *J* = 1.5 Hz), 130.40 – 130.06 (m), 129.15 (t, *J* = 4.6 Hz), 129.36 (d, *J* = 80.8 Hz), 127.99 (d, *J* = 12.8 Hz), 126.52 (t, *J* = 3.4 Hz), 45.30 (d, *J* = 47.4 Hz), 24.76 – 23.53 (m), 20.97.

^{31}P NMR (162 MHz, CDCl$_3$) δ 42.68.

HRMS (ESI, m/Z): calcd. for $C_{38}H_{41}P_2S_2^+$ [M+H]$^+$: 623.2119, found: 623.2112.

Melting point: 129.4 ^{o}C

Specific rotation: $[\alpha]_{\mathbf{D}}^{\mathbf{20}}$= -176.41 (c = 3.111 g/100mL, CHCl$_3$).

HPLC CHIRALPAK OD-H, heptane/*i*PrOH = 95/5, flow rate: 0.5 mL/min, 40 ^{o}C, detection at 221 nm. Retention time: 30.67 min. (major) and 52.00 min. (minor). 99% *ee*, 10:1 *dr*.

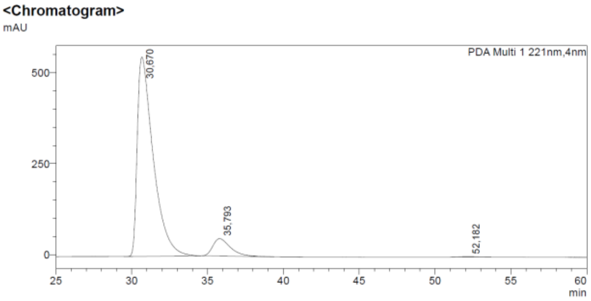

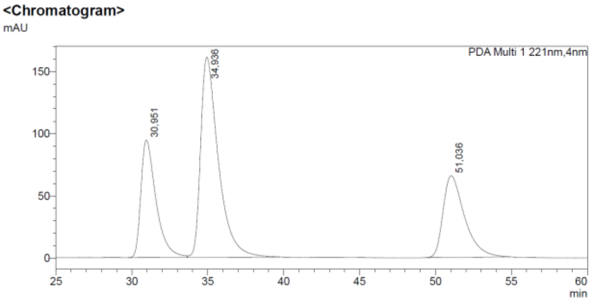

<Peak Table>

PDA Ch1 221nm

Peak#	Ret. Time	Area	Height	Conc.	Area%
1	30,670	40154079	548027	0,000	90,981
2	35,793	3870899	47799	0,000	8,771
3	52,182	109412	1345	0,000	0,248
Total		44134390	597170		100,000

<Peak Table>

PDA Ch1 221nm

Peak#	Ret. Time	Area	Height	Area%
1	30,951	6555404	94605	25,099
2	34,936	13088522	161086	50,113
3	51,036	6474214	65817	24,788
Total		26118140	321508	100,000

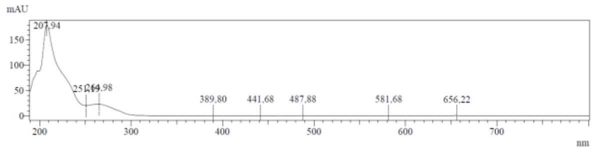

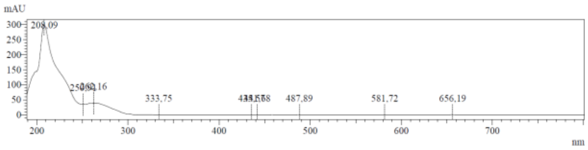

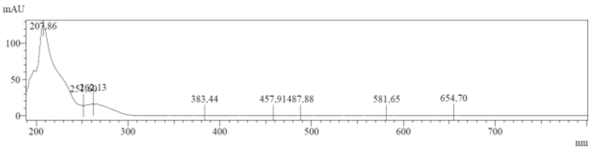

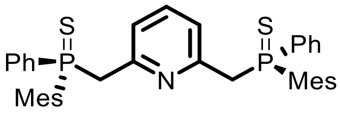

(1*S*,1'*S*)-(pyridine-2,6-diylbis(methylene))bis(mesityl(phenyl)phosphine sulfide) (6'c)

62.1 mg, 99% yield, white solid

^{1}H NMR (400 MHz, $CDCl_3$) δ 7.36 – 7.24 (m, 6H), 7.20 – 7.11 (m, 5H), 6.97 (dt, *J* = 7.7, 2.3 Hz, 2H), 6.84 (d, *J* = 4.0 Hz, 4H), 3.74 – 3.52 (m, 4H), 2.27 (s, 18H).

^{13}C NMR (101 MHz, $CDCl_3$) δ 151.28 (d, *J* = 9.5 Hz), 141.69 (d, *J* = 10.4 Hz), 140.94 (d, *J* = 2.5 Hz), 134.89 (t, *J* = 2.9 Hz), 133.46 (d, *J* = 80.3 Hz), 131.34 (d, *J* = 11.5 Hz), 131.08 (d, *J* = 10.4 Hz), 130.39 (d, *J* = 2.8 Hz), 129.03 (d, *J* = 82.0 Hz), 127.65 (d, *J* = 12.6 Hz), 123.23 (t, *J* = 3.7 Hz), 48.30 (d, *J* = 46.8 Hz), 29.79, 24.34 (d, *J* = 5.2 Hz), 20.98.

^{31}P NMR (162 MHz, $CDCl_3$) δ 41.74.

HRMS (ESI, m/Z): calcd. for $C_{37}H_{40}NP_2S_2^+$ [M+H]$^+$: 624.2072, found: 624.2061.

Melting point: 94.2 ^{o}C

Specific rotation: $[\alpha]_D^{20}$= +68.09 (c = 4.168 g/100mL, $CHCl_3$).

SFC Chiralcel OD, CO_2/MeOH with gradient from 97% to 50% CO_2 in 8 min, 1.0 mL/min., 40 °C, detection at 210 nm. Retention time: 3.67 min. (minor) and 3.87 min. (major). 99% *ee*, 9:1 *dr*.

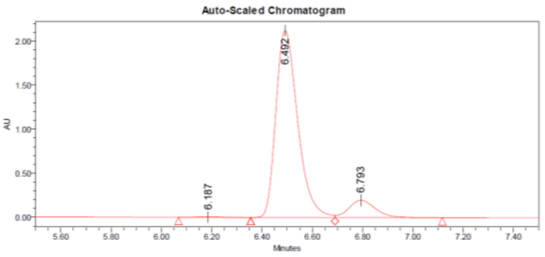

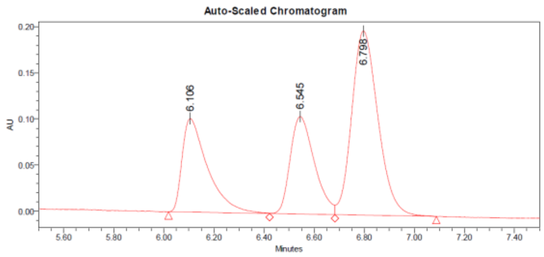

Peak Results

	Name	RT	Area	% Area	Height
1		6.187	29519	0.21	4157
2		6.492	12755971	89.84	2120485
3		6.793	1412747	9.95	194567

Peak Results

	Name	RT	Area	% Area	Height
1		6.106	714268	24.87	101426
2		6.545	707246	24.63	105701
3		6.798	1450398	50.50	199428

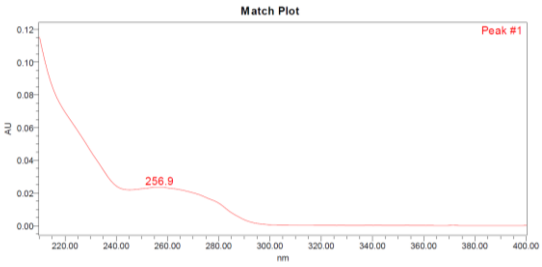

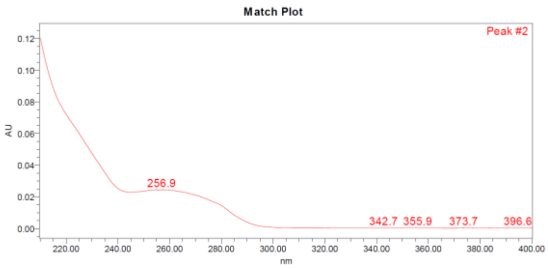

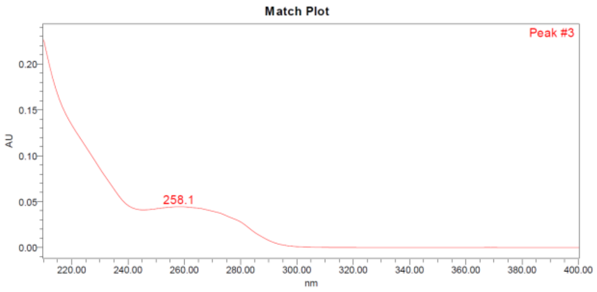

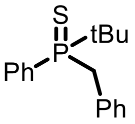

benzyl(tert-butyl)(phenyl)phosphine sulfide

This compound was synthesized following the condition with HPhtBu (0.1 mmol, 1.0equiv.), benzyl chloride (0.3 mmol, 3.0 equiv.), (R_C, S_P)-Clarke catalyst (0.008 mol, 0.08 equiv.), Cs_2CO_3 (0.3 mmol, 3 equiv.), in dry, deoxygenated acetonitrile for 16 h. The reaction mixture was filtered through a plug of silica in order to remove the base and catalyst. Diethyl ether was

added to flush the plug. After protecting with S_8, the crude was purified with chromatography and gave the desired product in 78% yield.

^{1}H NMR (401 MHz, cdcl$_3$) δ 7.97 (ddd, *J* = 11.5, 7.8, 1.7 Hz, 2H), 7.53 – 7.40 (m, 3H), 7.30 – 7.24 (m, 2H), 7.22 – 7.10 (m, 3H), 3.85 (dd, *J* = 14.1, 10.4 Hz, 1H), 3.48 (t, *J* = 13.7 Hz, 1H), 1.23 (d, *J* = 16.1 Hz, 9H).

^{13}C NMR (101 MHz, cdcl$_3$) δ 133.25 (d, *J* = 8.5 Hz), 131.77 (d, *J* = 7.7 Hz), 131.48 (d, *J* = 2.9 Hz), 130.70 (d, *J* = 5.0 Hz), 128.20 (d, *J* = 69.7 Hz), 128.11 (d, *J* = 2.7 Hz), 128.03 (d, *J* = 11.2 Hz), 127.08 (d, *J* = 3.2 Hz), 35.55 (d, *J* = 48.8 Hz), 32.84 (d, *J* = 45.1 Hz), 25.38 (d, *J* = 1.7 Hz).

^{31}P NMR (162 MHz, cdcl$_3$) δ 63.09.

HRMS (ESI, m/Z): calcd. for $C_{17}H_{22}PS^{+}$ $[M+H]^{+}$: 289.1174, found: 289.1172.

SFC Chiralcel OJ-3, CO_2/MeOH with gradient from 97% to 50% CO_2 in 4.5 min, 1.8 mL/min., 40 ^{o}C, detection at 220 nm. Retention time: 2.35min and 2.53 min. 6% *ee.*

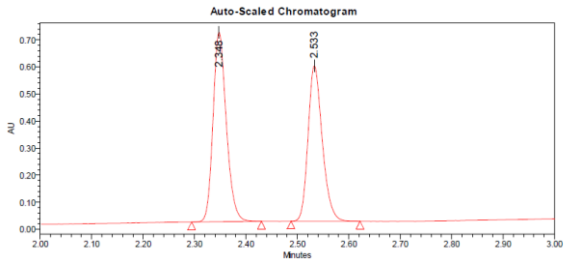

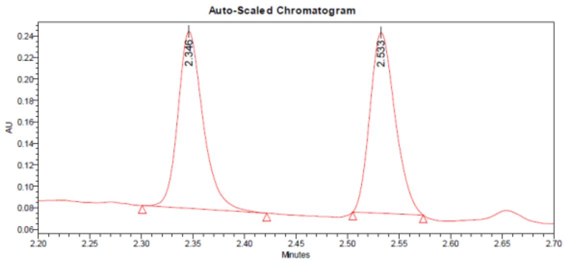

Peak Results

	Name	RT	Area	% Area	Height
1		2.348	1255191	53.24	699797
2		2.533	1102403	46.76	574068

Peak Results

	Name	RT	Area	% Area	Height
1		2.346	278878	49.38	164800
2		2.533	285902	50.62	168372

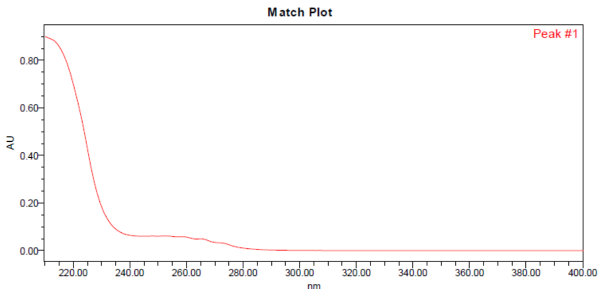

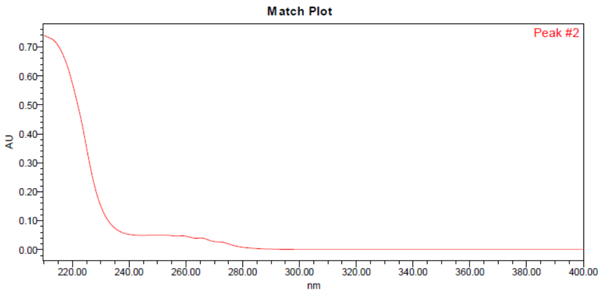

8. Application of the methodology: Synthesis and characterization of copper complex and manganese complex

8.1.1 Synthesis of **L-Cu(I)** complex

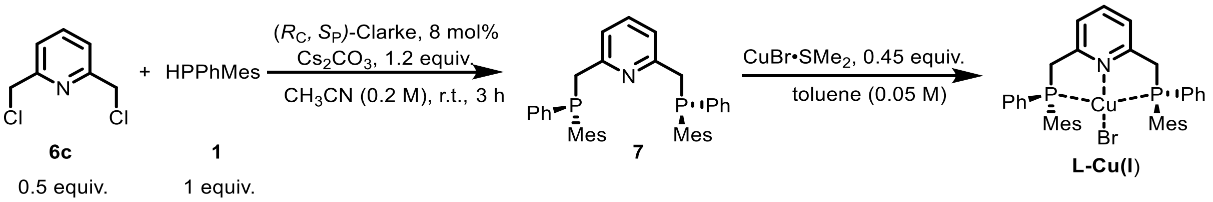

A 4 ml vial equipped with a magnetic stirring bar was charged with (R_C, S_P)-Clarke catalyst (23.2 mg, 0.032 mmol, 0.08 equiv.) and Cs_2CO_3 (156 mg, 0.48 mmol, 1.2 equiv.). Then the vial was transferred into the glovebox. HPPhMes (0.4 mmol, 1.0 equiv.), 2,6-bis(chloromethyl)pyridine (0.2 mmol, 0.5 equiv.) and anhydrous CH_3CN (2 mL) were added into the vial. The resulting mixture was stirred at room temperature for 3 hours. Then the reaction mixture was subjected to a flush column chromatography to remove the base and the catalyst. After removing the solvent under reduced pressure, CuBr•SMe_2 (37 mg, 0.18 mmol, 0.45 mmol) and toluene (0.05 M) were added into 20 ml vial with the residue. The mixture was stirred for 12 h under room temperature. Then the solvent was removed under reduced pressure and the resulting solid was dissolved with minimum DCM giving a clear solution after filtration. After adding pentane, a yellow precipitate formed which was washed with pentane for 5 times.

8.1.2 Characterization of the **L-Cu(I)** complex

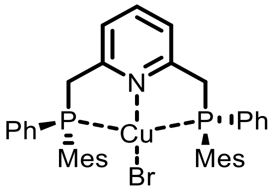

^{1}H NMR (400 MHz, $CDCl_3$) δ 7.61 (t, J = 7.9 Hz, 1H), 7.34 – 6.97 (m, 12H), 6.75 (s, 4H), 4.11 (s, 4H), 2.51 (s, 12H), 2.24 (s, 6H).

^{13}C NMR (101 MHz, $CDCl_3$) δ 155.69, 143.91, 140.11, 138.38, 134.83, 130.89, 130.83, 130.76, 130.65, 130.62, 130.59, 128.62, 128.57, 128.55, 123.74, 121.75, 41.23, 24.47, 24.39, 24.31, 21.07.

^{31}P NMR (162 MHz, $CDCl_3$) δ -12.92.

HRMS (ESI, m/Z): calcd. for $C_{37}H_{40}BrCuNP_2^+$ $[M+H]^+$: 702.1110, found: 702.1068; $[M-Br]^+$: 622.1854, found: 622.1826.

Melting point: 181.8 °C

The copper complex (around 15 mg) was dissolved in 4 mL vial by dry and degassed THF (in the glovebox). The 4 mL vial was placed inside of a 20 mL vial filled with pentane (around 10 mL). The single crystal was grown by slow solvent diffusion at room temperature. CCDC 2357465 contains the supplementary crystallographic data of the copper complex.

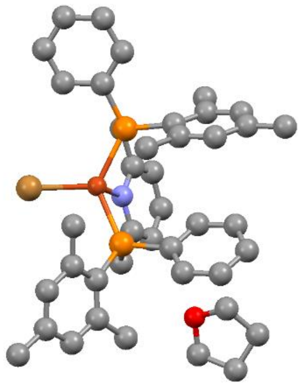

Table 3. Crystal data and structure refinement for CCDC 2357465

chem. formula	C41H47BrCuNOP2
Mr	775.18
cryst syst.	orthorhombic
color, habit	gold, block
size (mm)	0.590 x 0.333 x 0.197
space group	P 21 21 21
a (Å)	11.2851(9)
b (Å)	14.8215(12)
c (Å)	22.0662(16)
α, deg	90
β, deg	90
γ, deg	90

V (Å3)	3690.8(5)
Z	4
ρcalc, g.cm-3	1.395
μ(Cu Kα), cm-1	1.794
F(000)	1608
temp (K)	100(2)
θ range (deg)	2.449 - 29.816
data collected (h,k,l)	-15:15, -20:20, -30:30
no. of rflns collected	67832
no. of indpndt reflns	10510
observed reflns (Fo ≥ 2 σ(Fo))	9306
R(F) (%)	3.07
wR(F2) (%)	6.6
GooF	1.017
Weighting a,b	0.0210, 1.3211
params refined	431
restraints	0
min, max resid dens	-0.602, 0.717
Flack x	0.038(7)

Note: Large reflection outliers with (Iobs-Icalc)/Sigma(W) >10 were omitted from the refinement.

8.2.1 Synthesis of **L-Mn(I)** complex

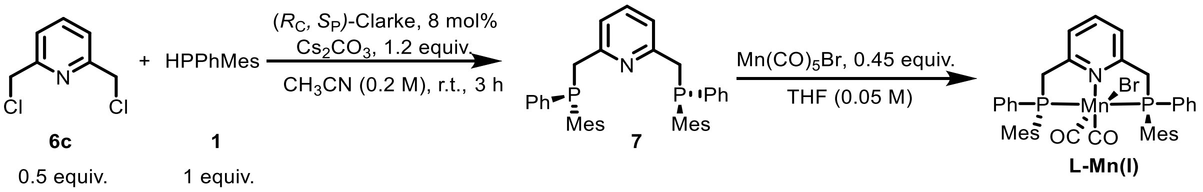

A 4 ml vial equipped with a magnetic stirring bar was charged with (R_C, S_P)-Clarke catalyst (23.2 mg, 0.032 mmol, 0.08 equiv.) and Cs_2CO_3 (156 mg, 0.48 mmol, 1.2 equiv.). The vial was transferred to the glovebox. HPPhMes (0.4 mmol, 1.0 equiv.), 2,6-bis(chloromethyl)pyridine (0.2 mmol, 0.5 equiv.) and anhydrous CH_3CN (2 mL) were added into the vial. The resulting mixture was stirred at room temperature for 3 hours. Then the reaction mixture was subjected

to a flush column chromatography to remove the base and the catalyst. After removing the solvent under reduced pressure, $Mn(CO)_5Br$ (50 mg, 0.18 mmol, 0.45 eq.) and THF (0.05 M) were added into 20 ml vial with the residue. The mixture was stirred for 12 h under room temperature. The solvent was removed under reduced pressure and the resulting solid was dissolved with minimum amount of DCM giving a clear solution after filtration. After adding pentane, a yellow precipitate formed, which was washed with pentane for 5 times. The resulting solid was dried to give the Mn-complex.

8.2.2 Characterization of the **L-Mn(I)** complex

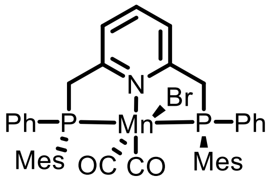

^{31}P NMR (162 MHz, cdcl$_3$) δ 85.47 (d, *J* = 108.1 Hz), 80.15 (d, *J* = 106.8 Hz).

HRMS (ESI, m/Z): calcd. For $C_{39}H_{40}BrMnNO_2P_2{}^+$ [M+H]$^+$: 750.1093, found: 750.0913; [M-Br]$^+$: 670.1837, found: 670.1835; [M-Br-2CO]$^+$: 614.1938, found: 614.1936.

Melting point: When the temperature increased to around 180 °C, the compound just decomposed. The melting point cannot be measured.

The **L-Mn(I)** complex (around 15 mg) was dissolved in 4 mL vial by dry and degassed THF (in the glovebox). The 4 mL vial was placed inside of a 20 mL vial filled with pentane (around 10 mL). The single crystal was grown by slow solvent diffusion at room temperature. CCDC 2358166 contains the supplementary crystallographic data of the **L-Mn(I)** complex.

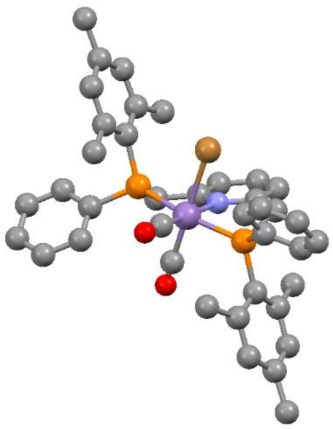

(The major disorder component is depicted)

Table 4. Crystal data and structure refinement for CCDC 2358166

chem. formula	C39H39BrMnNO2P2
Mr	750.50
cryst syst.	orthorhombic
color, habit	gold, needle
space group	P 21 21 21
a (Å)	10.6787(3)
b (Å)	11.0328(2)
c (Å)	33.3811(8)
α, deg	90
β, deg	90
γ, deg	90
V (Å3)	3932.83(16)
Z	4
ρcalc, g.cm-3	1.268
μ(Cu Kα), cm-1	4.930
F(000)	1544
temp (K)	100(2)
θ range (deg)	2.647 - 74.649
data collected (h,k,l)	-13:13, -13:11, -41:41
no. of rflns collected	52163
no. of indpndt reflns	8019
observed reflns (Fo ⩾ 2 σ(Fo))	7218
R(F) (%)	3.75
wR(F2) (%)	9.04
GooF	1.029
Weighting a,b	
params refined	450

restraints	17
min, max resid dens	-0.507, 0.511
Flack x	0.049(7)

Note:

In the last step of the refinement for the model of the **L-Mn(I)**, no realistic displacement parameter was found for the CO ligand located trans to the bromide ligand. This issue was interpreted as substitutional disorder in which the positions of the bromide ligand (Br1A) and the CO ligand (C1A, O1A) located trans to the bromide (Br1A) are switched for the major and minor disorder components.

The disorder was expressed through a 2 step occupancy model for the carbonyl ligand, the bromide ligand and the manganese atom (C1A, C1B, O1A, O1B, Br1A, Br1B, Mn1A, Mn1B).

When trying to find the second disorder component of the CO ligand, the oxygen atom O1B was found to be *Non Positive Definite*. Therefore, constraints were introduced using the SAME and ISOR instruction to construct the minor CO (C1B, O1B) component with a chemically realistic bond angle and bond lengths.

Electron density associated with a disordered THF solvent molecule was removed using Platon Squeeze.

The major disorder component is depicted

8.2.3 Application of the **L-Mn(I)** complex in catalysis

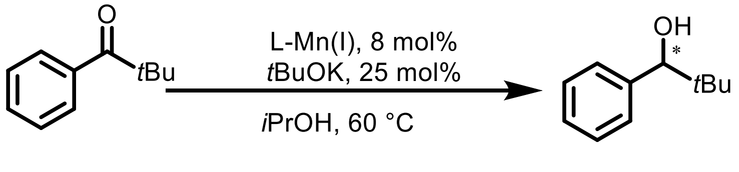

A 20 mL vial equipped with a magnetic stirring bar was charged with the **L-Mn(I)** (6 mg,8 mol%, 0.008 mmol), and *t*BuOK (2.5 mg, 25 mol%, 0.025 mmol). After transferring the tube into the glove box, THF was added. Then *i*PrOH (0.5 mL) and ketone (0.1 mmol) were added after 5 minutes's stirring in the glove box. Next, the vial was sealed with cap and heated up to 60 °C for 48 h. The resulting mixture was purified via silica gel column chromatography (pentane/diethyl ether = 50/1-5/1) to give the desired product.

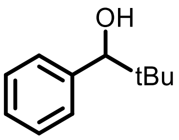

10 mg, 61% yield, white solid

^{1}H NMR (400 MHz, $CDCl_3$) δ 7.52 – 7.07 (m, 5H), 4.40 (d, *J* = 2.6 Hz, 1H), 1.85 (d, *J* = 2.8 Hz, 1H), 0.93 (s, 9H).

^{13}C NMR (101 MHz, $CDCl_3$) δ 142.18, 127.60, 127.55, 127.27, 82.40, 35.62, 25.91.

The NMR data are in agreement with literature precedents.[4]

HPLC CHIRALPAK OD-H, heptane/*i*PrOH = 95/5, flow rate: 0.5 mL/min, 40 ^{o}C, detection at 221 nm. Retention time: 12.51 min. (major) and 17.21 min. (minor). 98% *ee*.

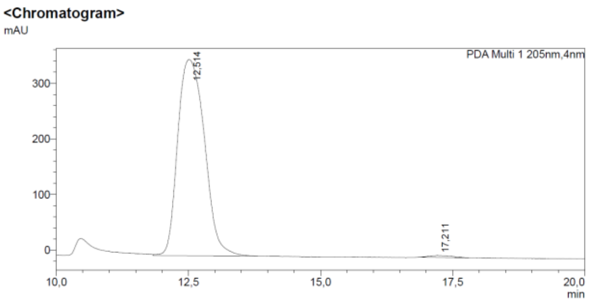

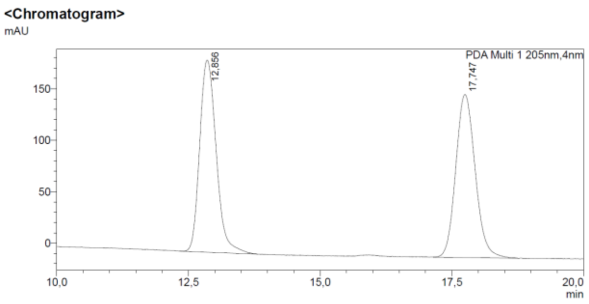

<Peak Table>

PDA Ch1 205nm

Peak#	Ret. Time	Area	Height	Area%
1	12,514	12468564	352858	99,185
2	17,211	102416	3428	0,815
Total		12570981	356286	100,000

<Peak Table>

PDA Ch1 205nm

Peak#	Ret. Time	Area	Height	Area%
1	12,856	4068628	186628	50,077
2	17,747	4056105	158470	49,923
Total		8124733	345098	100,000

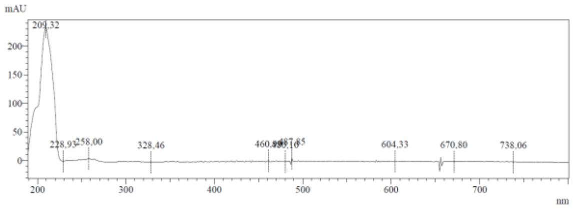

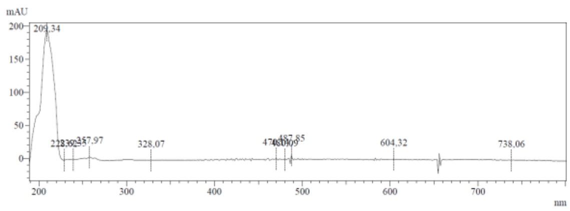

9. References

[1] W. Kohn, L. J. Sham. Phys. Rev. A 1965, 140, 1133–1138.

[2] (a) A. D. Becke. J. Chem. Phys. 1993, 98, 5648−5652. (b) A. D. Becke. J. Chem. Phys. 1993, 98, 1372−1377. (c) C. Lee, W. Yang, R. G. Parr. Phys. Rev. B: Condens. Matter Mater. Phys. 1988, 37, 785−789. (d) F. Weigend, R. Ahlrichs. Phys. Chem. Chem. Phys. 2005, 7, 3297−3305.

[3] J. Tomasi, B. Mennucci, R. Cammi. Chem. Rev. 2005, 105, 2999–3093.

[4] Gaussian 16, Revision C.01, M. J. Frisch, G. W. Trucks, H. B. Schlegel, G. E. Scuseria, M. A. Robb, J. R. Cheeseman, G. Scalmani, V. Barone, G. A. Petersson, H. Nakatsuji, X. Li, M. Caricato, A. V. Marenich, J. Bloino, B. G. Janesko, R. Gomperts, B. Mennucci, H. P. Hratchian, J. V. Ortiz, A. F. Izmaylov, J. L. Sonnenberg, D. Williams-Young, F. Ding, F. Lipparini, F. Egidi, J. Goings, B. Peng, A. Petrone, T. Henderson, D. Ranasinghe, V. G. Zakrzewski, J. Gao, N. Rega, G. Zheng, W. Liang, M. Hada, M. Ehara, K. Toyota, R. Fukuda, J. Hasegawa, M. Ishida, T. Nakajima, Y. Honda, O. Kitao, H. Nakai, T. Vreven, K. Throssell, J. A. Montgomery, Jr., J. E. Peralta, F. Ogliaro, M. J. Bearpark, J. J. Heyd, E. N. Brothers, K. N. Kudin, V. N. Staroverov, T. A. Keith, R. Kobayashi, J. Normand, K. Raghavachari, A. P. Rendell, J. C. Burant, S. S. Iyengar, J. Tomasi, M. Cossi, J. M. Millam, M. Klene, C. Adamo, R. Cammi, J. W. Ochterski, R. L. Martin, K. Morokuma, O. Farkas, J. B. Foresman, and D. J. Fox, Gaussian, Inc., Wallingford CT, 2016.

[5] (a) R. Bauernschmitt, R. Ahlrichs. J. Chem. Phys. 1996, 104, 9047–9052. (b) H. B. Schlegel, J. J. W. McDouall, Do you have SCF stability and convergence problems? in Computational Advances in Organic Chemistry: Molecular Structure and Reactivity 167–185 (Springer Netherlands, 1991). doi:10.1007/978-94-011-3262-6_2. (c) R. Seeger, J. A. Pople. J. Chem. Phys. 1977, 66, 3045–3050.

[6] (a) K. Fukui. Acc. Chem. Res. 1981, 14, 363–368. (b) S. Maeda, Y. Harabuchi, Y. Ono, T. Taketsugu, K. Morokuma. Int. J. Quantum Chem. 2015, 115, 258–269.

[7] Huang, Y.; Li, Y.; Leung, P.-H.; Hayashi, T. *J. Am. Chem. Soc*. **2014**, 136, 4865-4868.

[8] Zhang S., Xiao J.-Z., Li Y.-B., Shi C.-Y., Yin L. *J. Am. Chem. Soc.* **2021**, *143*(26), 9912–9921

[9] Tayama, Eiji, Goshu Shimizu, and Ryo Nakao. *Tetrahedron* **2022**, 111, 132721.

10. NMR spectra

¹H NMR (400 MHz, $CDCl_3$) spectrum of **2m**

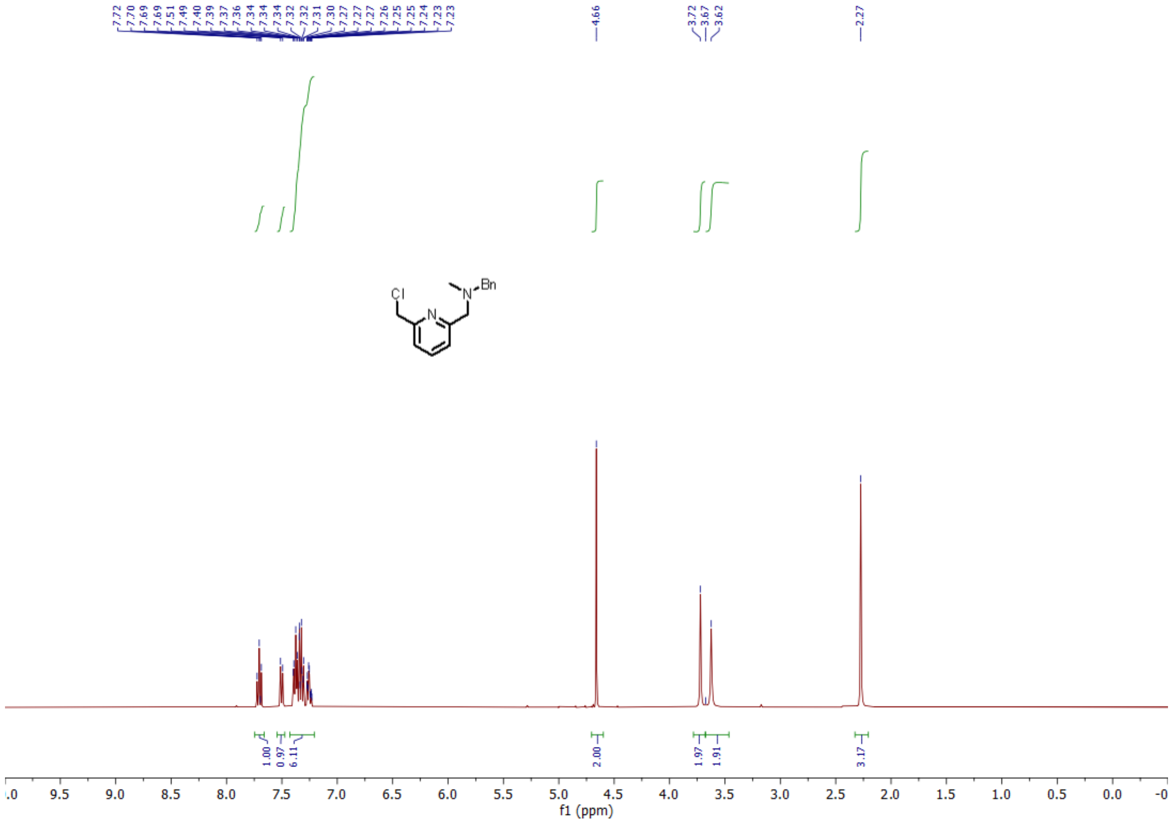

¹³C NMR (101 MHz, $CDCl_3$)spectrum of **2m**

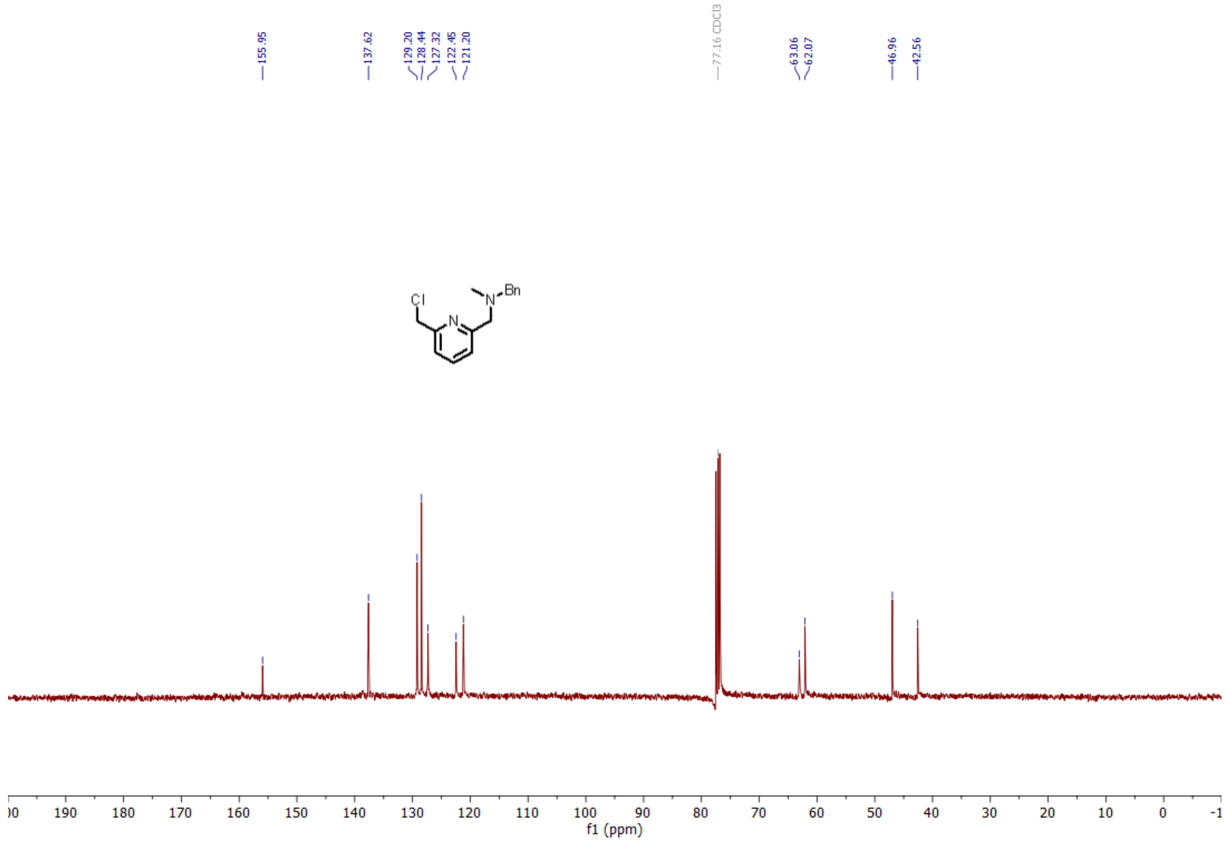

^{1}H NMR (400 MHz, $CDCl_3$) spectrum of **2’a**

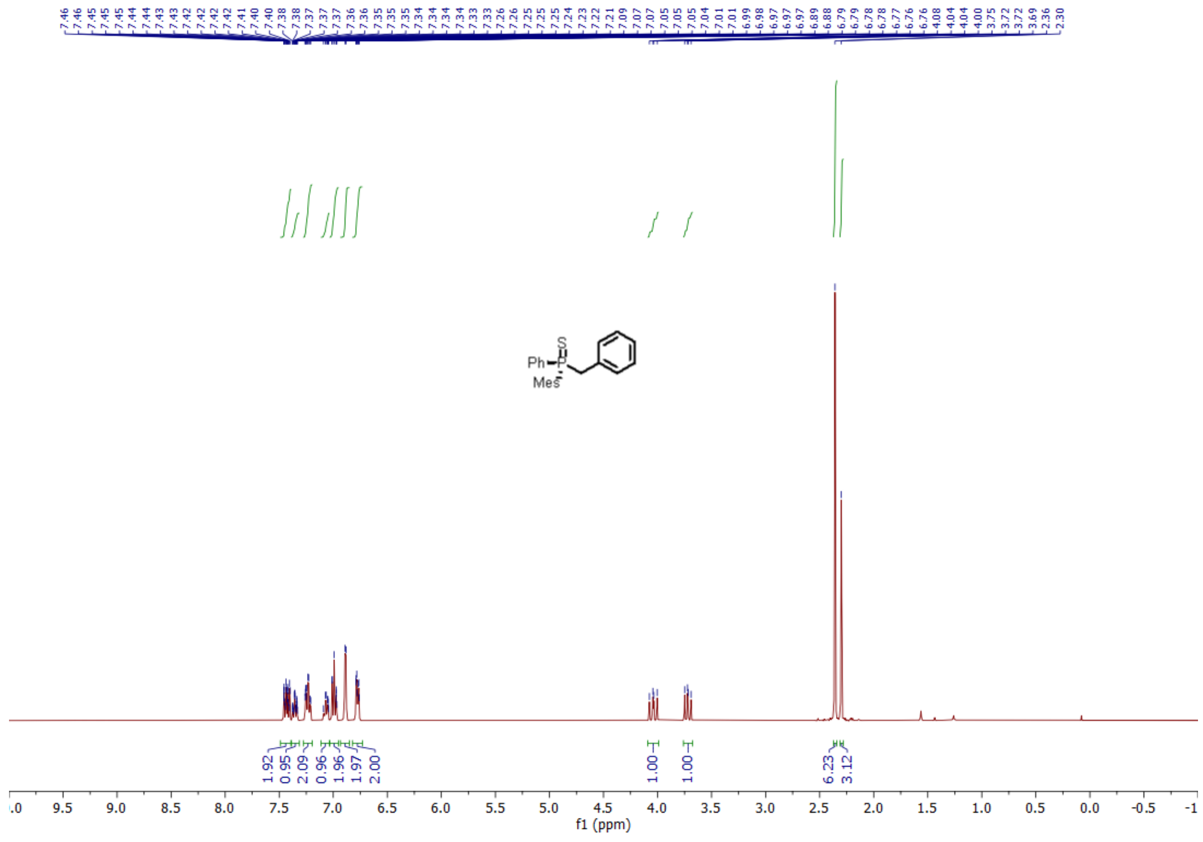

^{13}C NMR (101 MHz, $CDCl_3$)spectrum of **2’a**

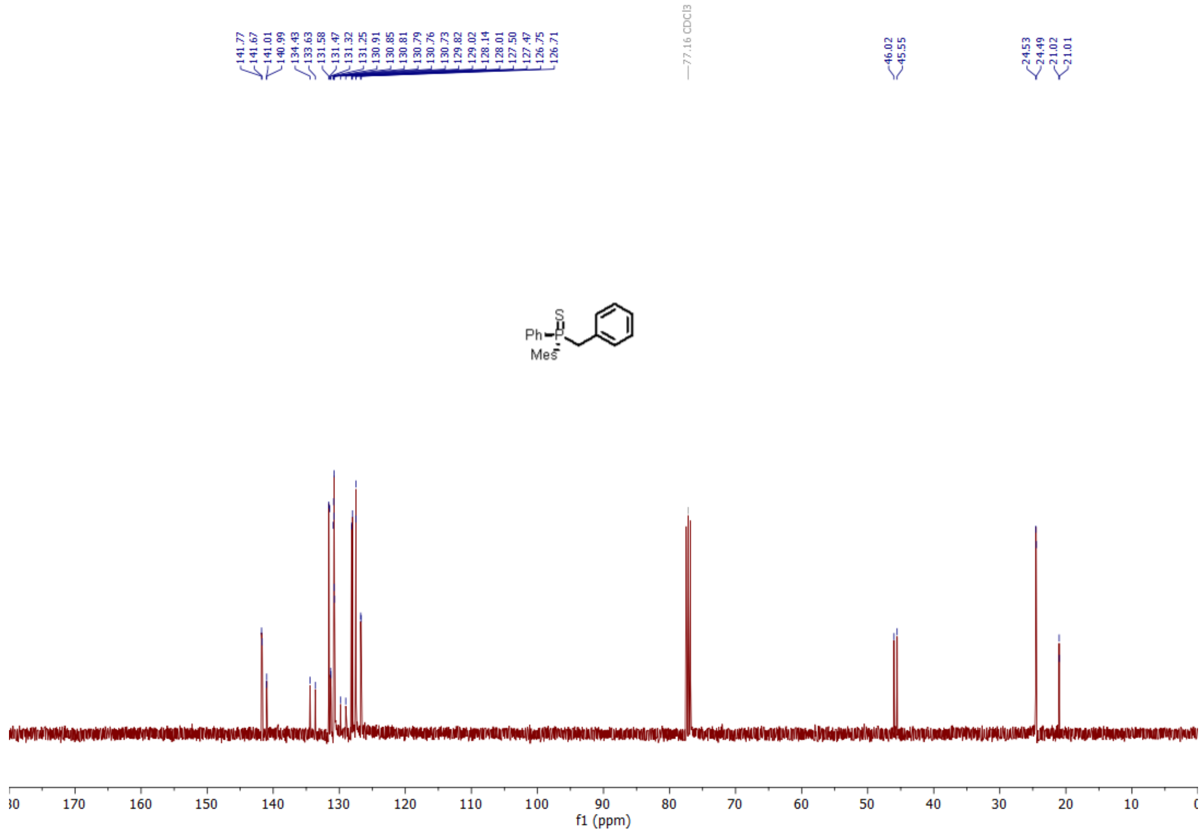

^{31}P NMR (162 MHz, $CDCl_3$) spectrum of **2’a**

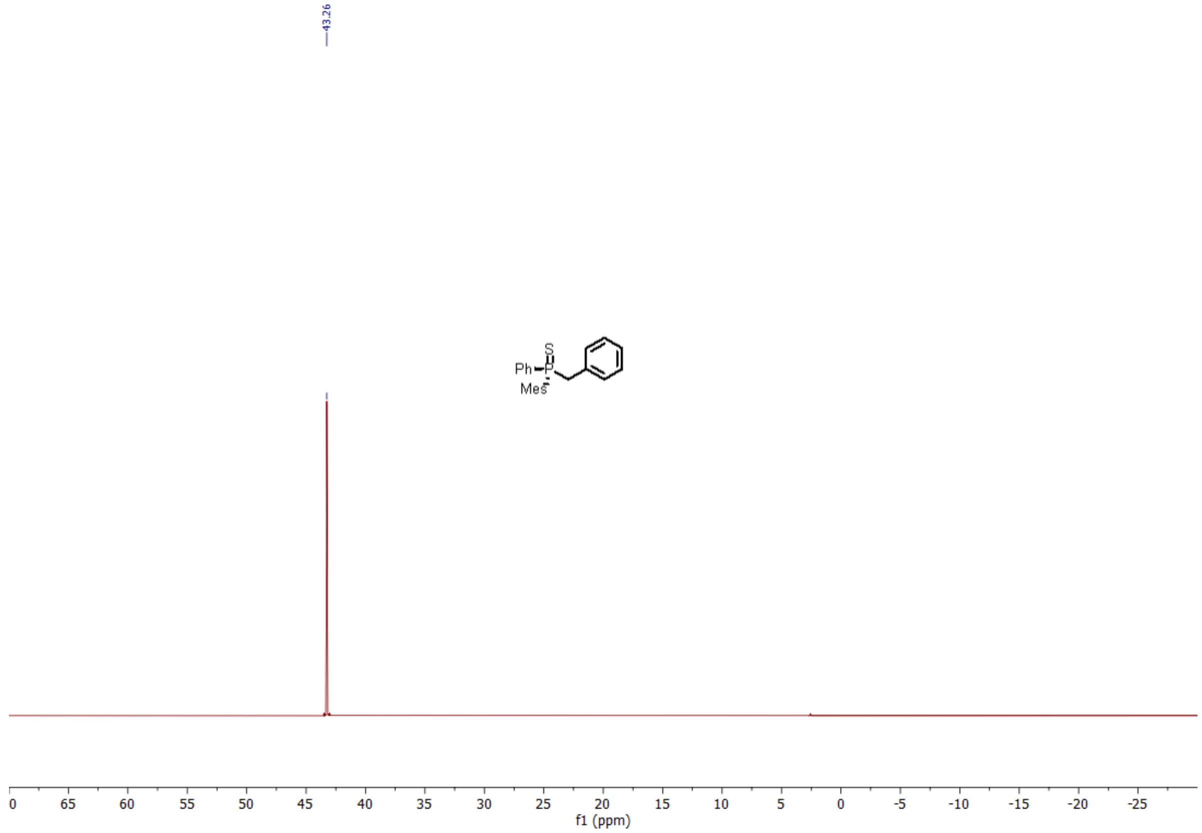

^{1}**H NMR** (400 MHz, $CDCl_3$) spectrum of **2'b**

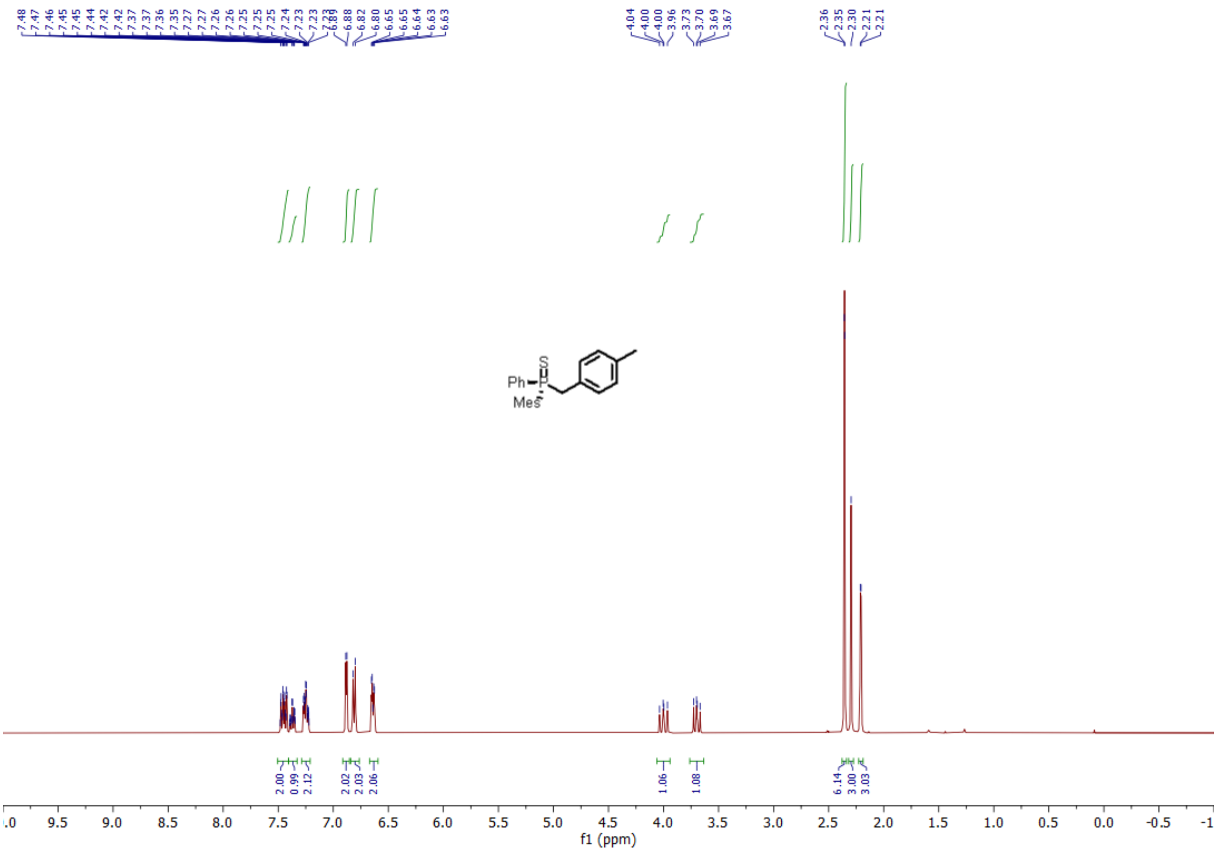

^{13}**C NMR** (101 MHz, $CDCl_3$)spectrum of **2'b**

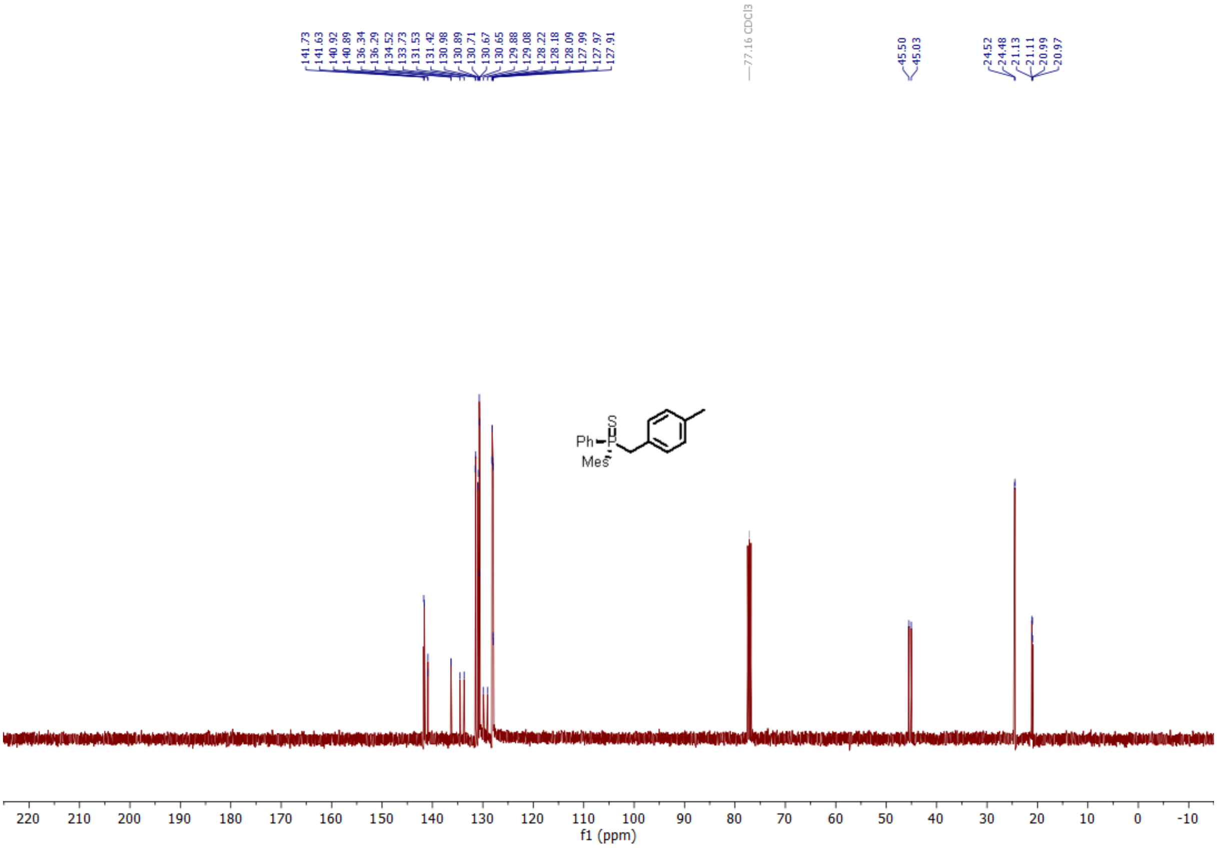

^{31}**P NMR** (162 MHz, $CDCl_3$) spectrum of **2’b**

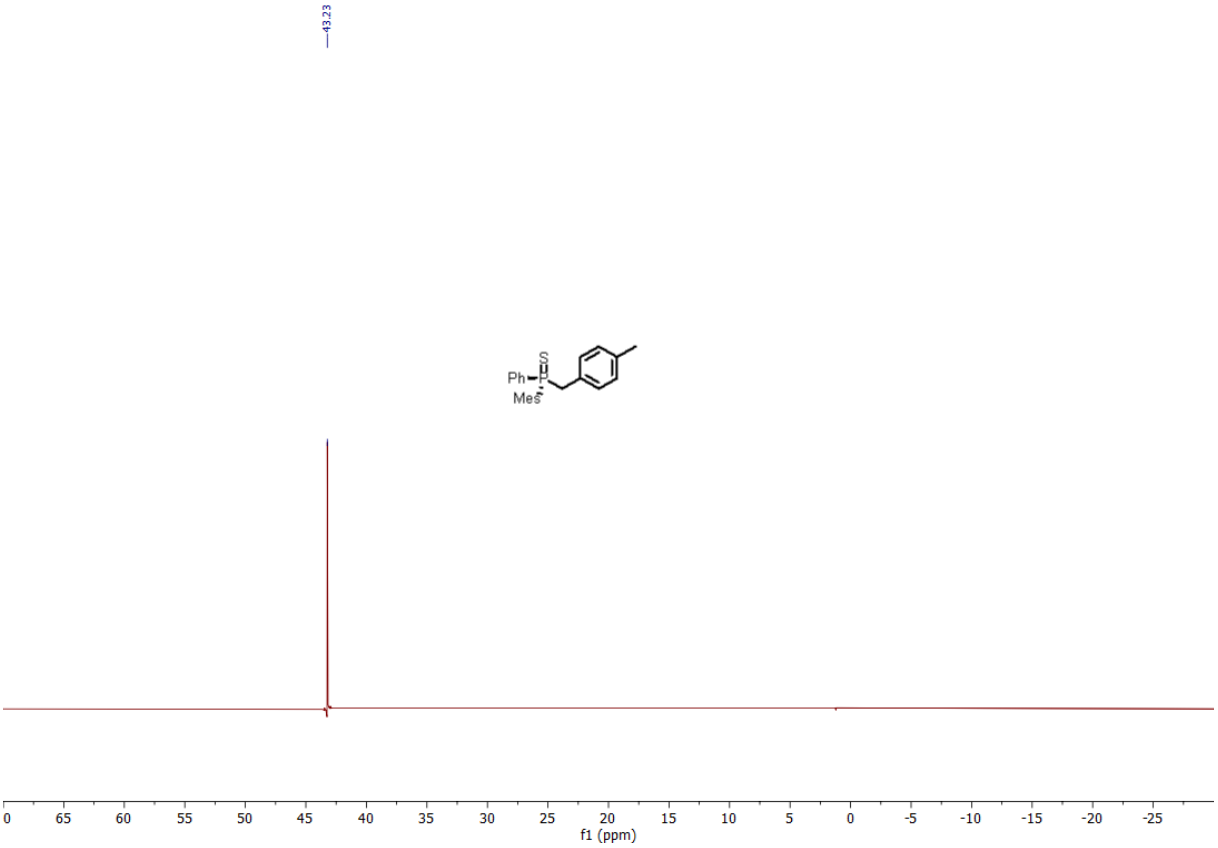

^{1}**H NMR** (400 MHz, $CDCl_3$) spectrum of **2’c**

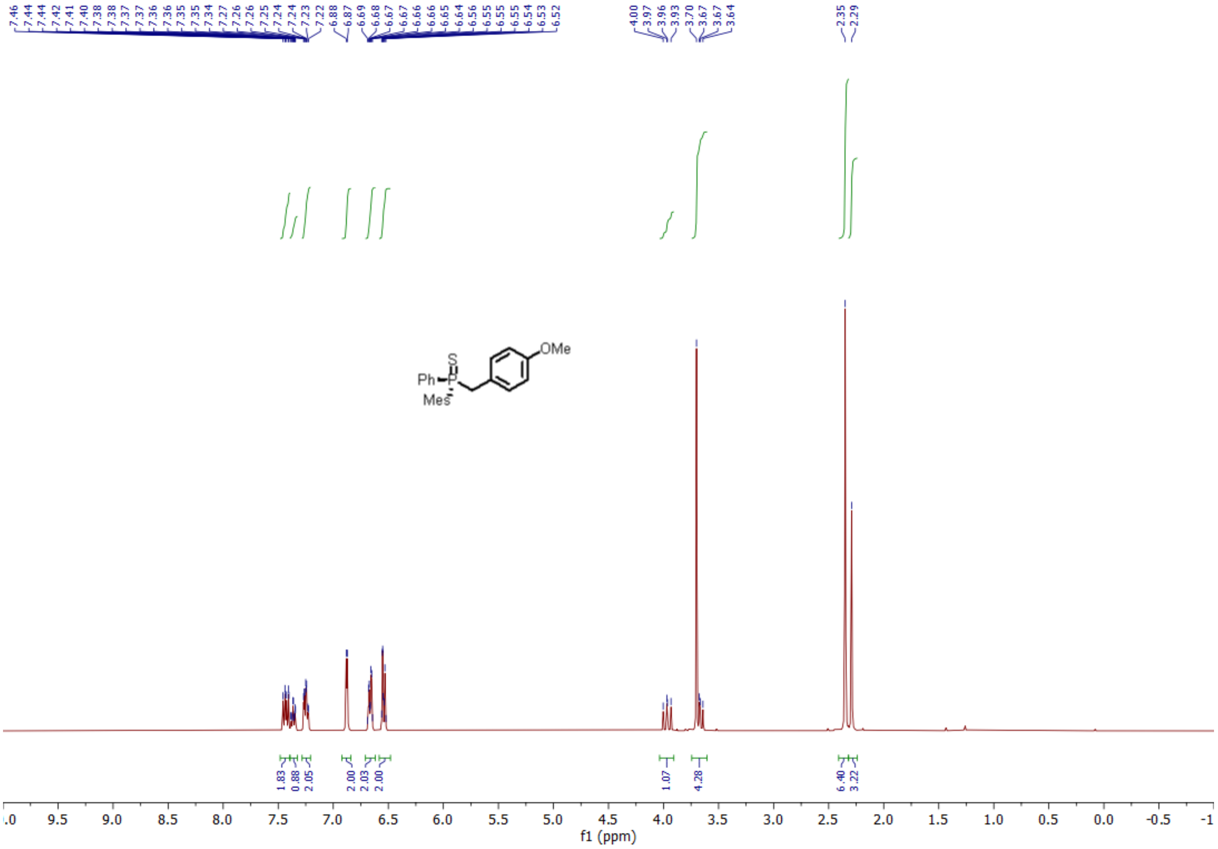

^{13}C NMR (101 MHz, $CDCl_3$)spectrum of **2’c**

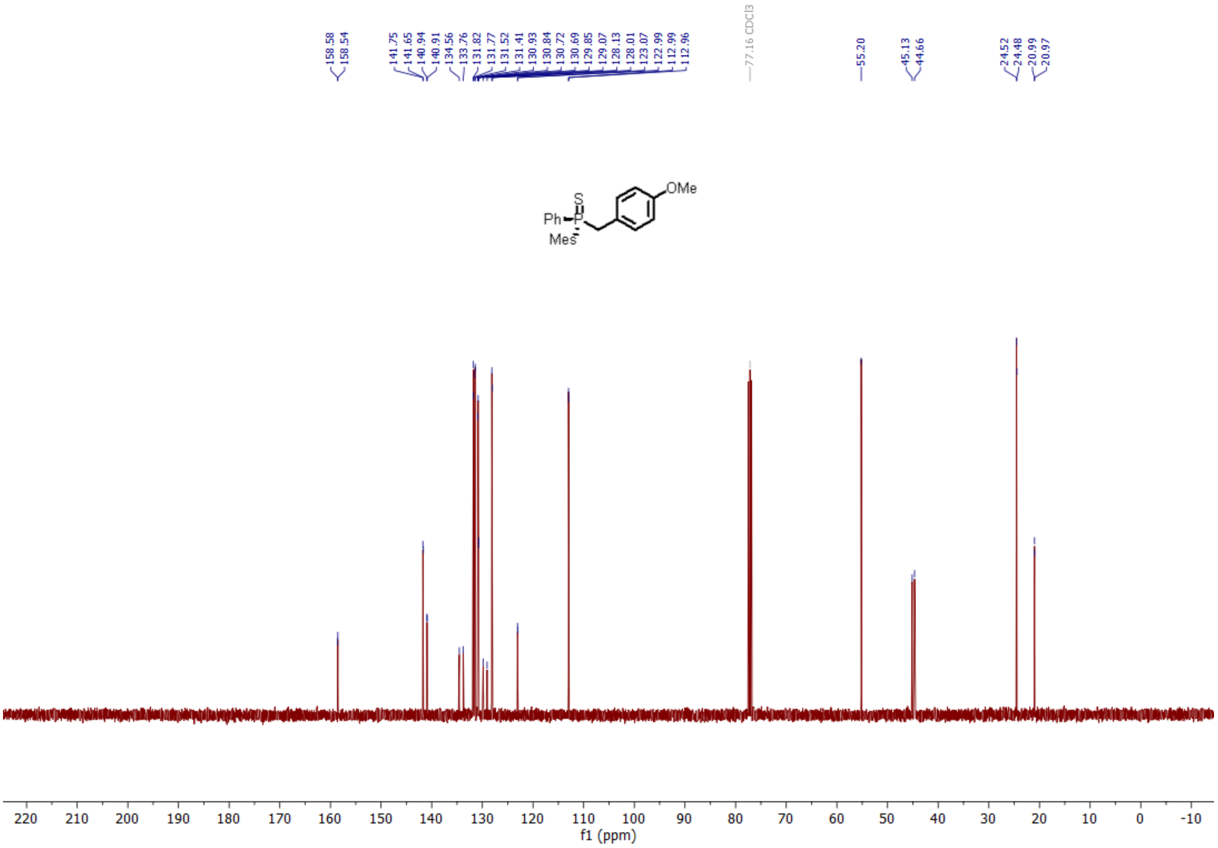

^{31}P NMR (162 MHz, $CDCl_3$) spectrum of **2’c**

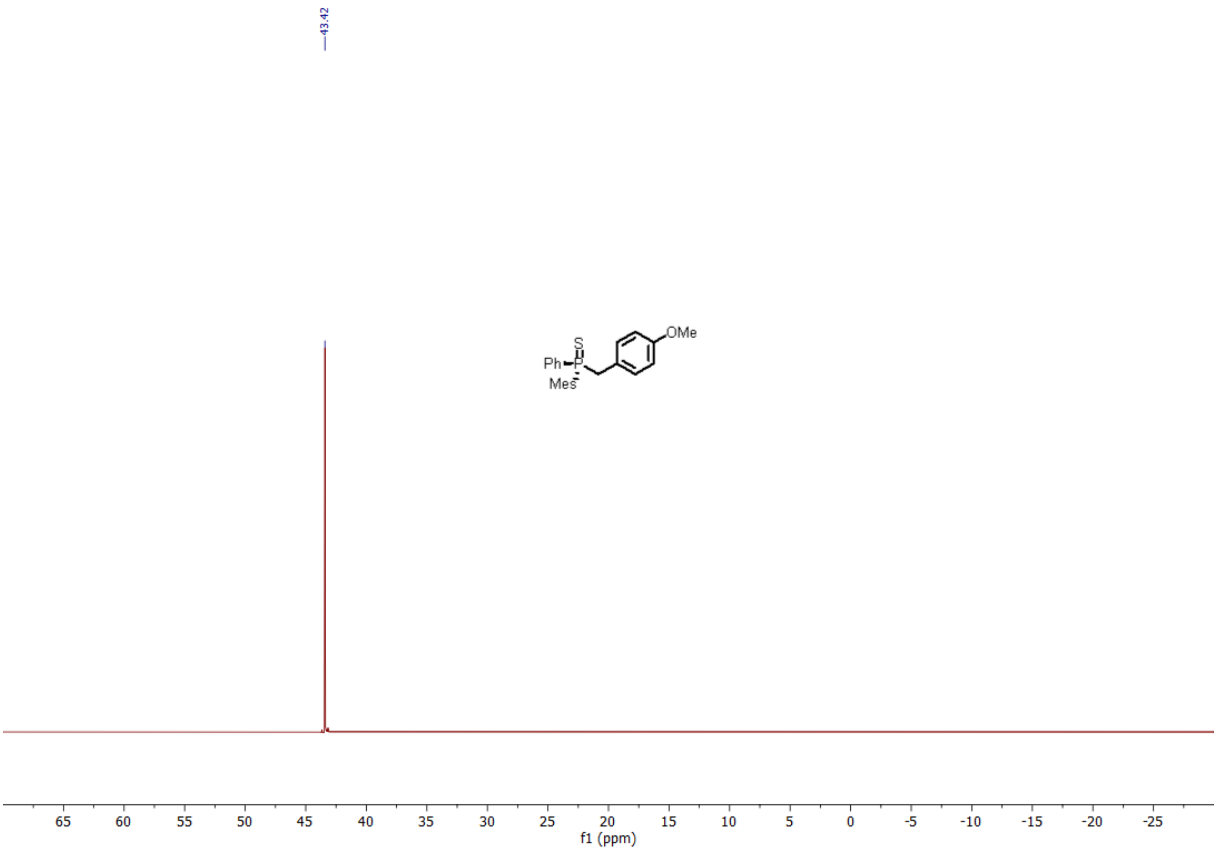

^{1}H NMR (400 MHz, $CDCl_3$) spectrum of **2’d**

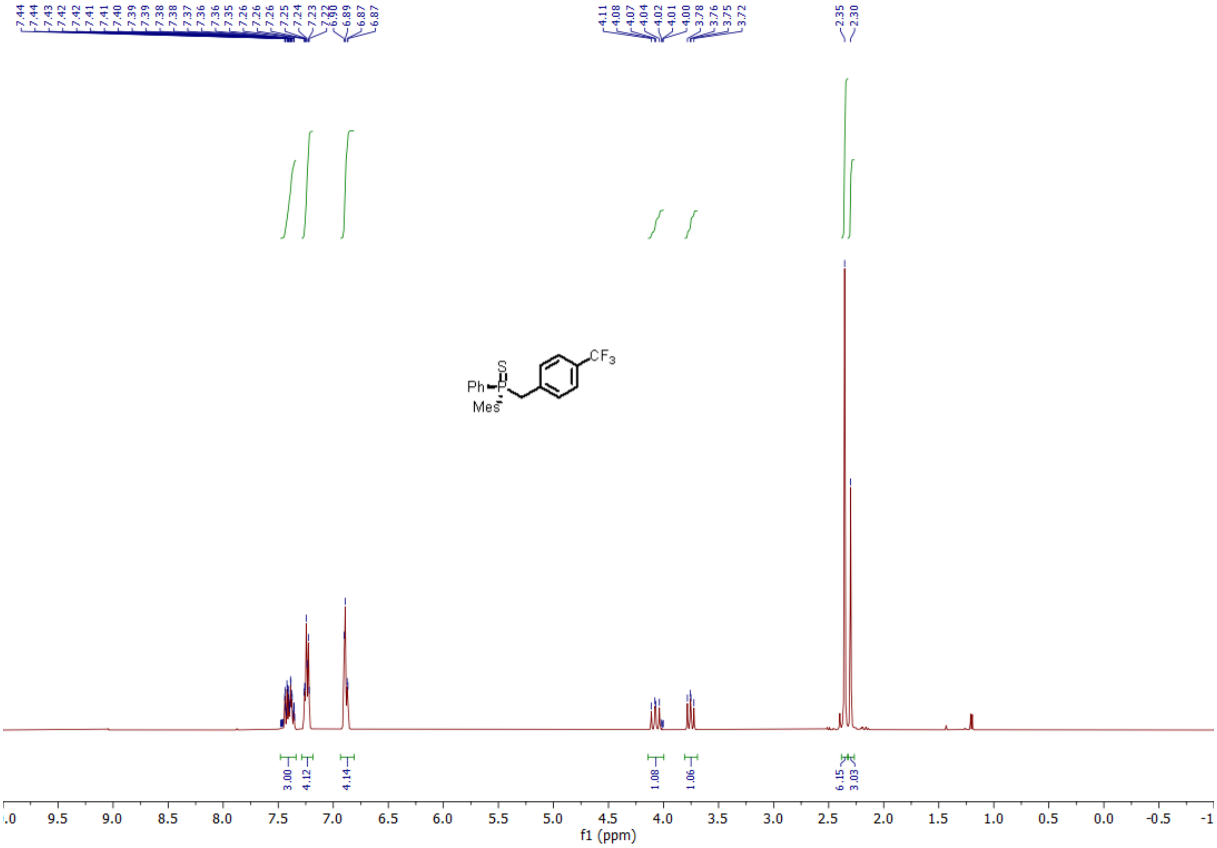

^{13}C NMR (101 MHz, $CDCl_3$)spectrum of **2’d**

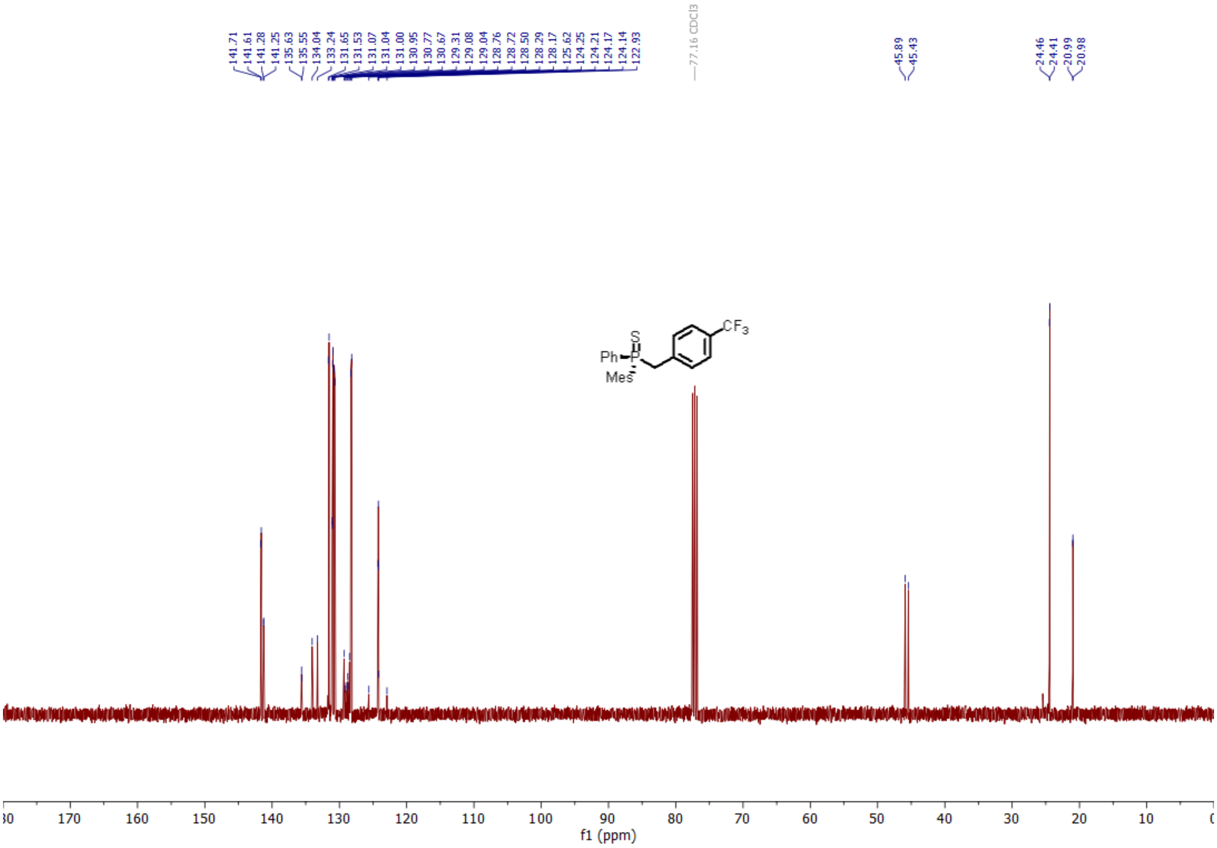

^{31}P NMR (162 MHz, $CDCl_3$) spectrum of **2’d**

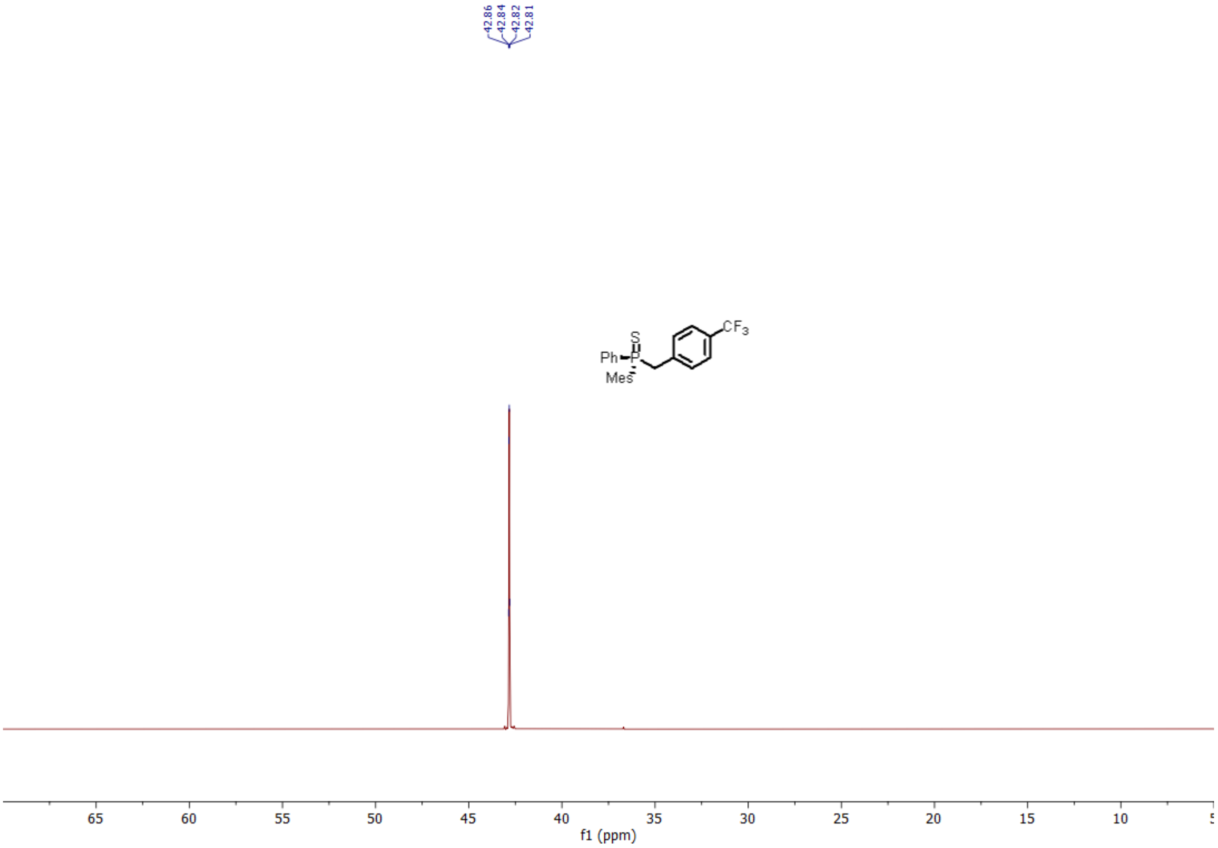

^{19}F NMR (376 MHz, $CDCl_3$) spectrum of **2’d**

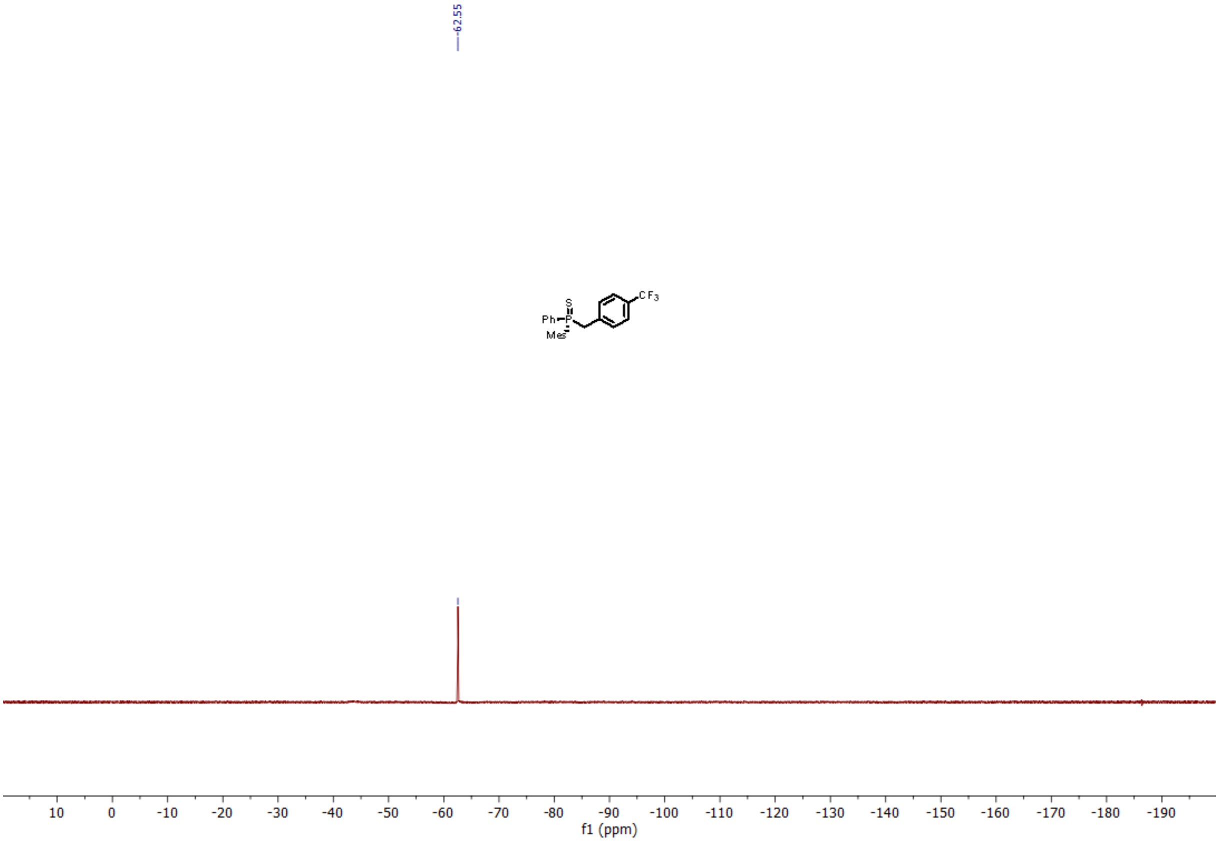

^{1}H NMR (400 MHz, $CDCl_3$) spectrum of **2’e**

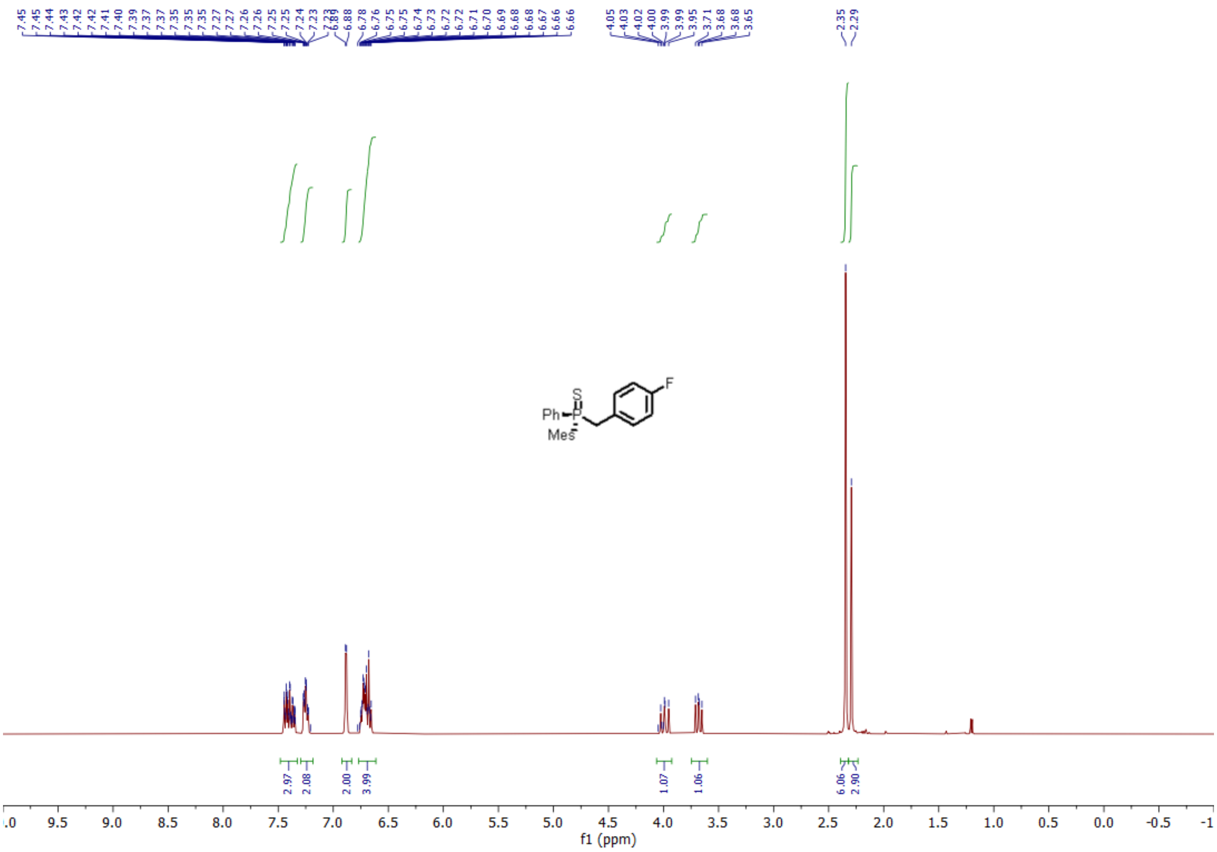

^{13}C NMR (101 MHz, $CDCl_3$)spectrum of **2’e**

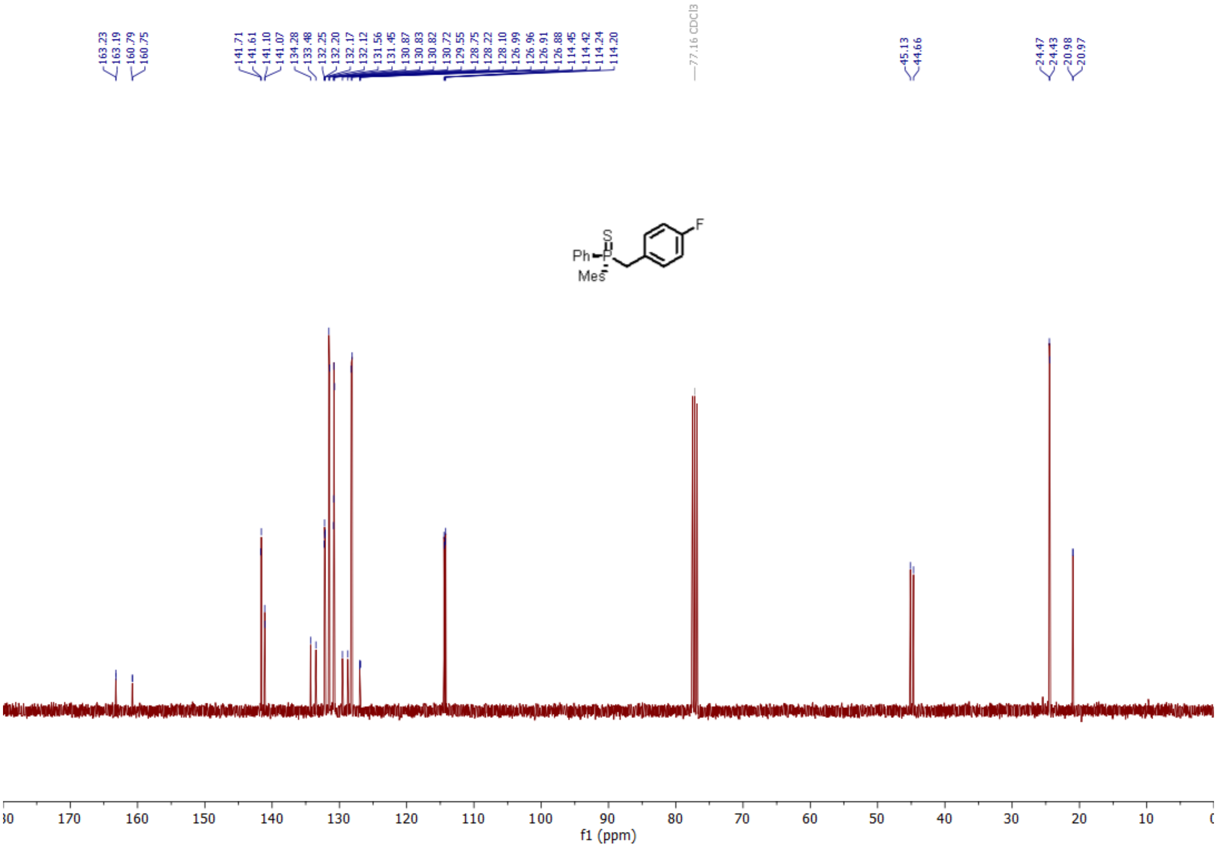

^{31}P NMR (162 MHz, $CDCl_3$) spectrum of **2’e**

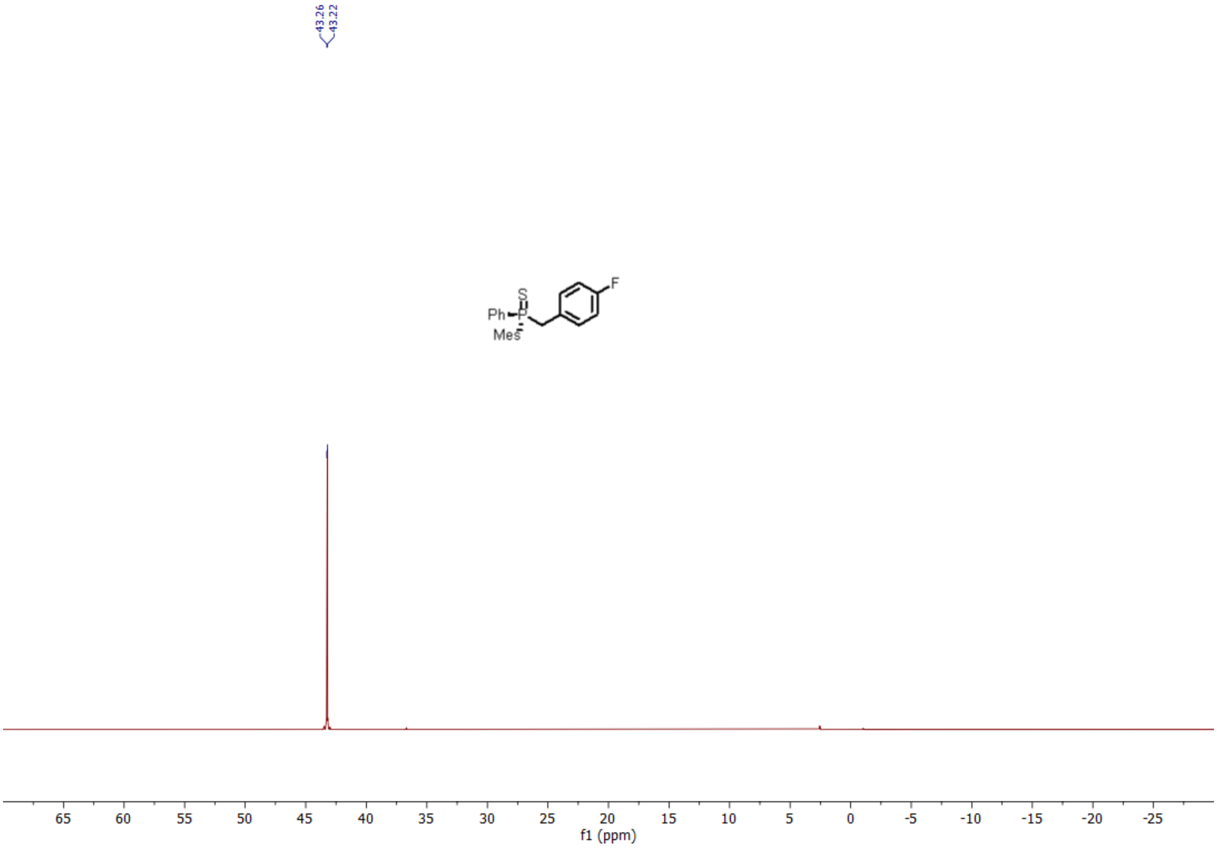

^{19}F NMR (376 MHz, $CDCl_3$) spectrum of **2’d**

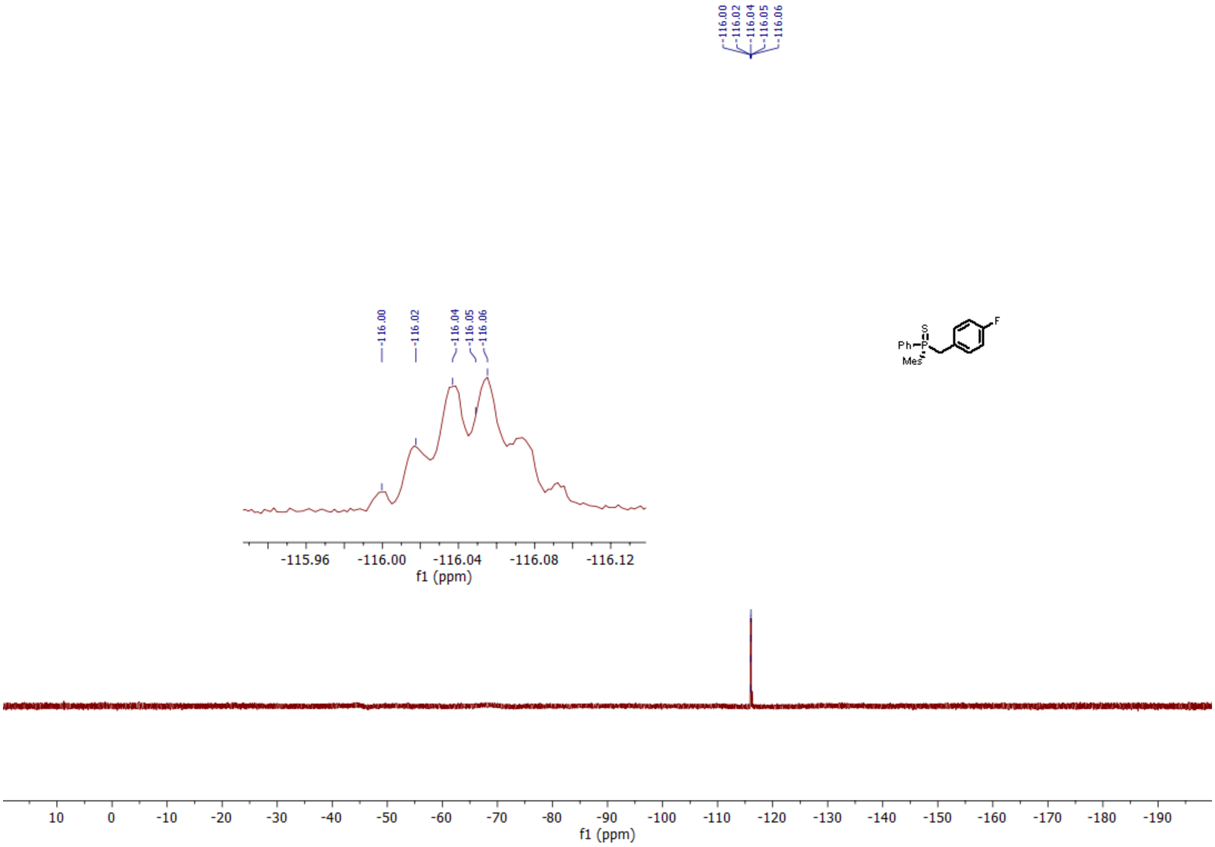

^{19}F NMR (376 MHz, $CDCl_3$) spectrum of **2d**

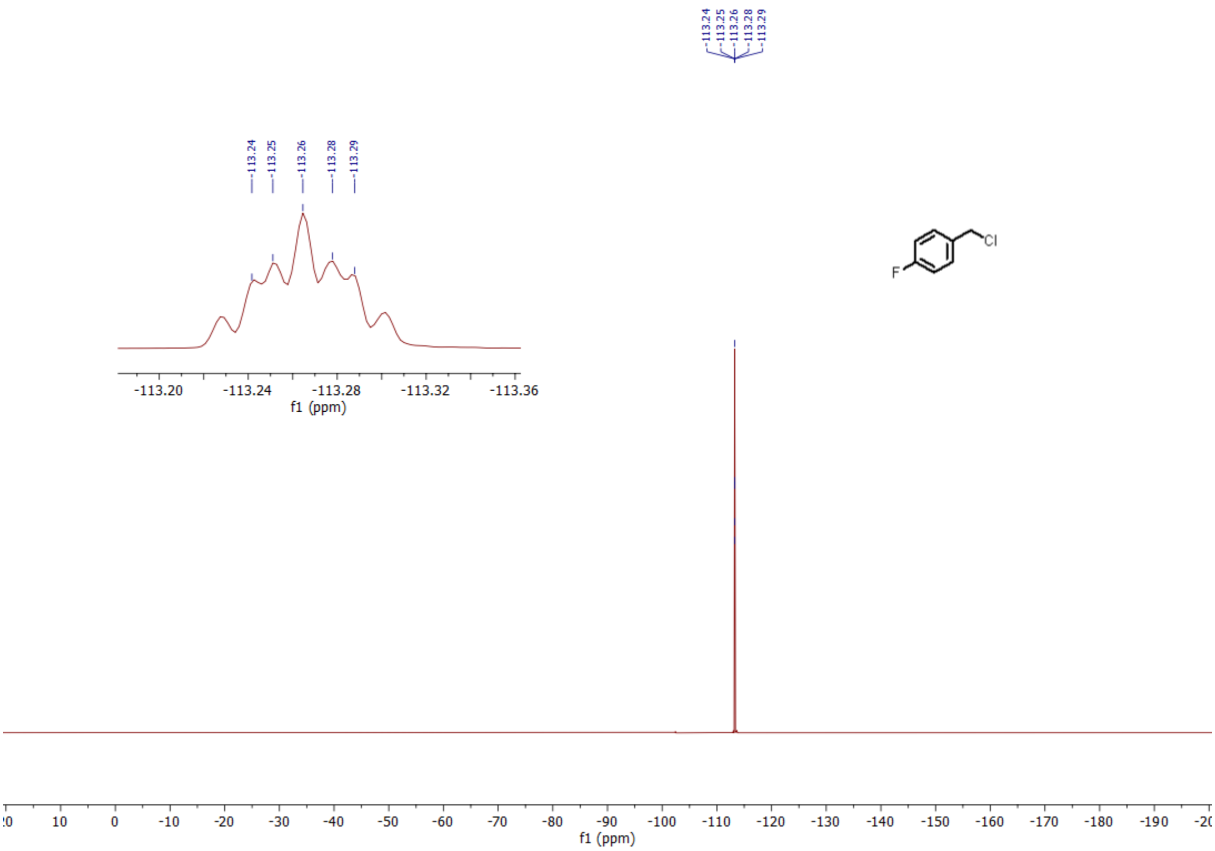

^{1}H NMR (400 MHz, $CDCl_3$) spectrum of **2'f**

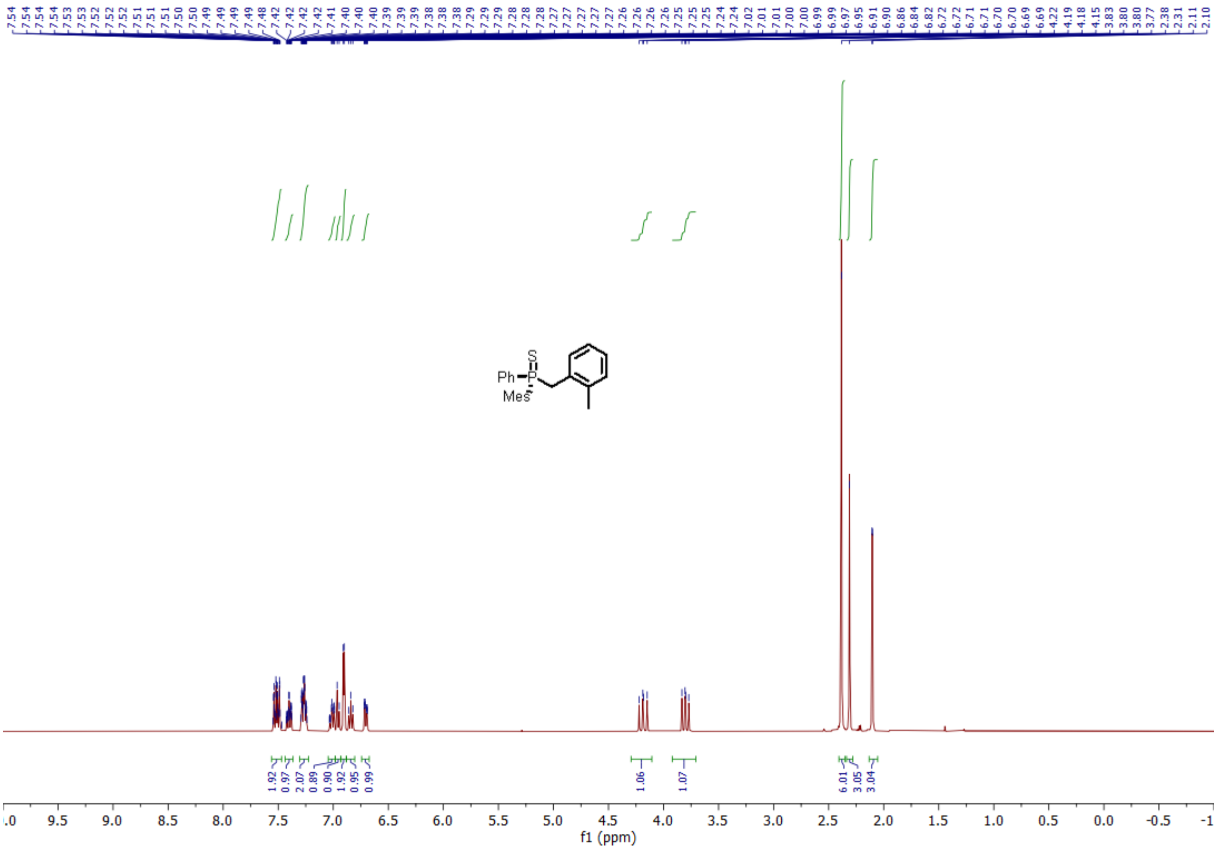

^{13}C NMR (101 MHz, $CDCl_3$)spectrum of **2’f**

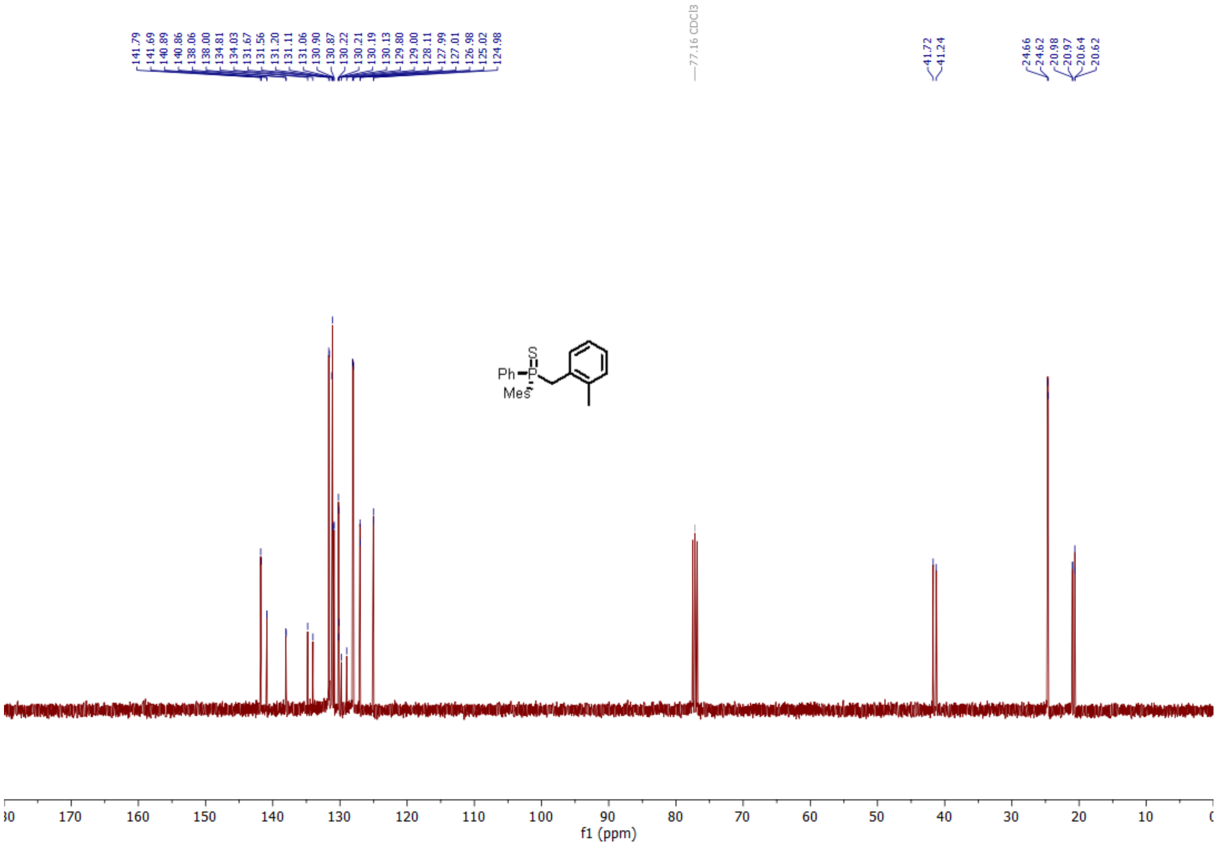

^{31}P NMR (162 MHz, $CDCl_3$) spectrum of **2’f**

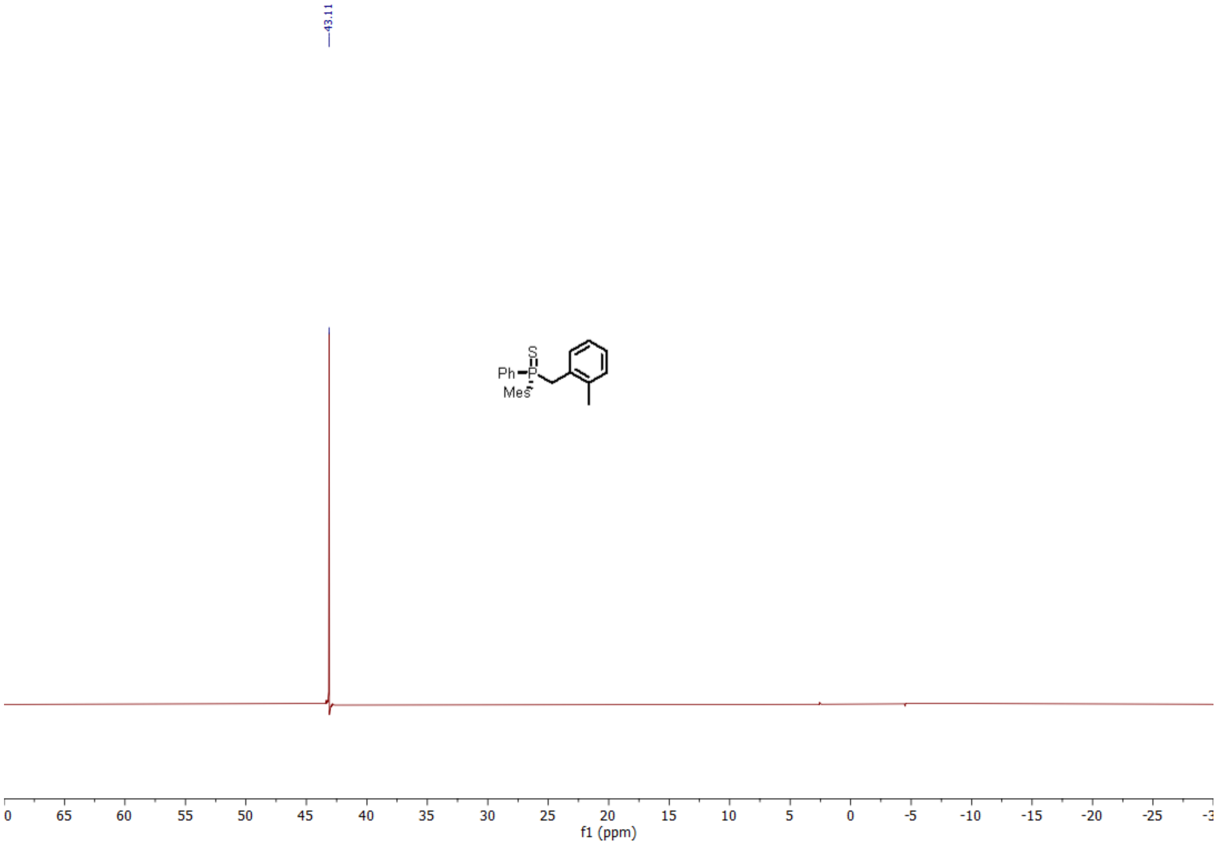

^{1}H NMR (400 MHz, $CDCl_3$) spectrum of **2’g**

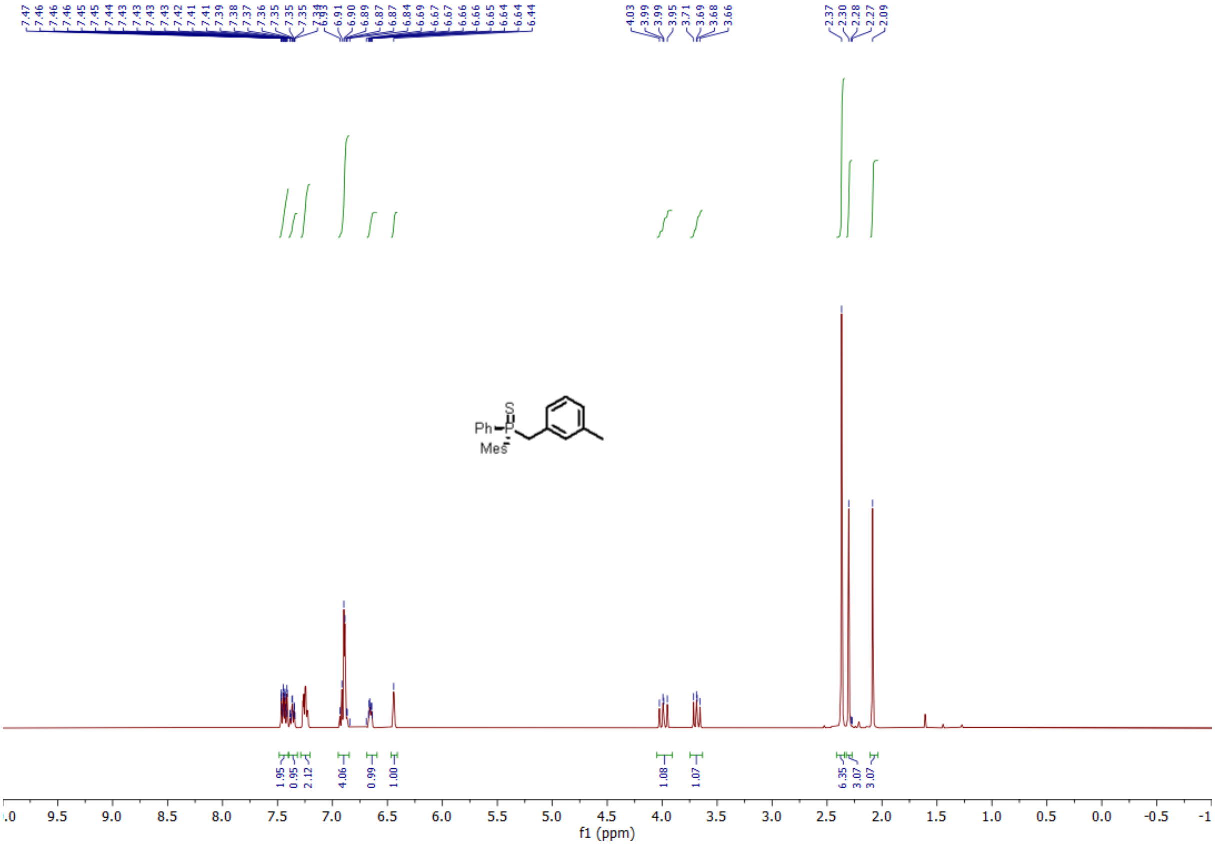

^{13}C NMR (101 MHz, $CDCl_3$)spectrum of **2’g**

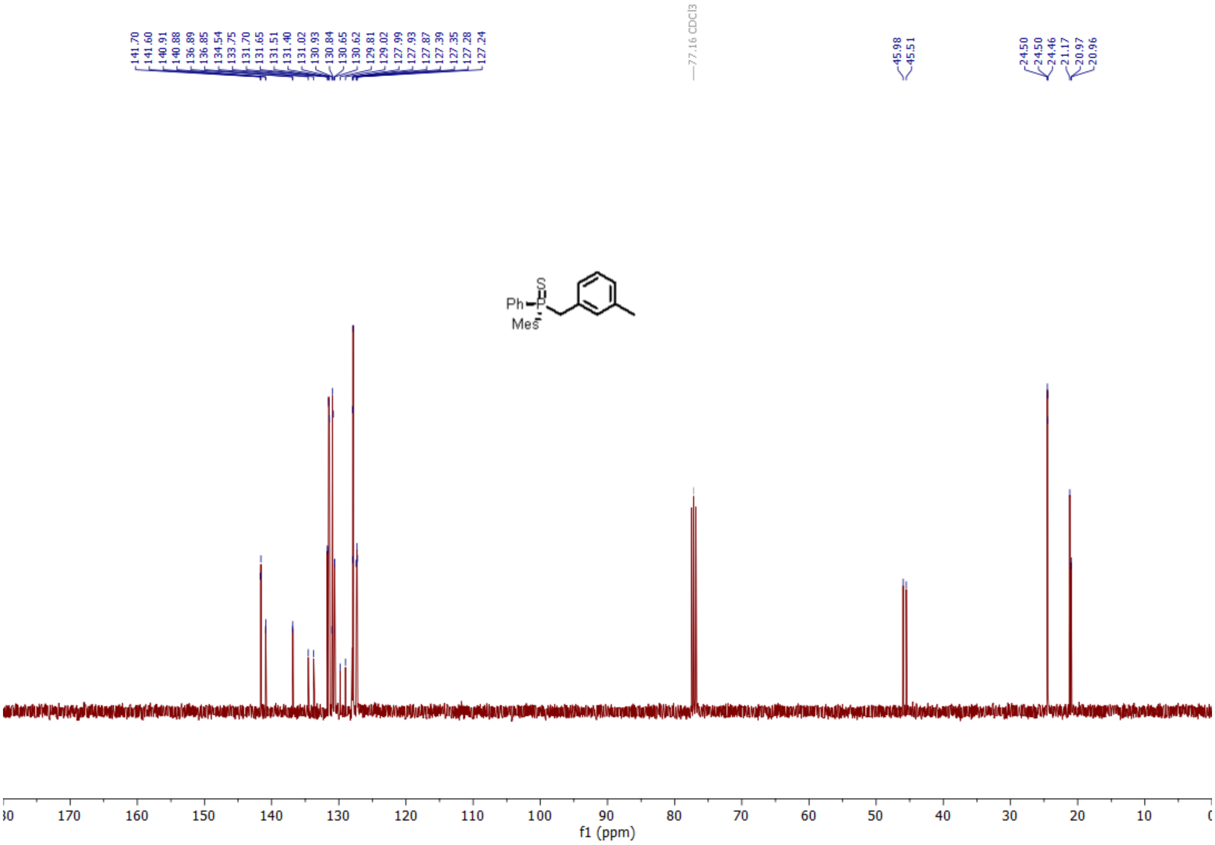

^{31}P NMR (162 MHz, $CDCl_3$) spectrum of **2’g**

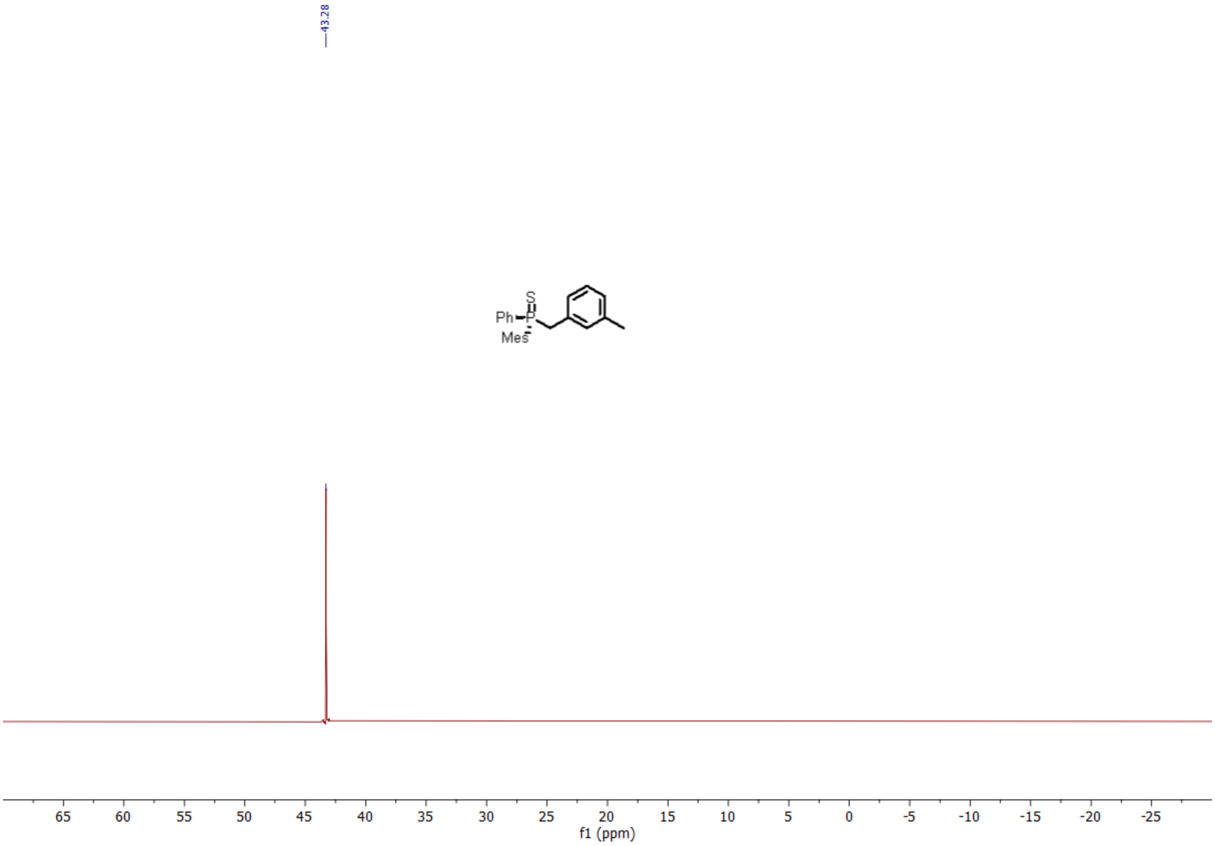

^{1}H NMR (400 MHz, $CDCl_3$) spectrum of **2’h**

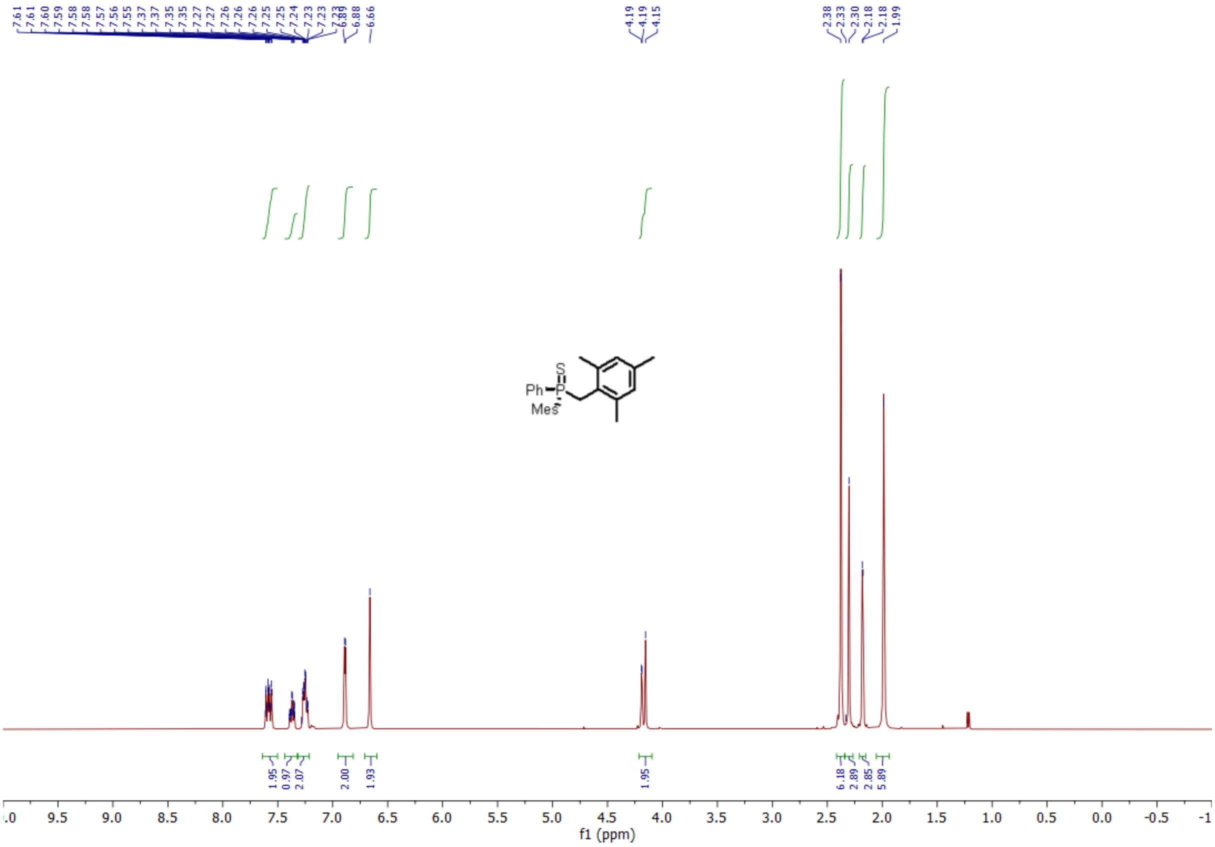

^{13}C NMR (101 MHz, $CDCl_3$)spectrum of **2’h**

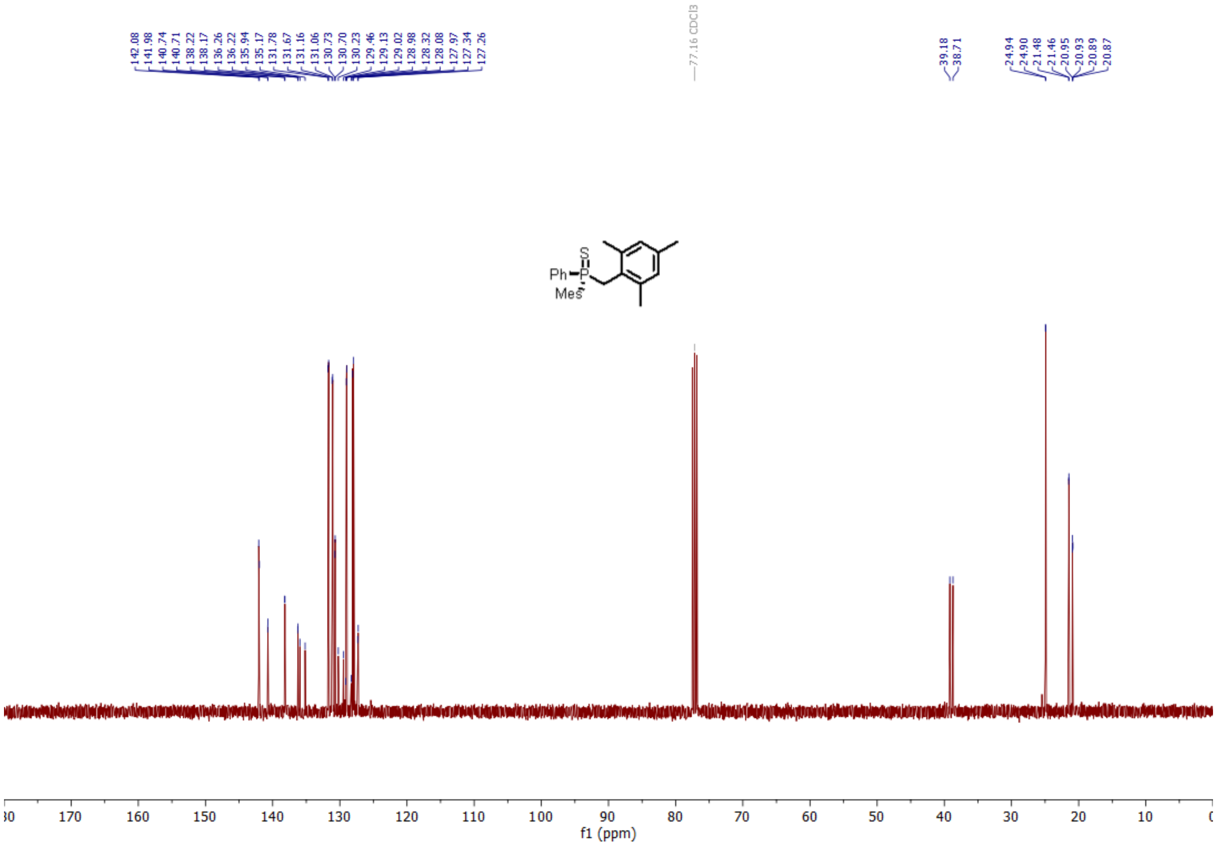

^{31}P NMR (162 MHz, $CDCl_3$) spectrum of **2’h**

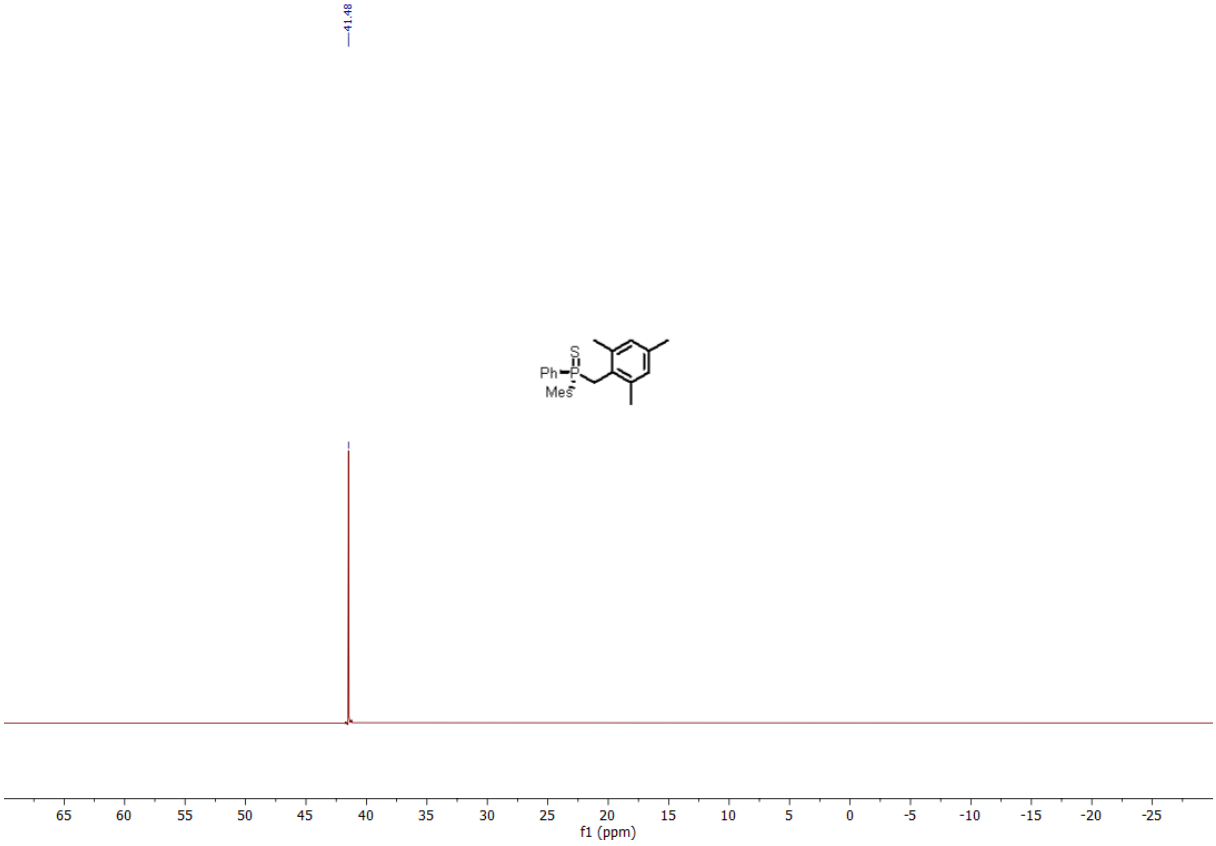

^{1}H NMR (400 MHz, $CDCl_3$) spectrum of **2'i**

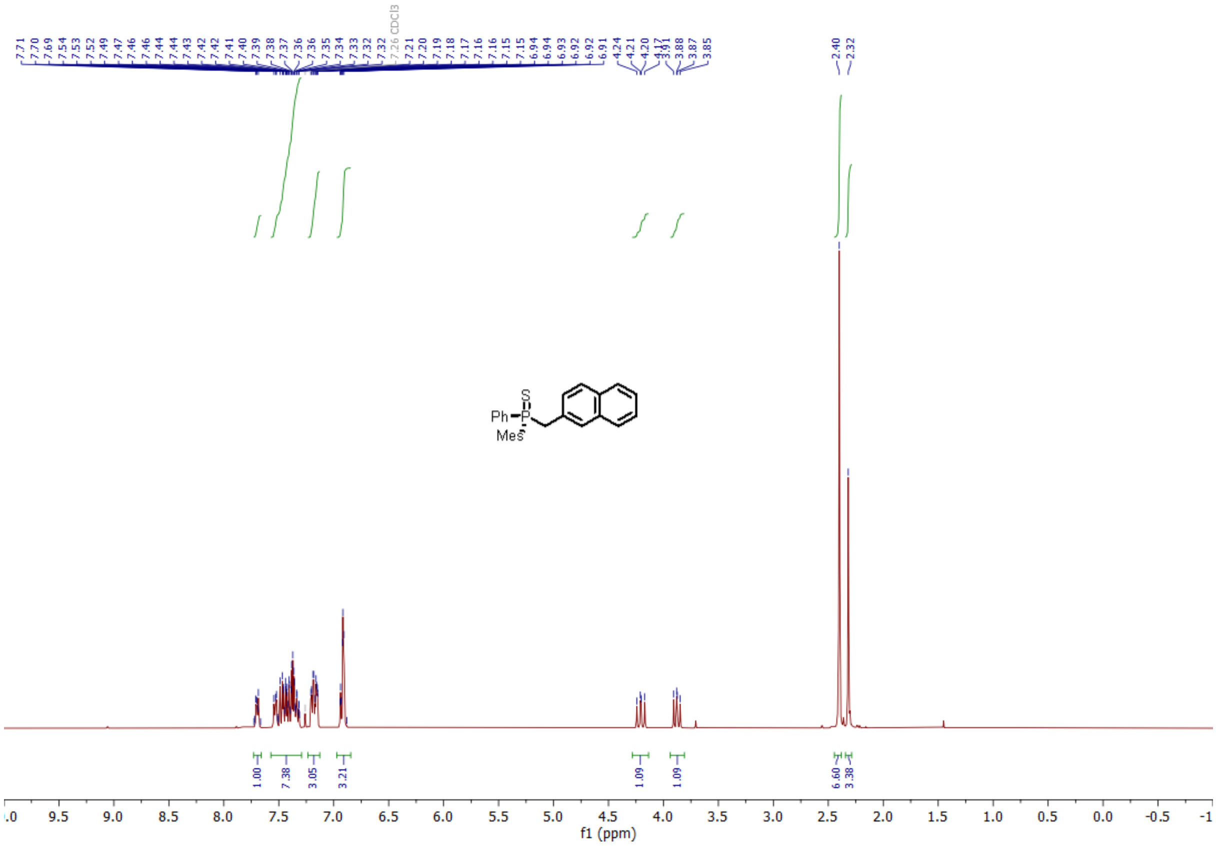

^{13}C NMR (101 MHz, $CDCl_3$)spectrum of **2'i**

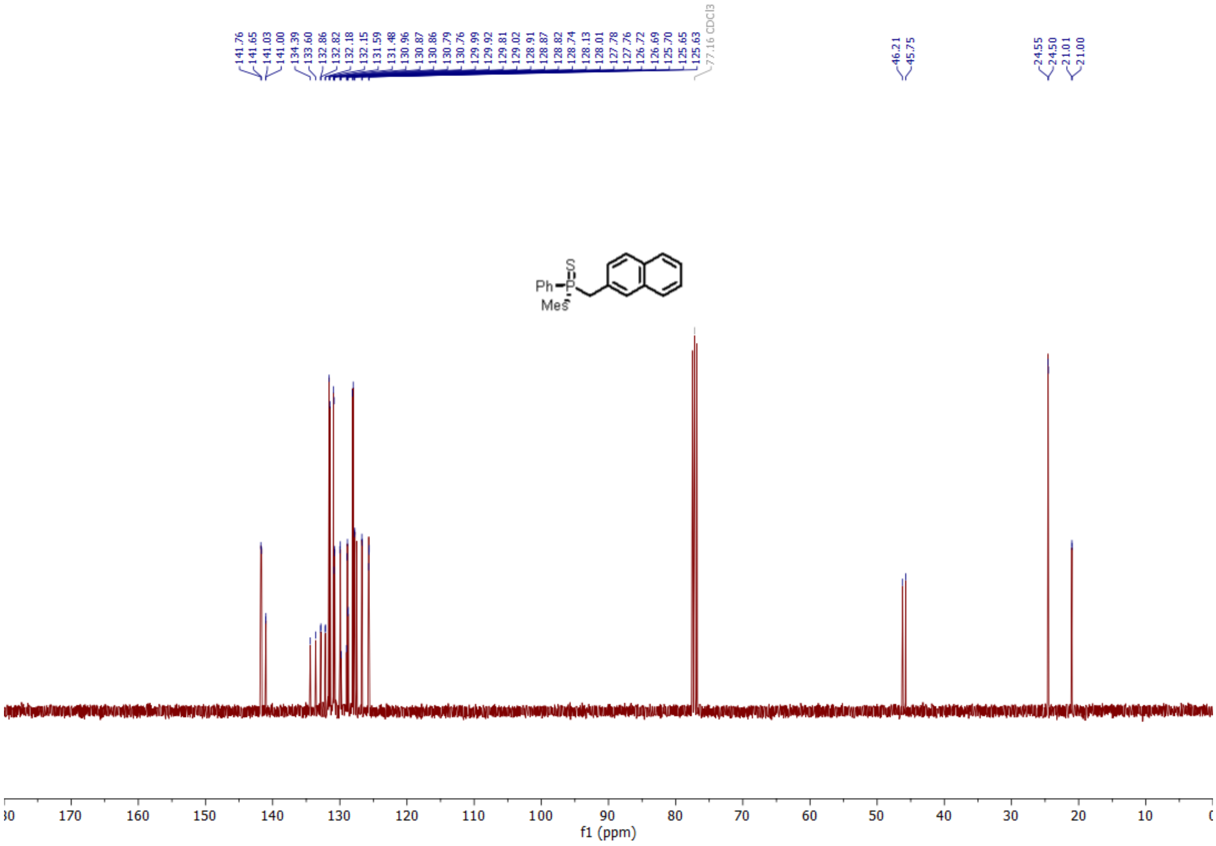

^{31}P NMR (162 MHz, $CDCl_3$) spectrum of **2’i**

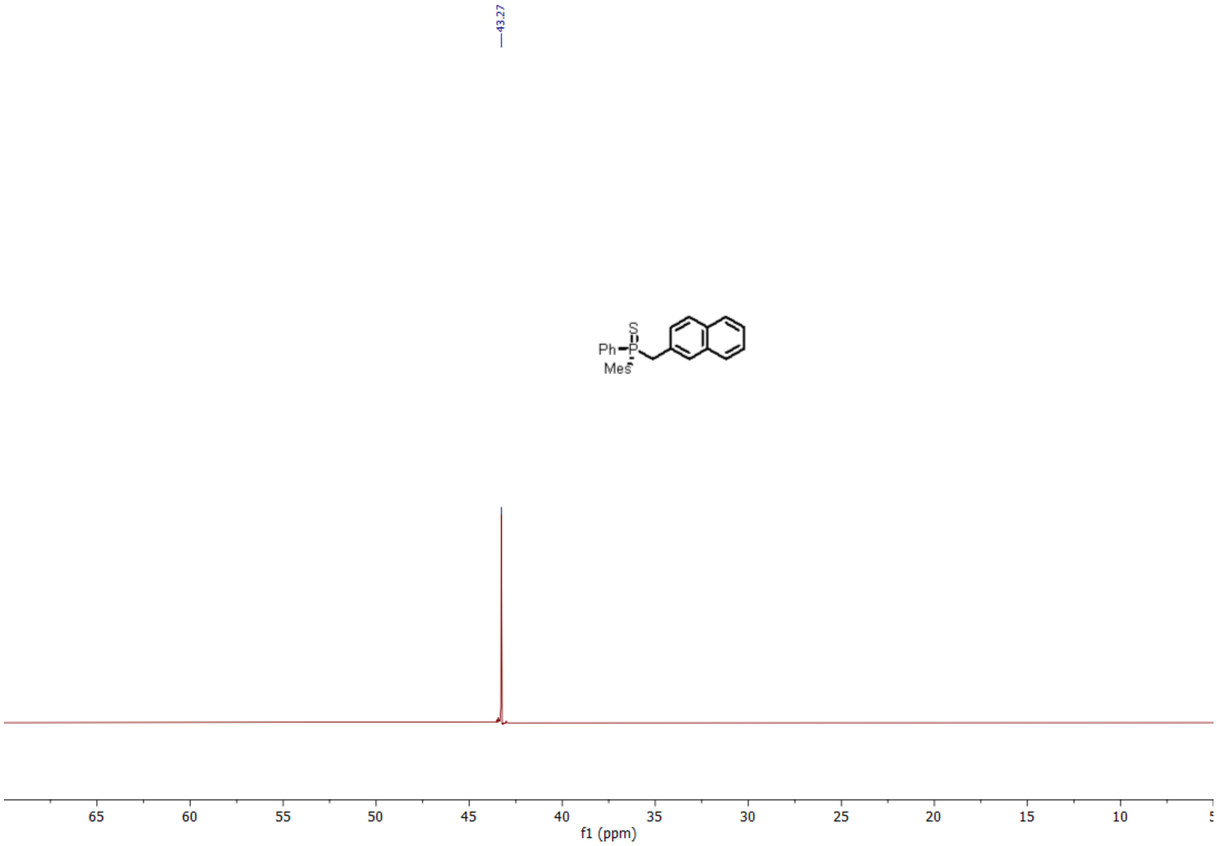

^{1}H NMR (400 MHz, $CDCl_3$) spectrum of **2’j**

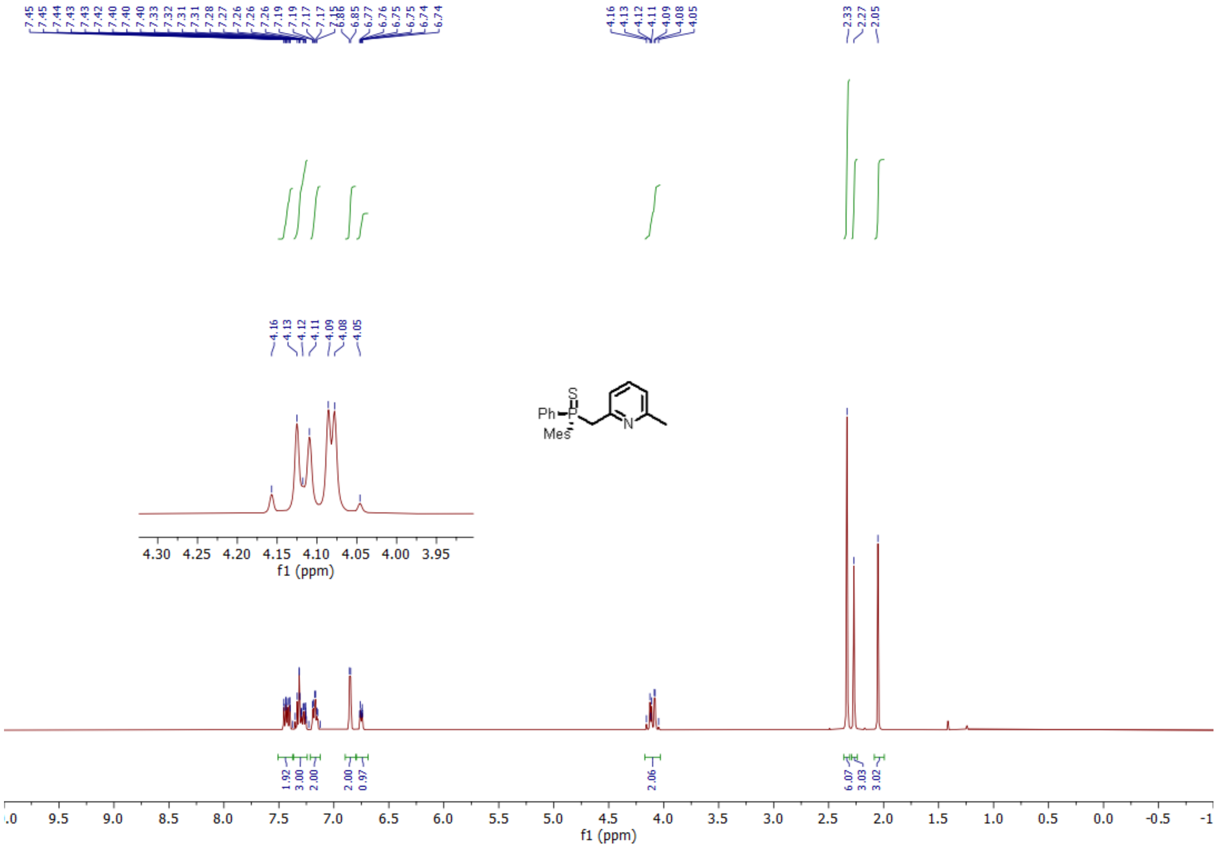

^{13}C NMR (101 MHz, CDCl$_3$)spectrum of **2'j**

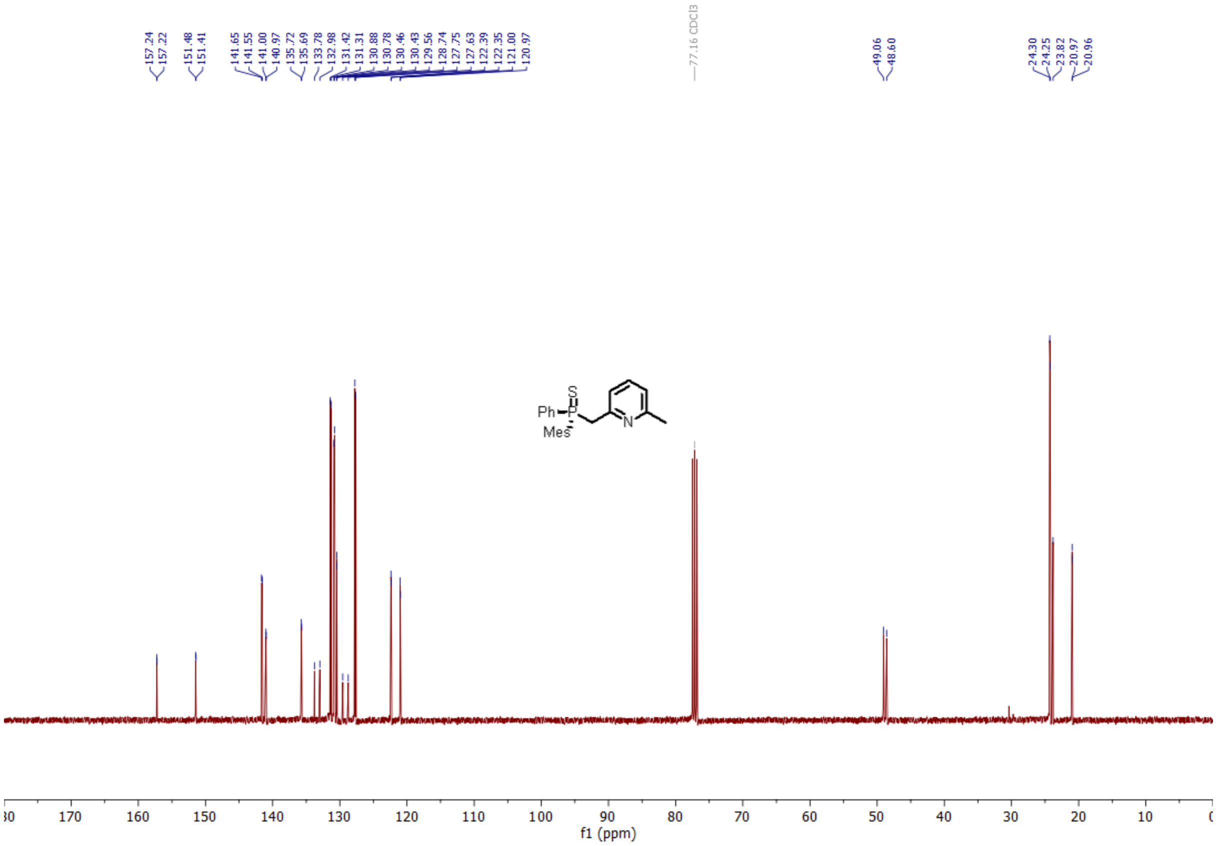

^{31}P NMR (162 MHz, CDCl$_3$) spectrum of **2'j**

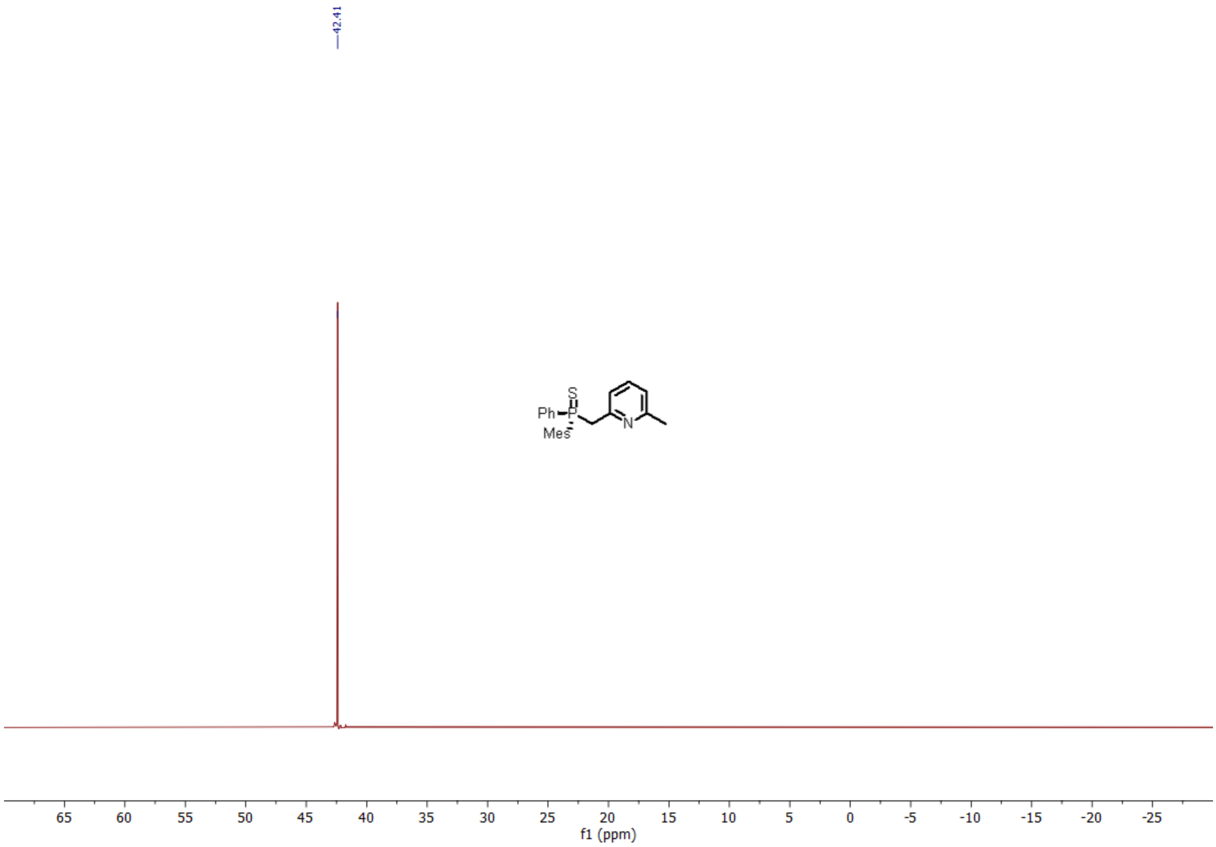

^{1}H NMR (400 MHz, $CDCl_3$) spectrum of **2’k**

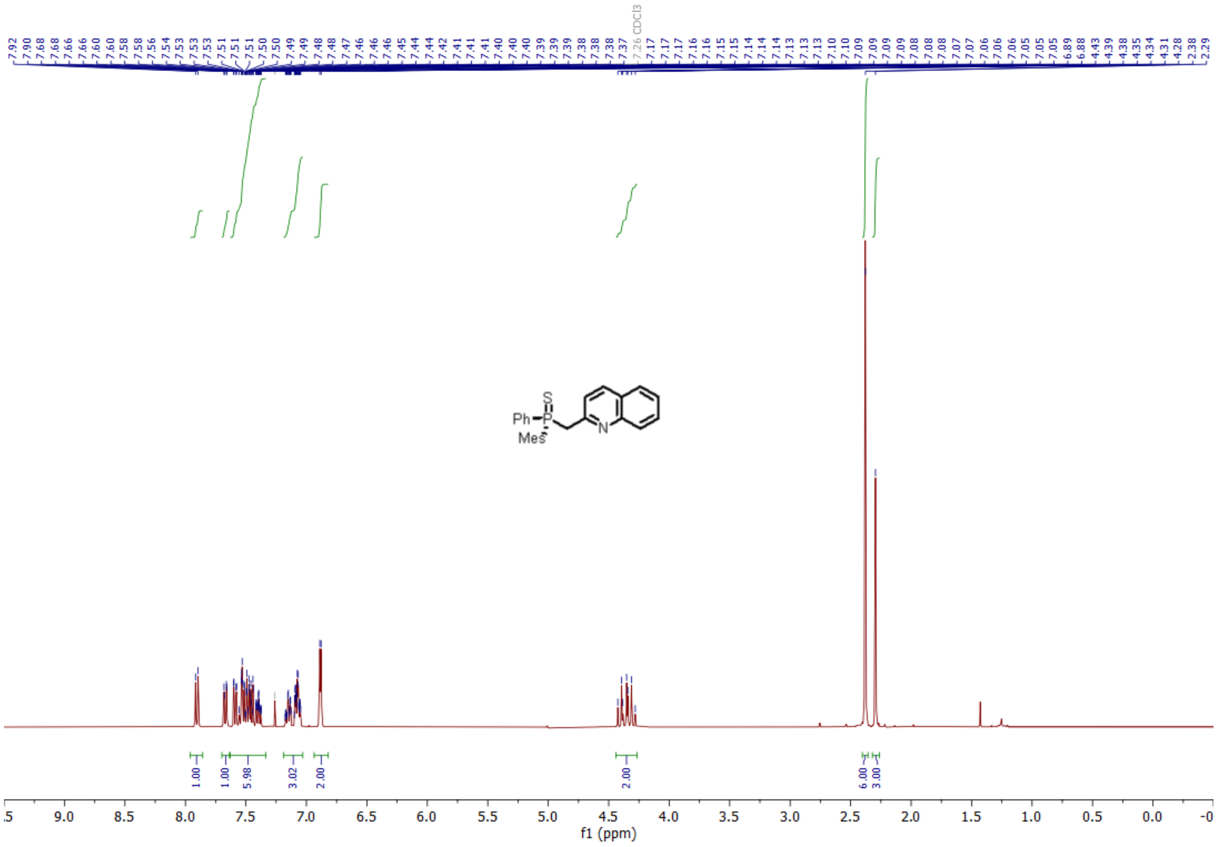

^{13}C NMR (101 MHz, $CDCl_3$)spectrum of **2’k**

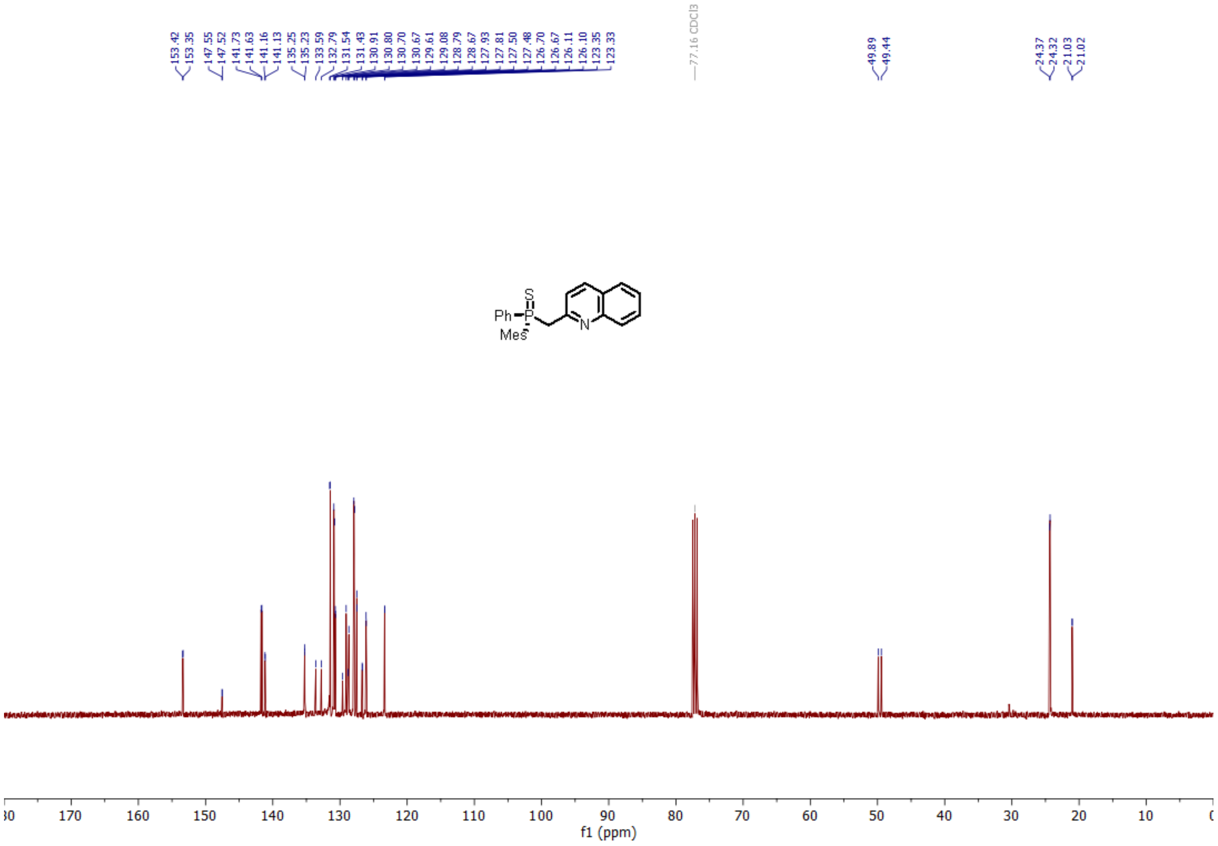

^{31}**P NMR** (162 MHz, $CDCl_3$) spectrum of **2’k**

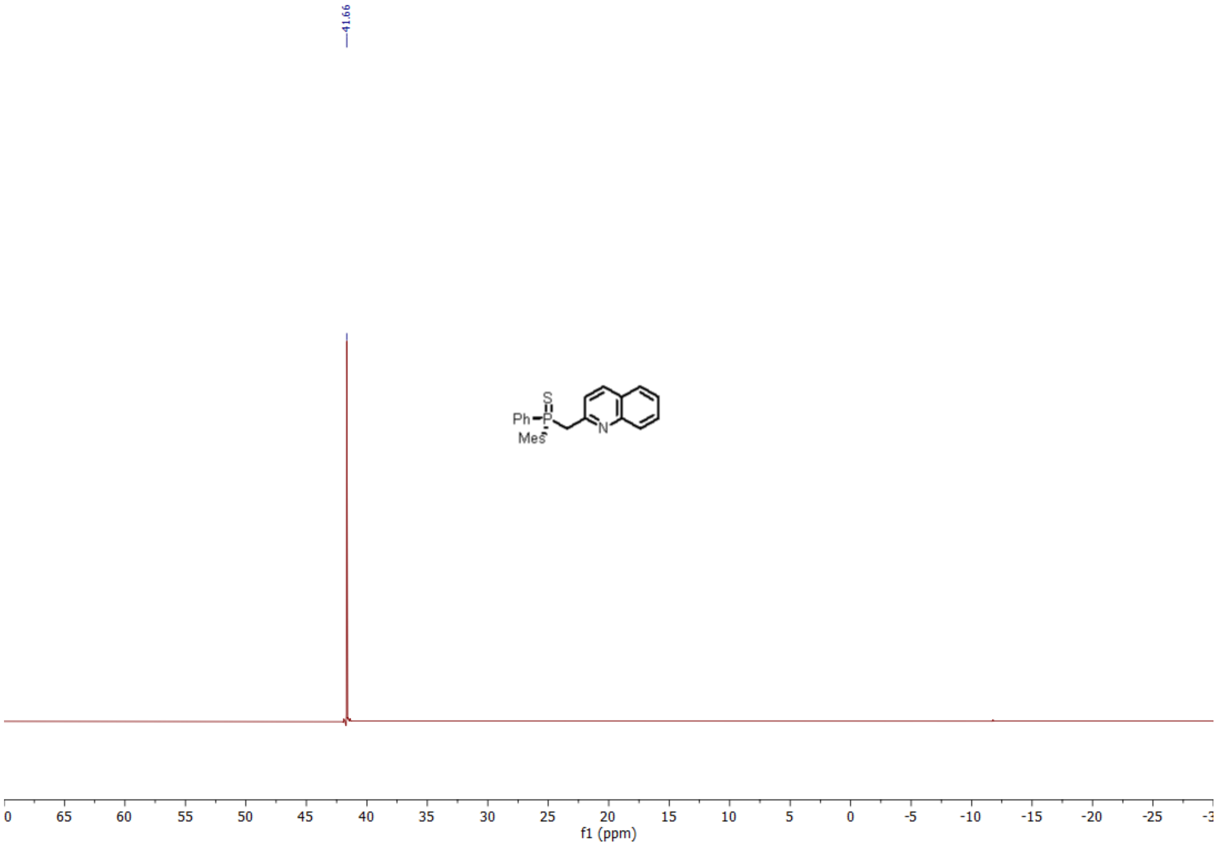

^{1}**H NMR** (400 MHz, $CDCl_3$) spectrum of **2’l**

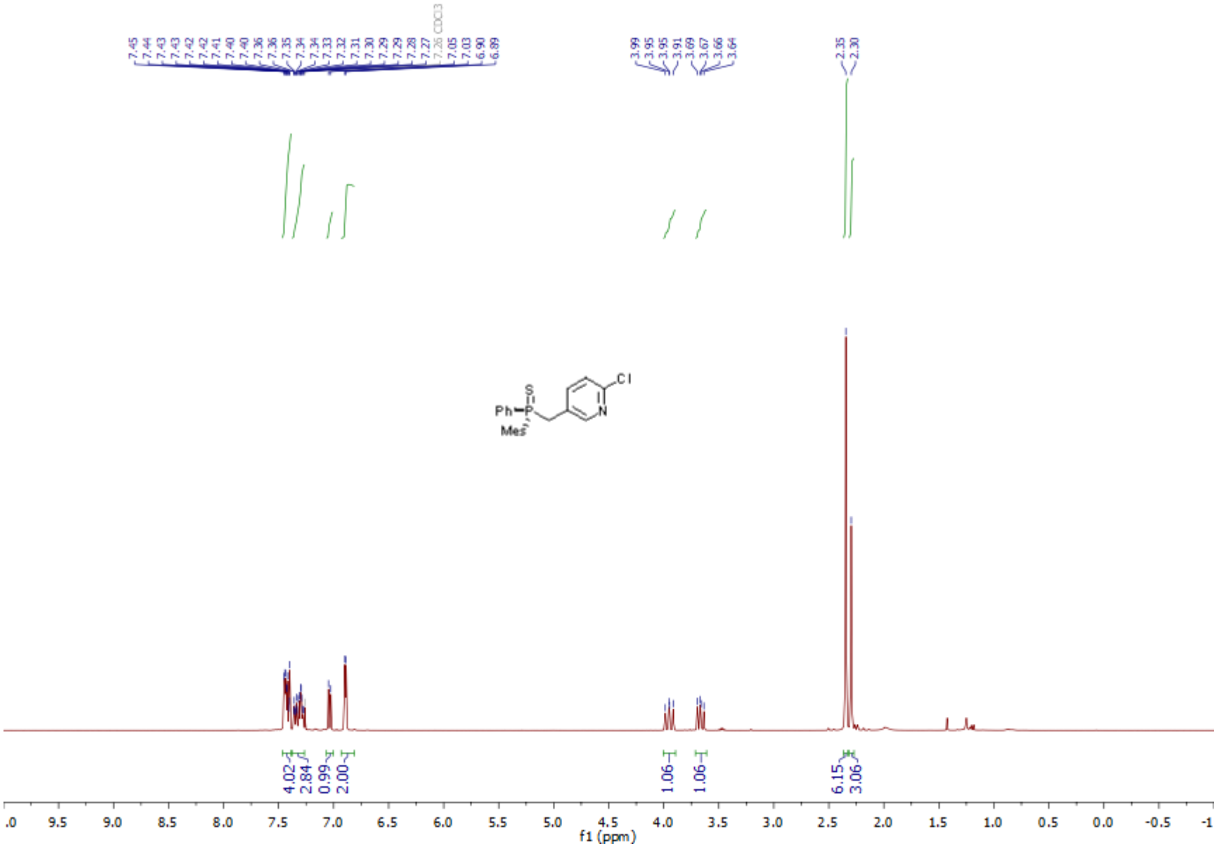

^{13}**C NMR** (101 MHz, $CDCl_3$)spectrum of **2'l**

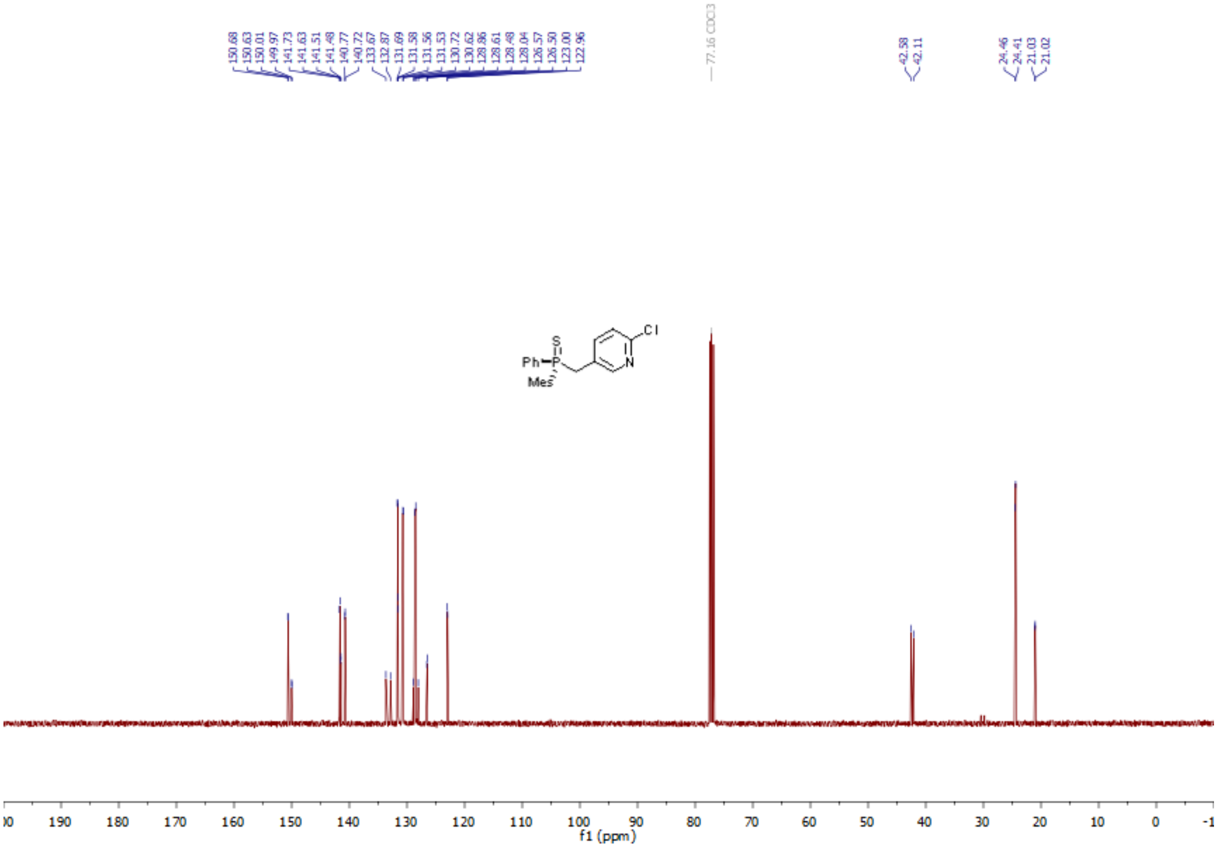

^{31}**P NMR** (162 MHz, $CDCl_3$) spectrum of **2'l**

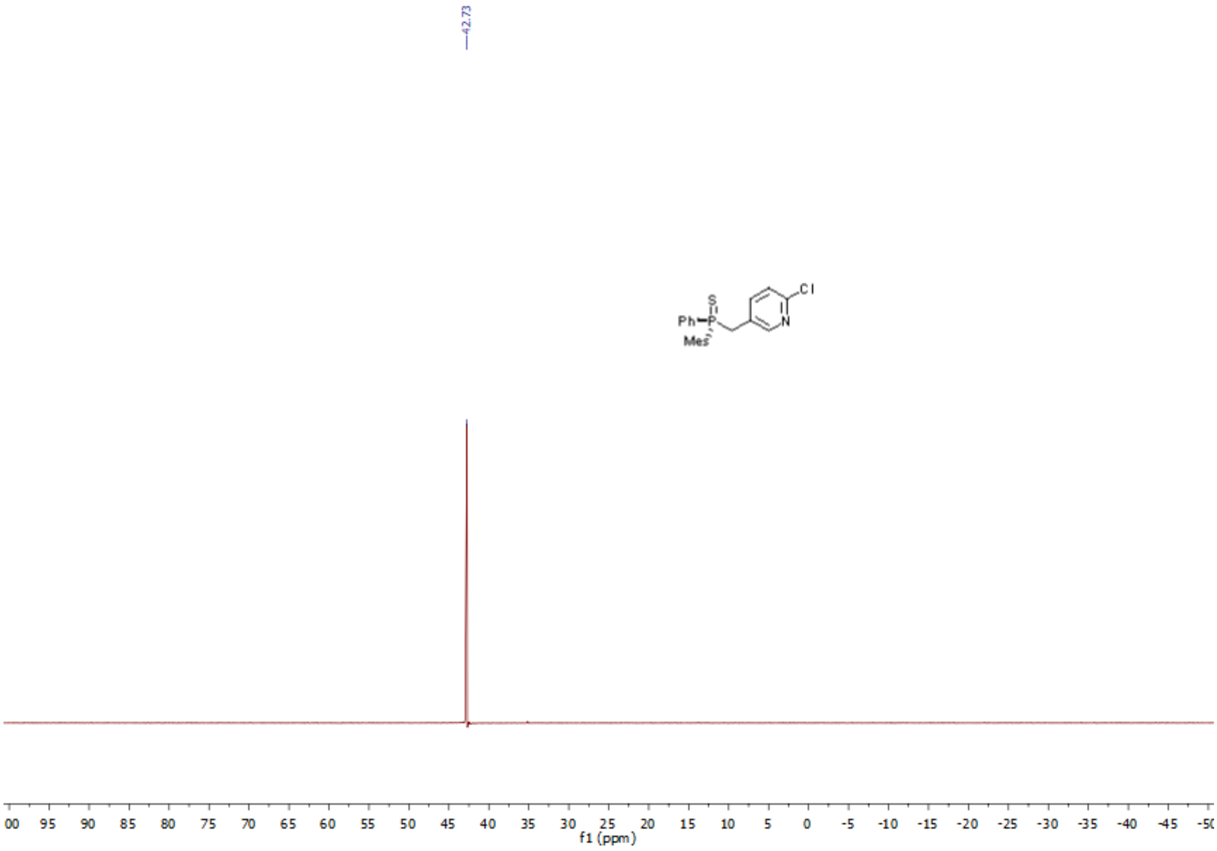

^{1}H NMR (400 MHz, $CDCl_3$) spectrum of **2'm**

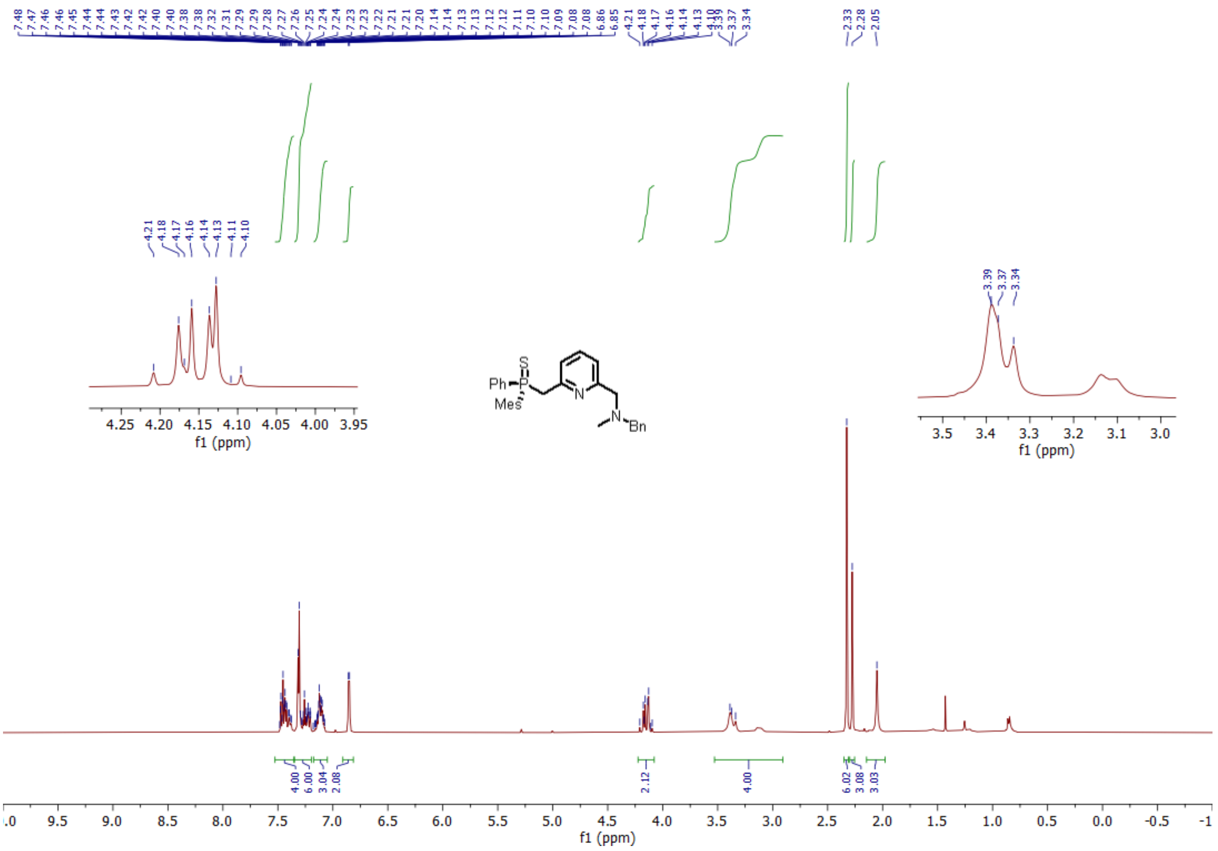

^{13}C NMR (101 MHz, $CDCl_3$)spectrum of **2'm**

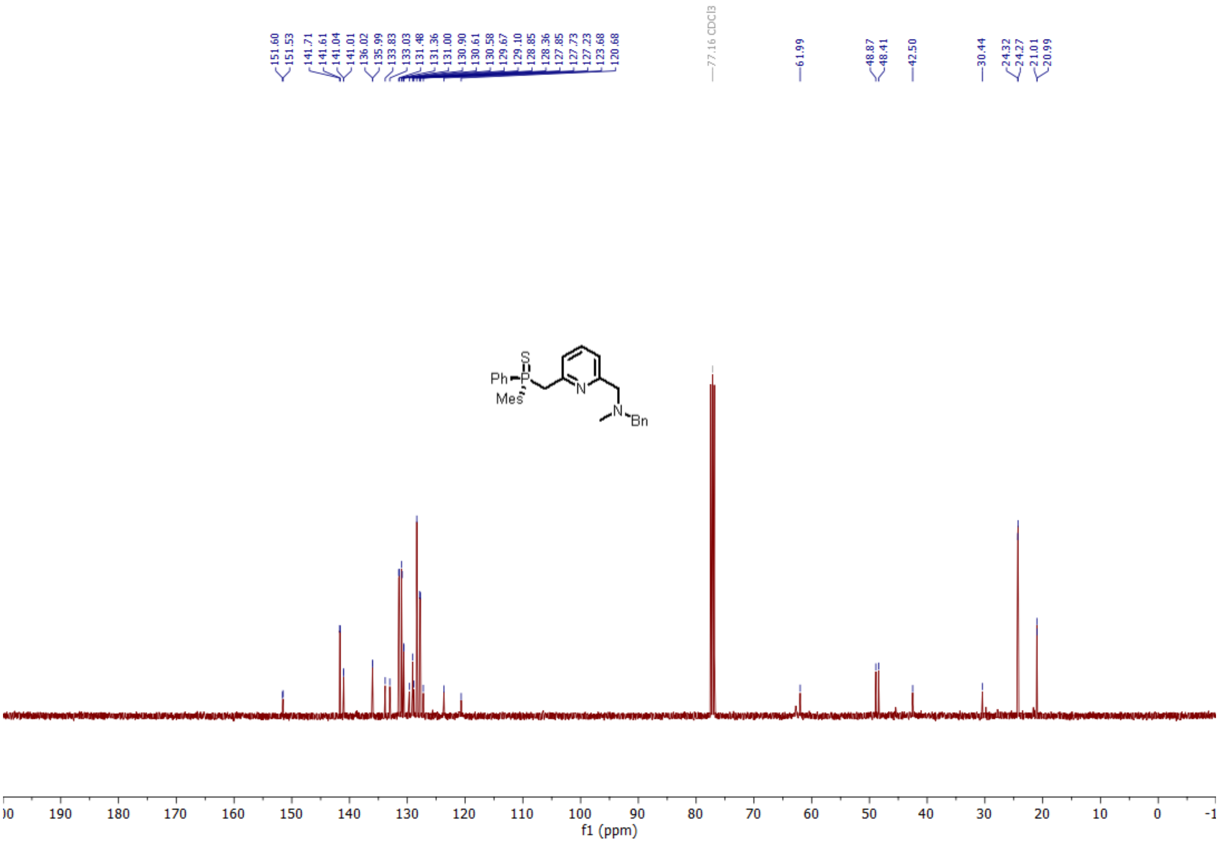

^{31}P NMR (162 MHz, CDCl$_3$) spectrum of **2’m**

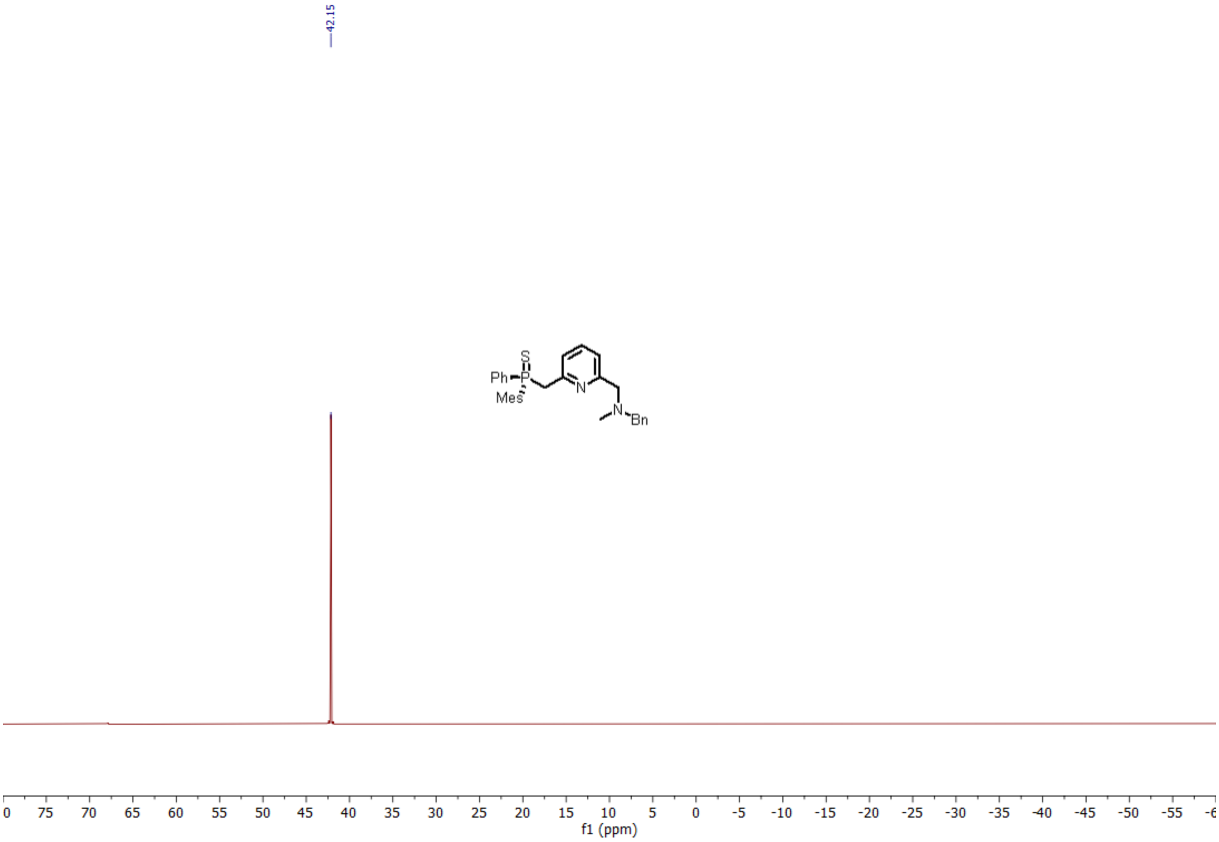

^{1}H NMR (400 MHz, CDCl$_3$) spectrum of **2’n**

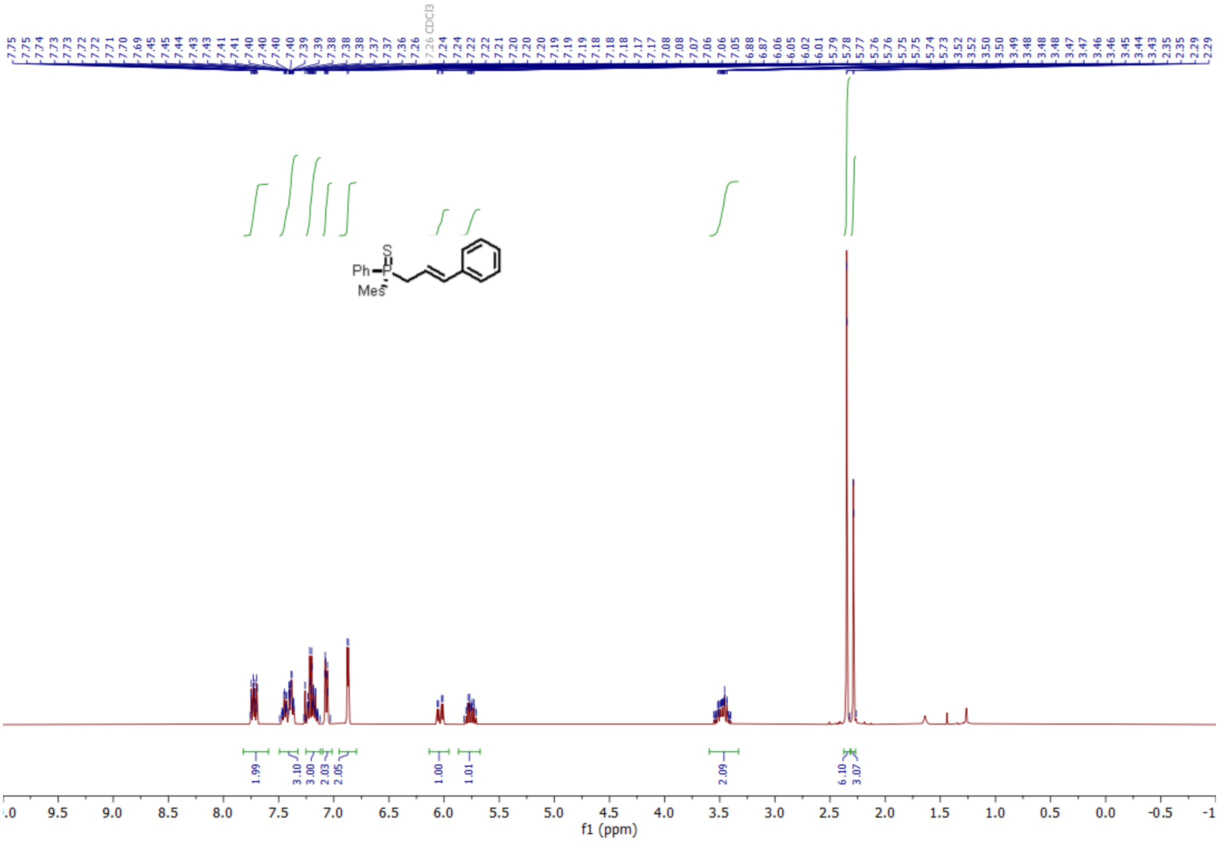

^{13}C NMR (101 MHz, $CDCl_3$)spectrum of **2’n**

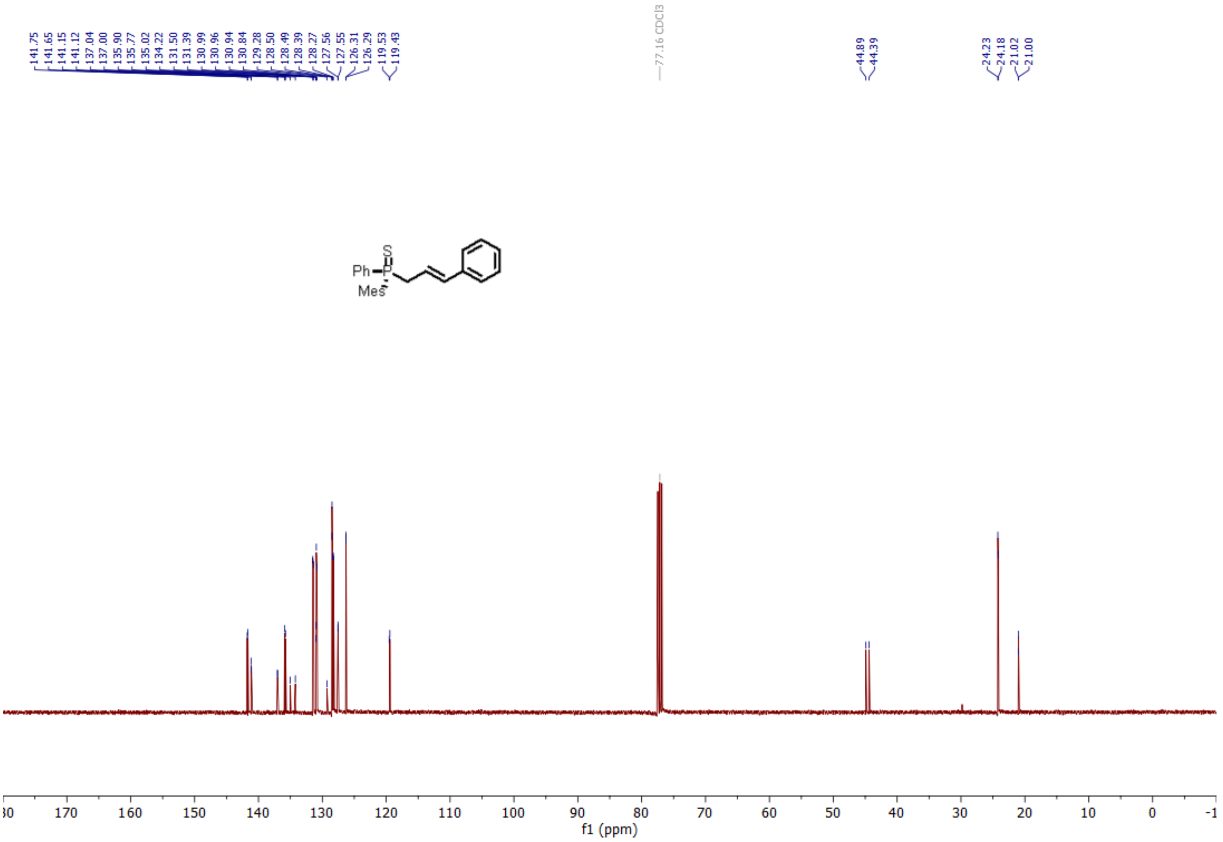

^{31}P NMR (162 MHz, $CDCl_3$) spectrum of **2’n**

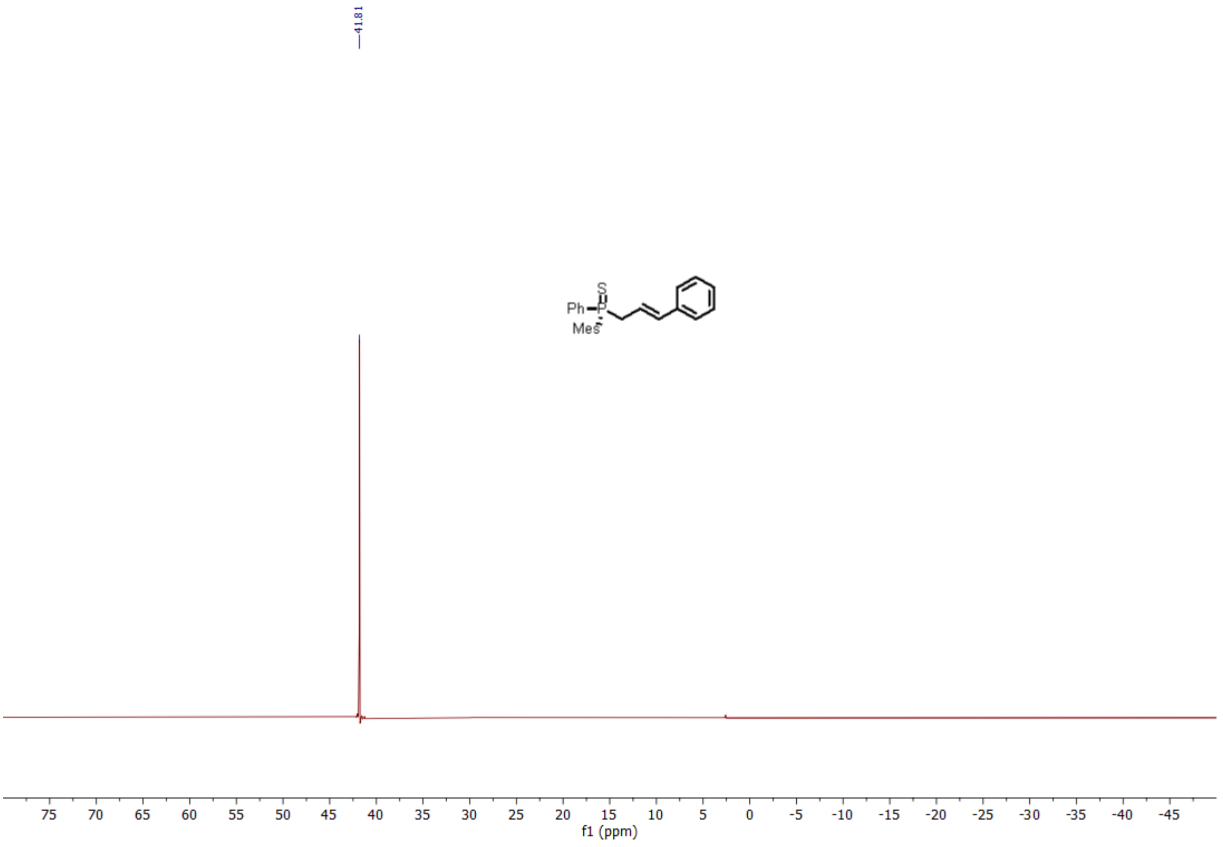

^{1}H **NMR** (400 MHz, $CDCl_3$) spectrum of **2'o**

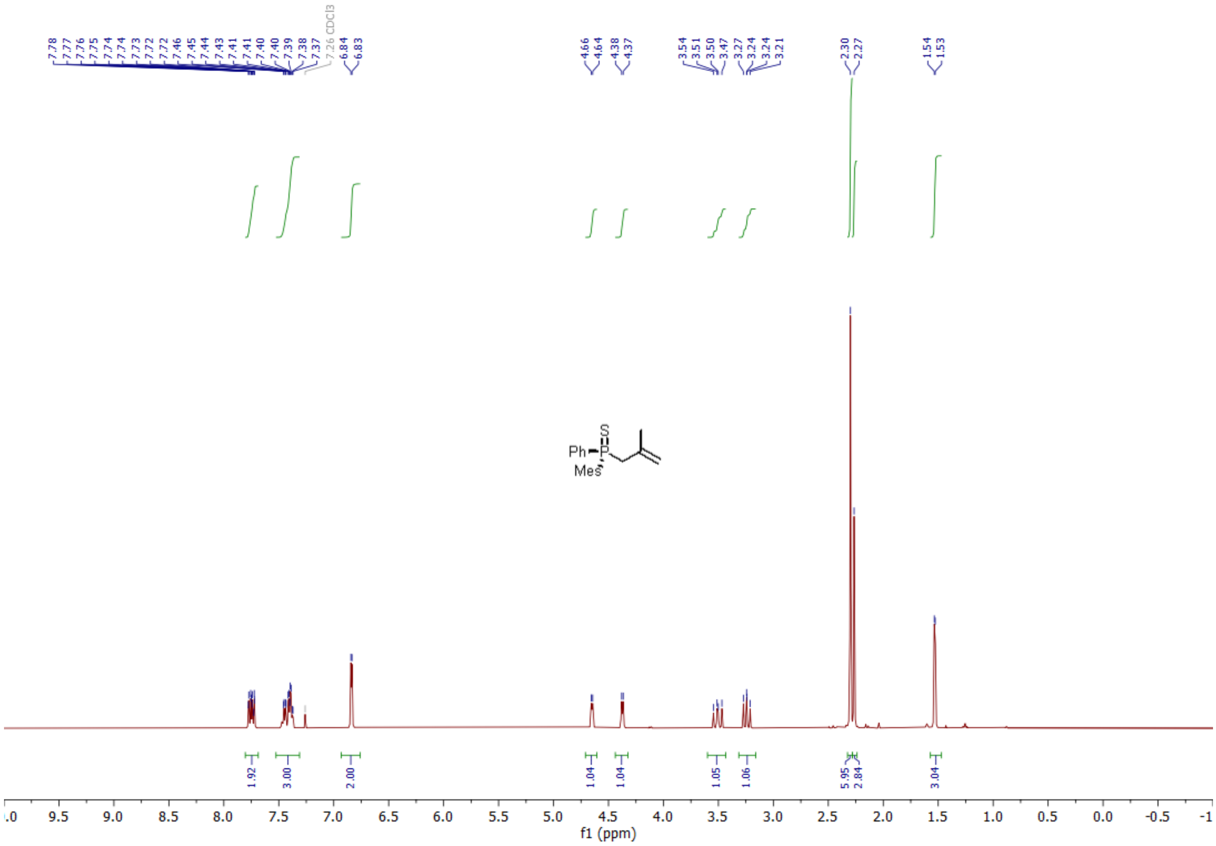

^{13}C **NMR** (101 MHz, $CDCl_3$)spectrum of **2'o**

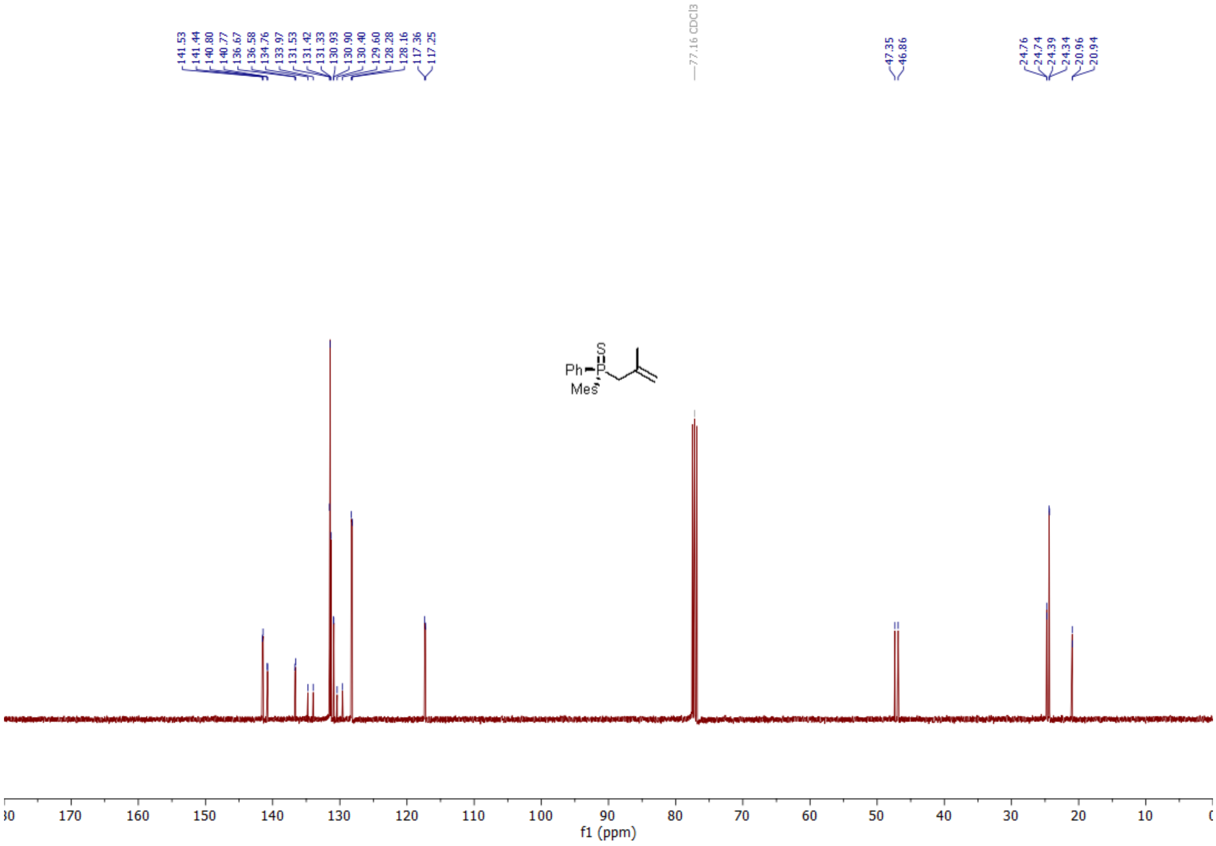

^{31}**P NMR** (162 MHz, $CDCl_3$) spectrum of **2’o**

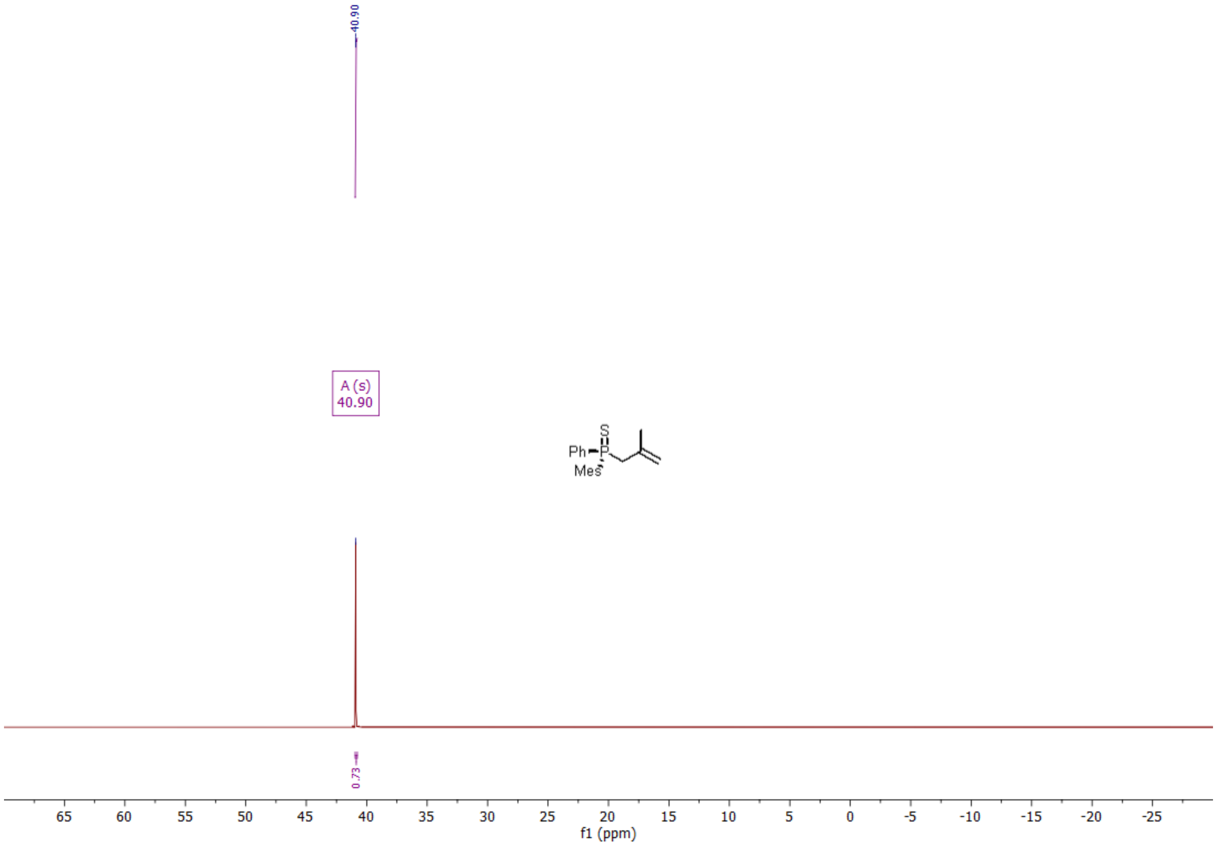

^{1}**H NMR** (400 MHz, $CDCl_3$) spectrum of **2’p**

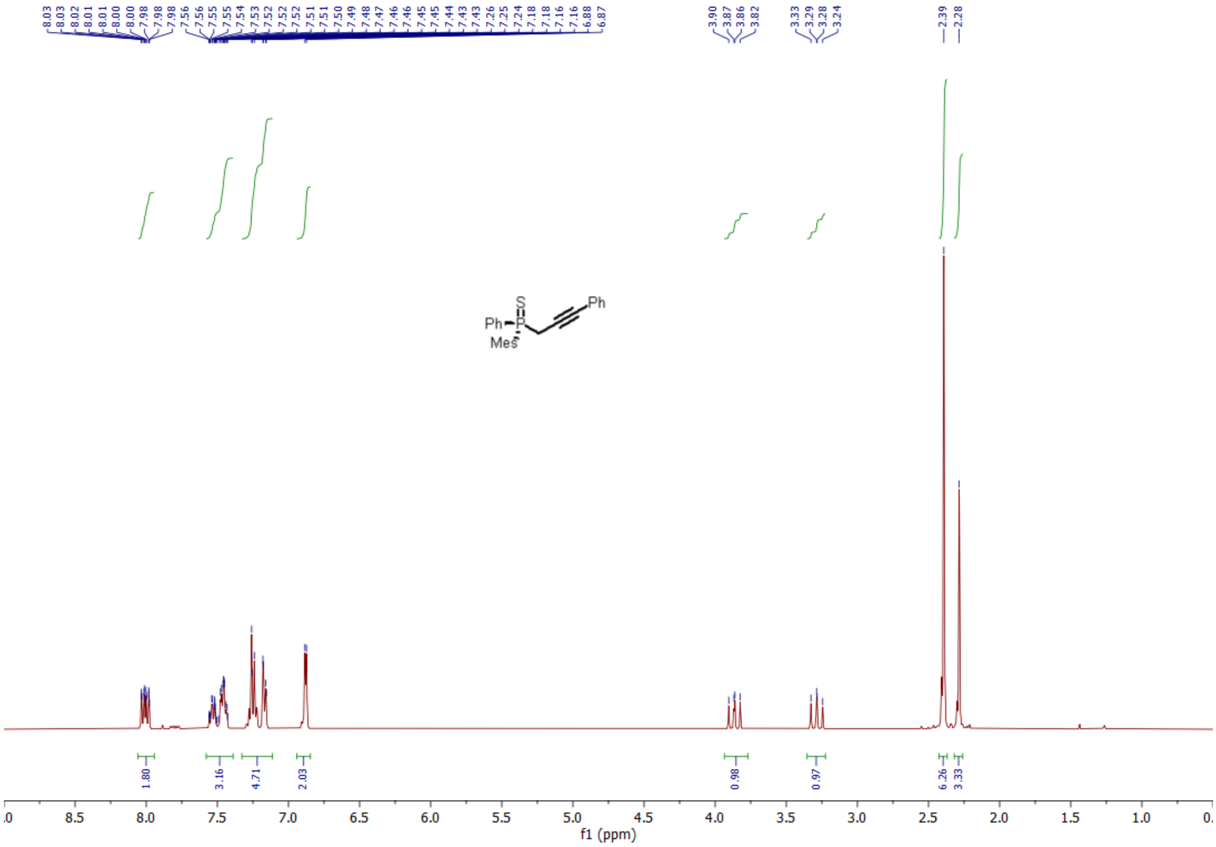

^{13}C NMR (101 MHz, $CDCl_3$)spectrum of **2’p**

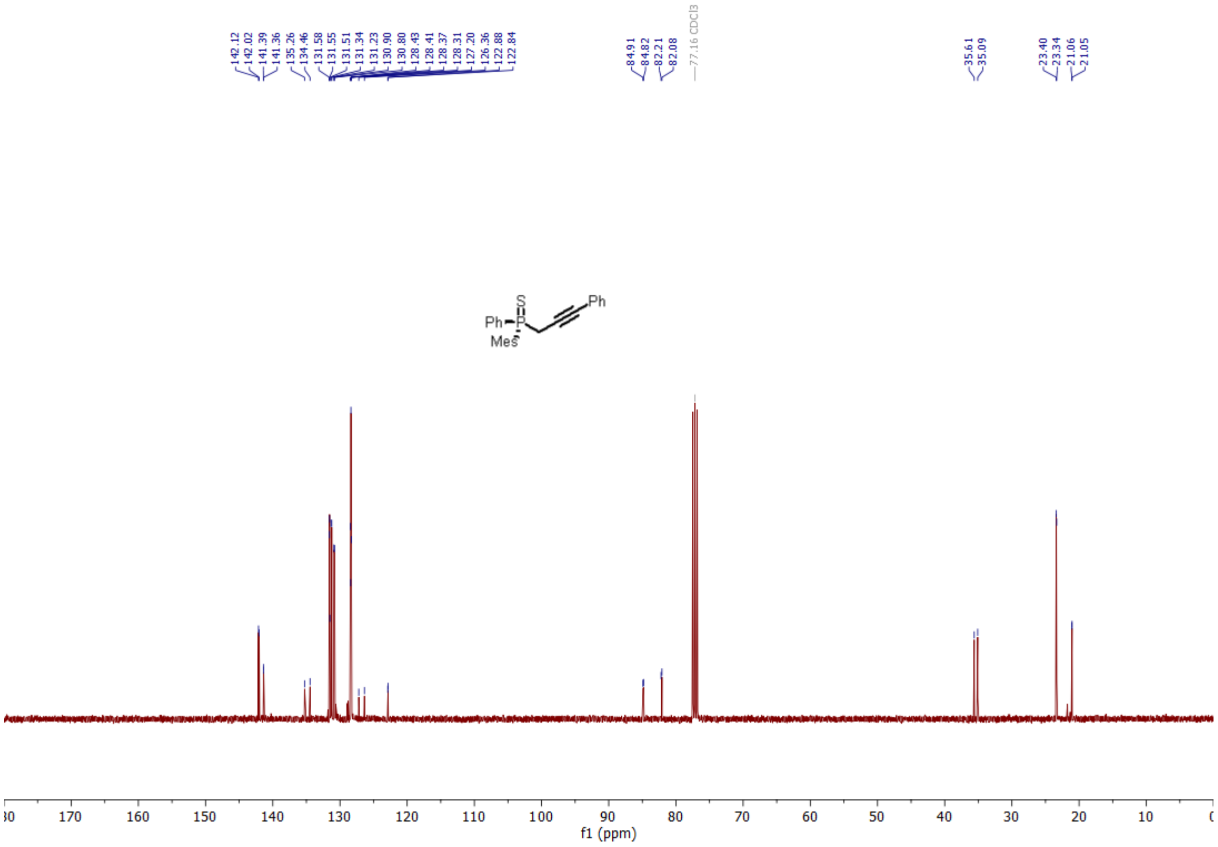

^{31}P NMR (162 MHz, $CDCl_3$) spectrum of **2’p**

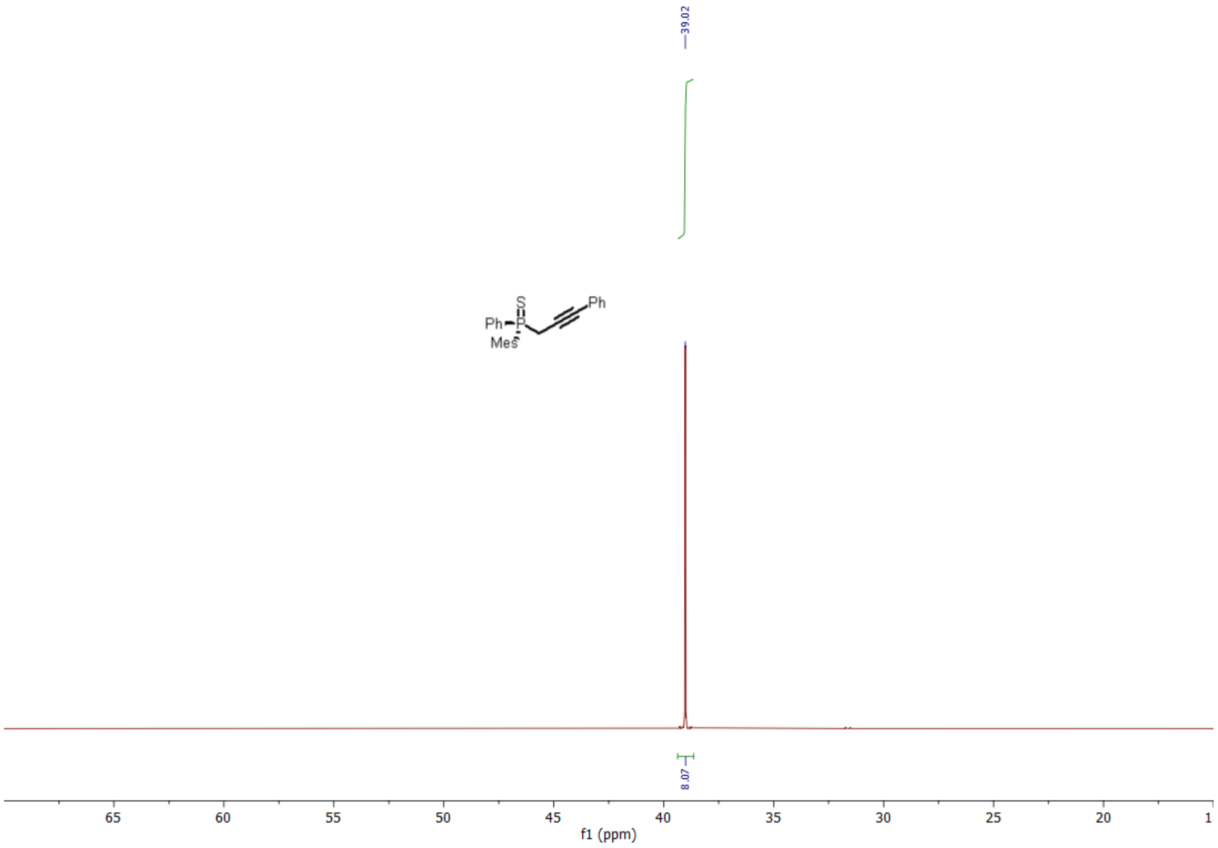

^{1}H NMR (400 MHz, $CDCl_3$) spectrum of **3’a**

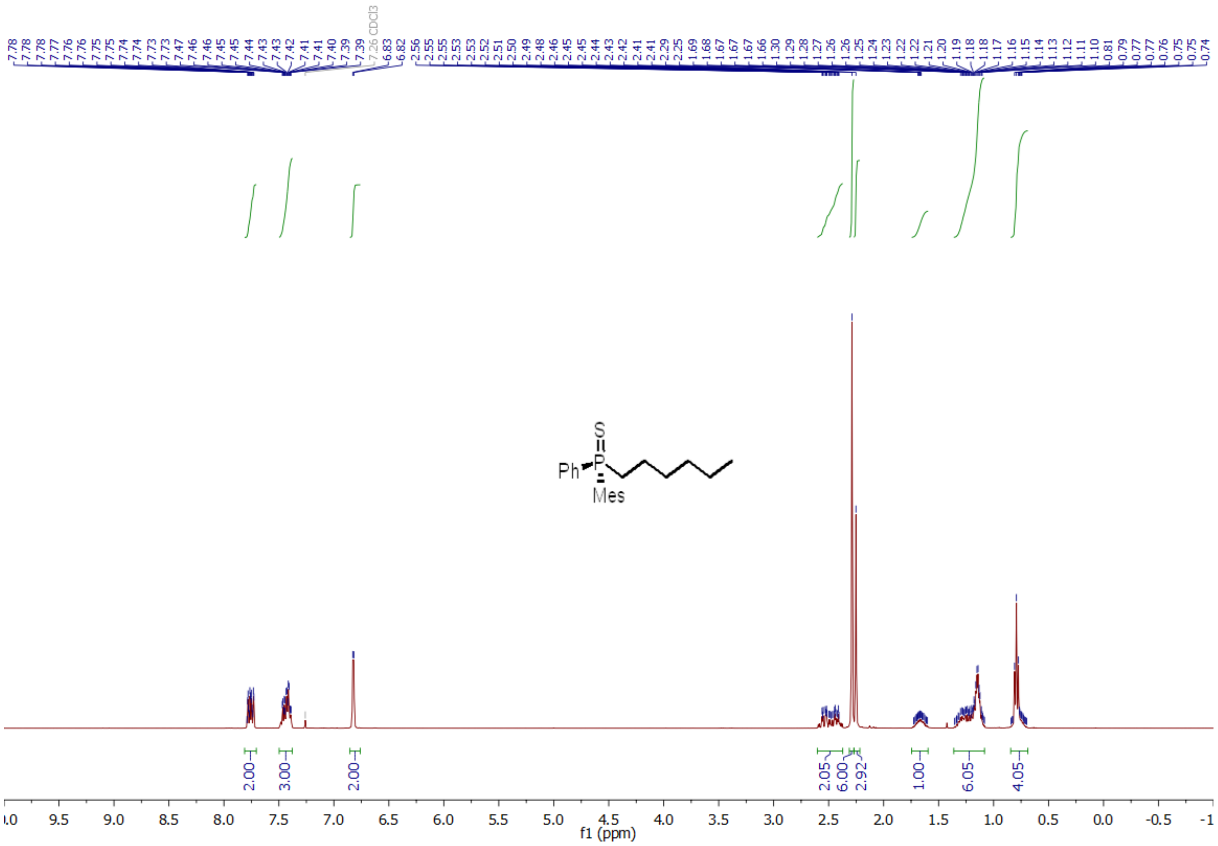

^{13}C NMR (101 MHz, $CDCl_3$)spectrum of **3’a**

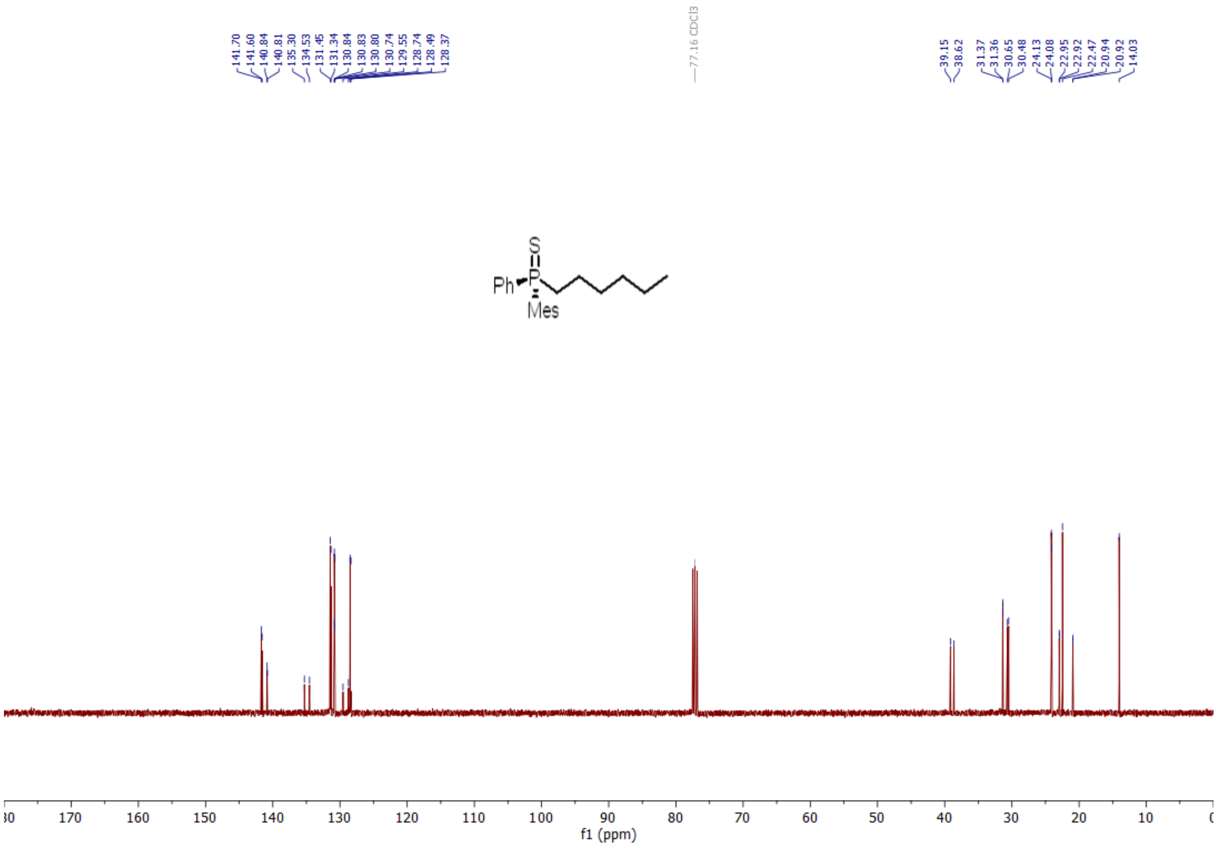

^{31}P NMR (162 MHz, $CDCl_3$) spectrum of **3'a**

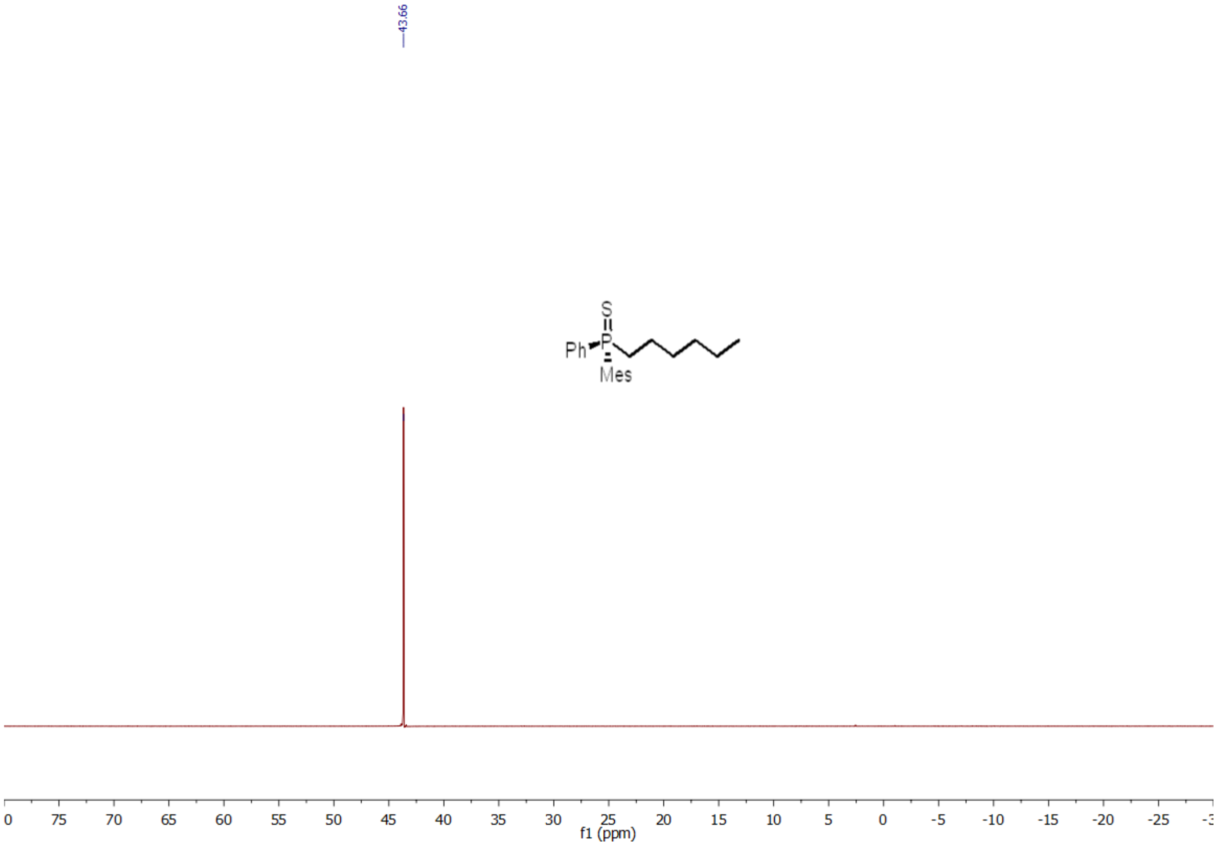

^{1}H NMR (400 MHz, $CDCl_3$) spectrum of **3'b**

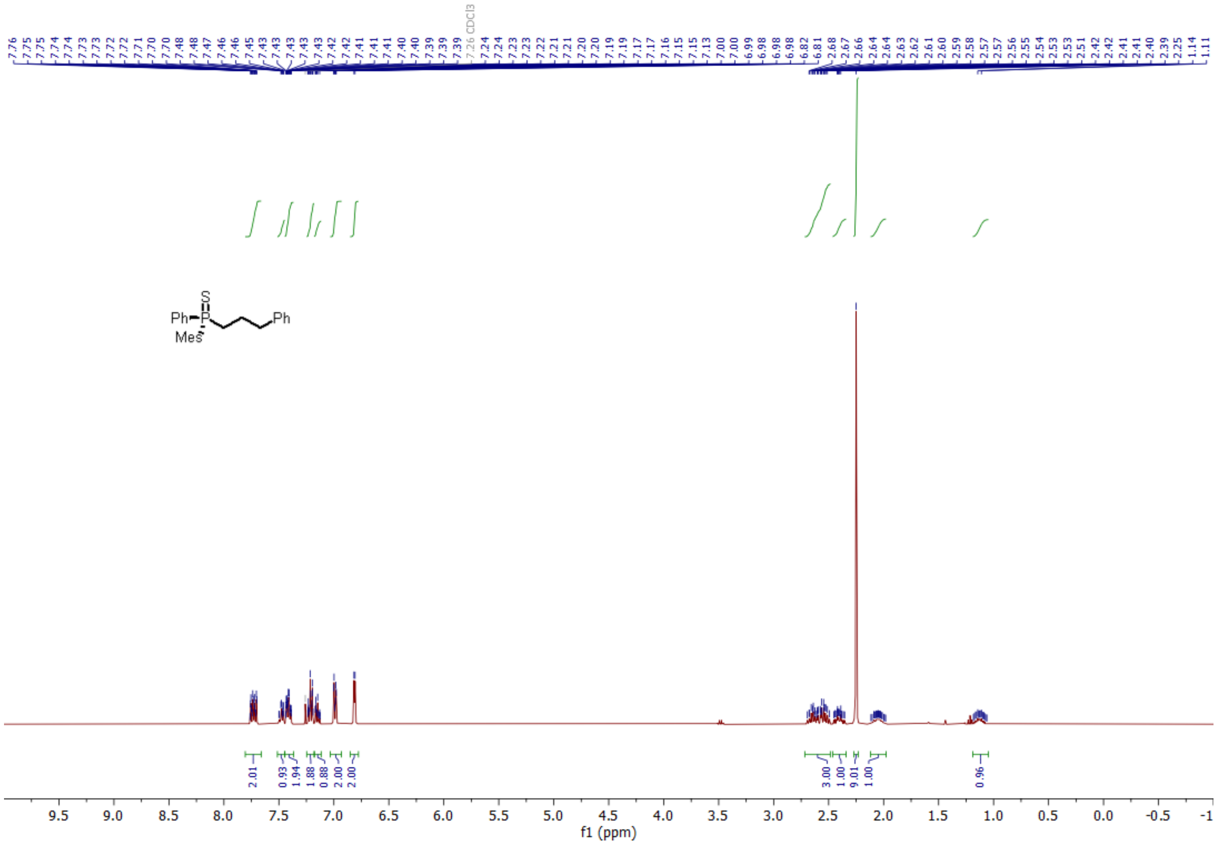

^{13}C NMR (101 MHz, CDCl$_3$)spectrum of **3’b**

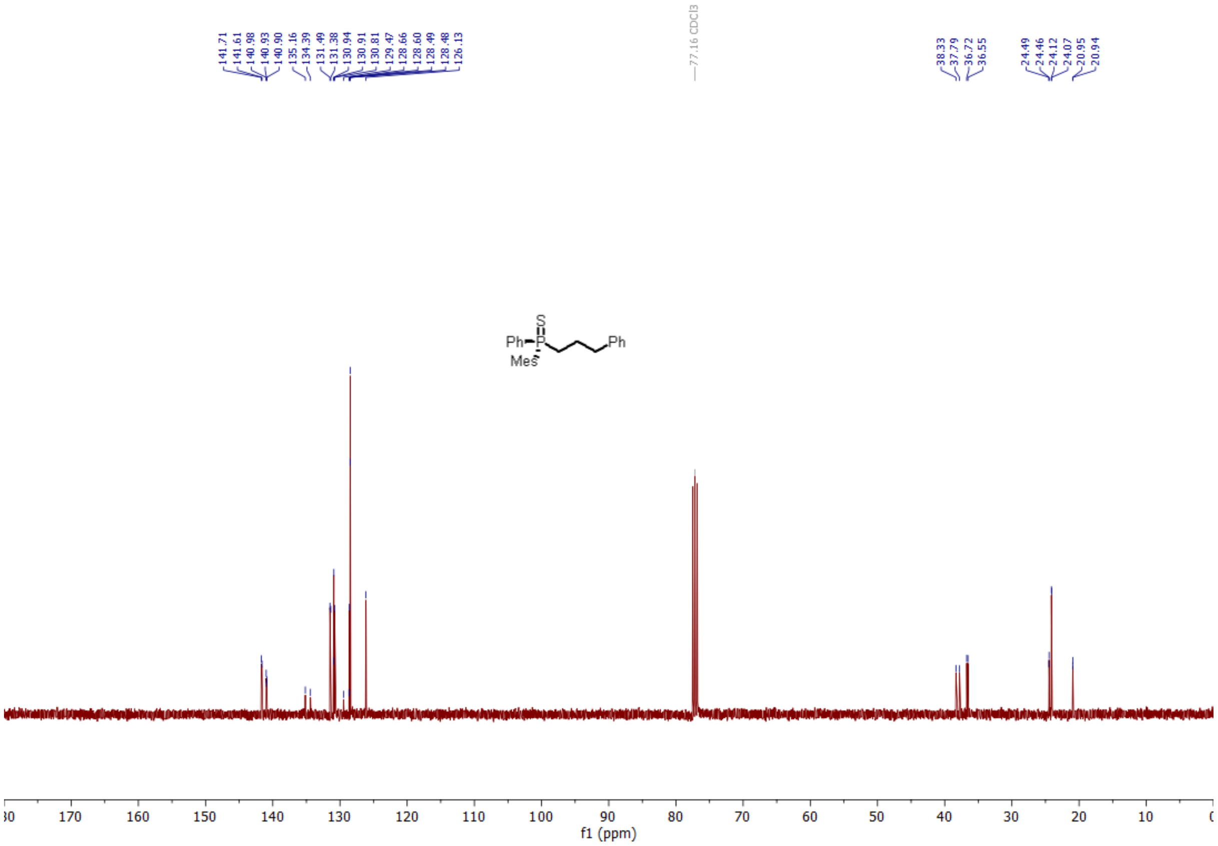

^{31}P NMR (162 MHz, CDCl$_3$) spectrum of **3’b**

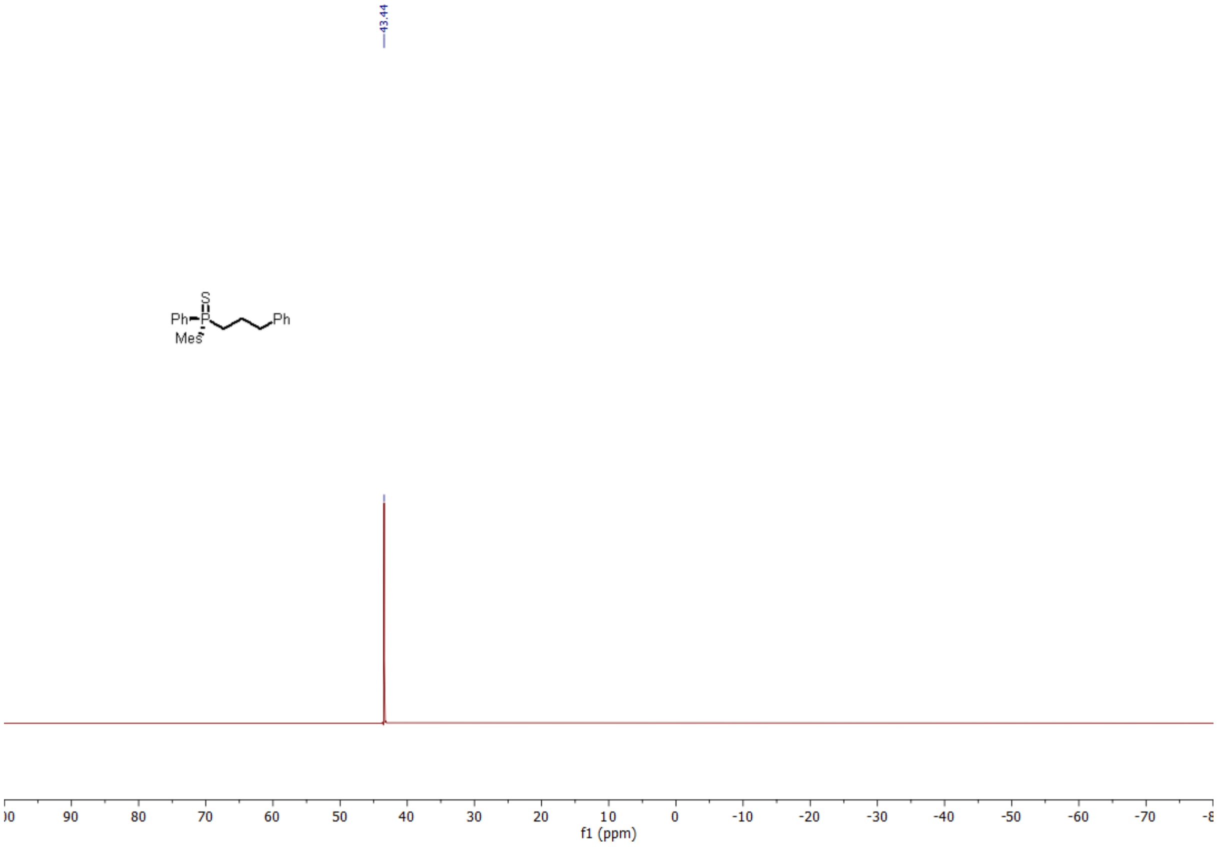

^{1}H NMR (400 MHz, $CDCl_3$) spectrum of **3’c**

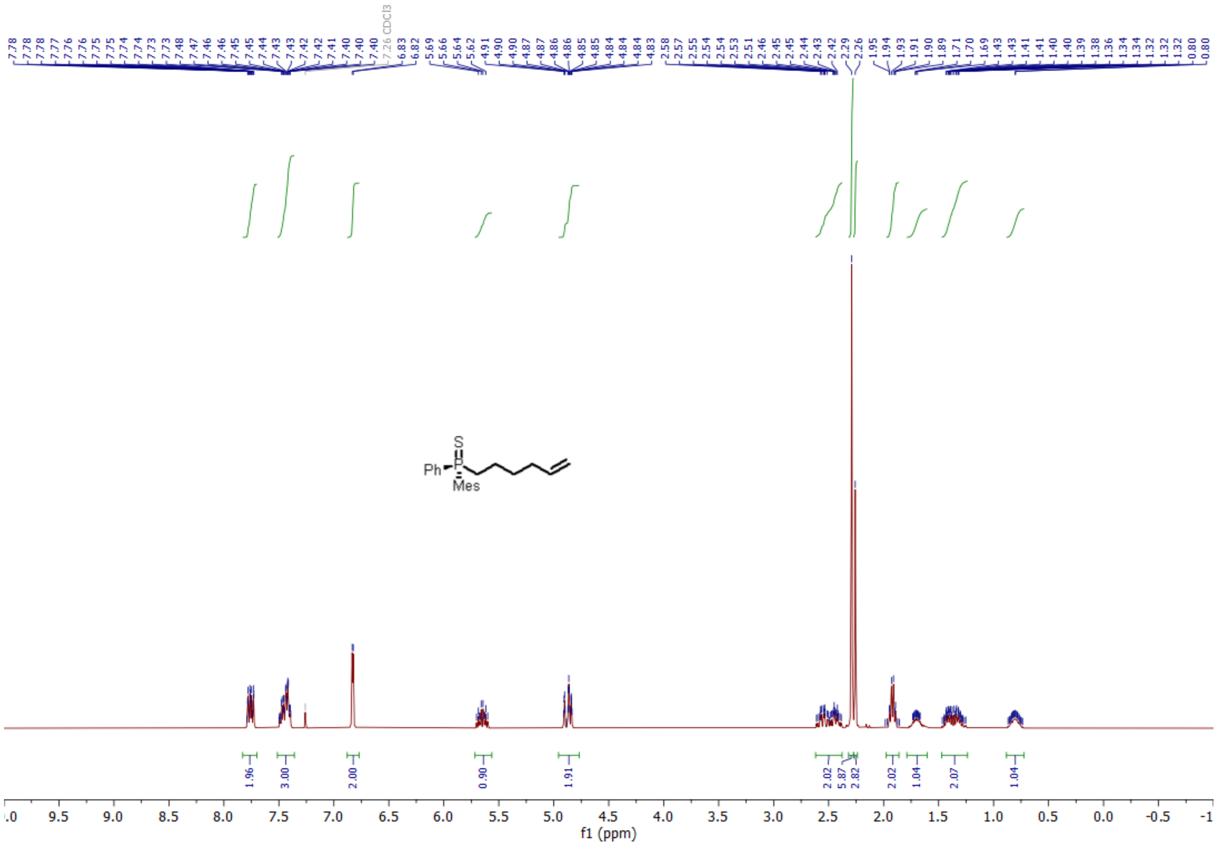

^{13}C NMR (101 MHz, $CDCl_3$)spectrum of **3’c**

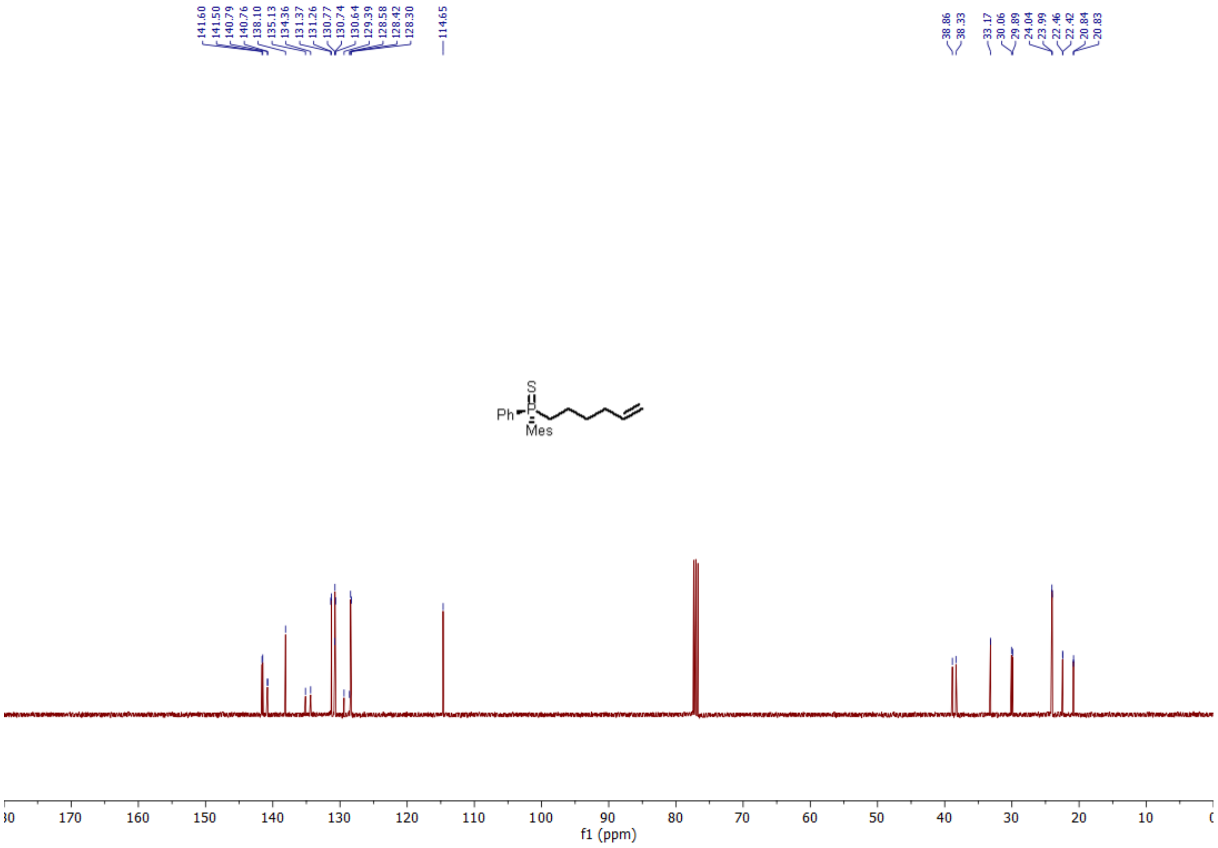

^{31}P NMR (162 MHz, $CDCl_3$) spectrum of **3’c**

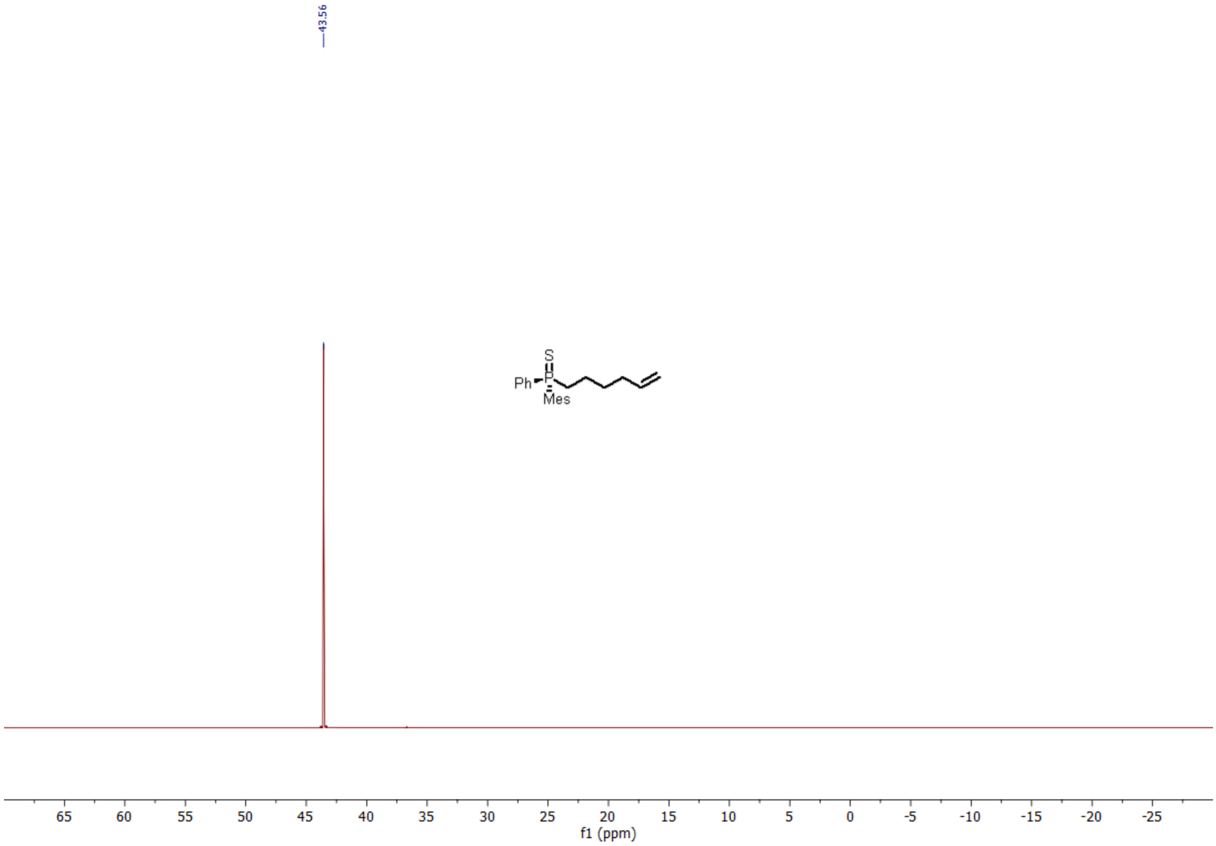

^{1}H NMR (400 MHz, $CDCl_3$) spectrum of **3’d**

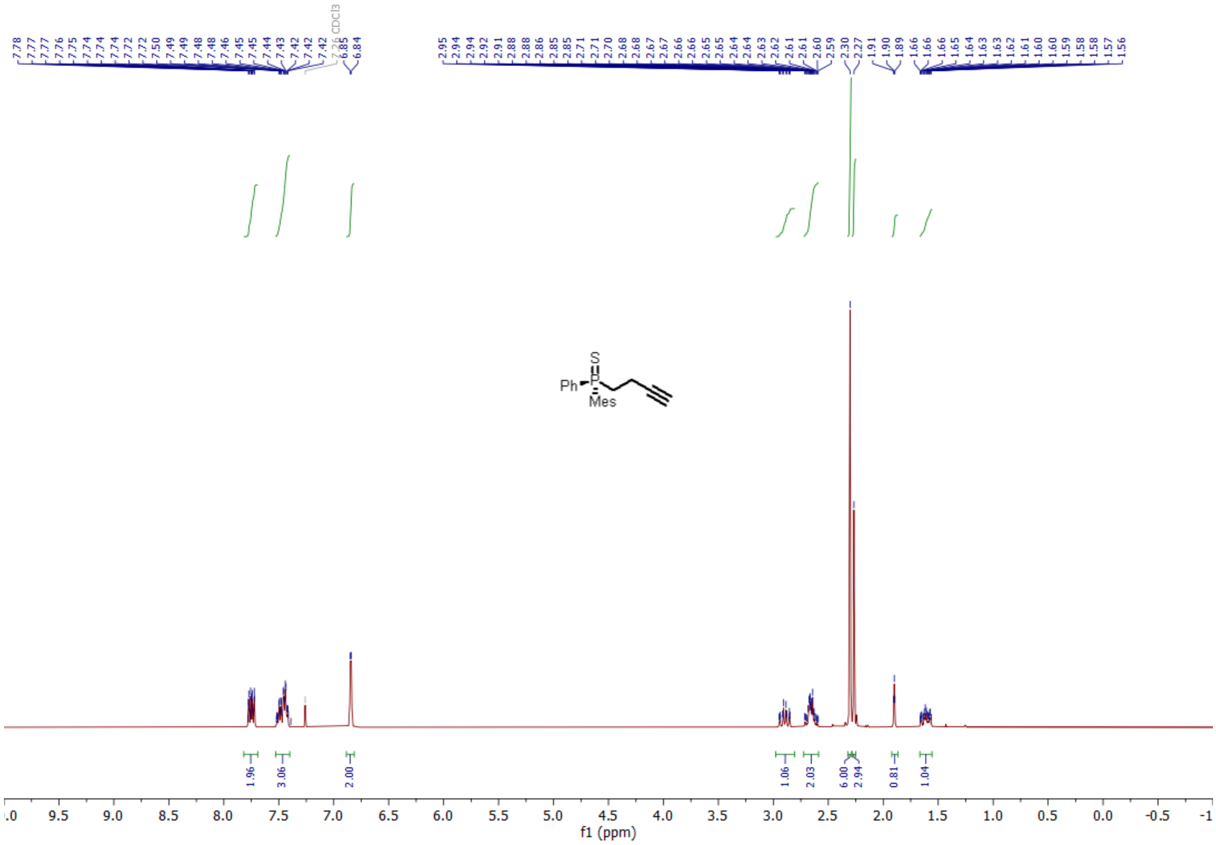

^{13}C NMR (101 MHz, $CDCl_3$)spectrum of **3’d**

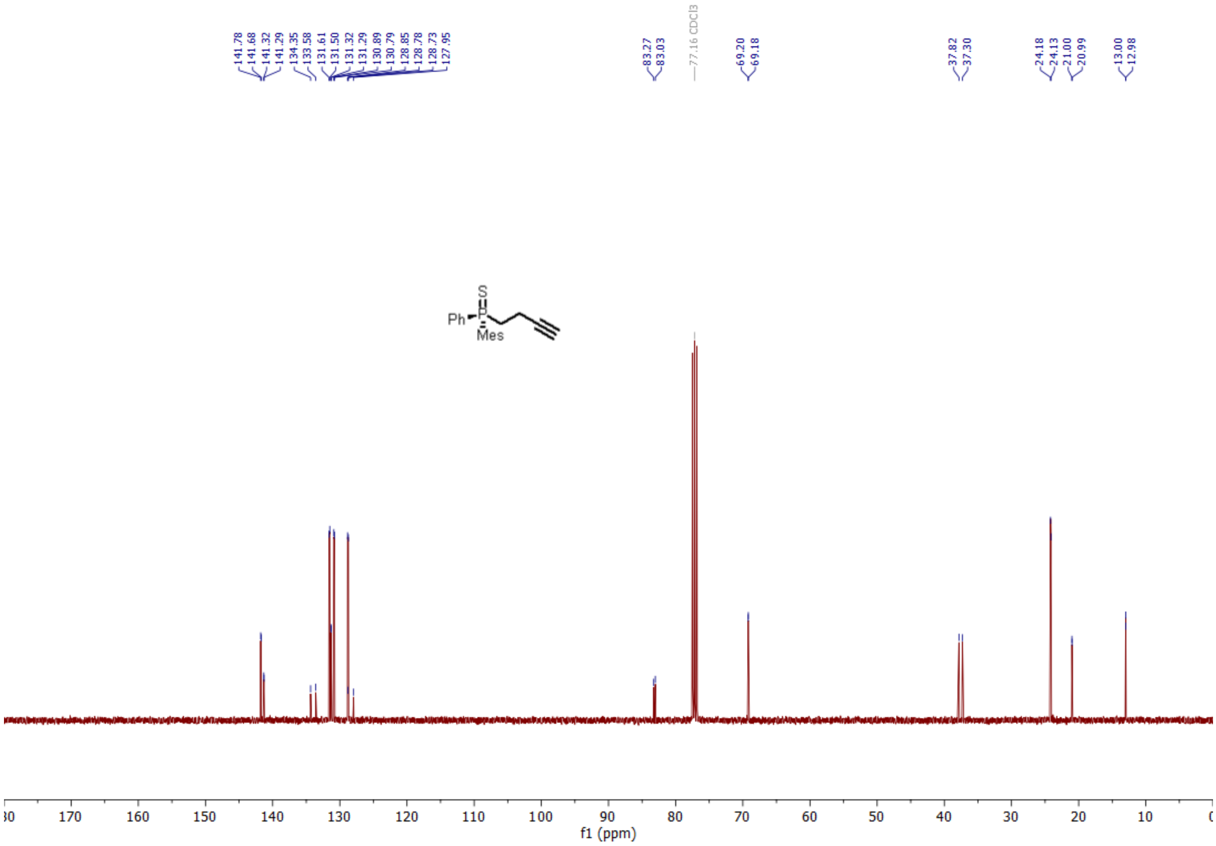

^{31}P NMR (162 MHz, $CDCl_3$) spectrum of **3’d**

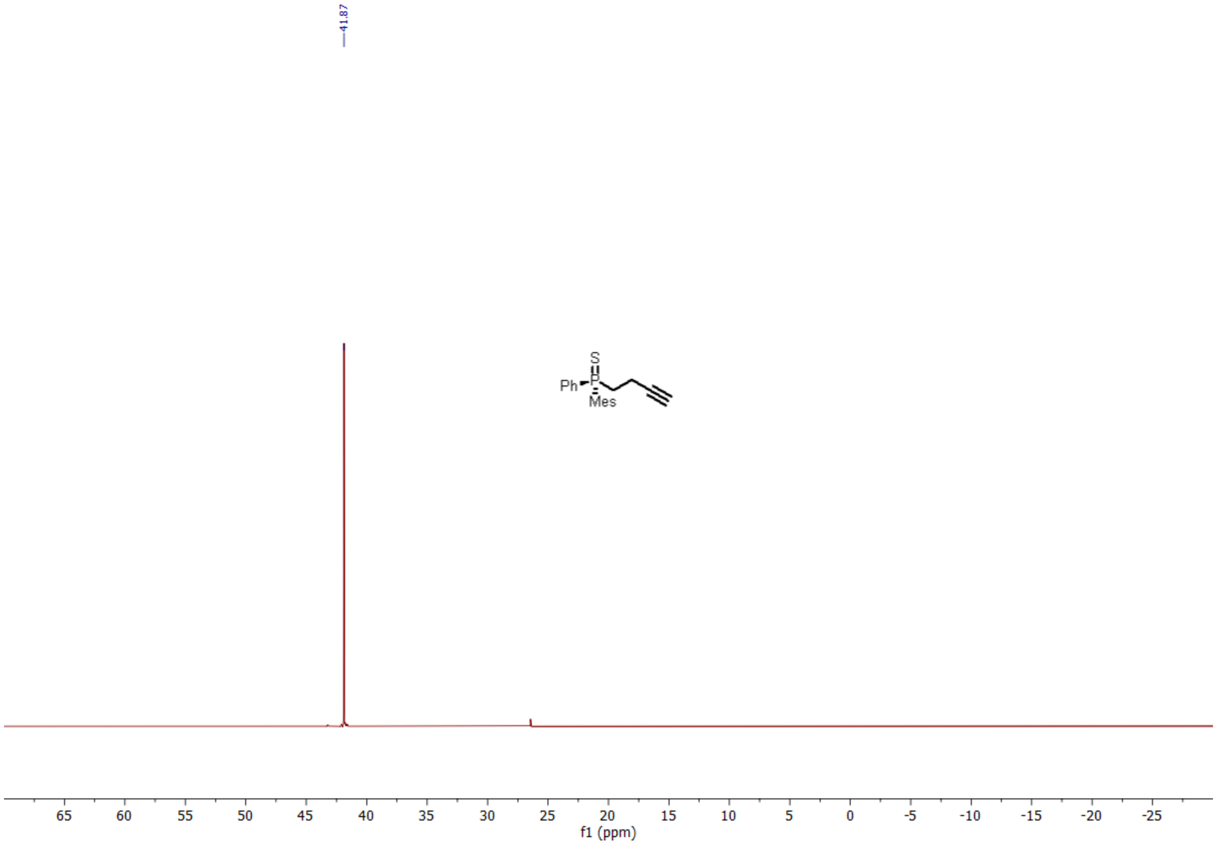

^{1}**H NMR** (400 MHz, $CDCl_3$) spectrum of **3’e**

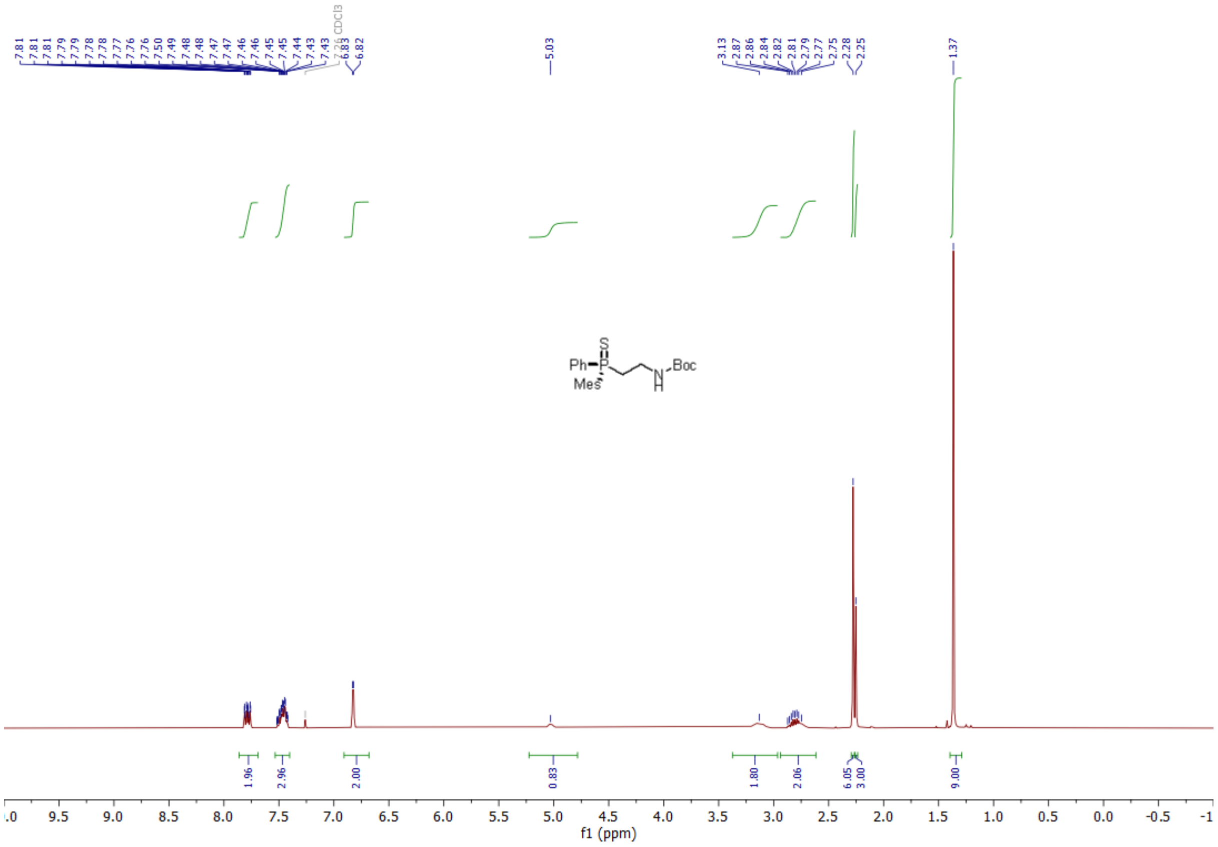

^{13}**C NMR** (101 MHz, $CDCl_3$)spectrum of **3’e**

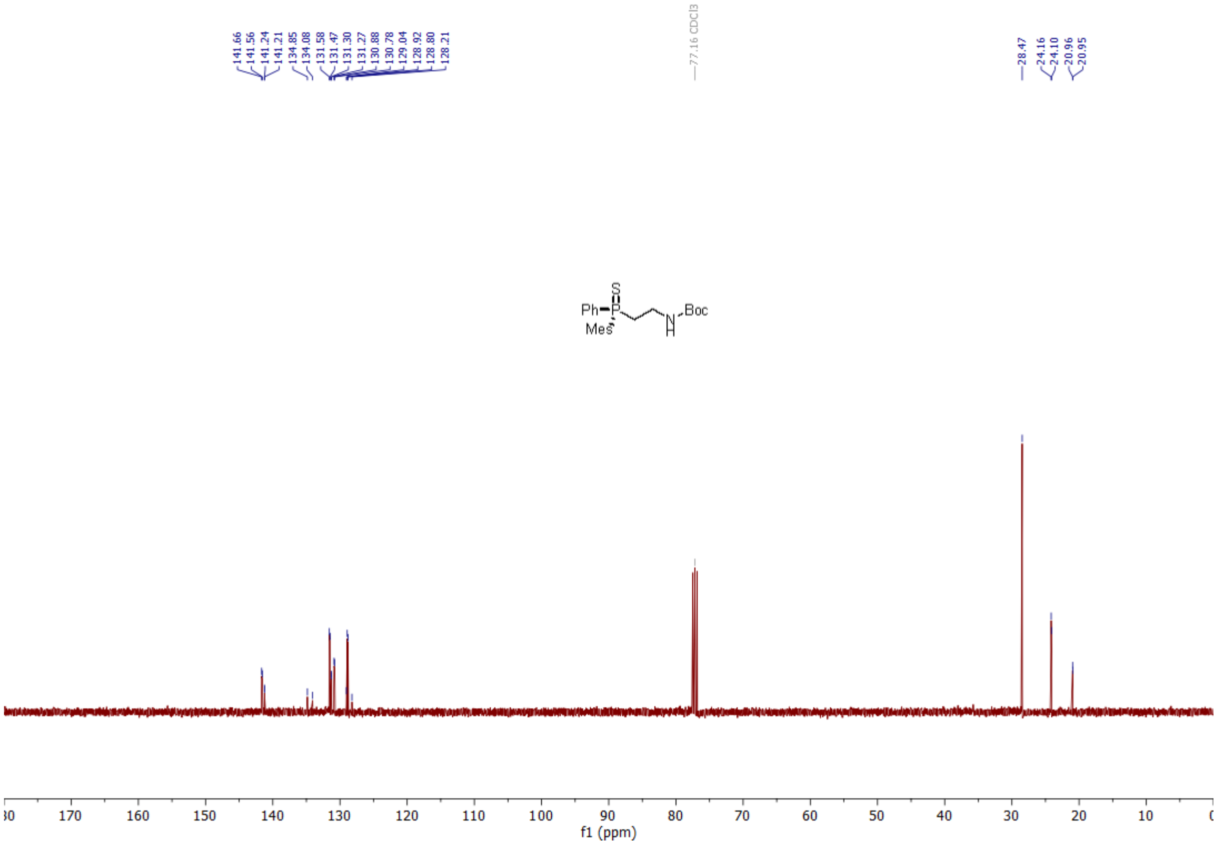

^{31}**P NMR** (162 MHz, $CDCl_3$) spectrum of **3’e**

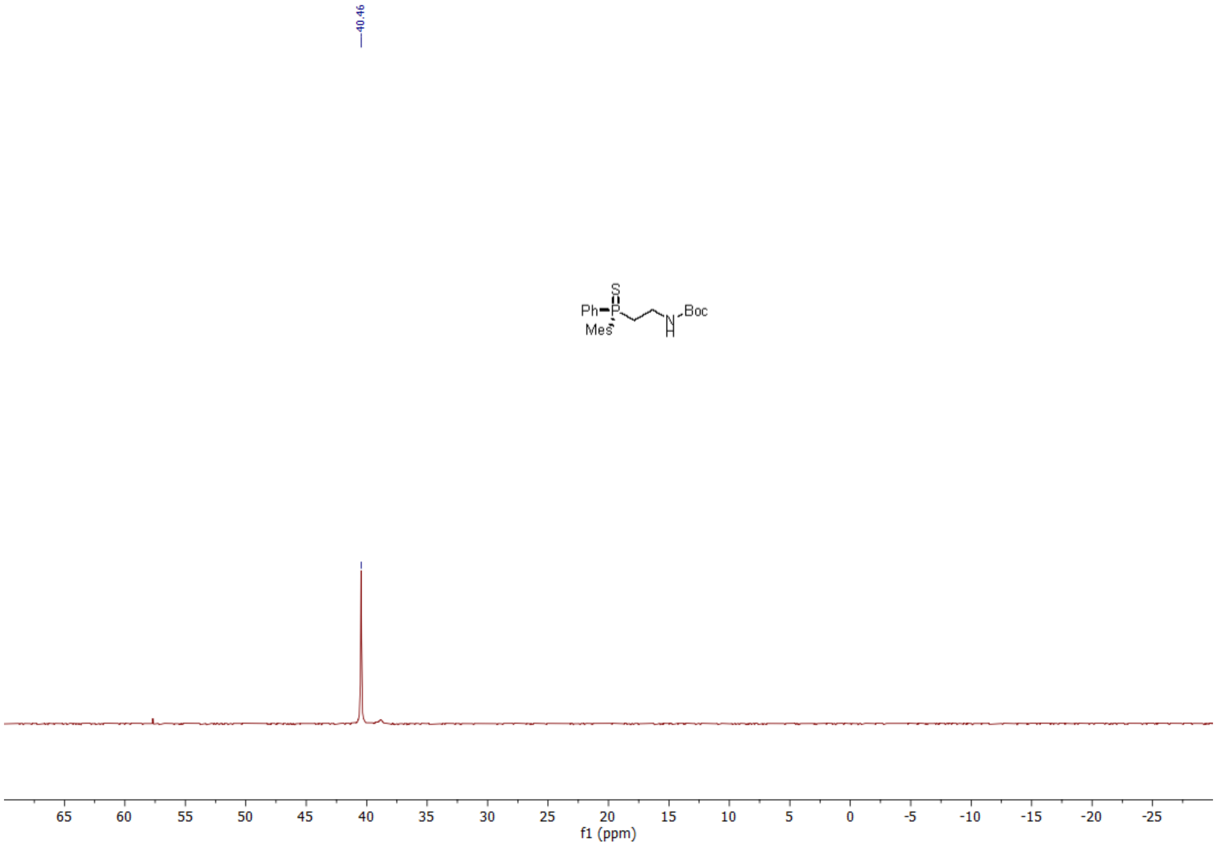

^{1}**H NMR** (400 MHz, $CDCl_3$) spectrum of **3’f**

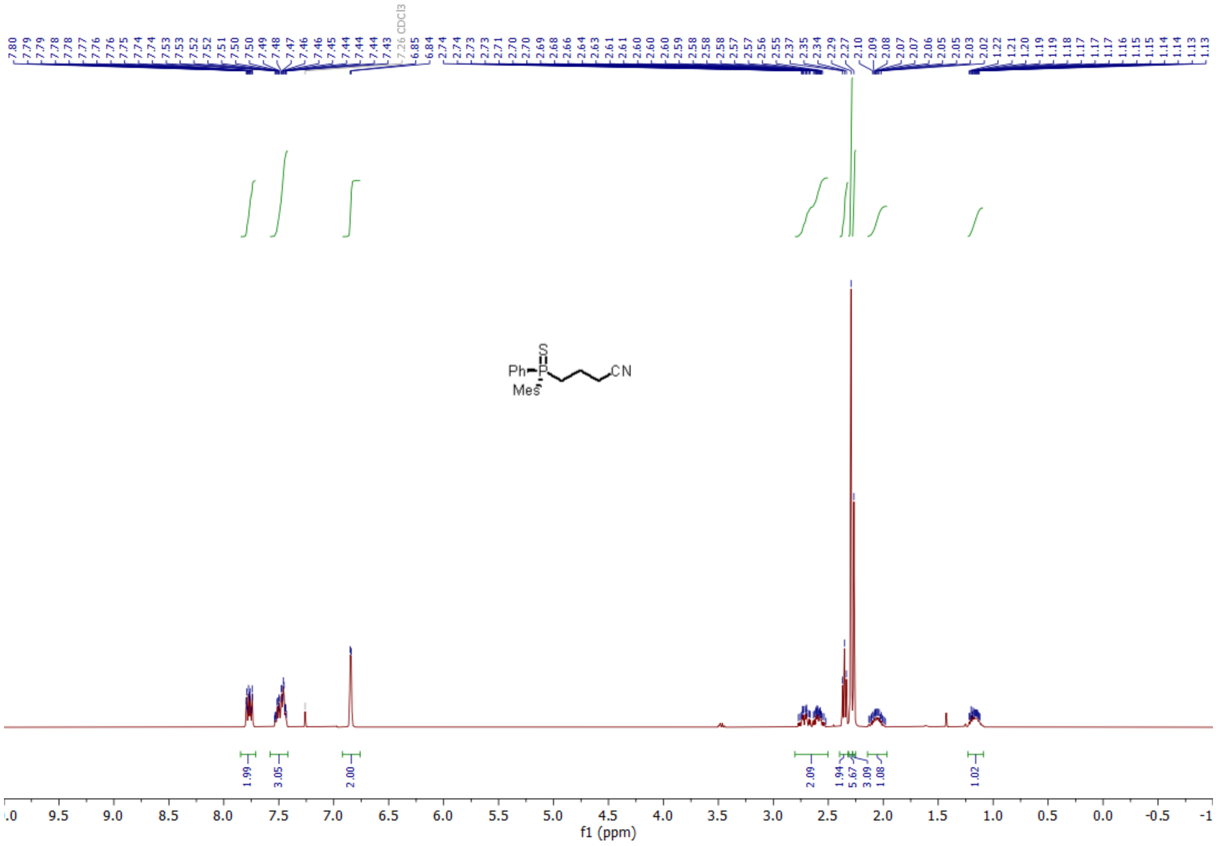

^{13}C NMR (101 MHz, $CDCl_3$)spectrum of **3’f**

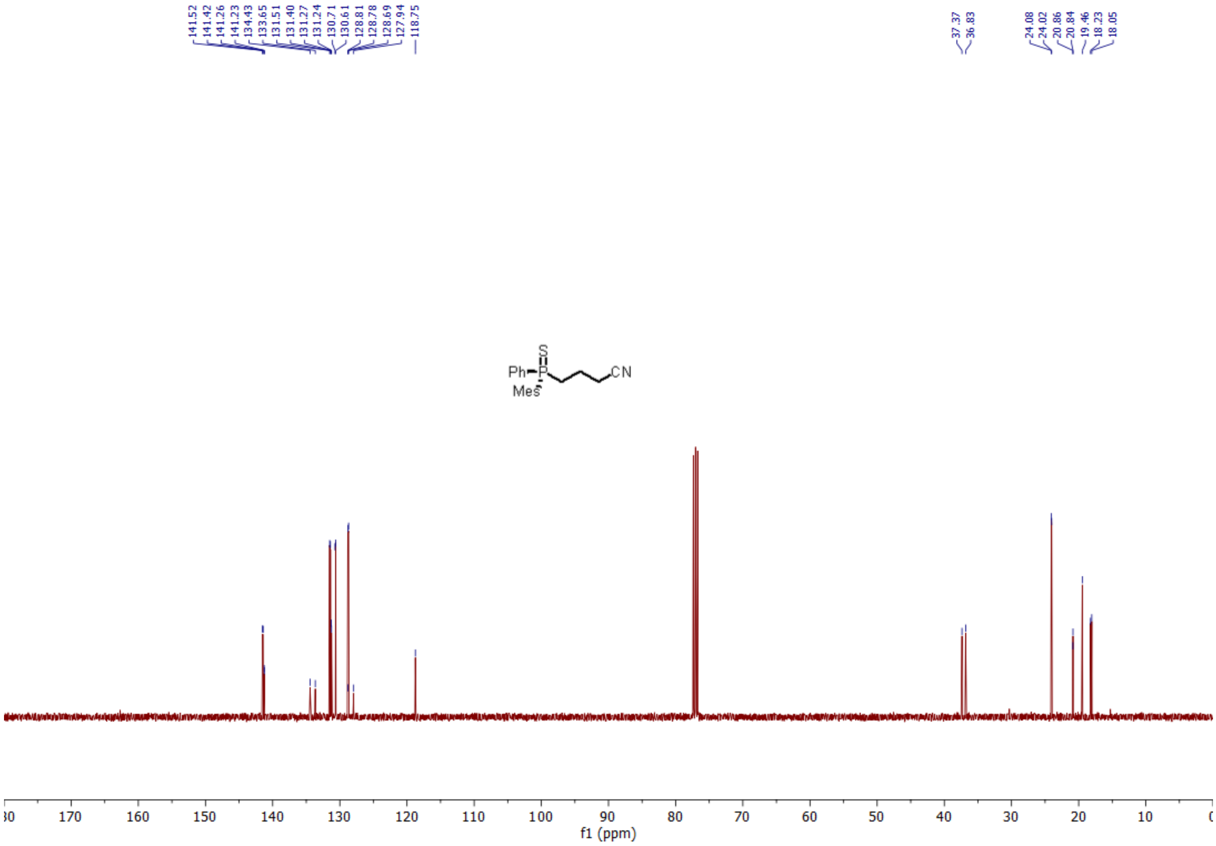

^{31}P NMR (162 MHz, $CDCl_3$) spectrum of **3’f**

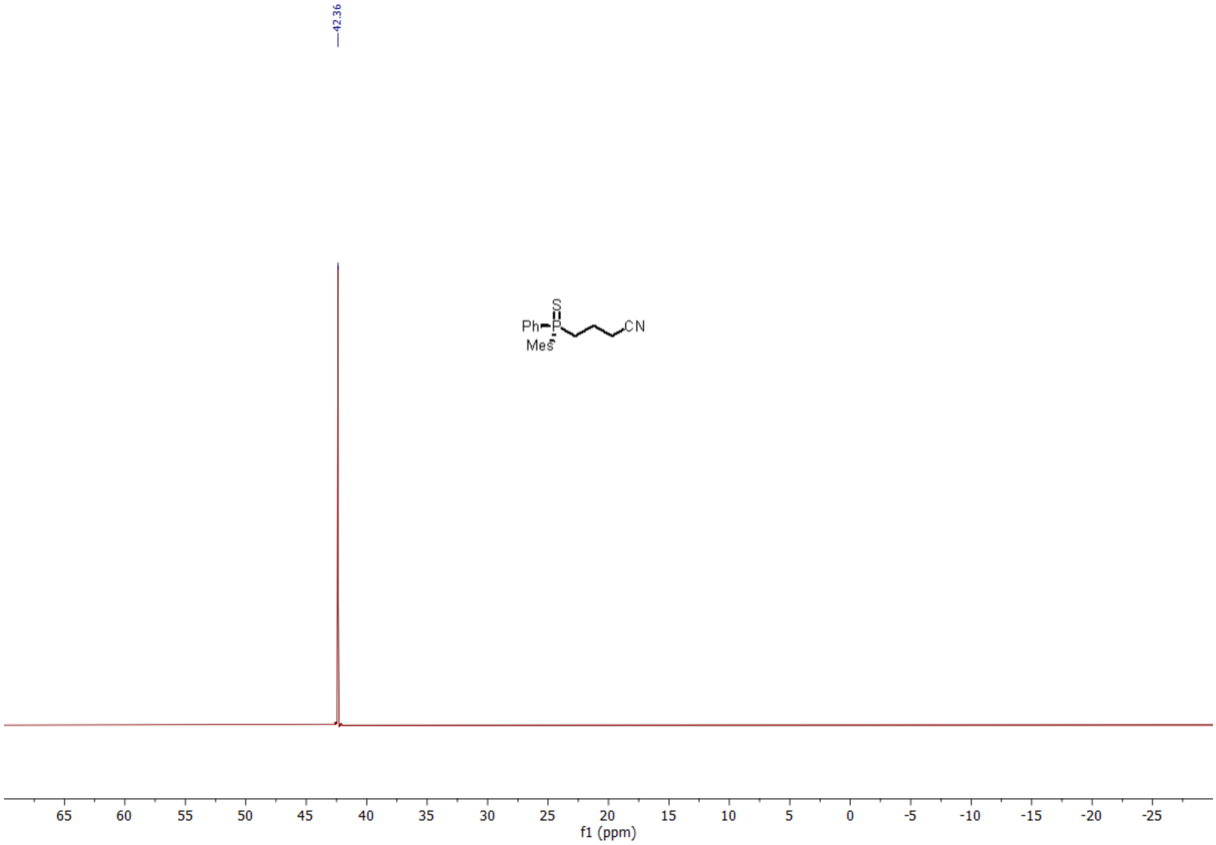

^{1}H NMR (400 MHz, $CDCl_3$) spectrum of **3'g**

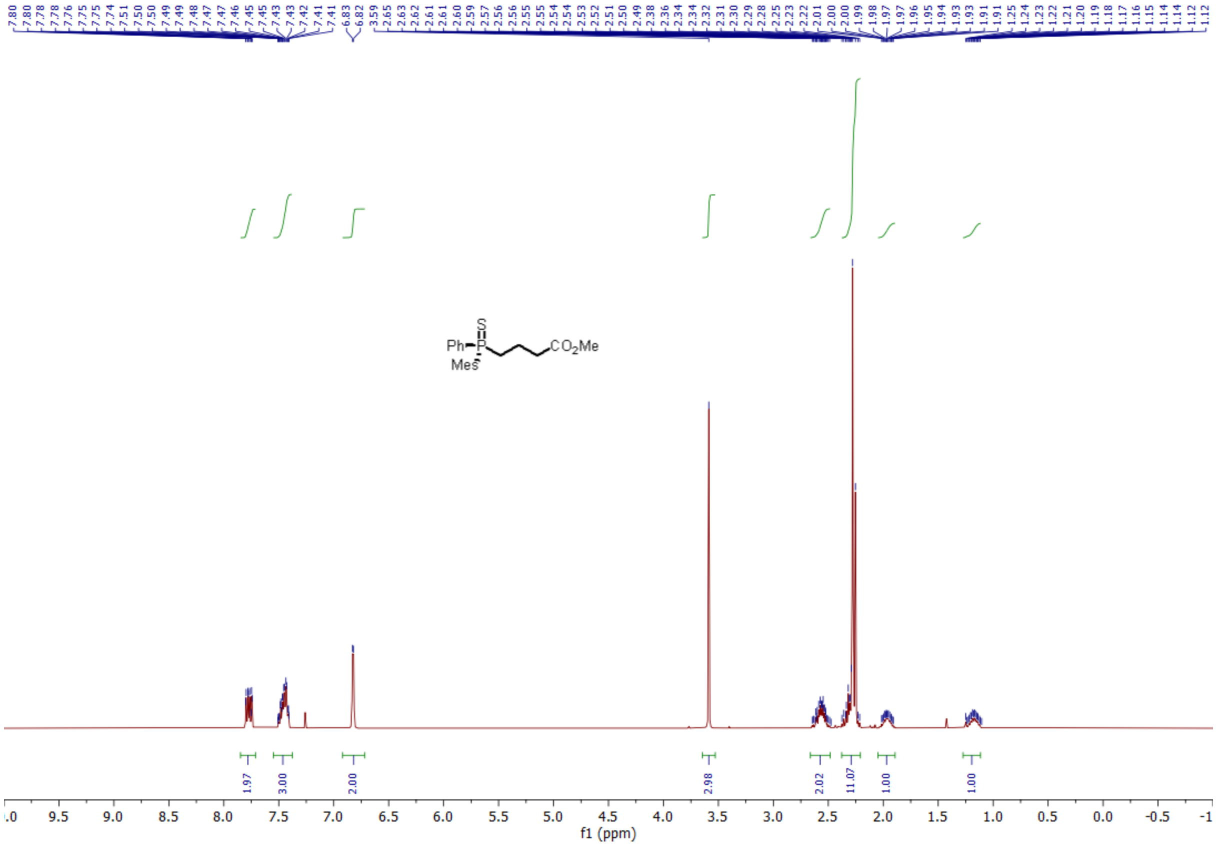

^{13}C NMR (101 MHz, $CDCl_3$)spectrum of **3'g**

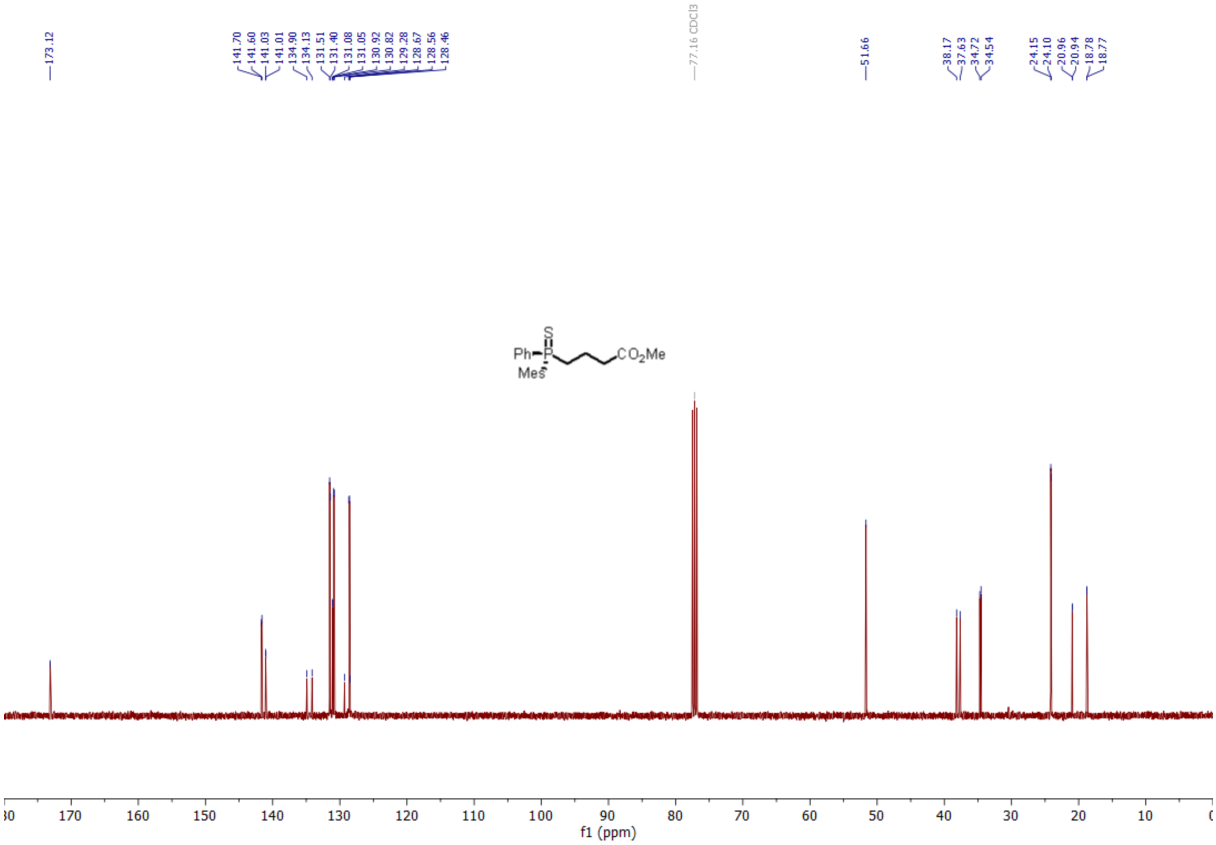

^{31}P NMR (162 MHz, $CDCl_3$) spectrum of **3’g**

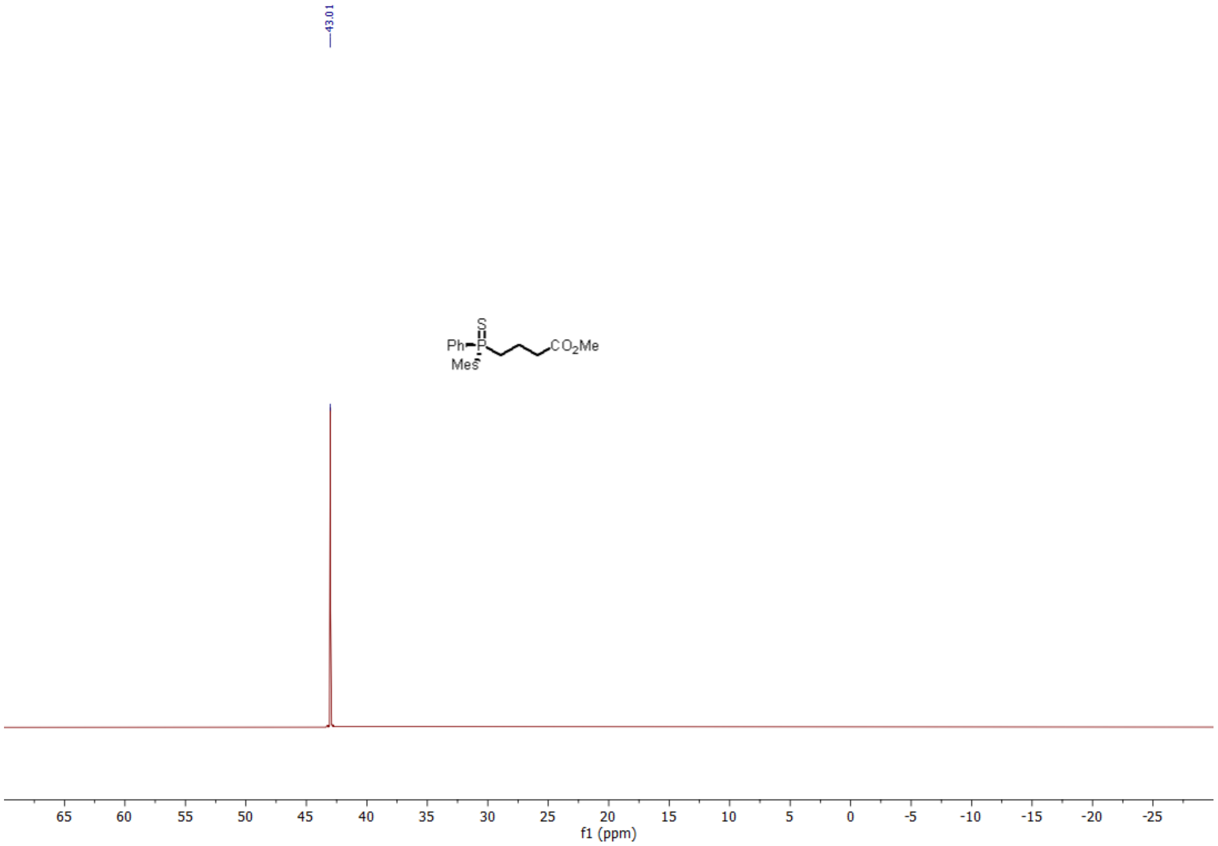

^{1}H NMR (400 MHz, $CDCl_3$) spectrum of **3’h**

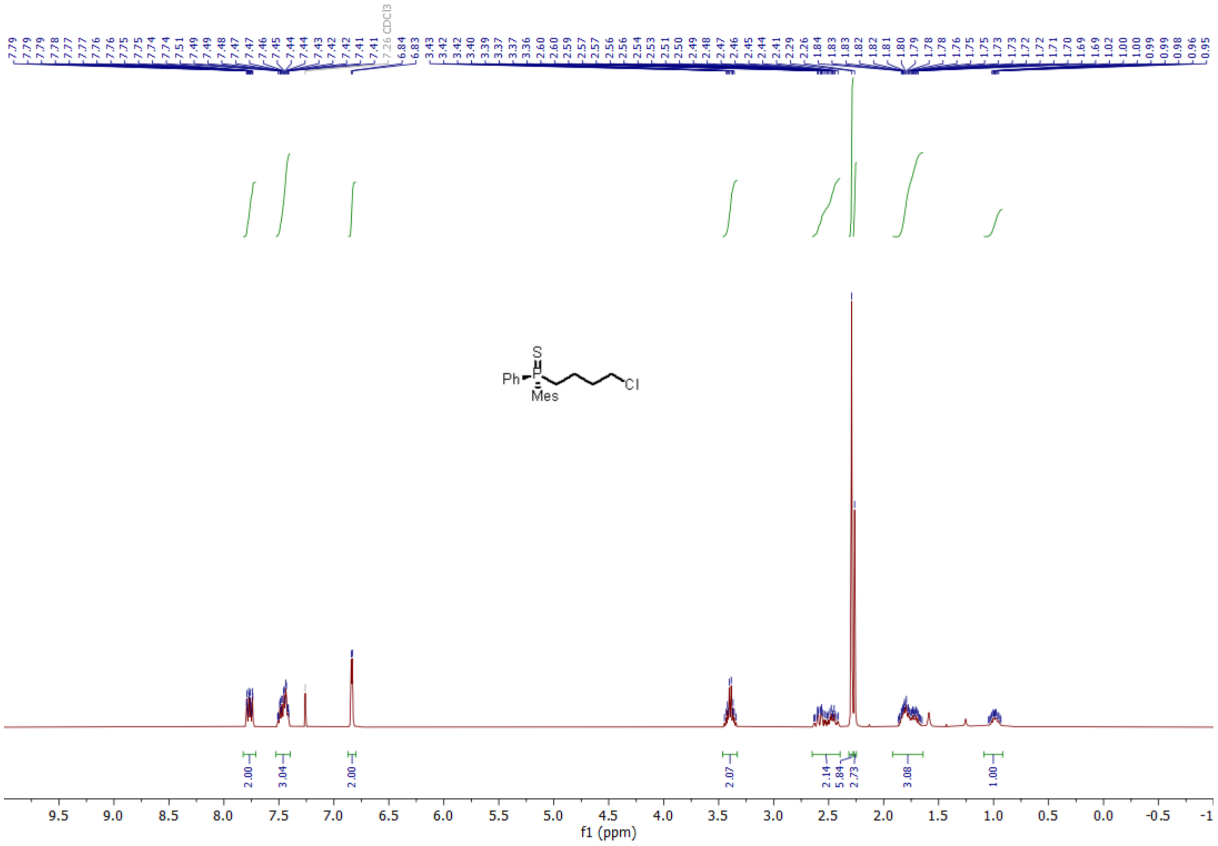

^{13}C NMR (101 MHz, $CDCl_3$)spectrum of **3’h**

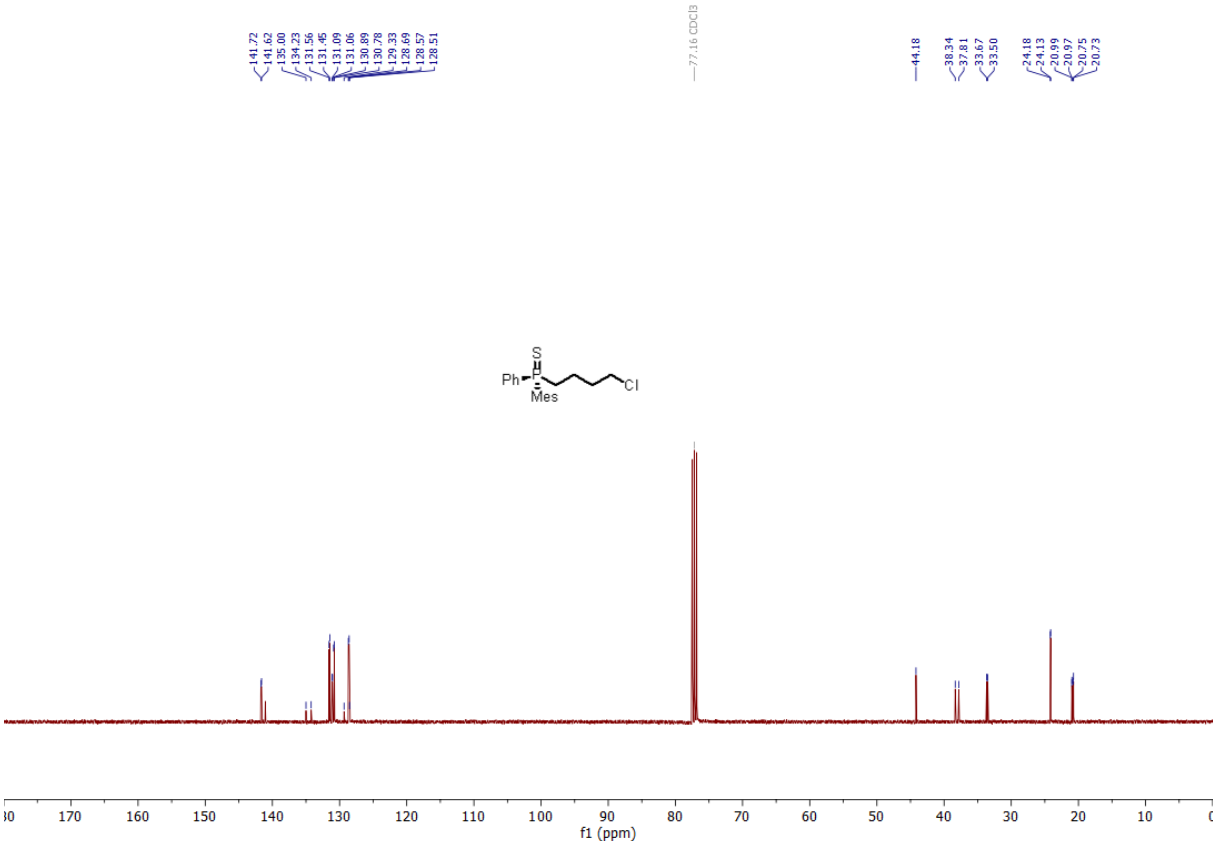

^{31}P NMR (162 MHz, $CDCl_3$) spectrum of **3’h**

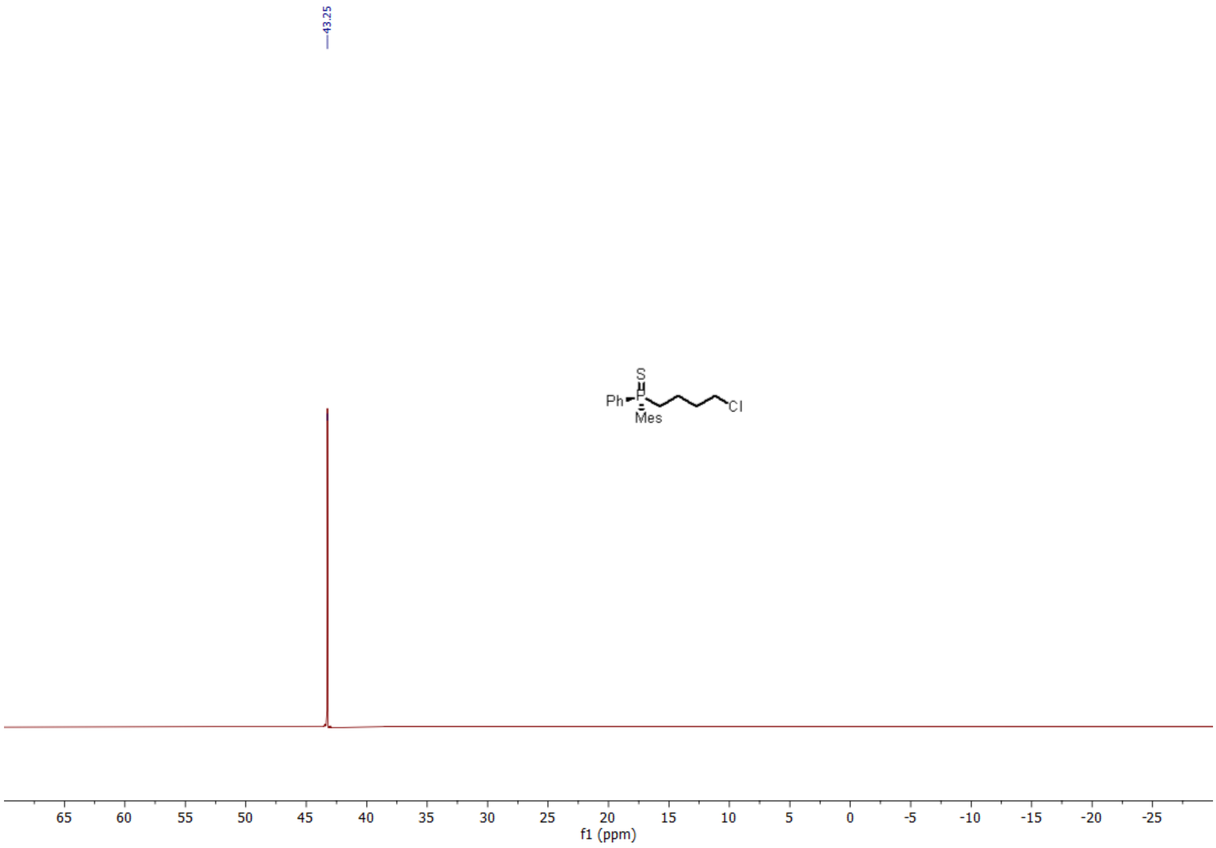

^{1}**H NMR** (400 MHz, $CDCl_3$) spectrum of **4’a**

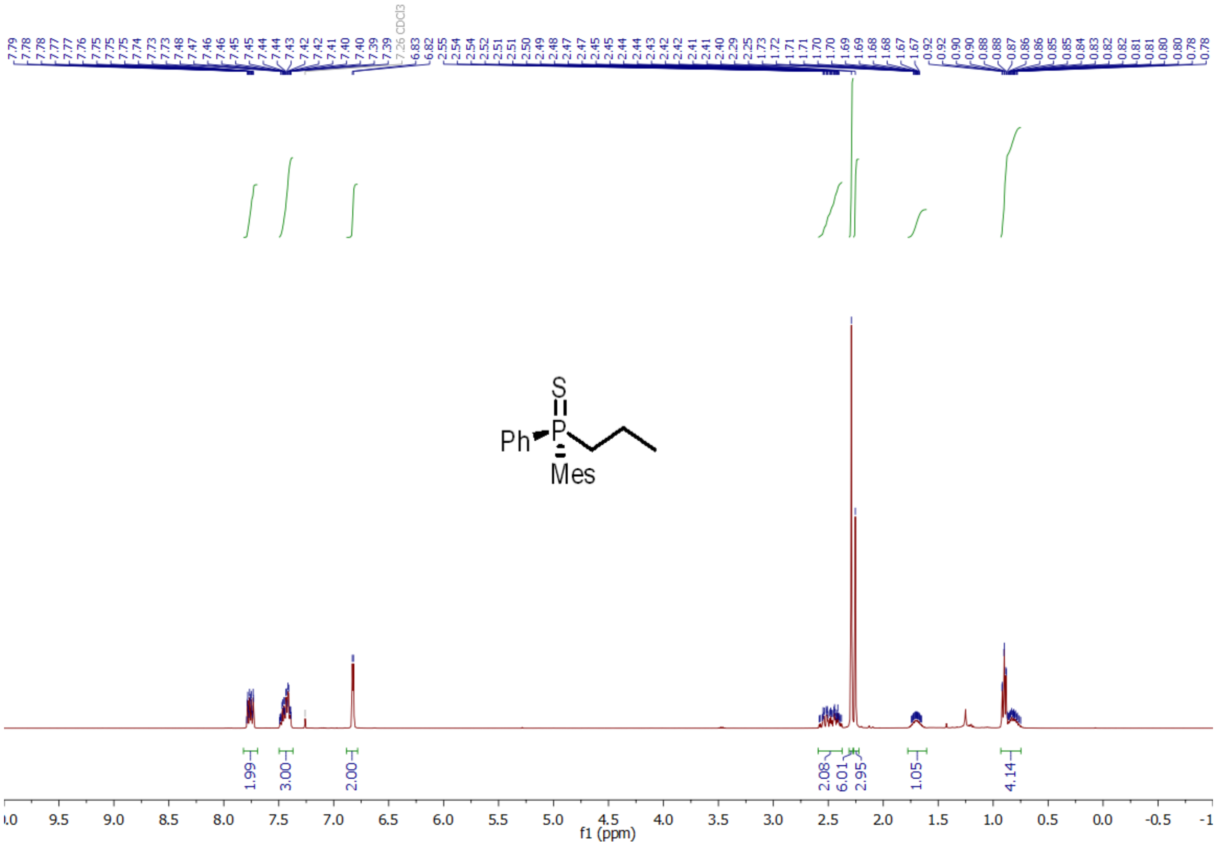

^{13}**C NMR** (101 MHz, $CDCl_3$)spectrum of **4’a**

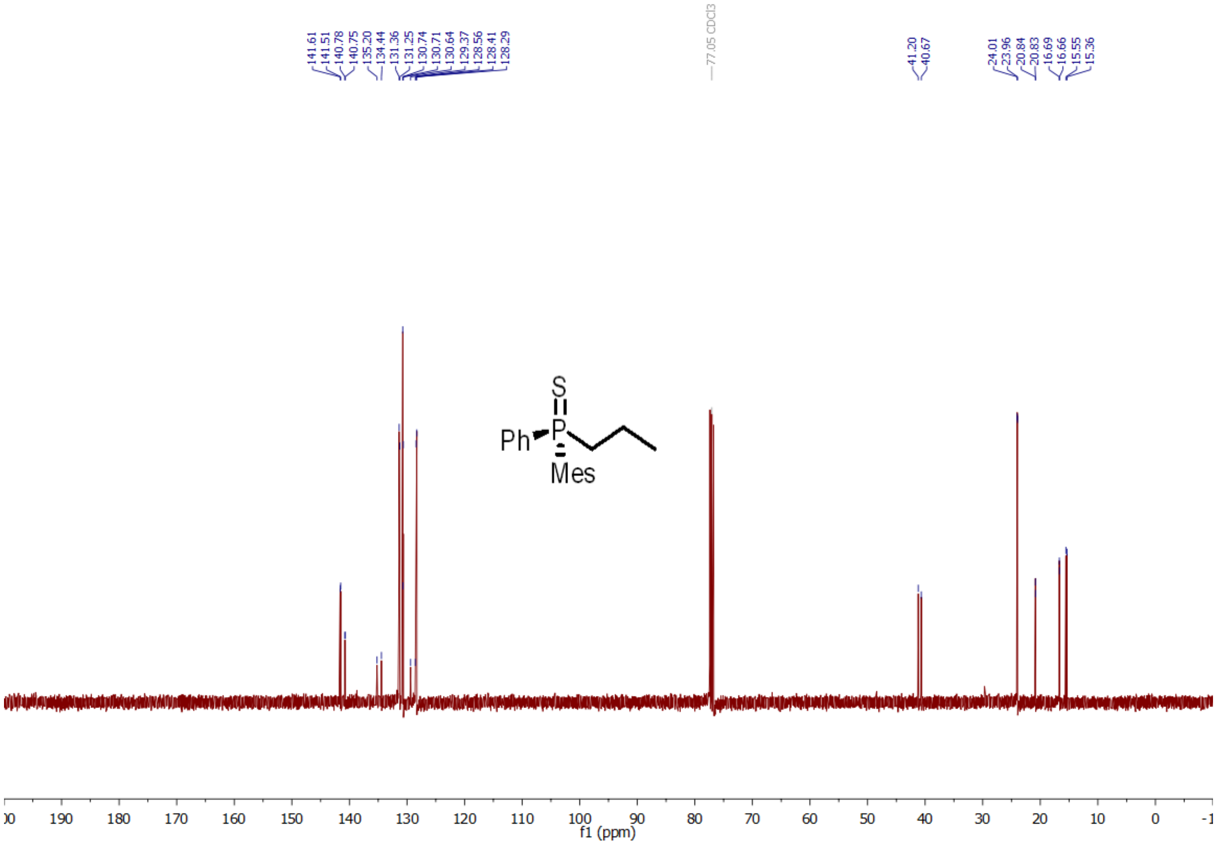

^{31}**P NMR** (162 MHz, CDCl$_3$) spectrum of **4’a**

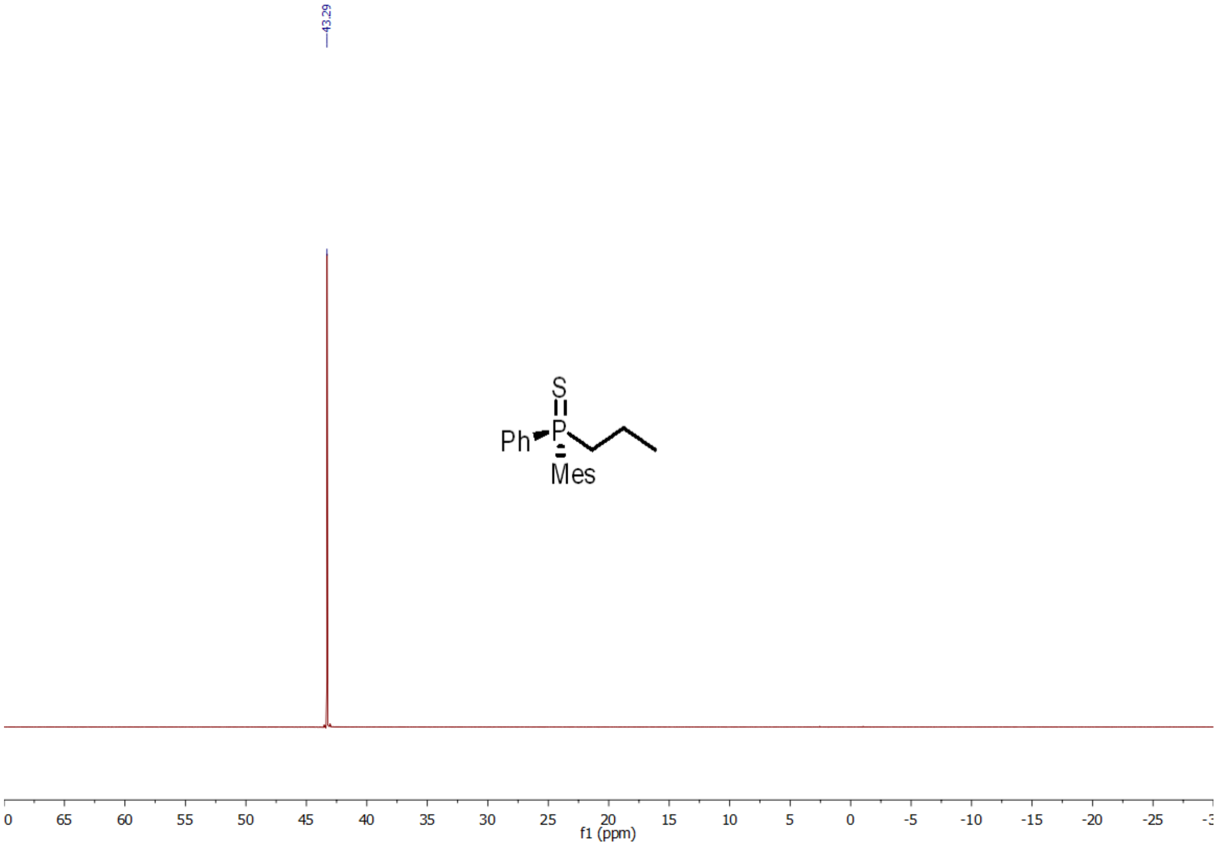

^{1}**H NMR** (400 MHz, CDCl$_3$) spectrum of **4’b**

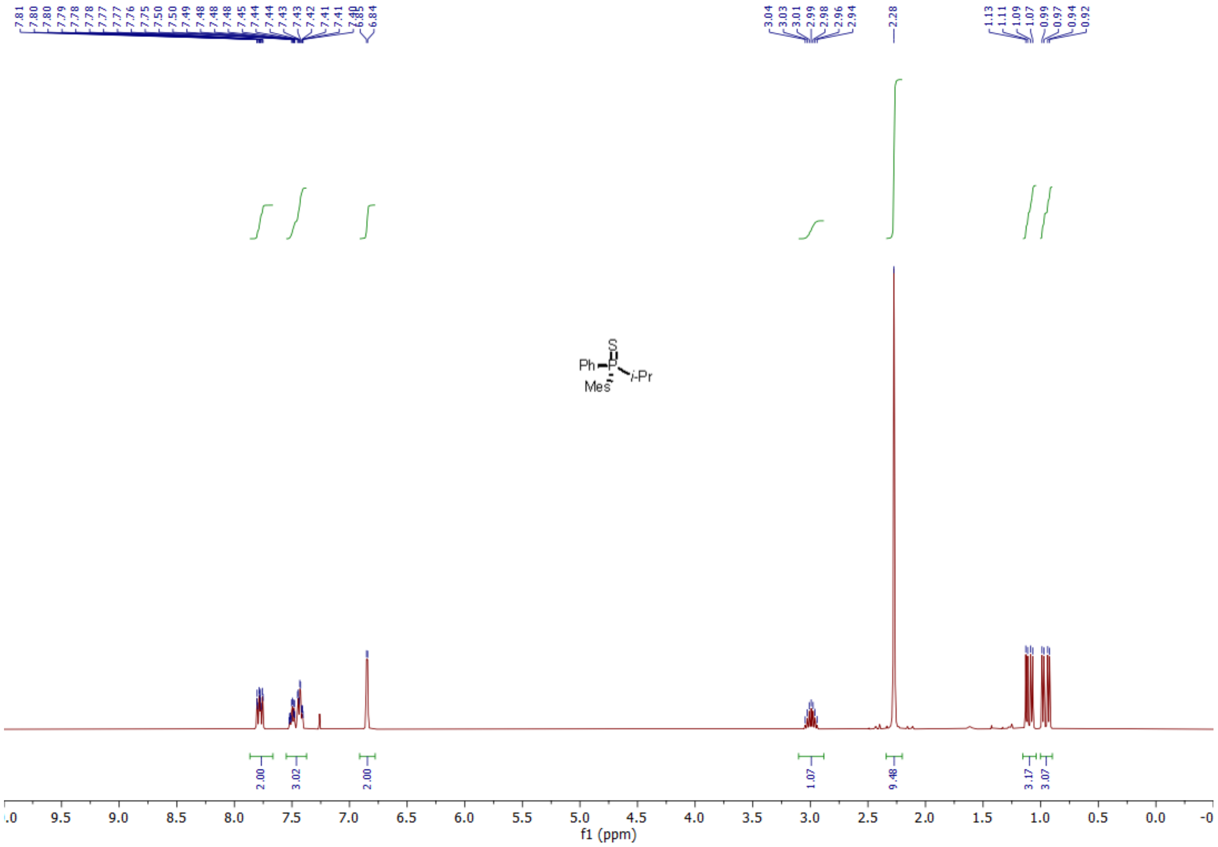

^{13}C NMR (101 MHz, $CDCl_3$)spectrum of **4’b**

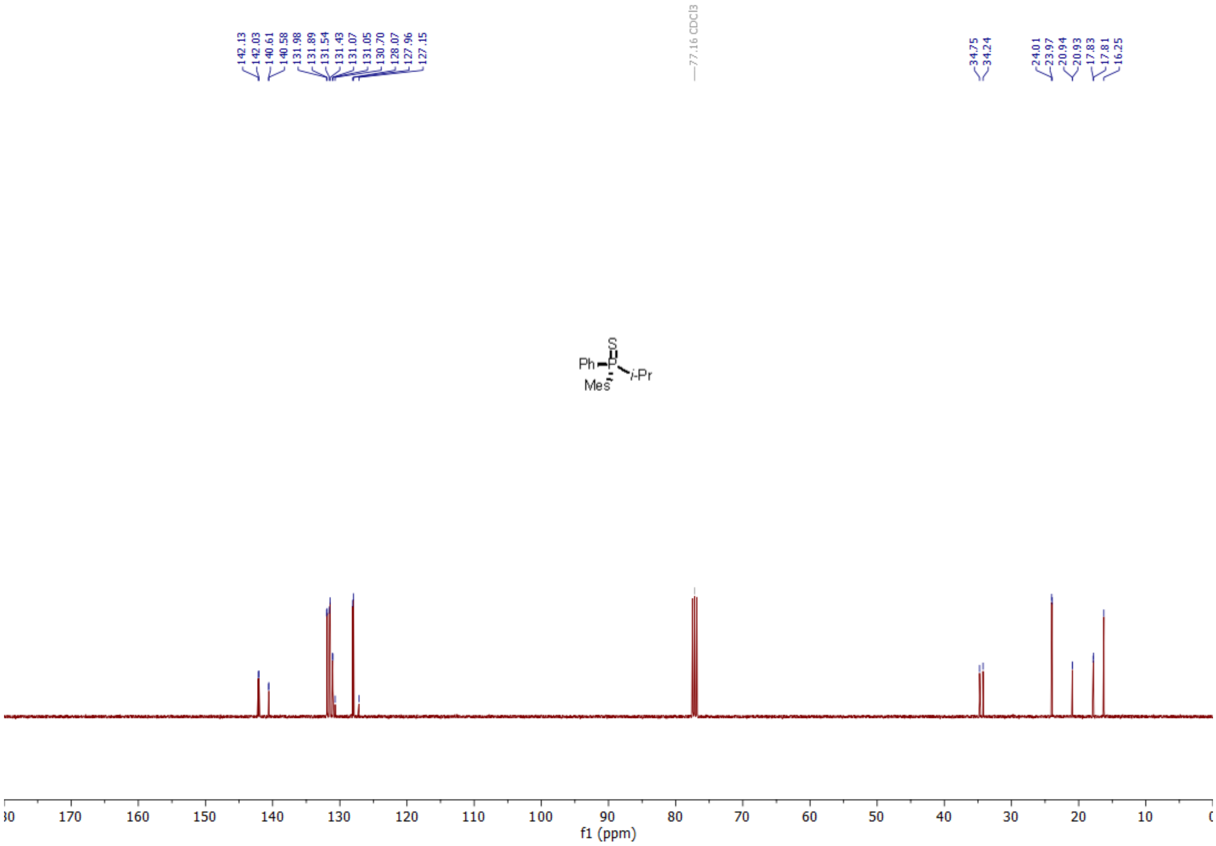

^{31}P NMR (162 MHz, $CDCl_3$) spectrum of **4’b**

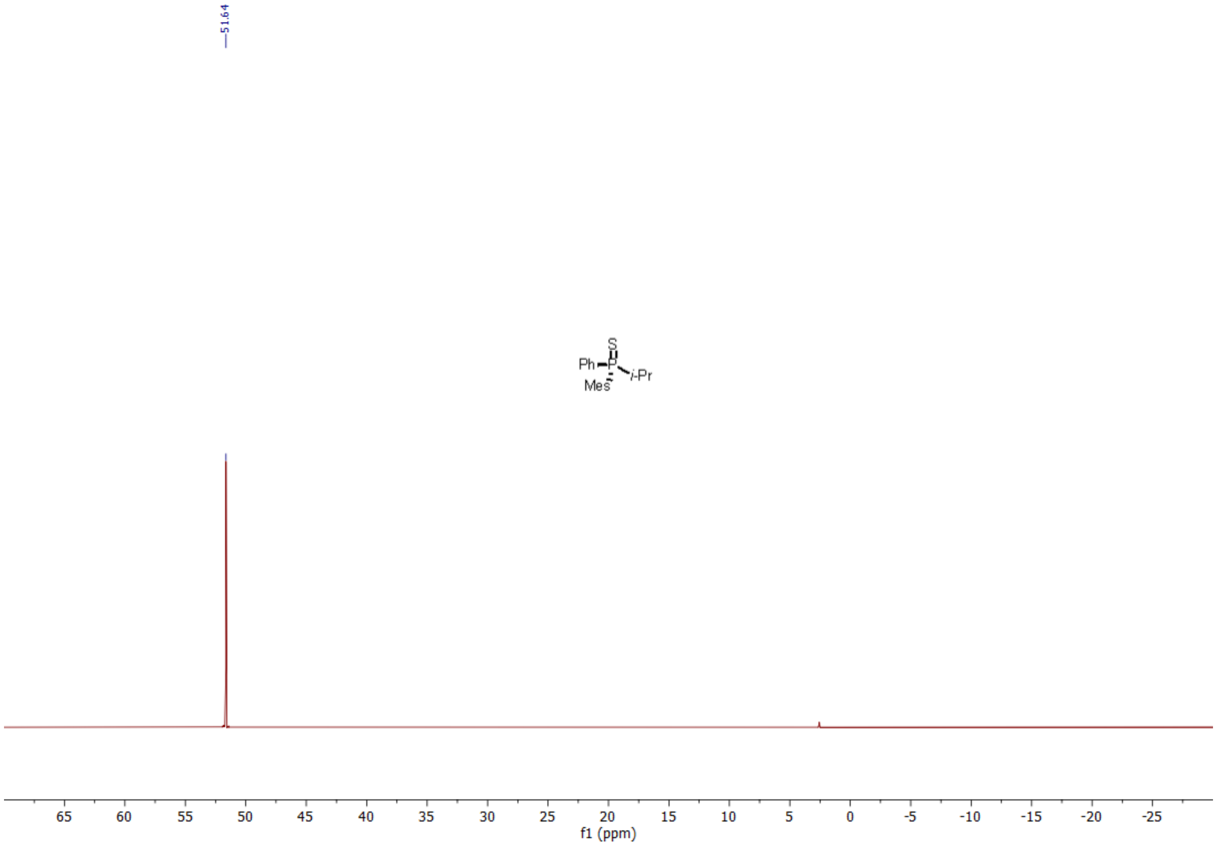

^{1}H NMR (400 MHz, $CDCl_3$) spectrum of **4’c**

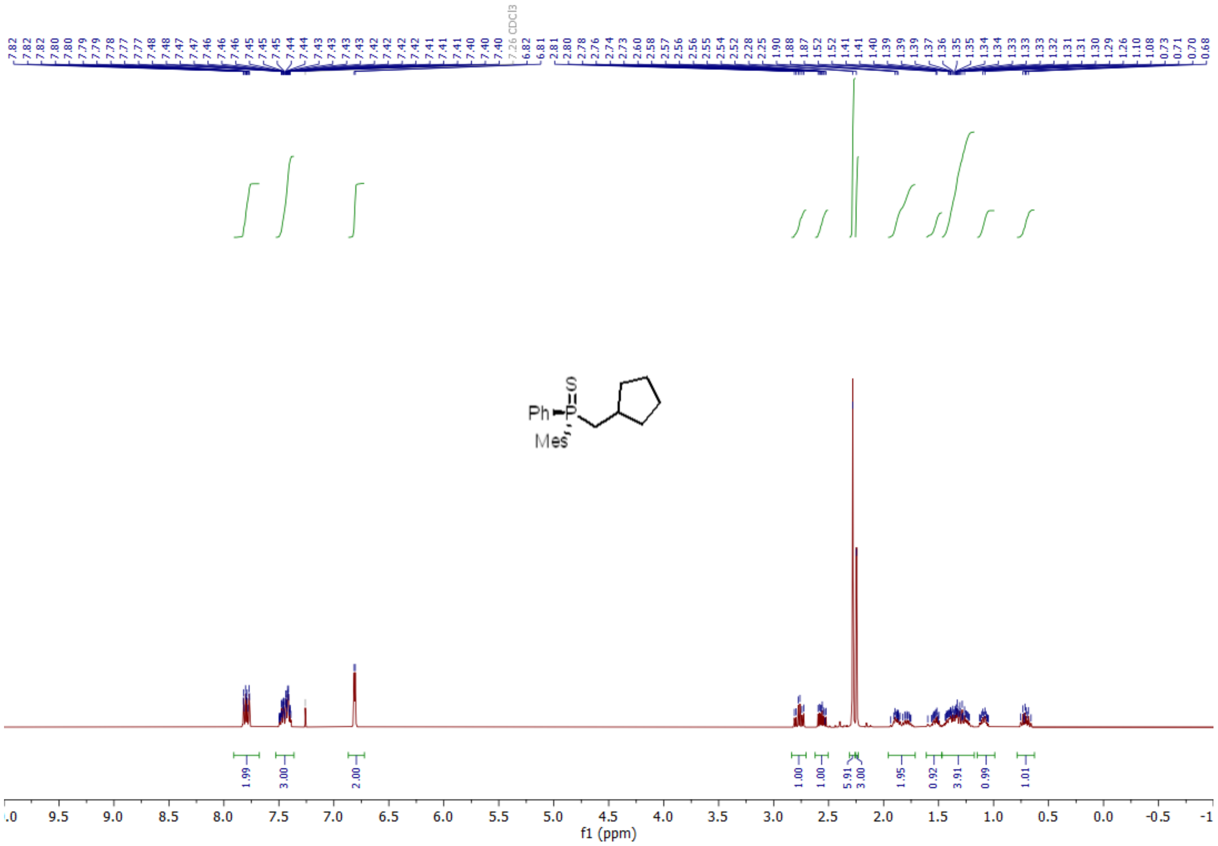

^{13}C NMR (101 MHz, $CDCl_3$)spectrum of **4’c**

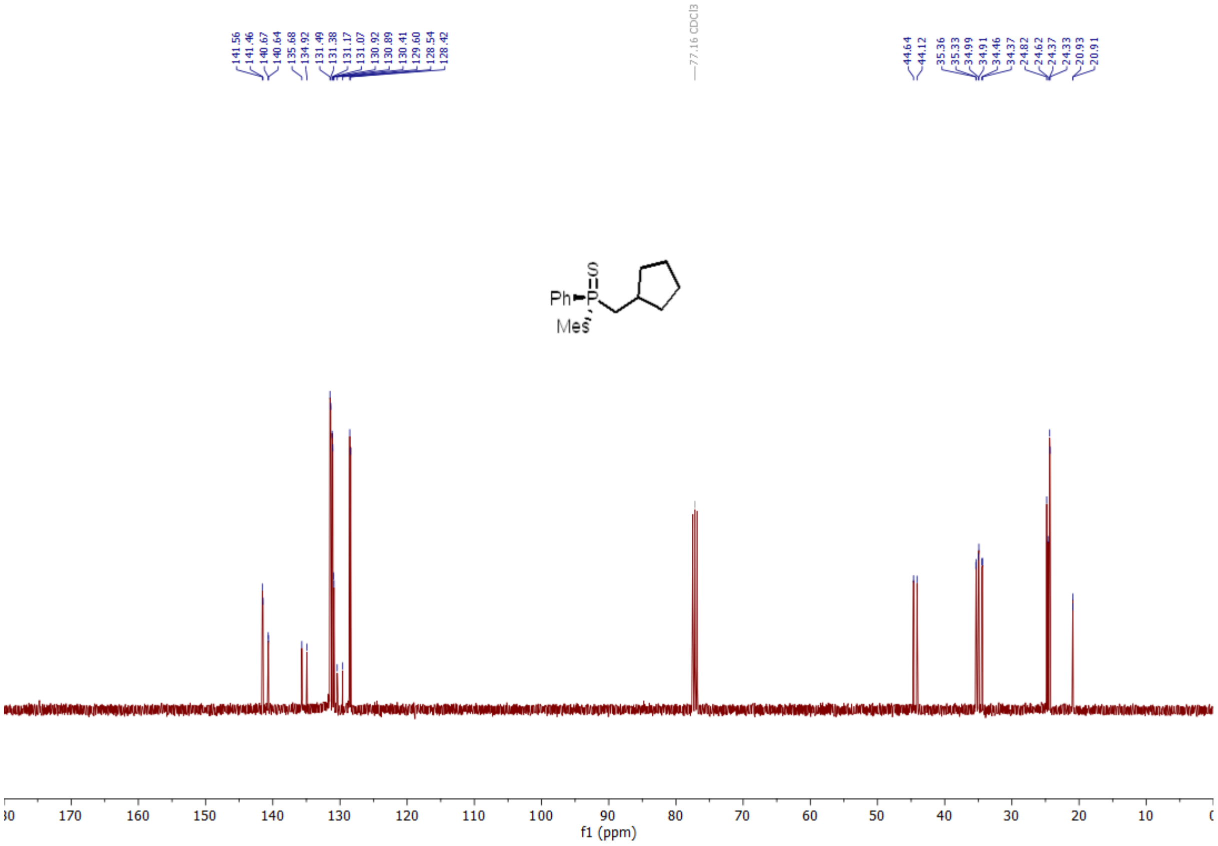

^{31}P NMR (162 MHz, CDCl$_3$) spectrum of **4’c**

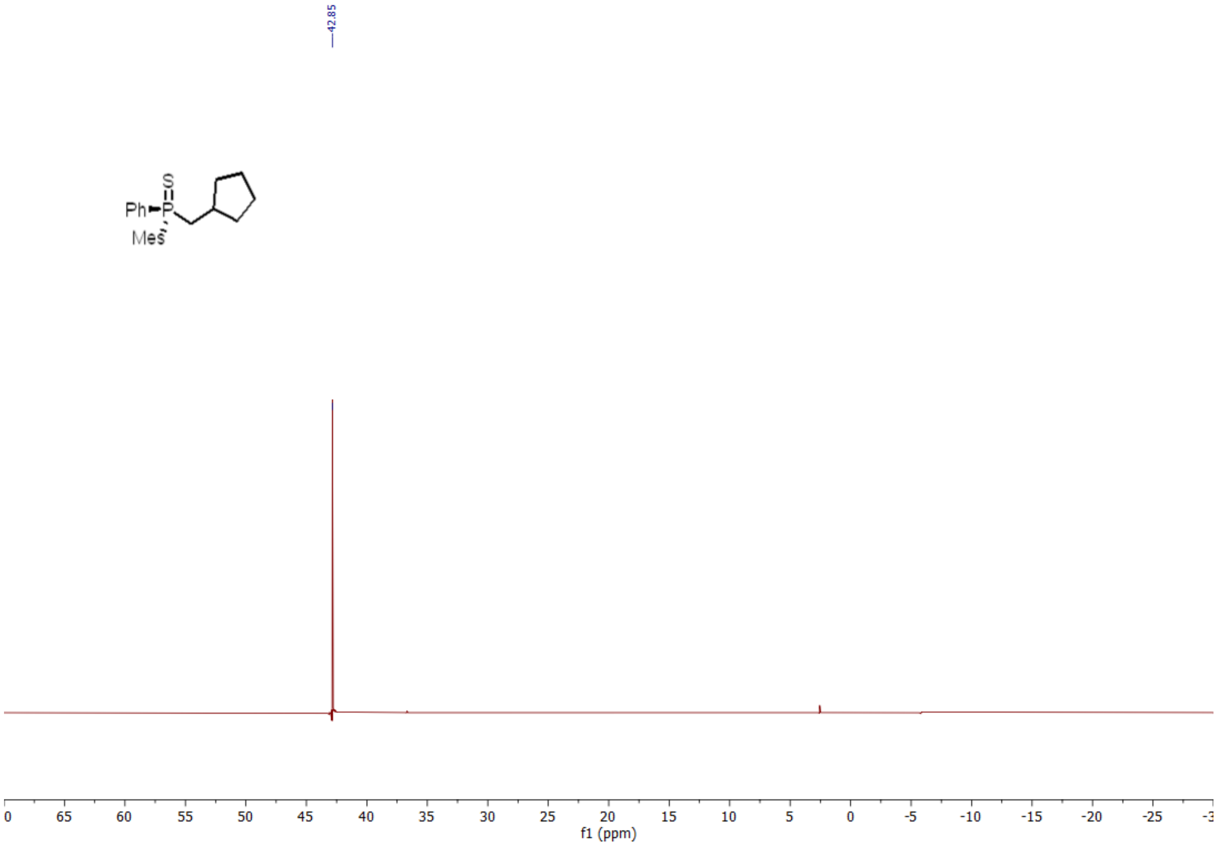

^{1}H NMR (400 MHz, CDCl$_3$) spectrum of **4’d**

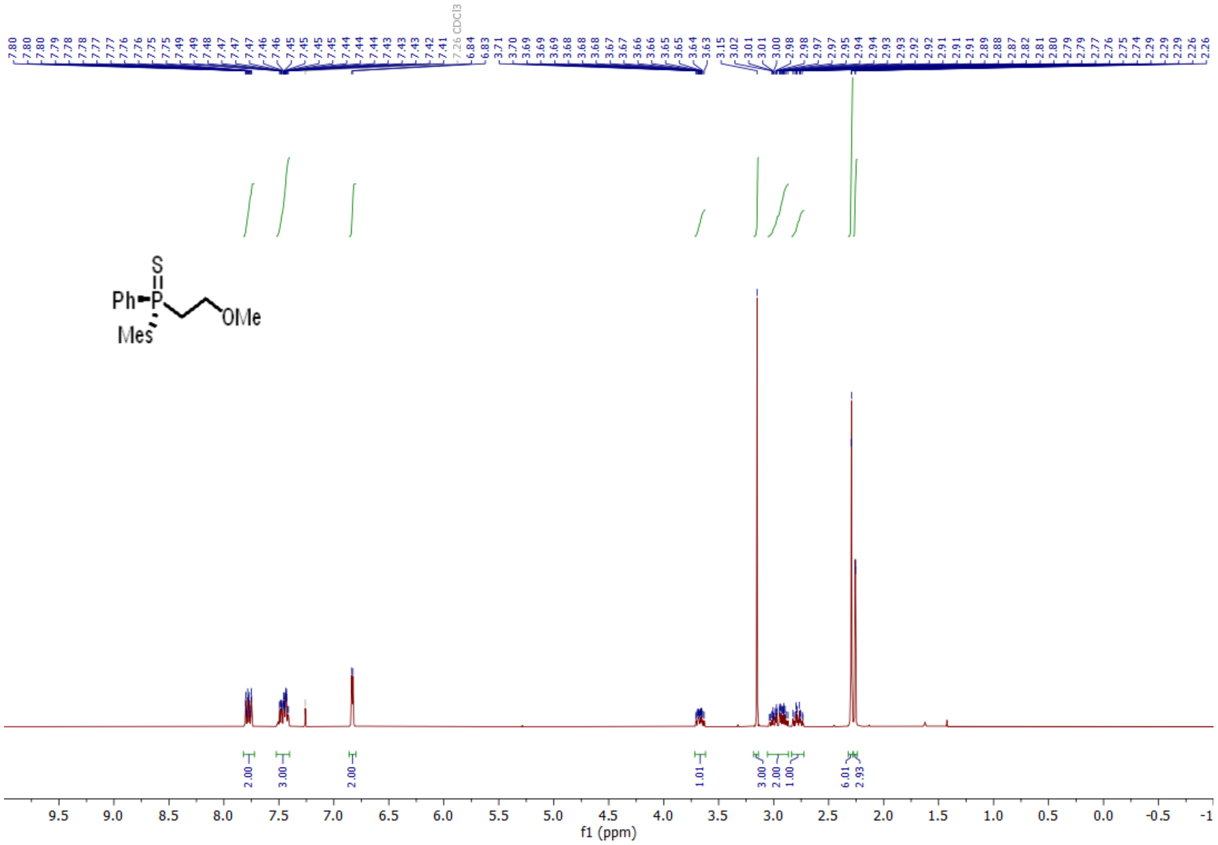

^{13}C NMR (101 MHz, $CDCl_3$)spectrum of **4’d**

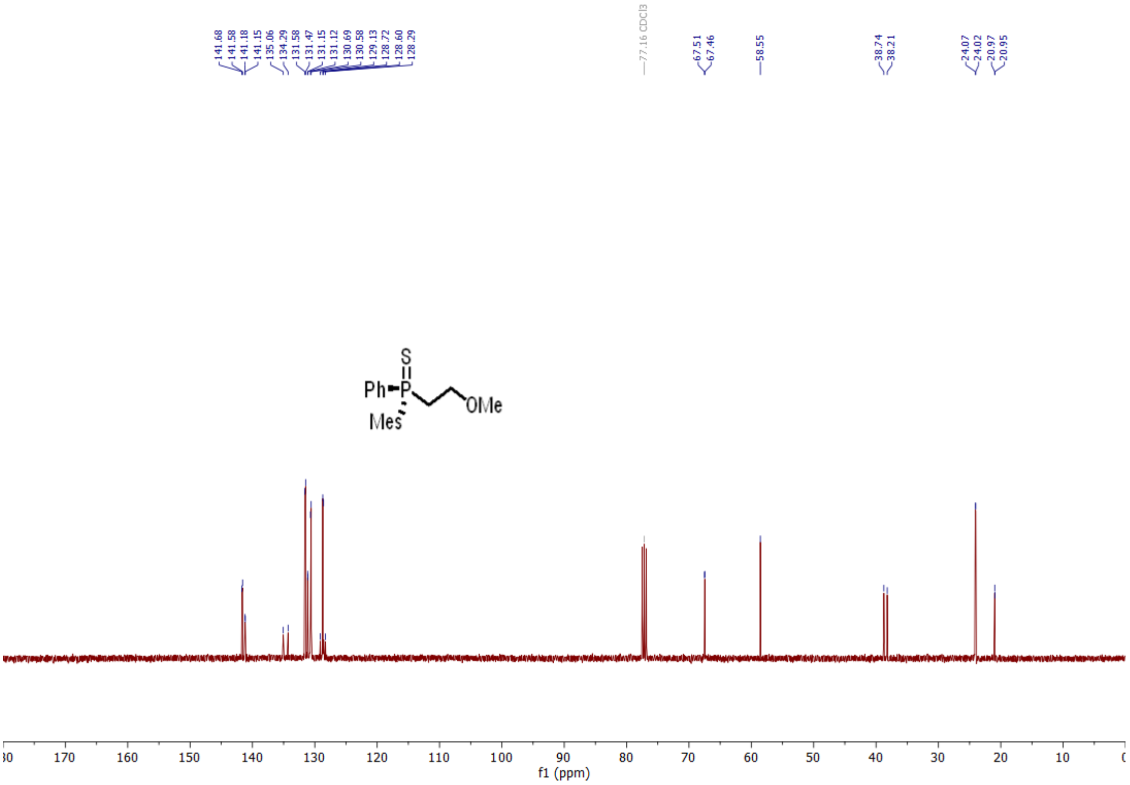

^{31}P NMR (162 MHz, $CDCl_3$) spectrum of **4’d**

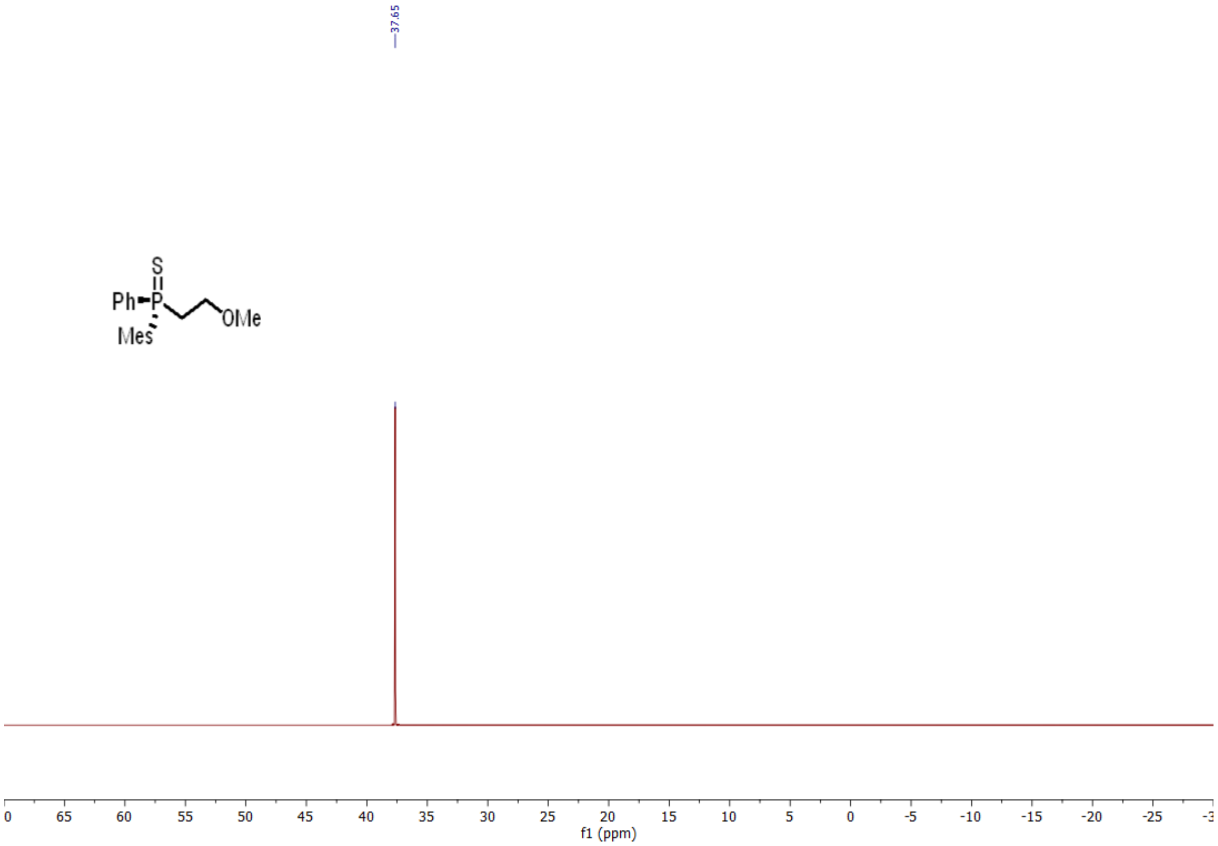

^{1}H NMR (400 MHz, $CDCl_3$) spectrum of **4'e**

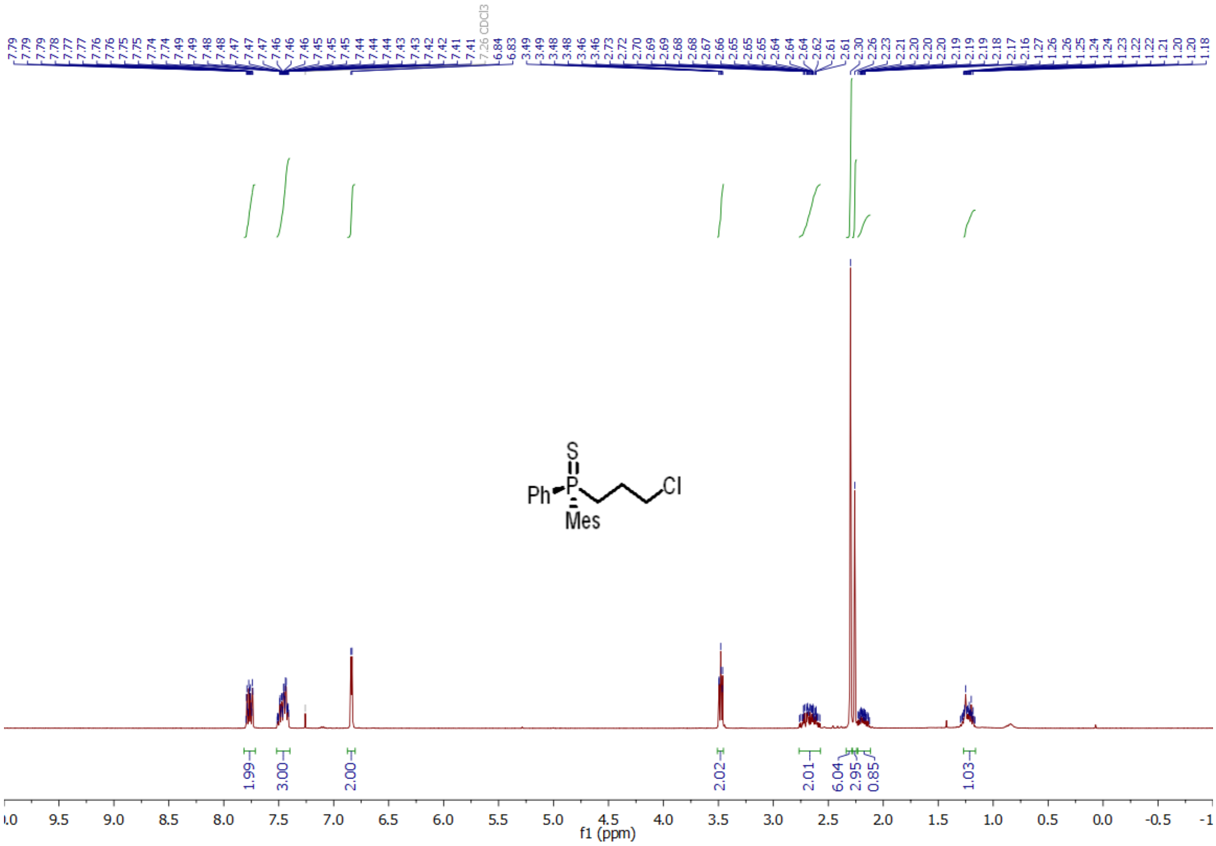

^{13}C NMR (101 MHz, $CDCl_3$)spectrum of **4'e**

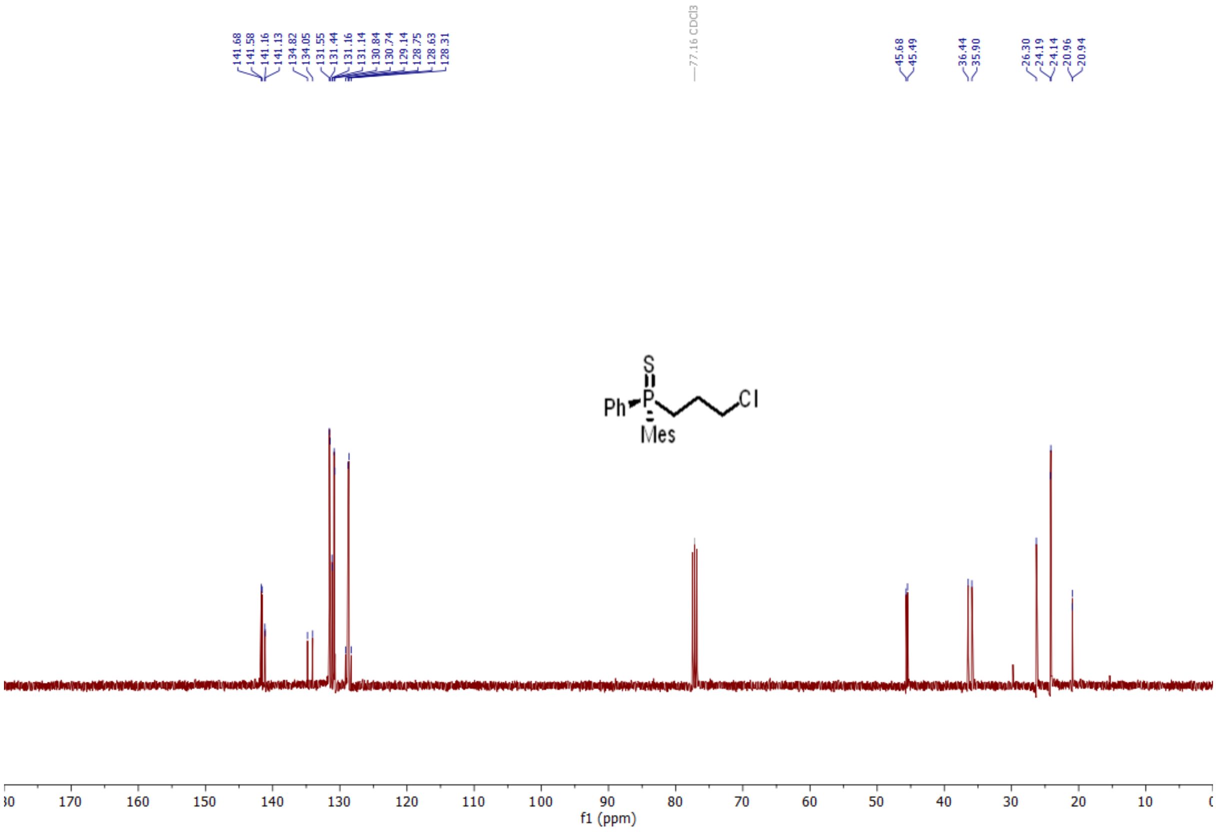

^{31}P NMR (162 MHz, $CDCl_3$) spectrum of **4’e**

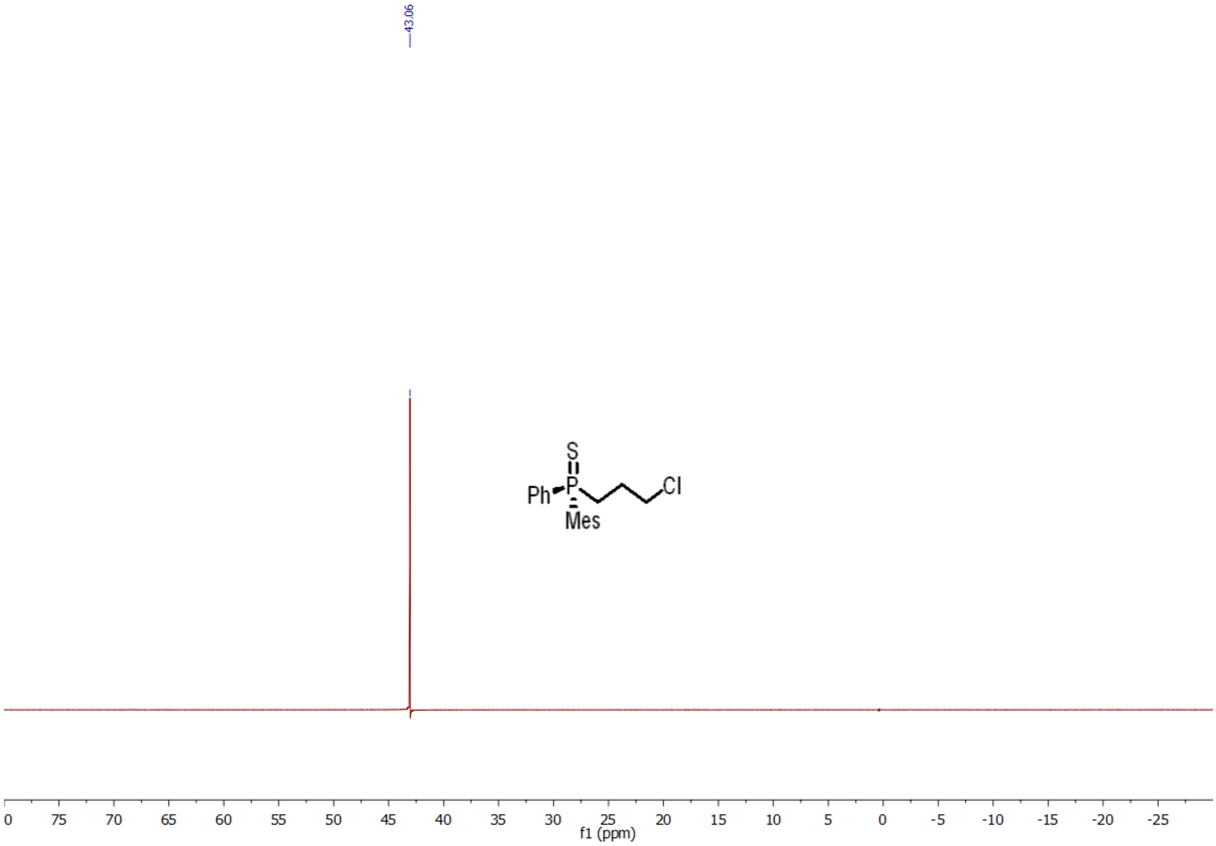

^{1}H NMR (400 MHz, $CDCl_3$) spectrum of **5a’**

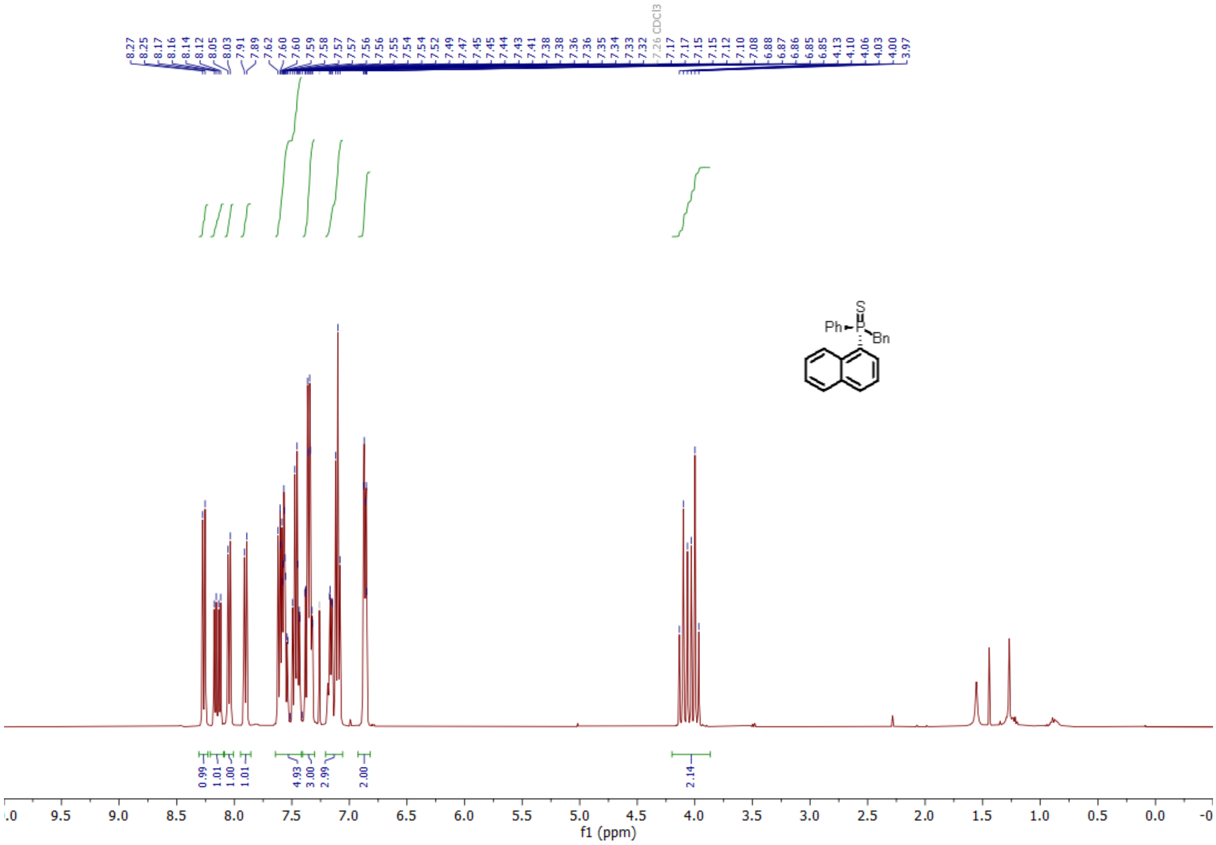

^{13}C NMR (101 MHz, $CDCl_3$)spectrum of **5a'**

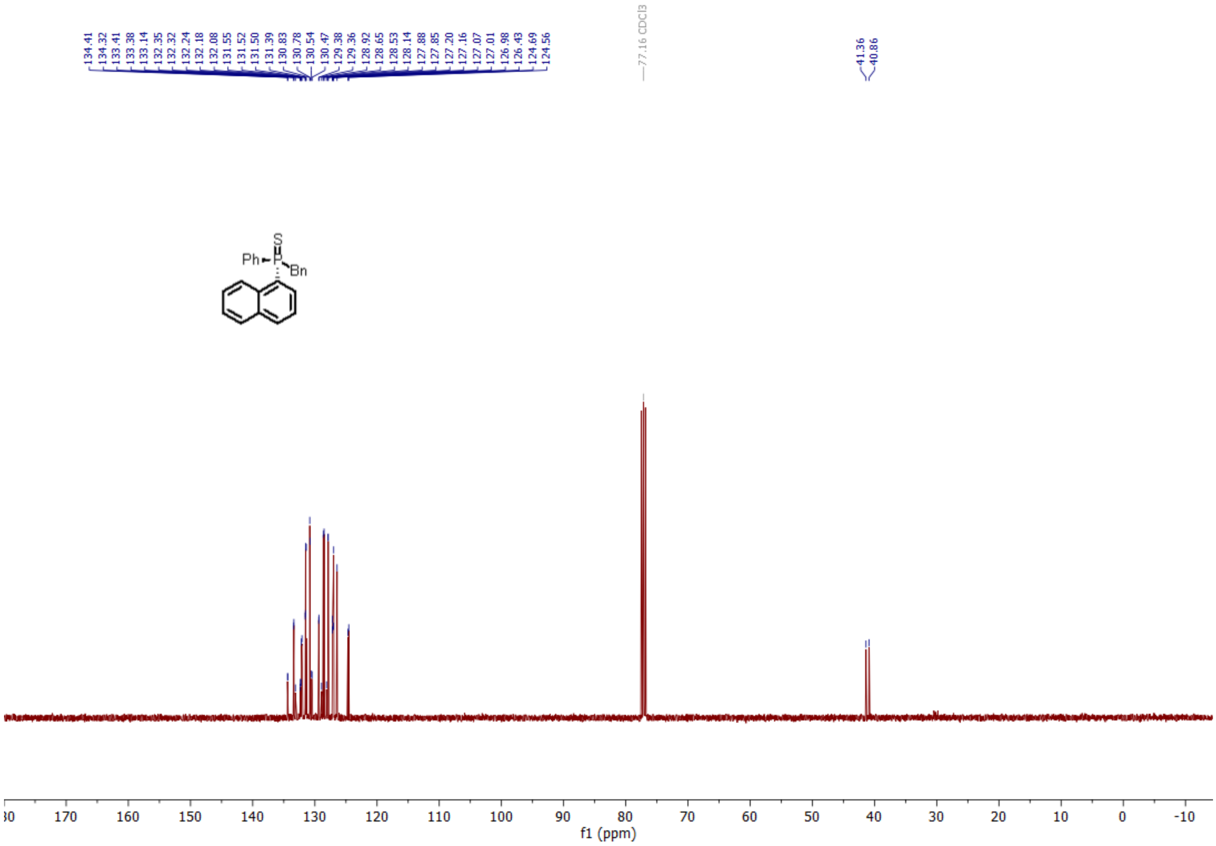

^{31}P NMR (162 MHz, $CDCl_3$) spectrum of **5a'**

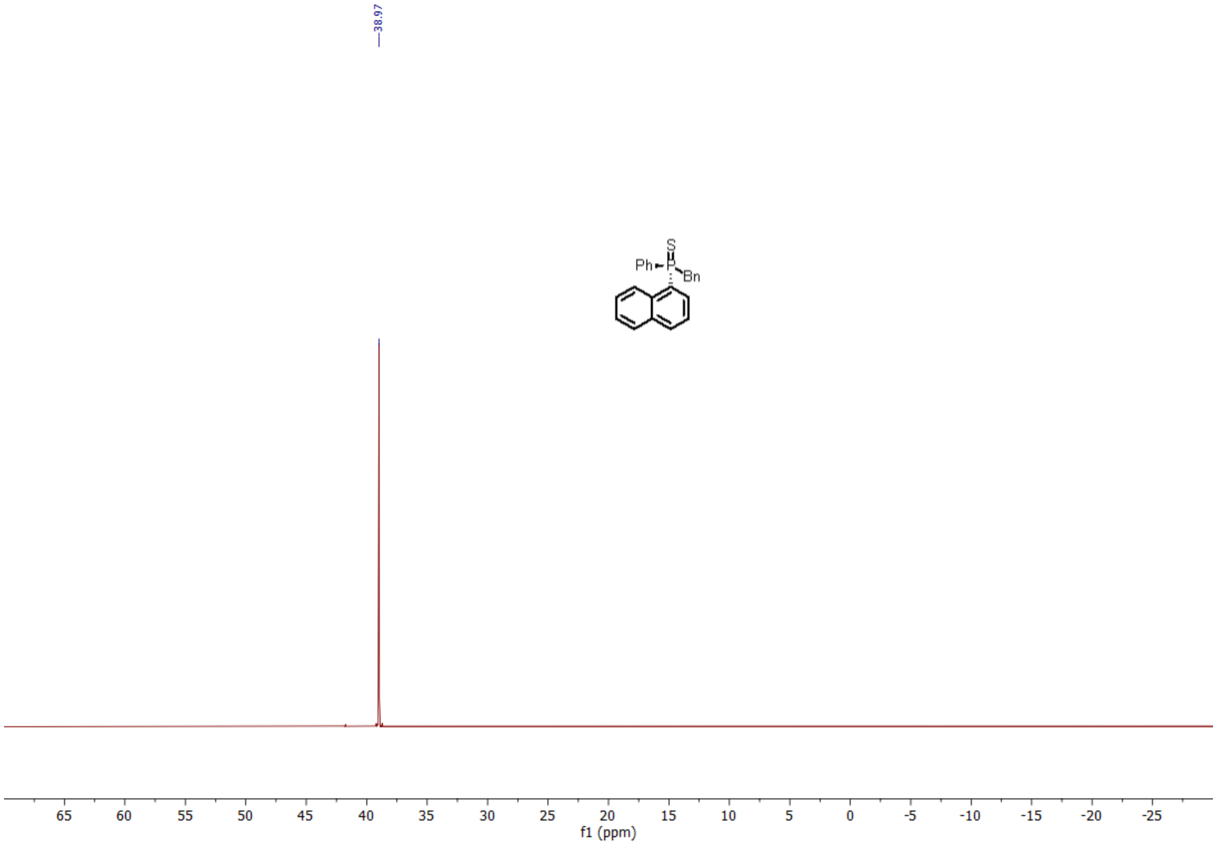

^{1}H NMR (400 MHz, $CDCl_3$) spectrum of **5b'**

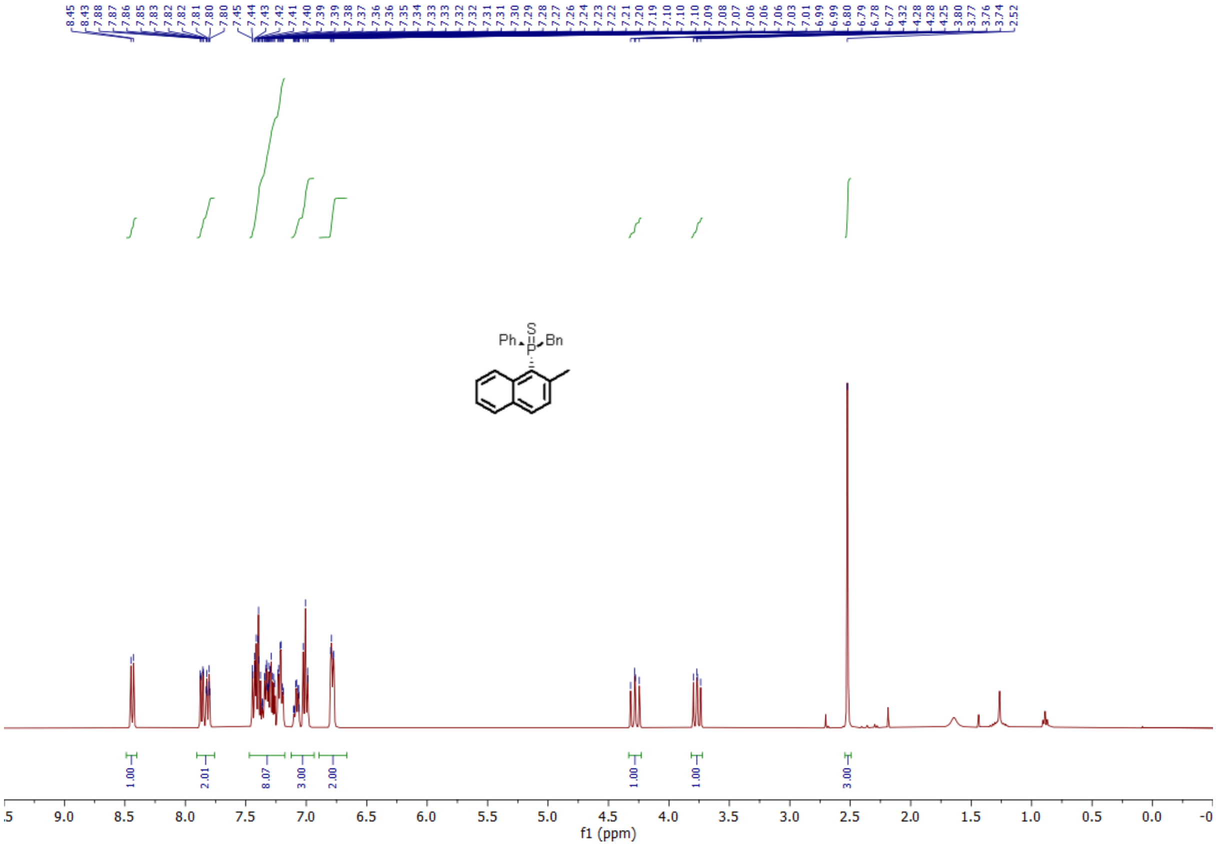

^{13}C NMR (101 MHz, $CDCl_3$)spectrum of **5b '**

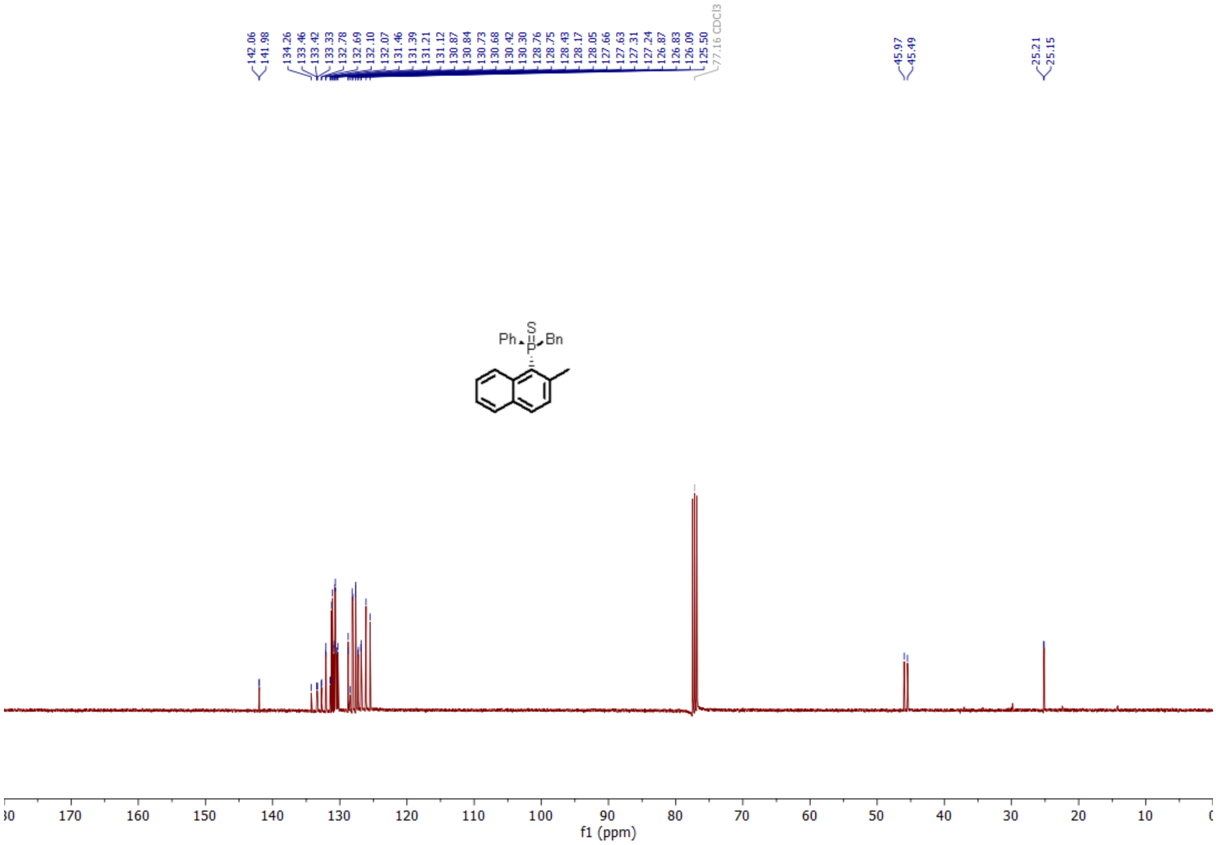

^{31}P NMR (162 MHz, $CDCl_3$) spectrum of **5b’**

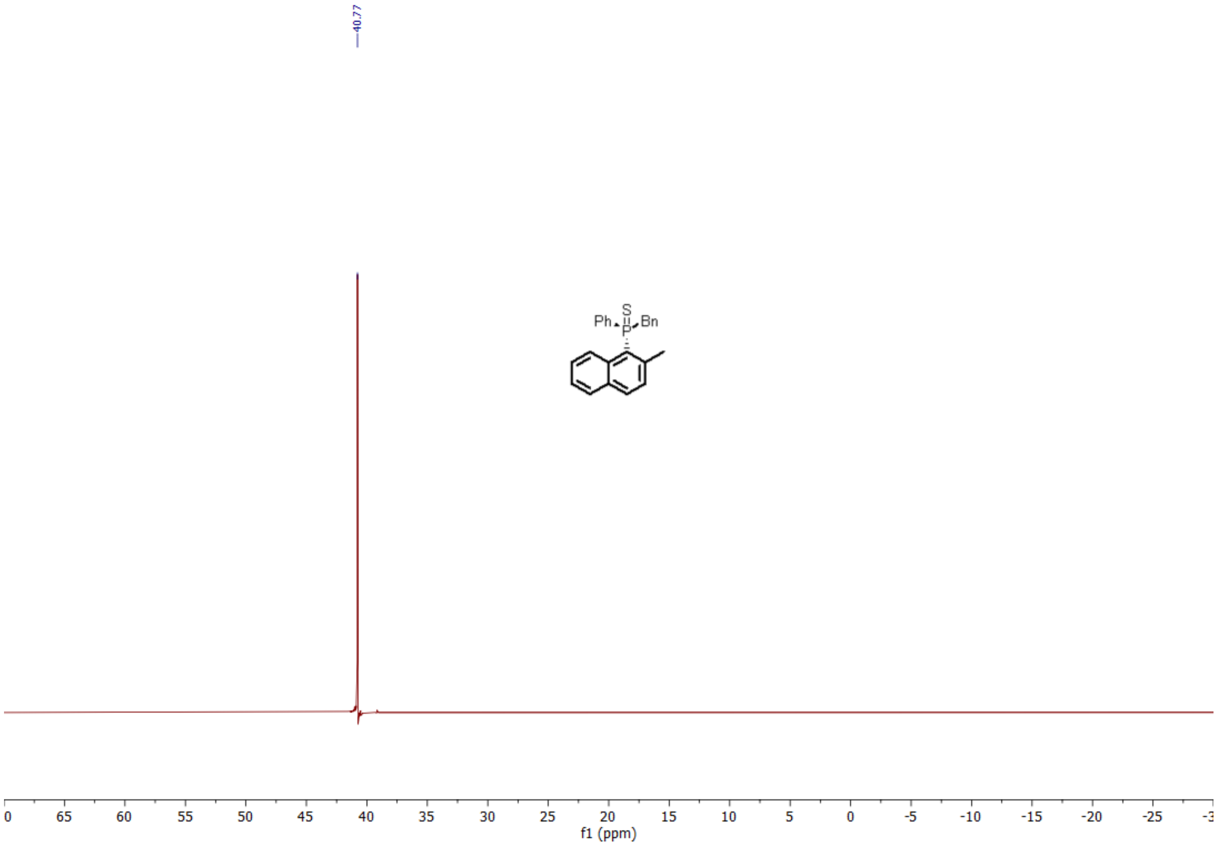

^{1}H NMR (400 MHz, $CDCl_3$) spectrum of **5c’**

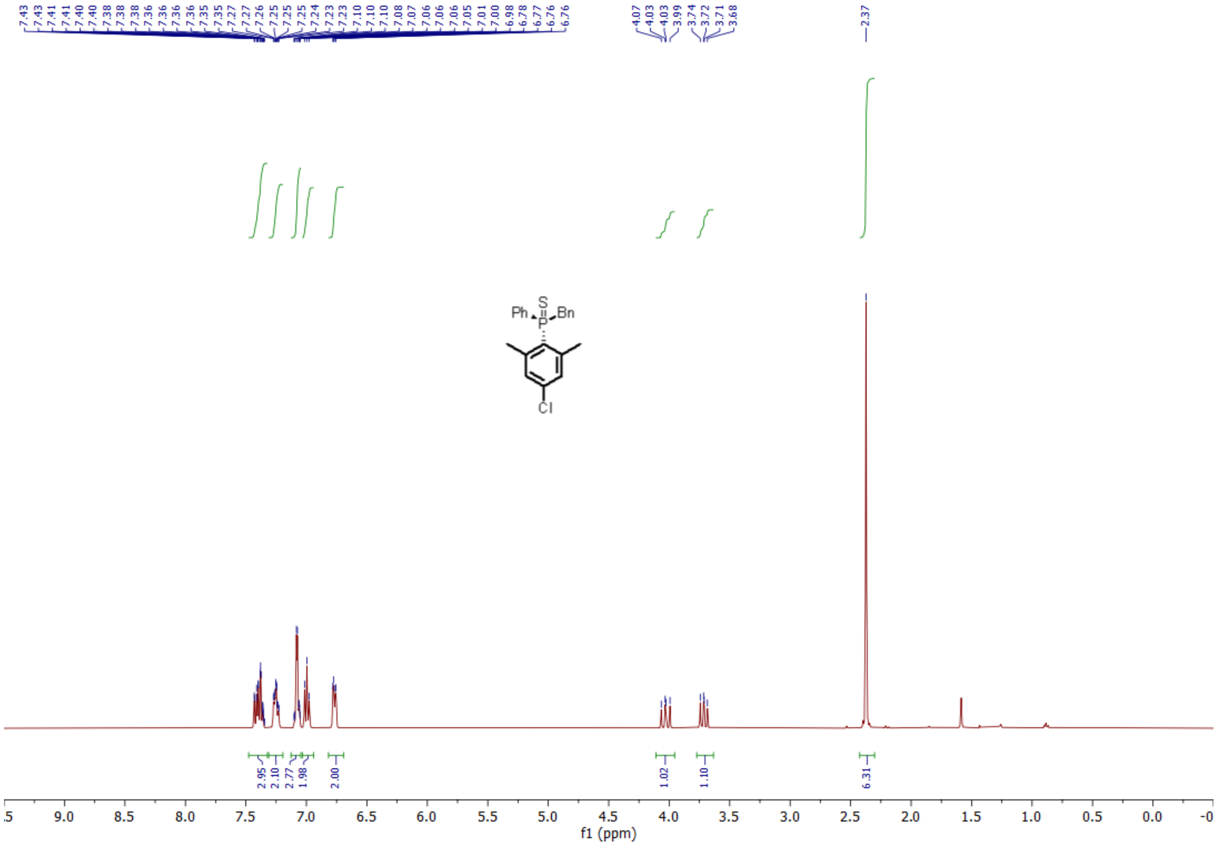

^{13}C NMR (101 MHz, $CDCl_3$)spectrum of **5c'**

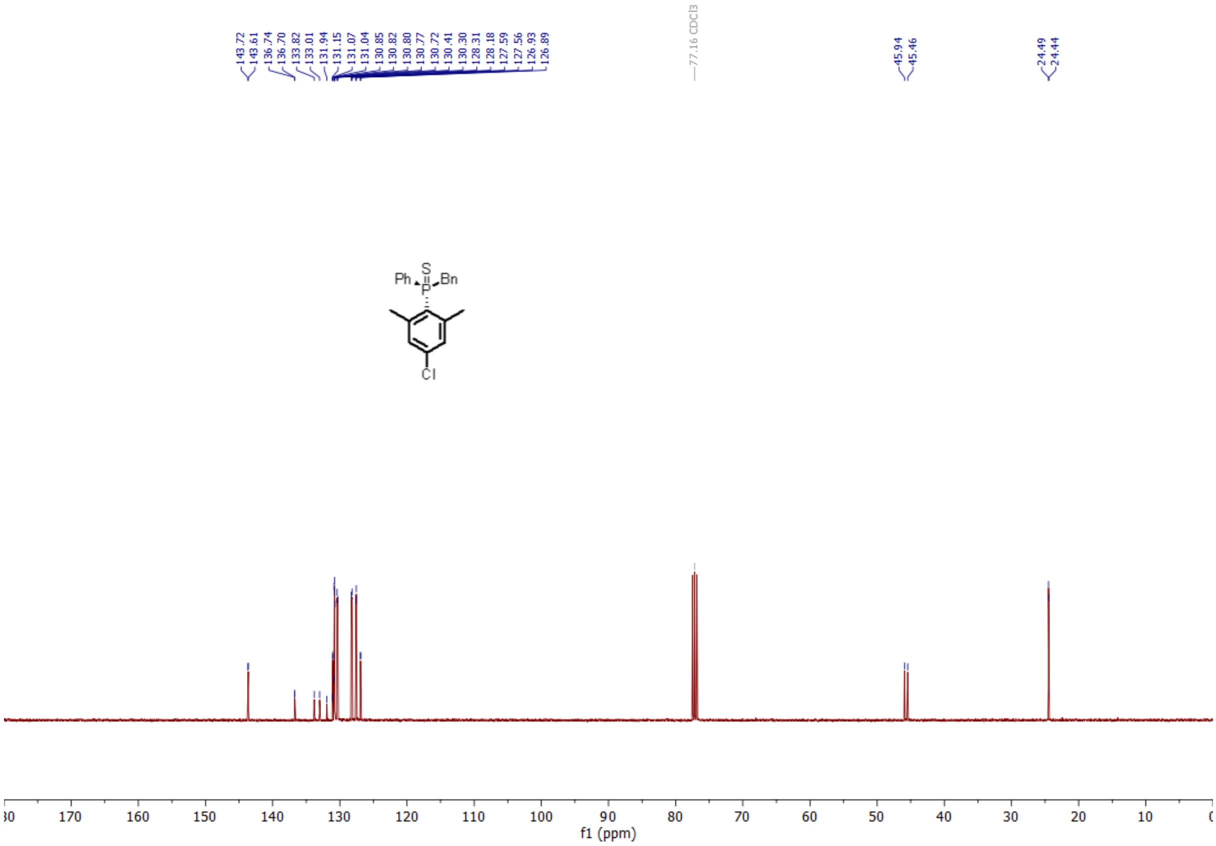

^{31}P NMR (162 MHz, $CDCl_3$) spectrum of **5c'**

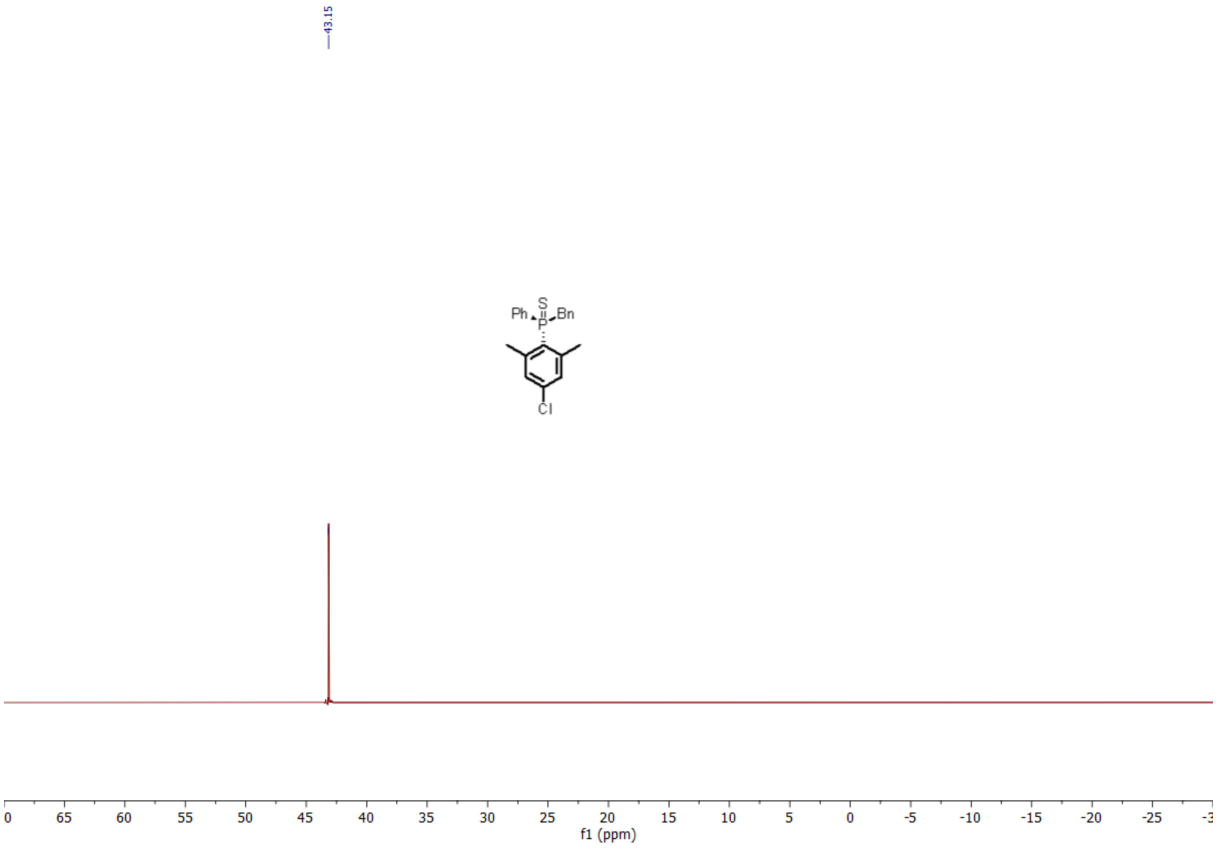

^{1}H NMR (400 MHz, $CDCl_3$) spectrum of **5d’**

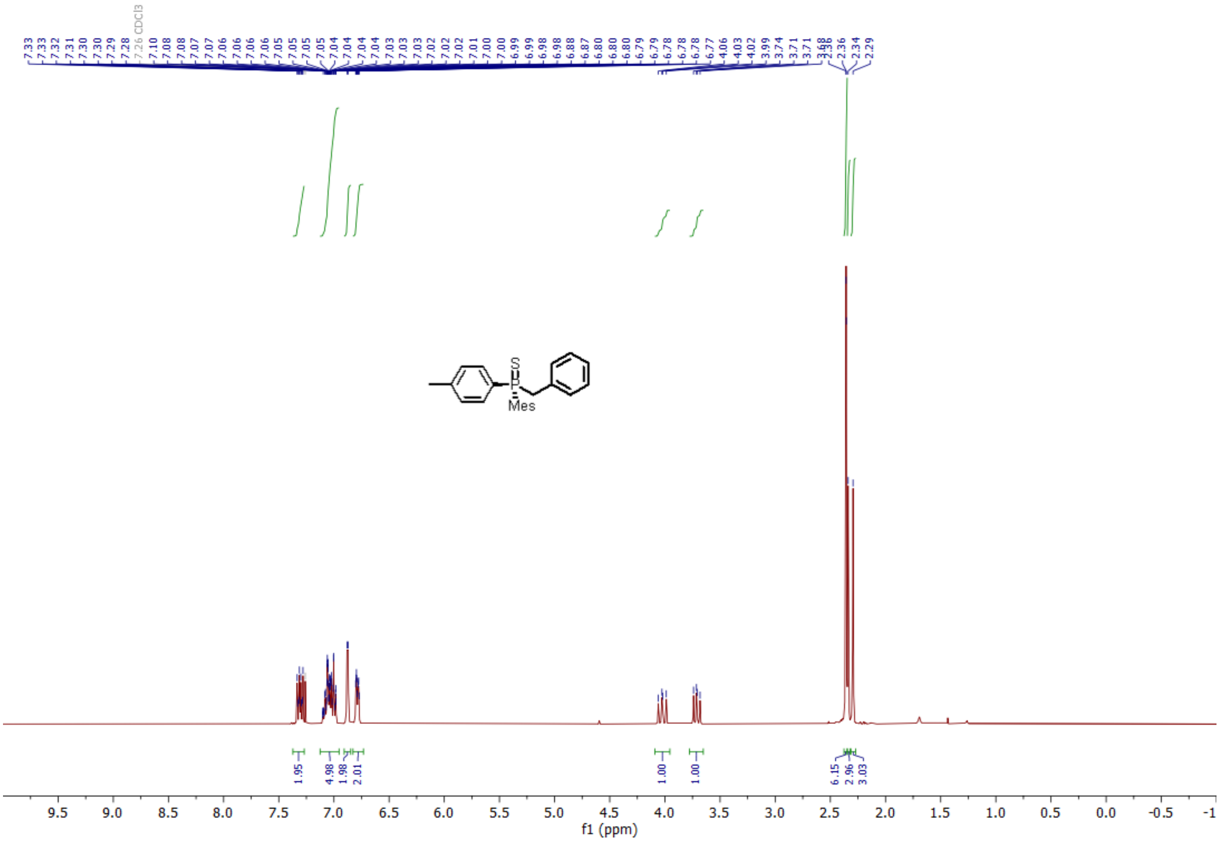

^{13}C NMR (101 MHz, $CDCl_3$)spectrum of **5d’**

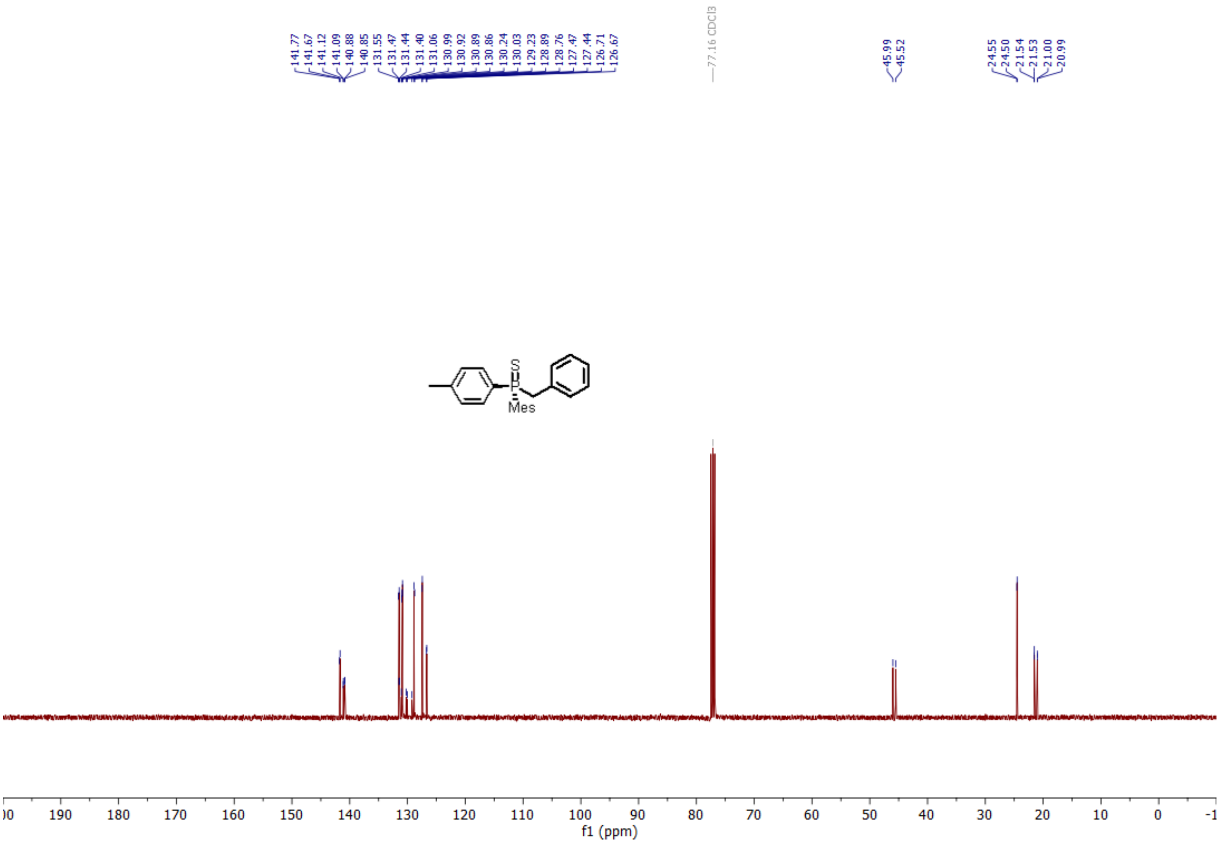

^{31}P NMR (162 MHz, $CDCl_3$) spectrum of **5d’**

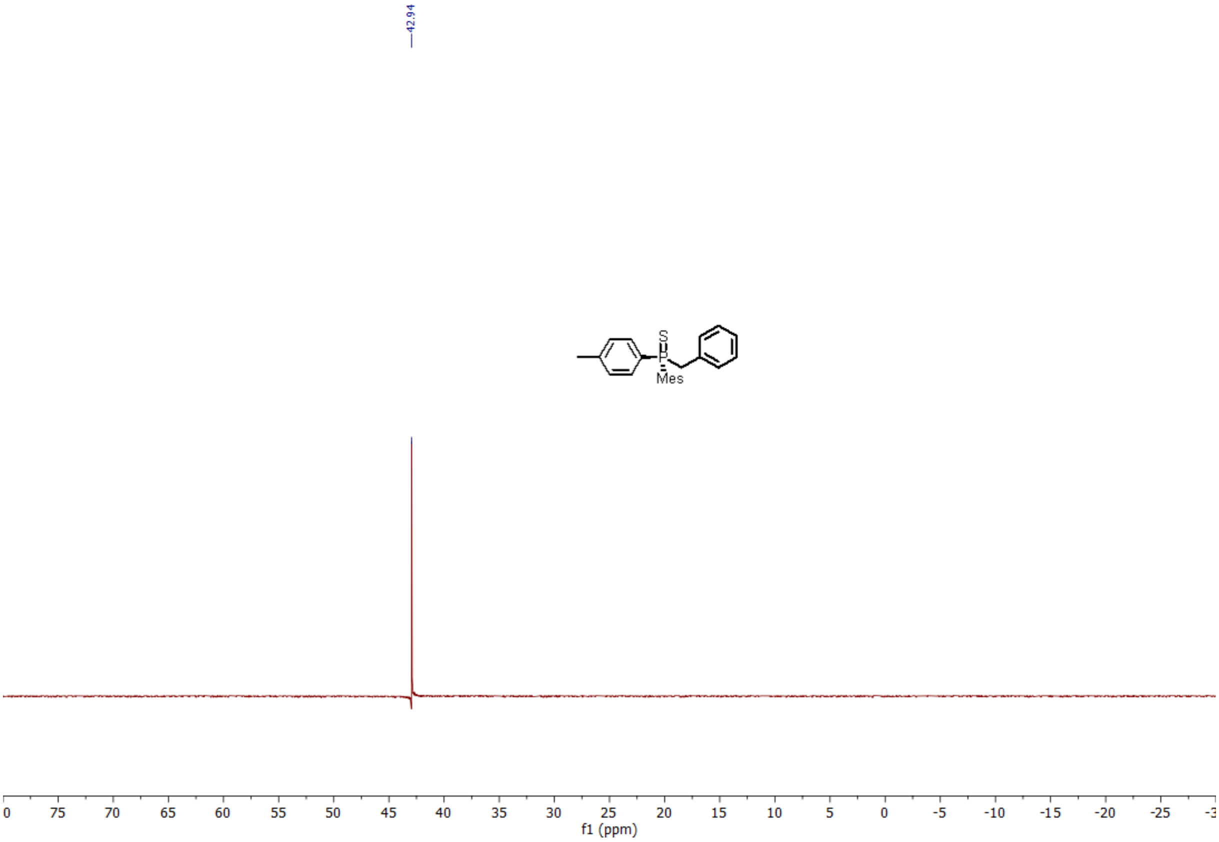

^{1}H NMR (400 MHz, $CDCl_3$) spectrum of **5e’**

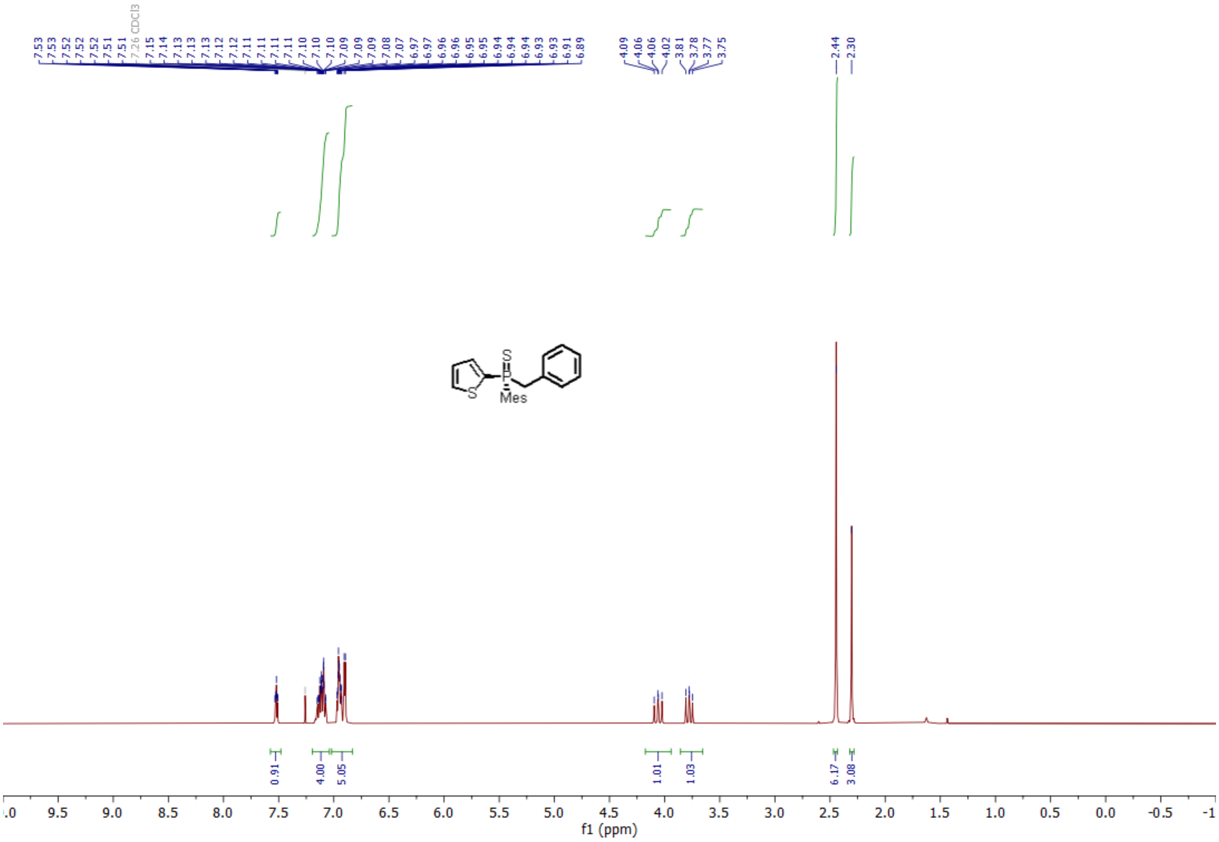

^{13}C NMR (101 MHz, $CDCl_3$)spectrum of **5e’**

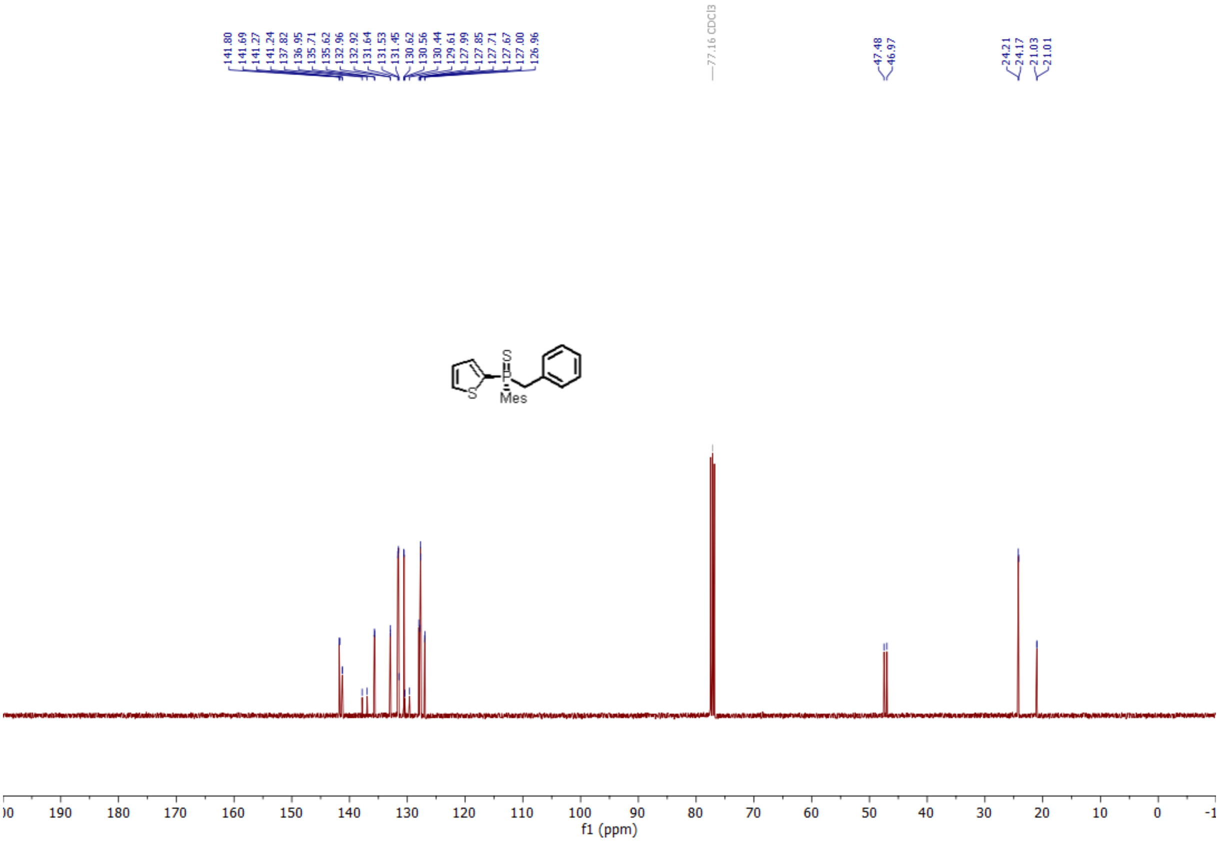

^{31}P NMR (162 MHz, $CDCl_3$) spectrum of **5e’**

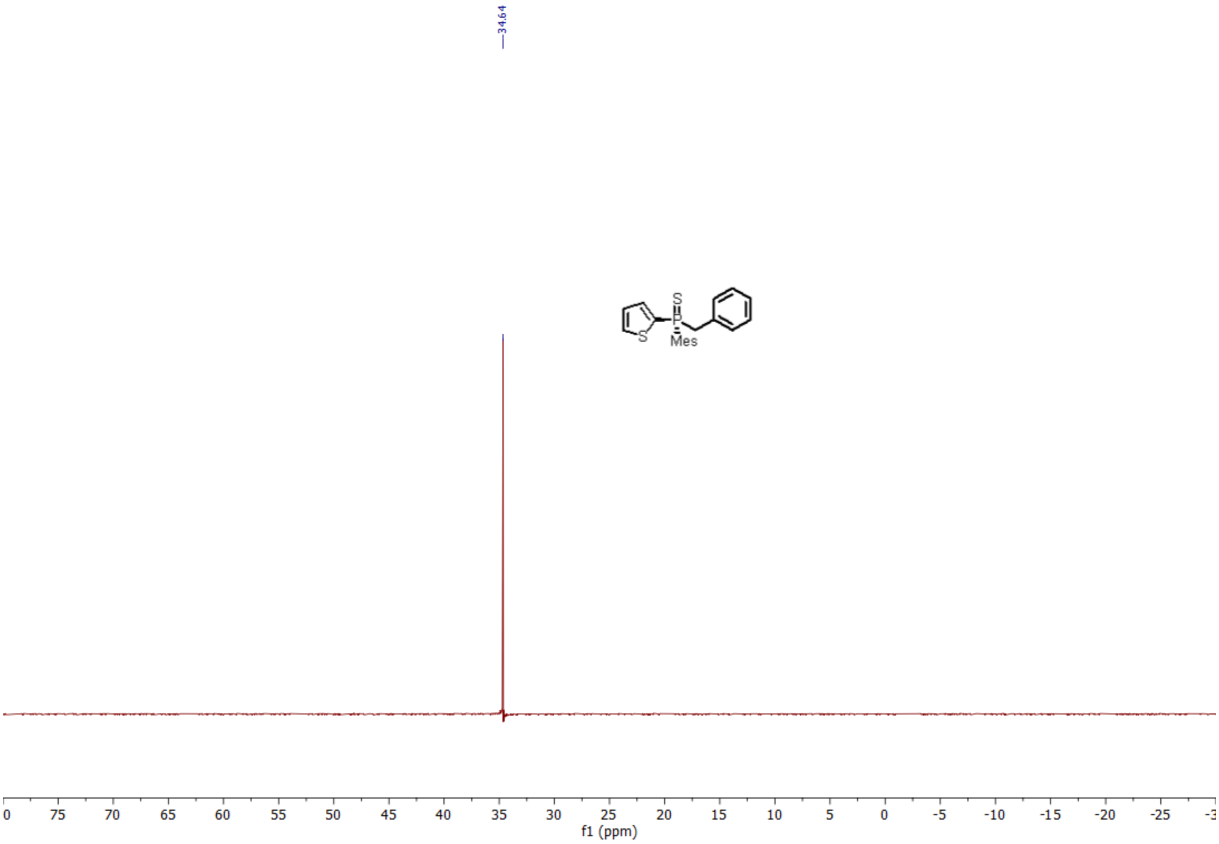

^{1}H NMR (400 MHz, $CDCl_3$) spectrum of **5f'**

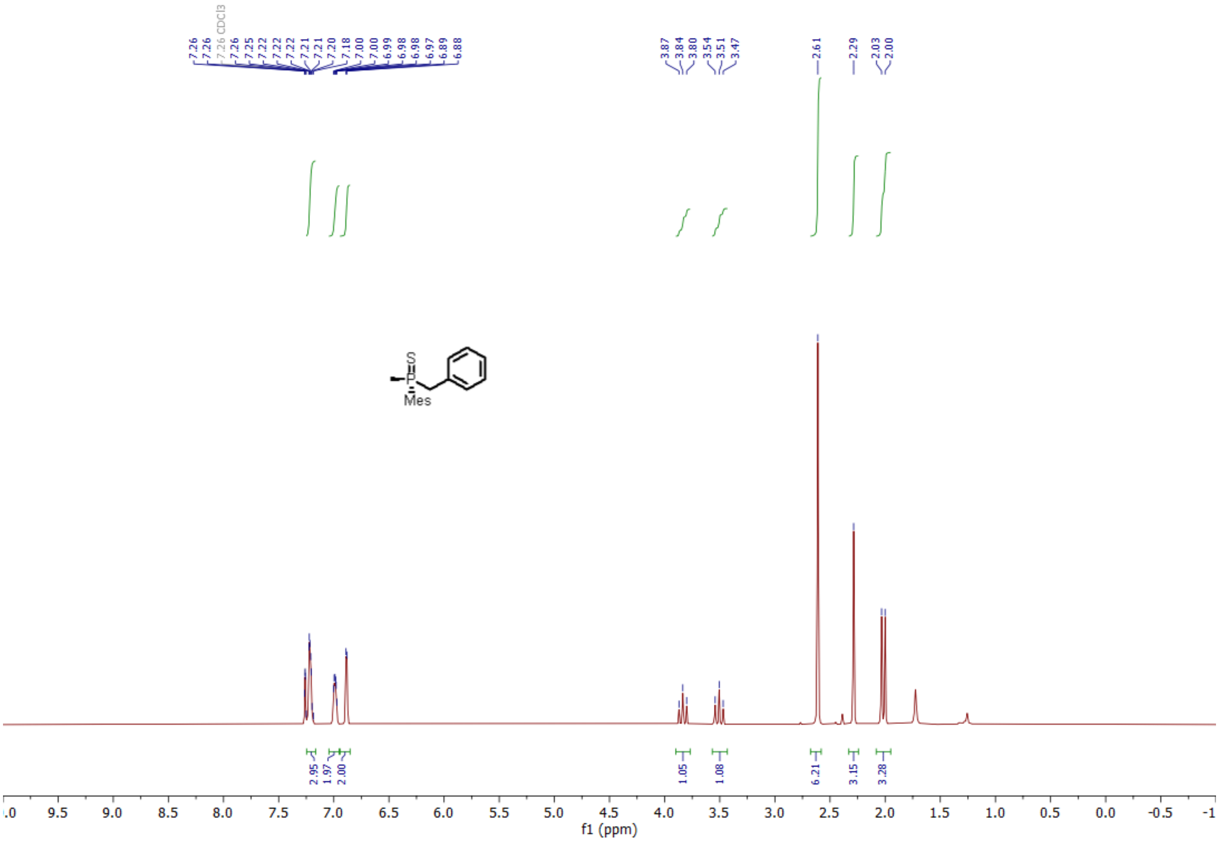

^{13}C NMR (101 MHz, $CDCl_3$)spectrum of **5f'**

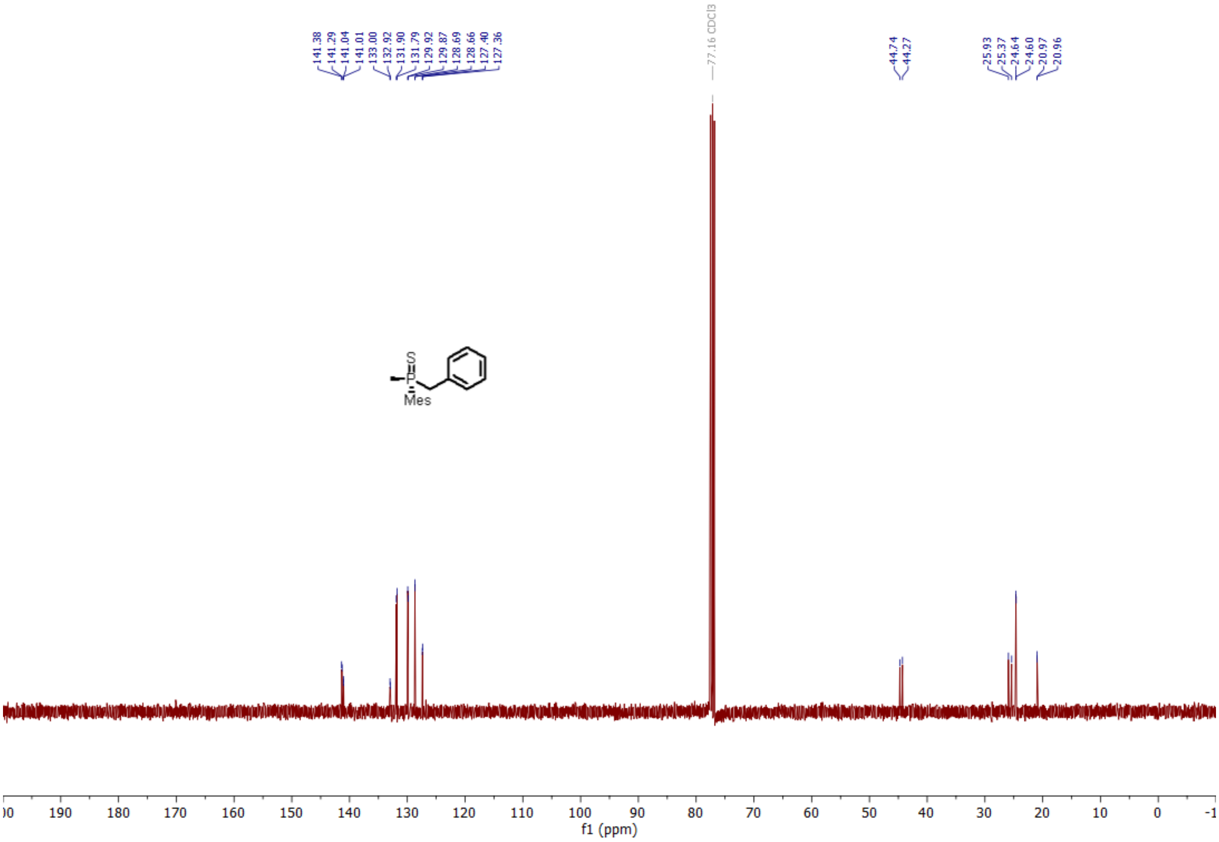

^{31}P NMR (162 MHz, $CDCl_3$) spectrum of **5f'**

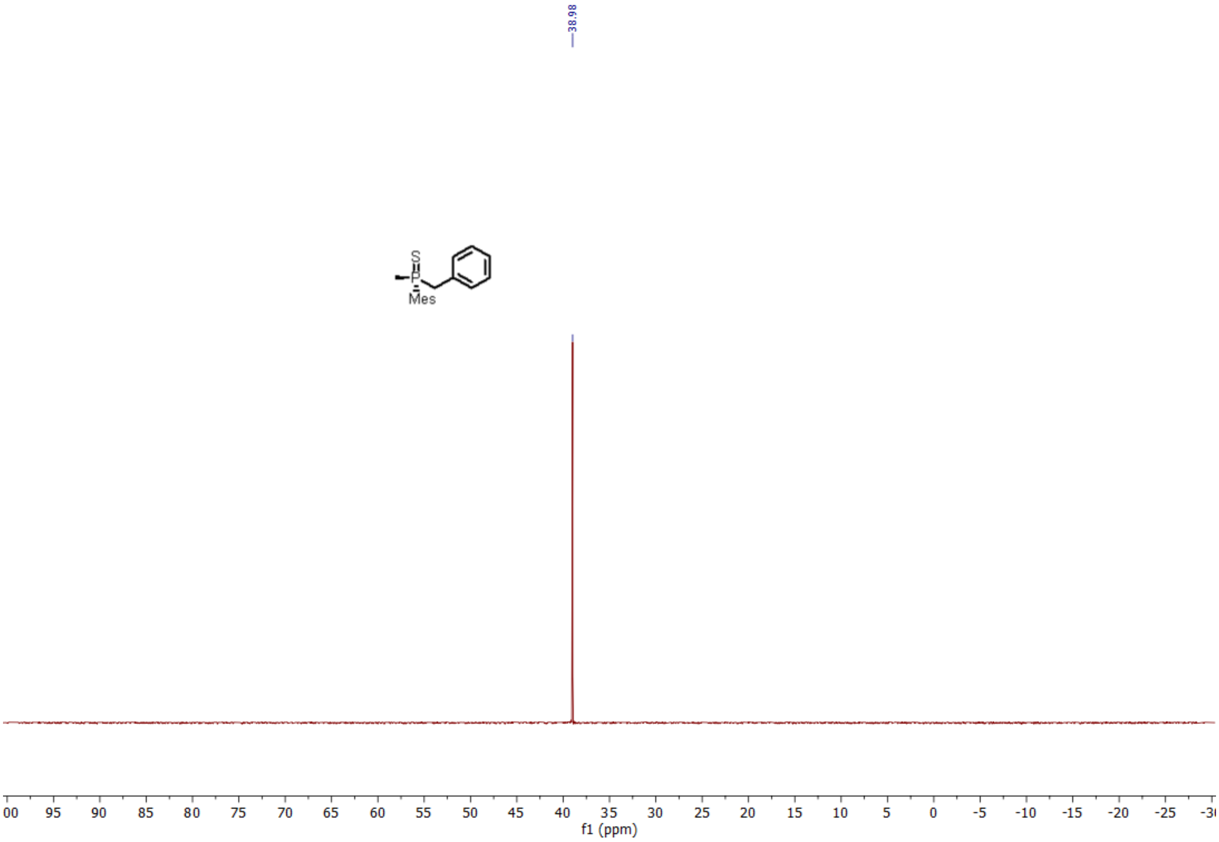

^{1}H NMR (400 MHz, $CDCl_3$) spectrum of **6'a**

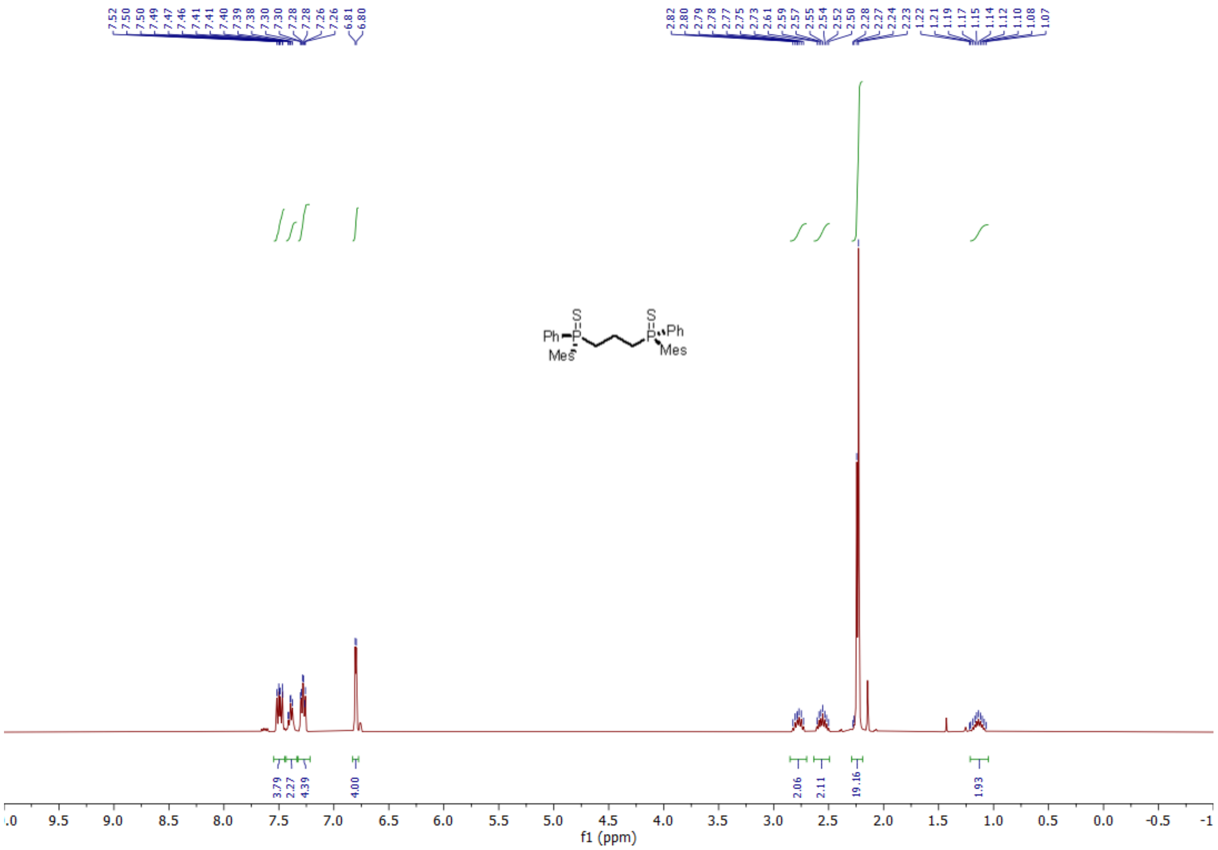

[13] **C NMR** (101 MHz, $CDCl_3$)spectrum of **6’a**

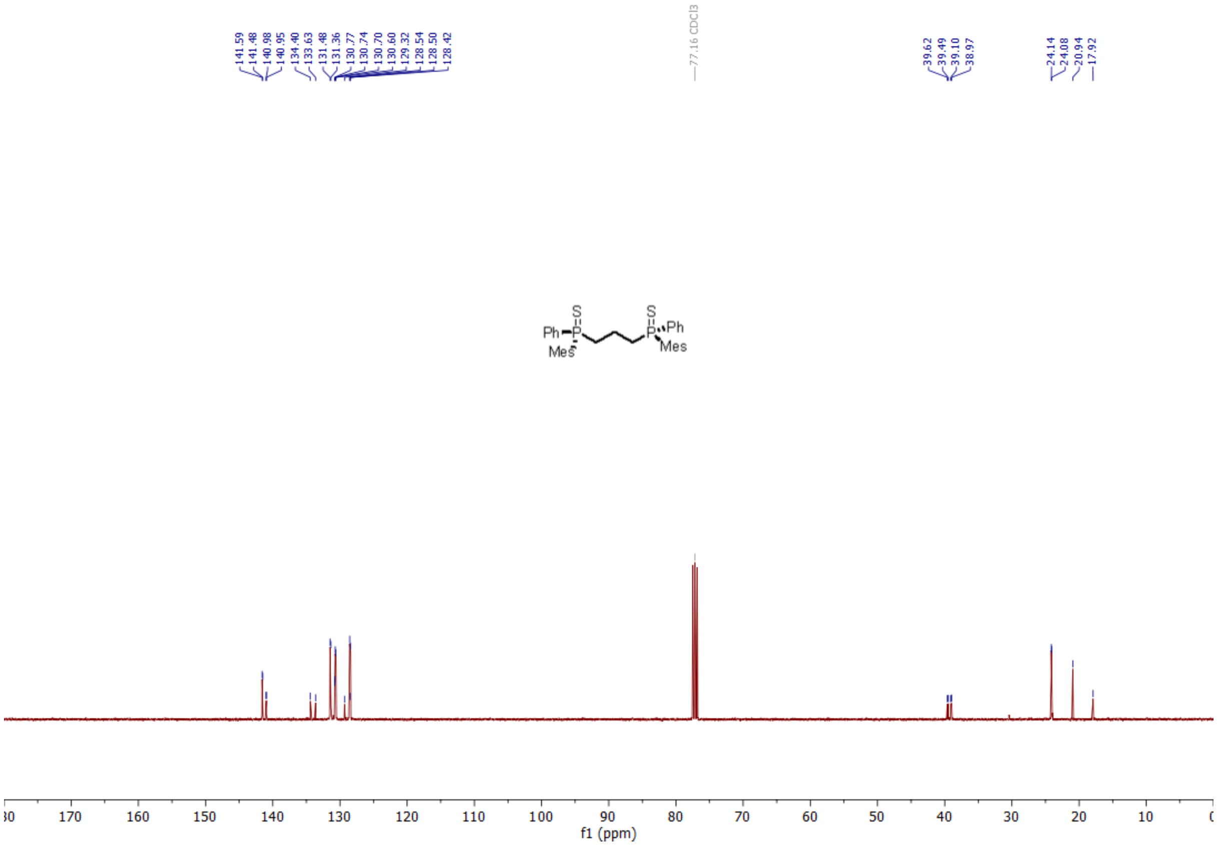

[31] **P NMR** (162 MHz, $CDCl_3$) spectrum of **6’a**

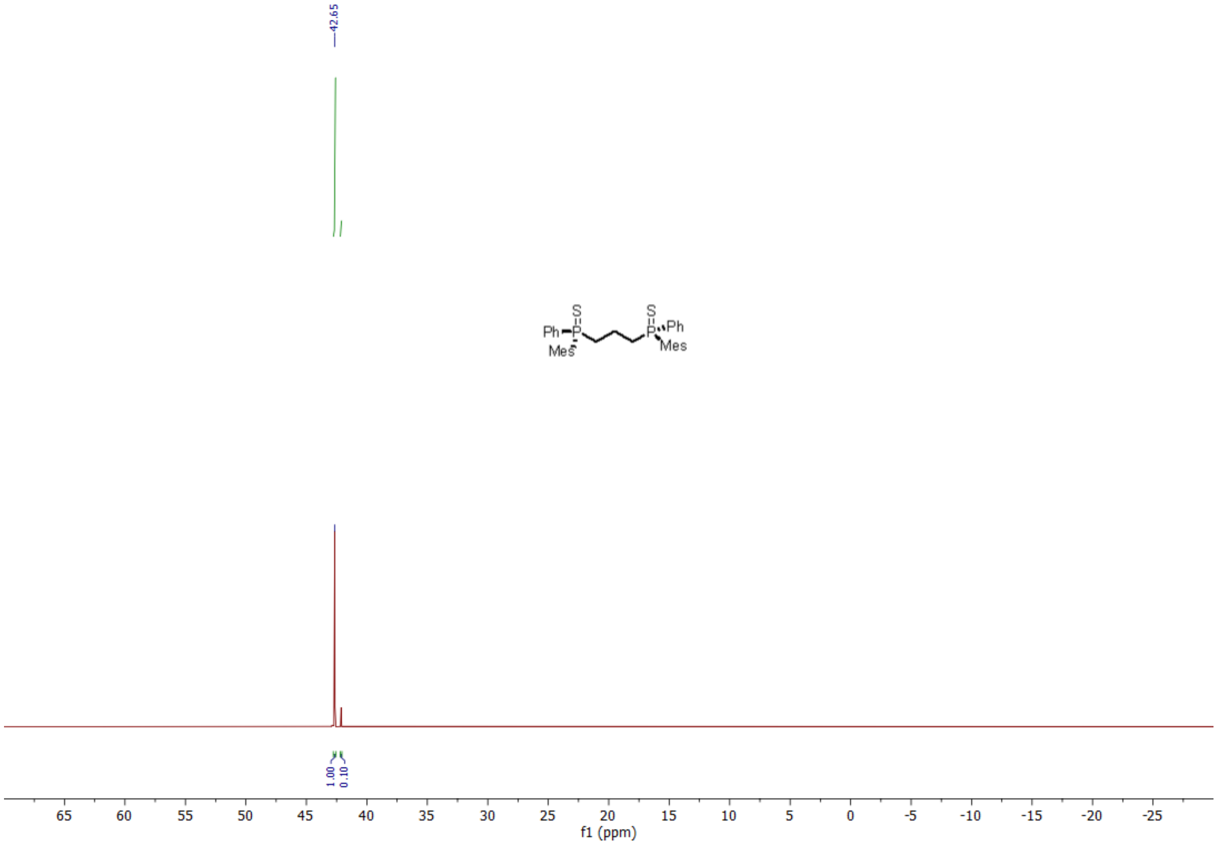

^{1}H NMR (400 MHz, $CDCl_3$) spectrum of **6’b**

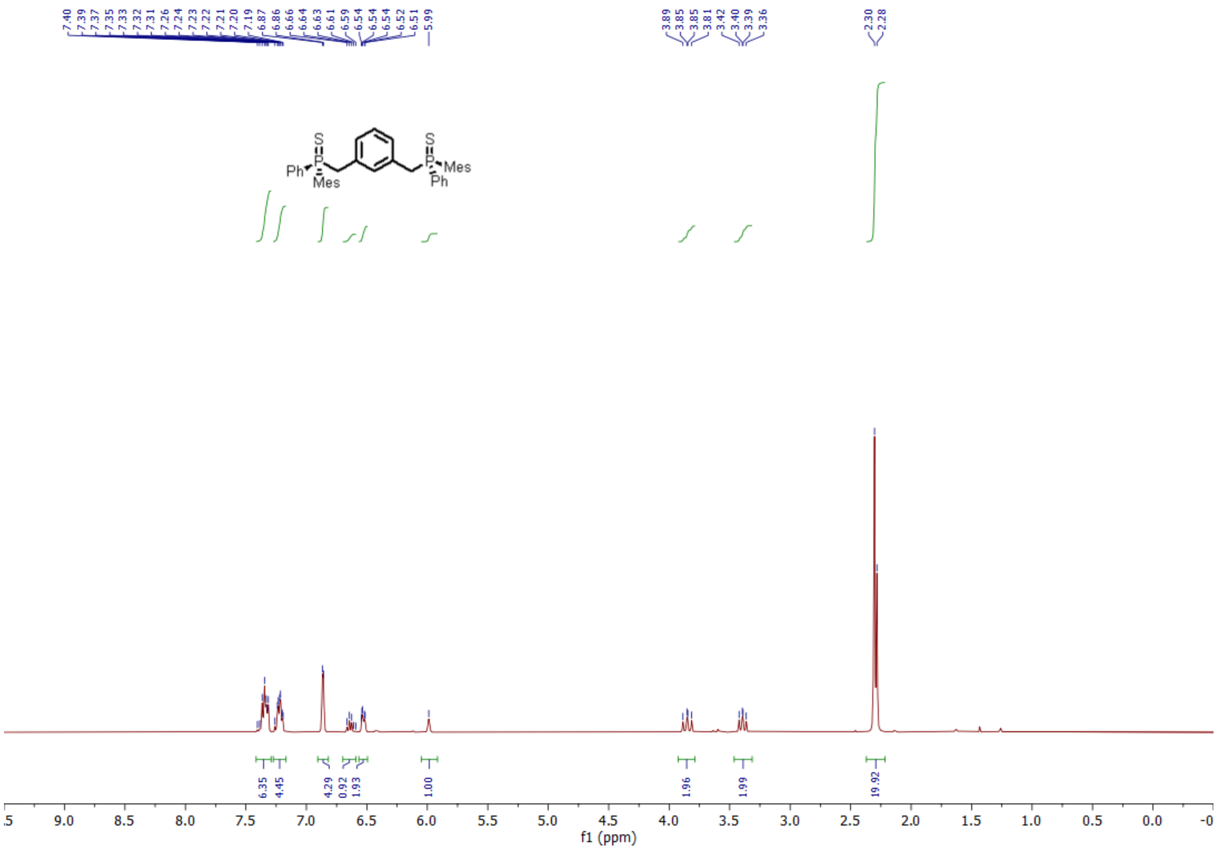

^{13}C NMR (101 MHz, $CDCl_3$)spectrum of **6’b**

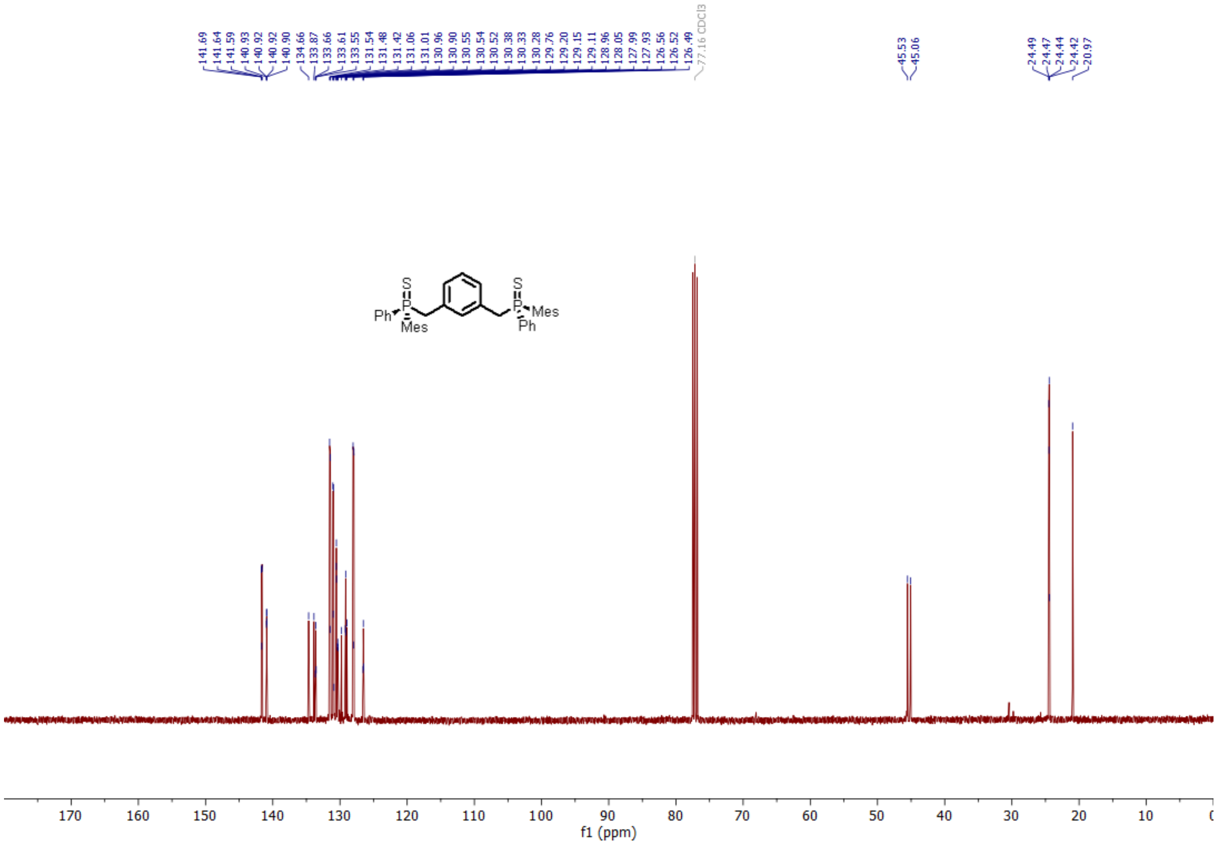

^{31}**P NMR** (162 MHz, $CDCl_3$) spectrum of **6’b**

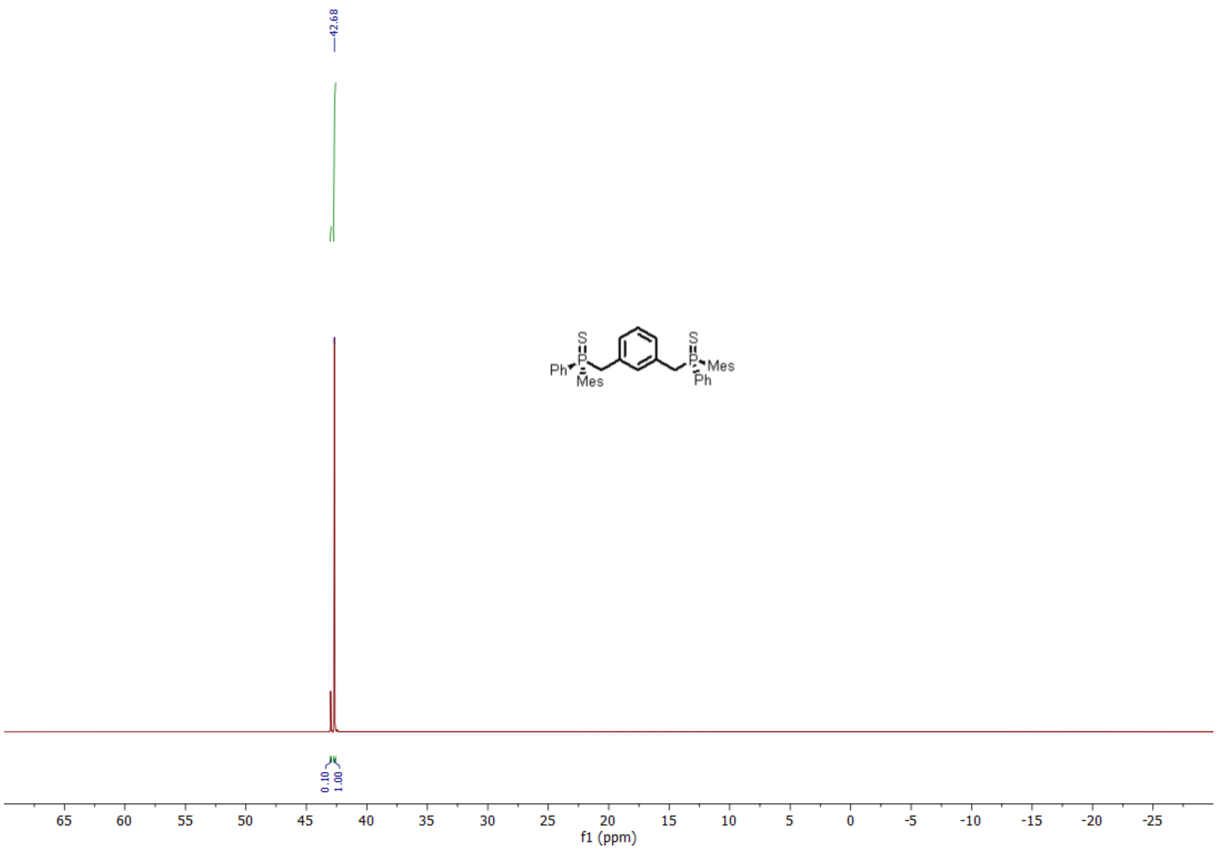

^{1}**H NMR** (400 MHz, $CDCl_3$) spectrum of **6’c**

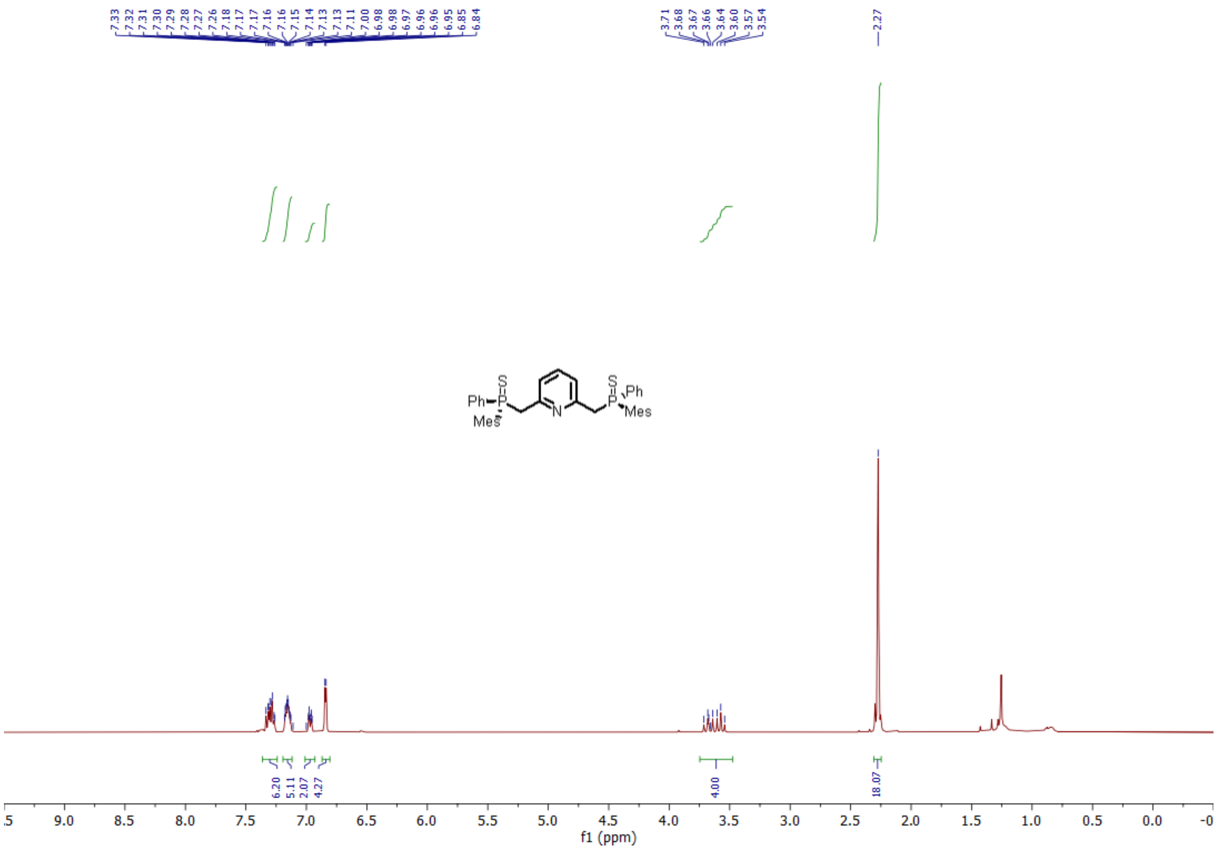

^{13}**C NMR** (101 MHz, $CDCl_3$)spectrum of **6’c**

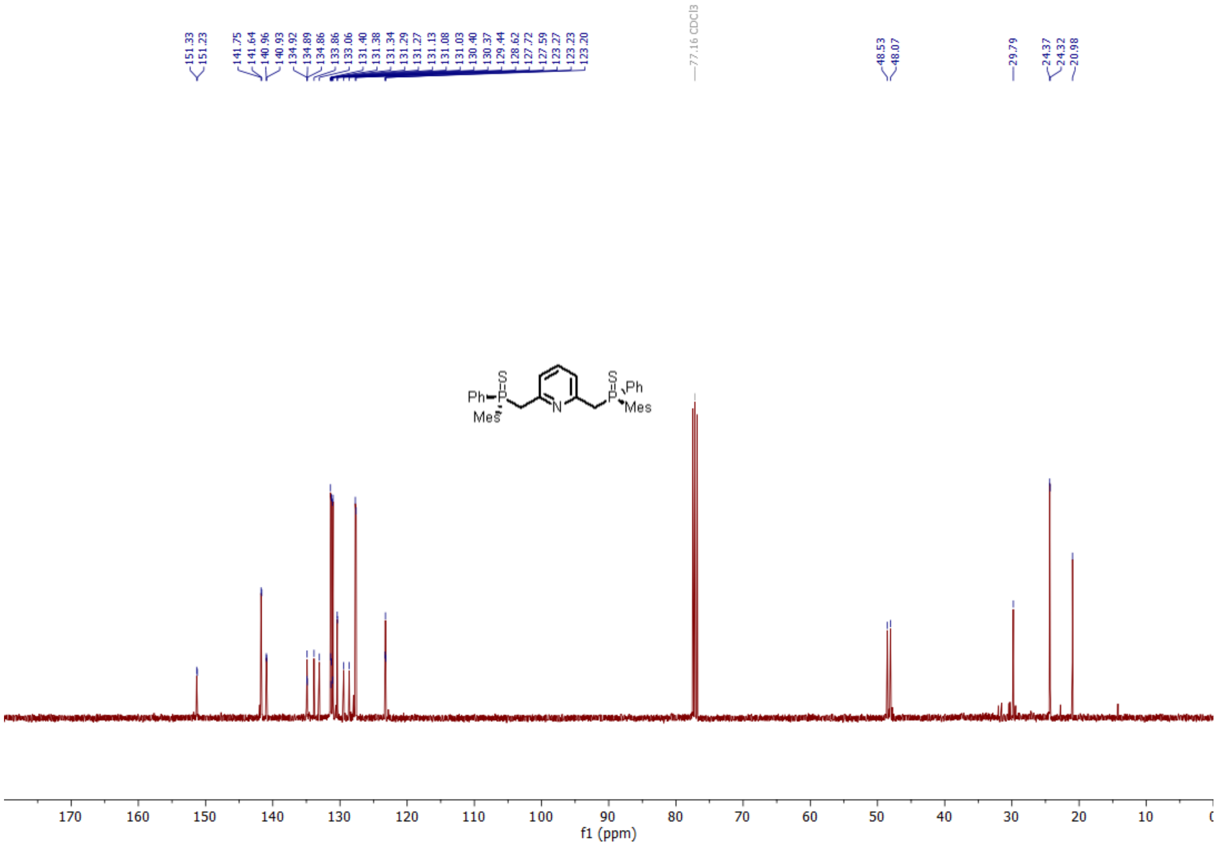

^{31}**P NMR** (162 MHz, $CDCl_3$) spectrum of **6’c**

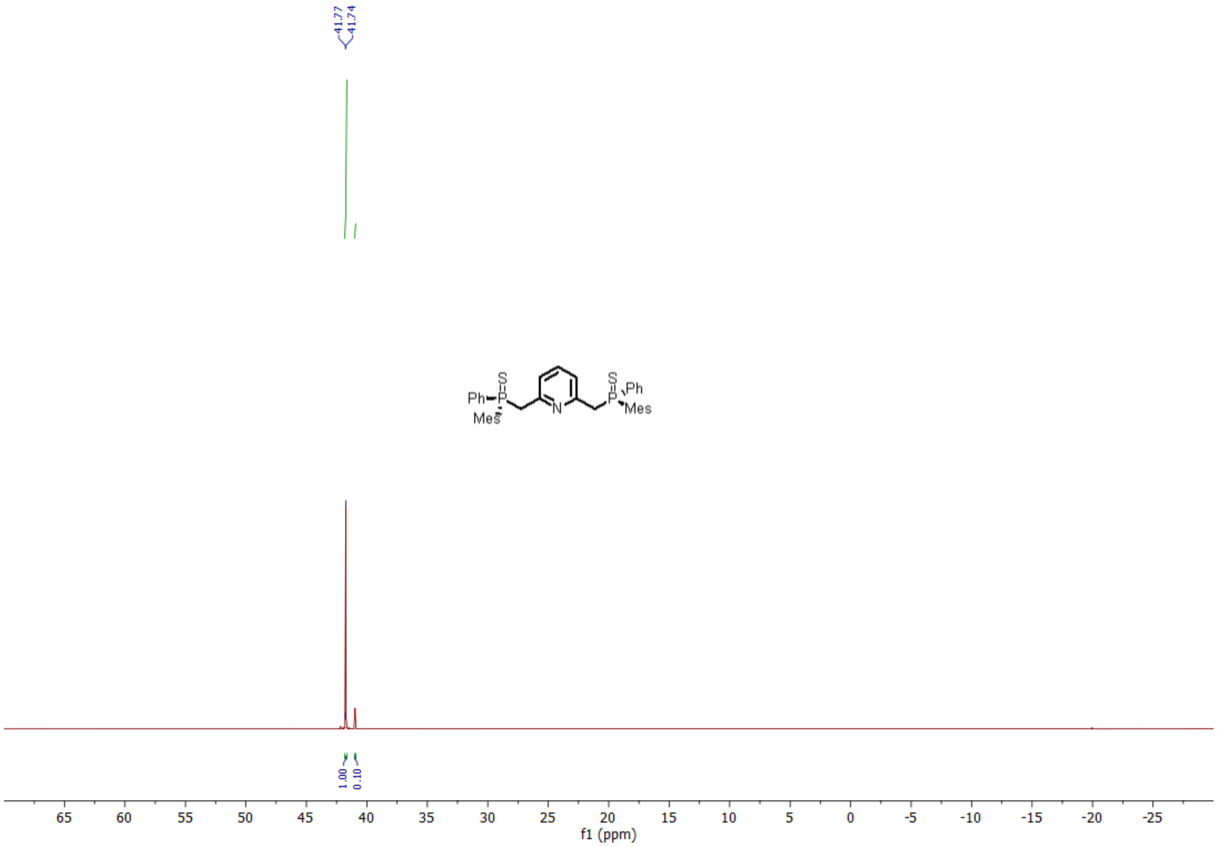

^{1}H NMR (400 MHz, $CDCl_3$) spectrum of **benzyl(tert-butyl)(phenyl)phosphine sulfide**

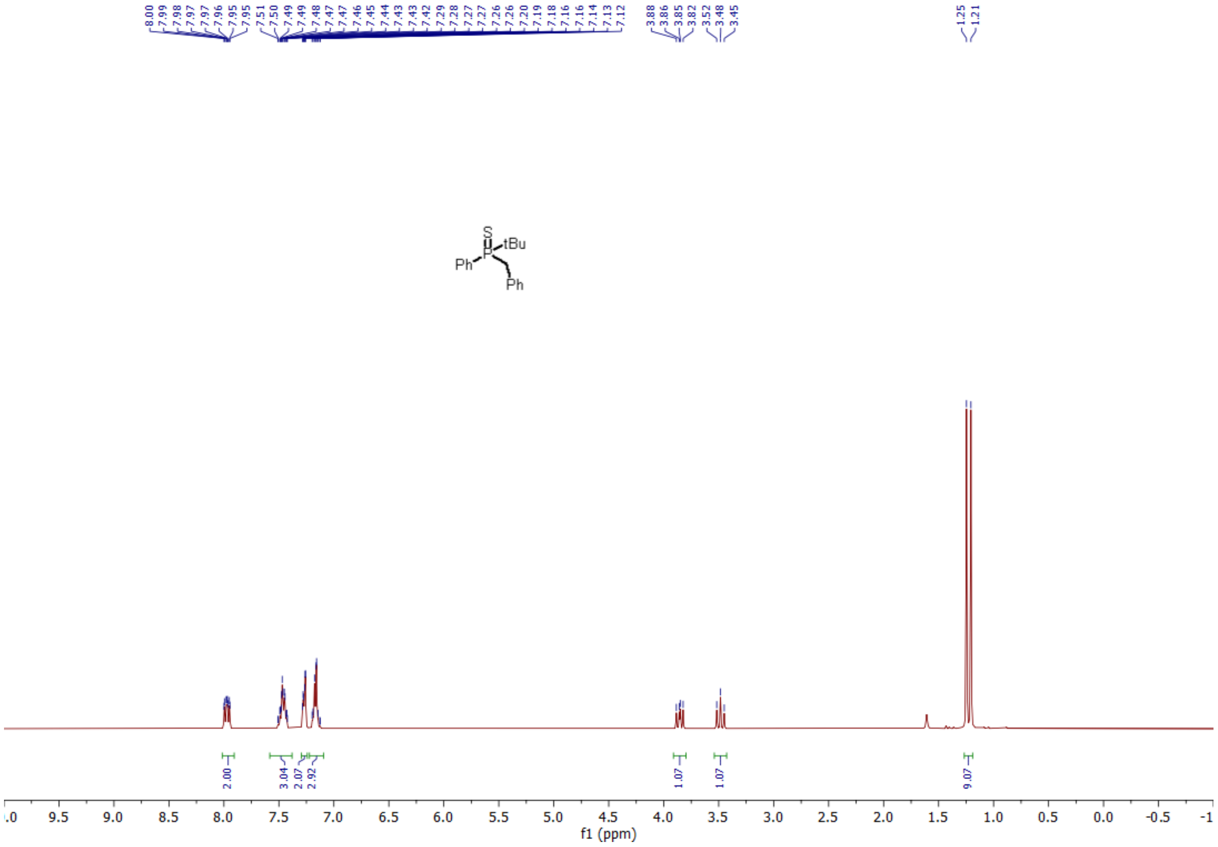

^{13}C NMR (101 MHz, $CDCl_3$)spectrum of **benzyl(tert-butyl)(phenyl)phosphine sulfide**

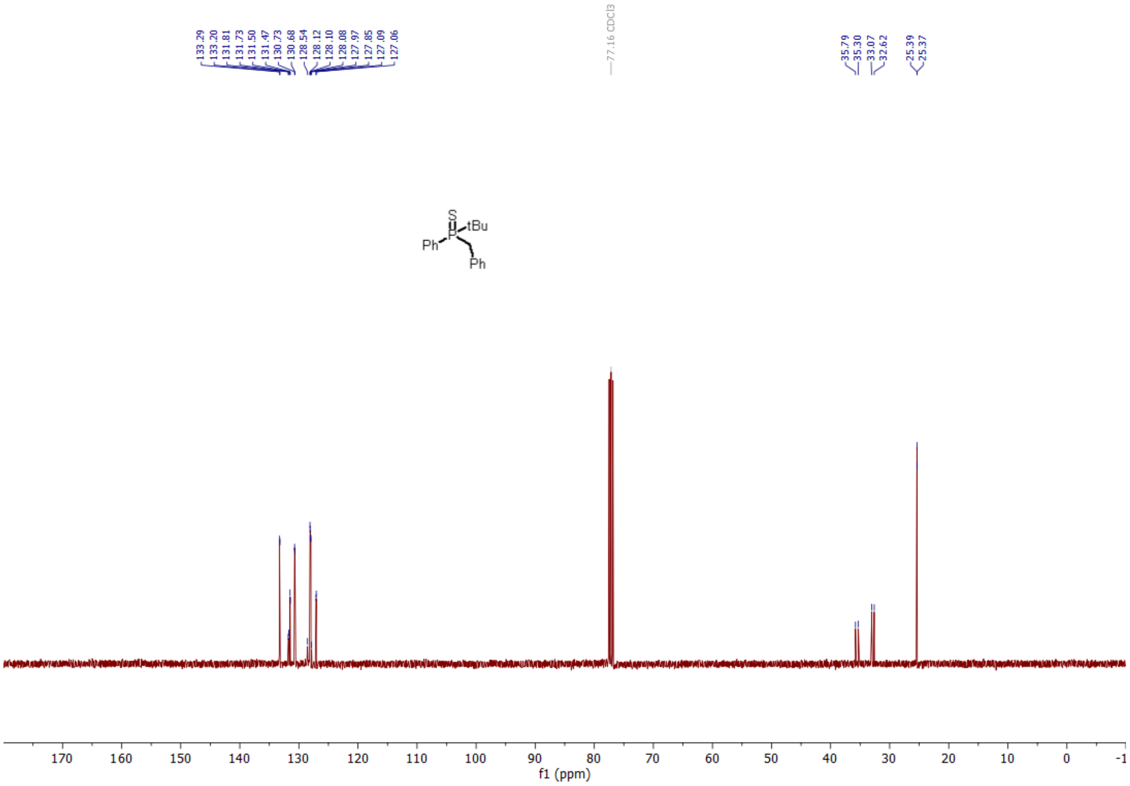

^{31}P NMR (162 MHz, $CDCl_3$) spectrum of **benzyl(tert-butyl)(phenyl)phosphine sulfide**

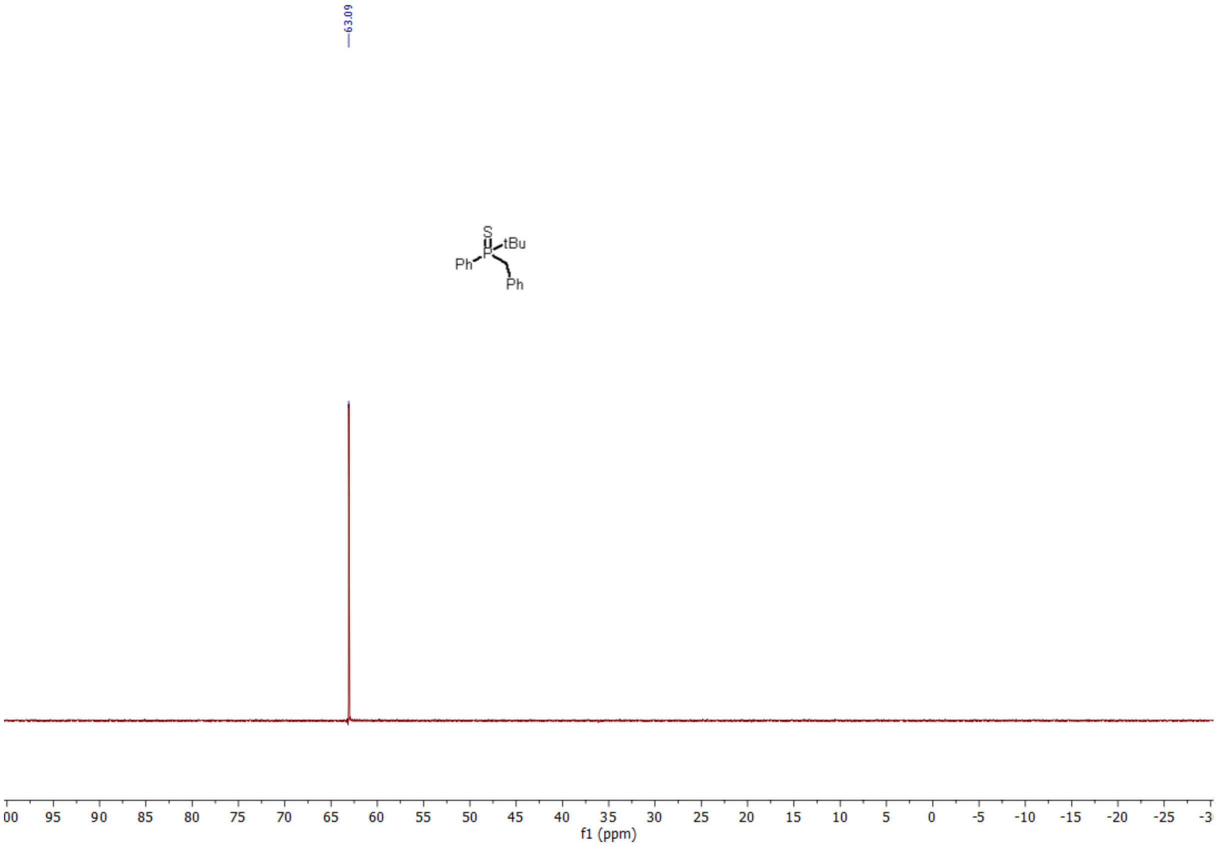

^{1}H NMR (400 MHz, $CDCl_3$) spectrum of **Cu-(I)**

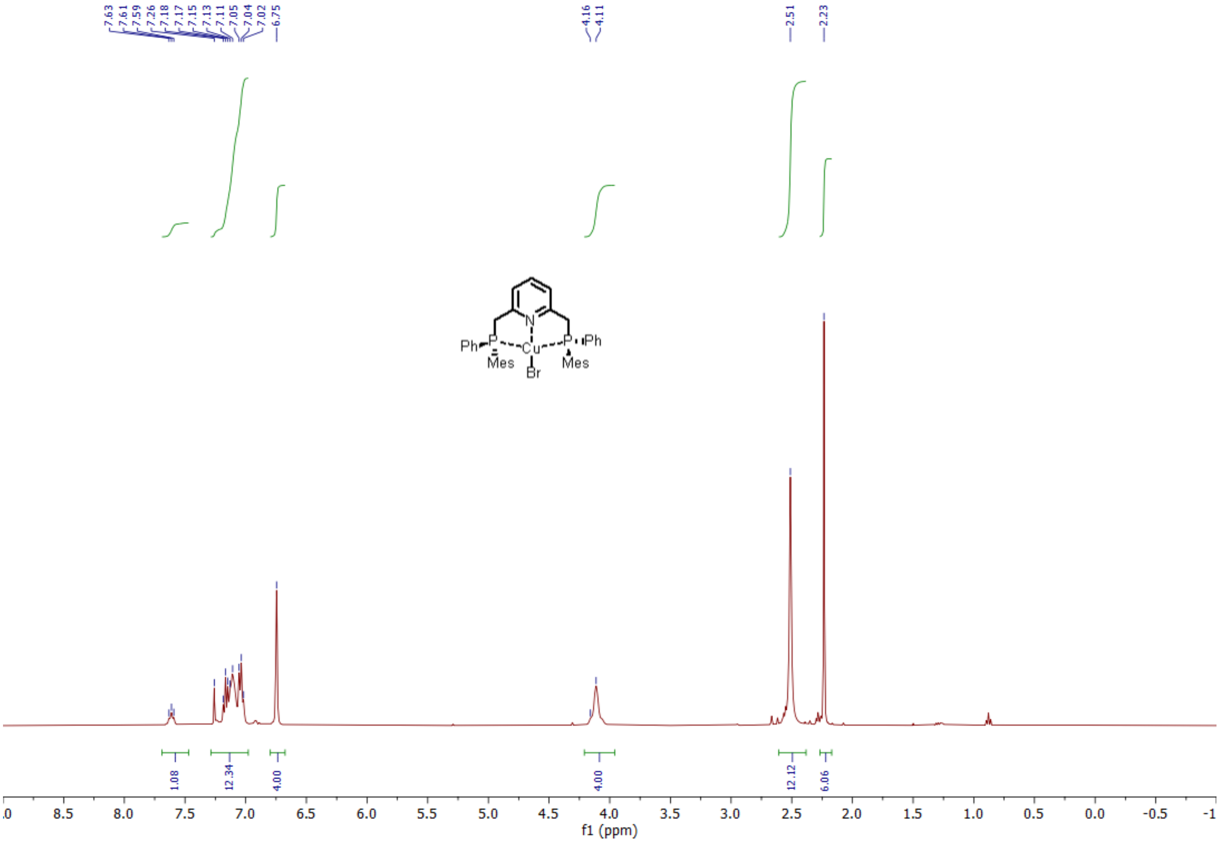

^{13}C NMR (101 MHz, $CDCl_3$)spectrum of **Cu-(I)**

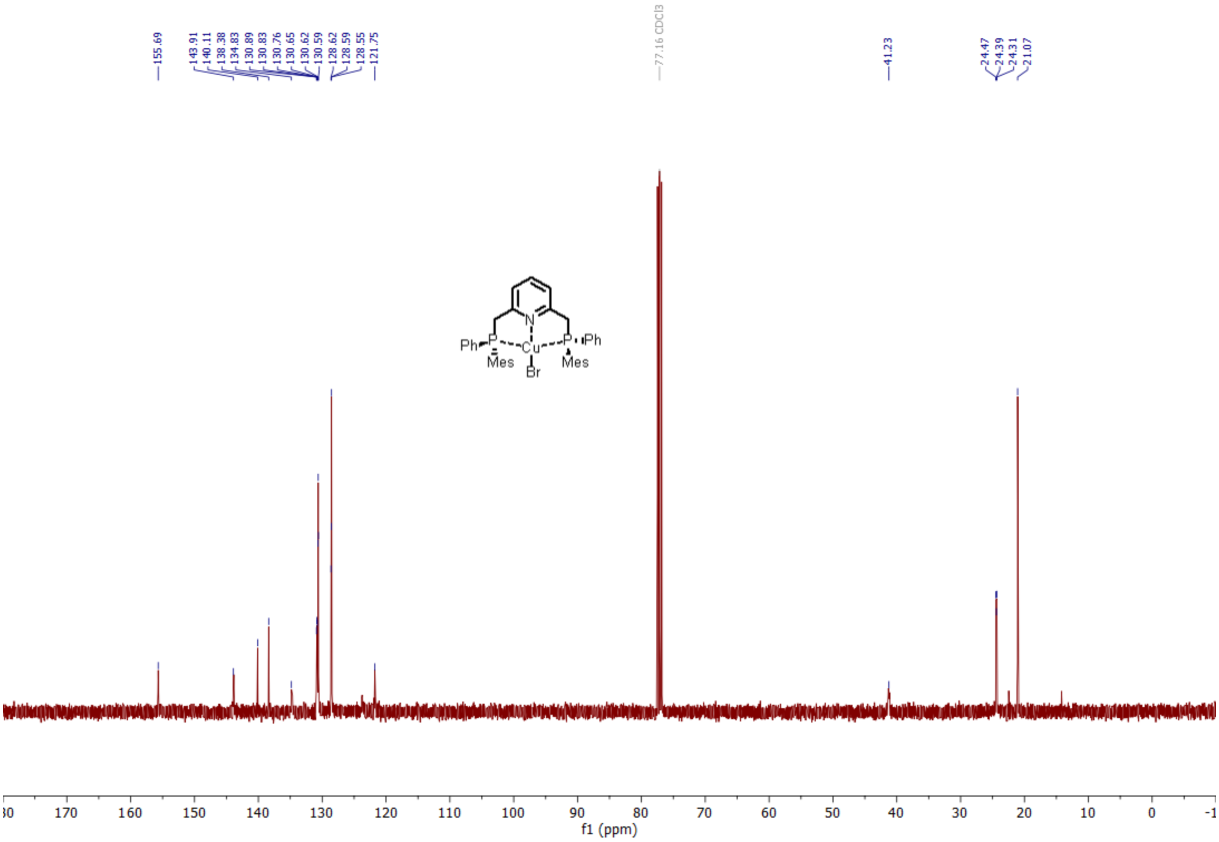

^{31}P NMR (162 MHz, $CDCl_3$) spectrum of **Cu-(I)**

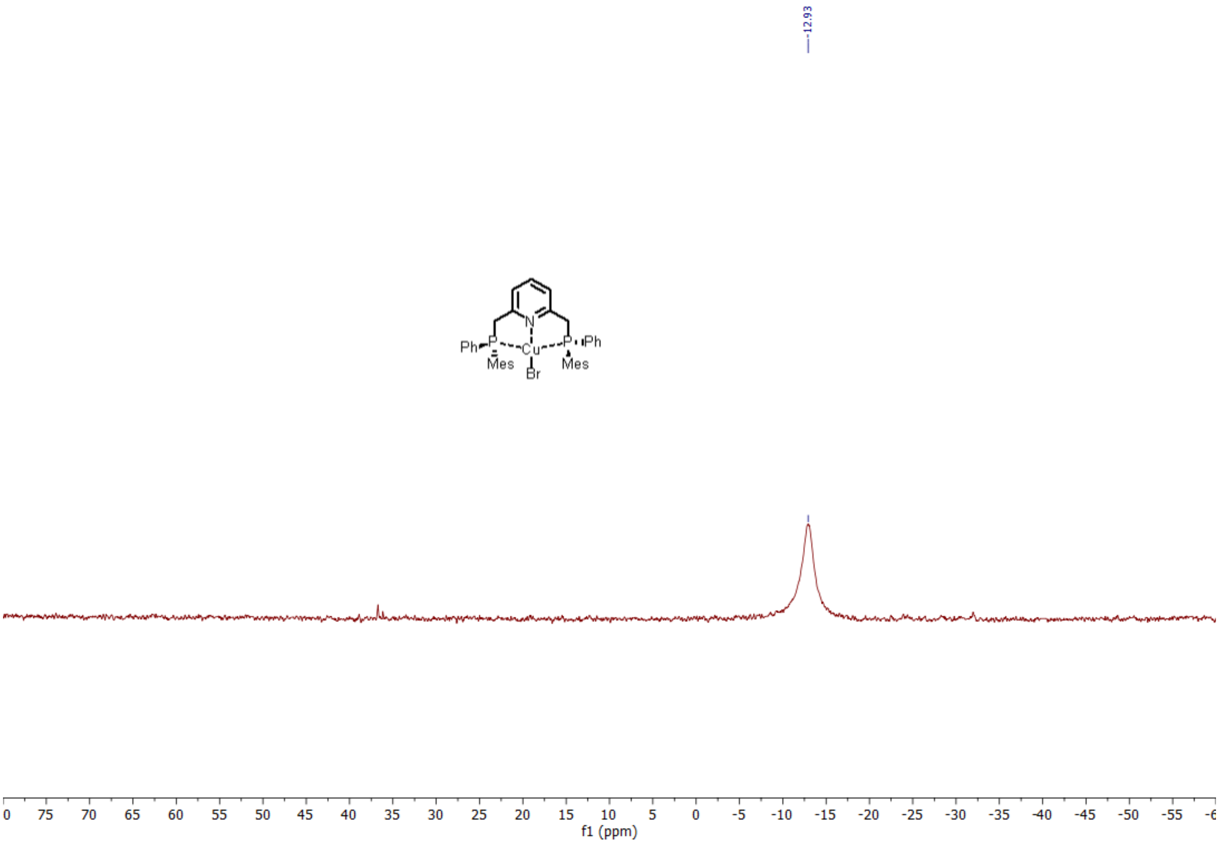

^{31}P NMR (162 MHz, $CDCl_3$) spectrum of **Mn-(I)**

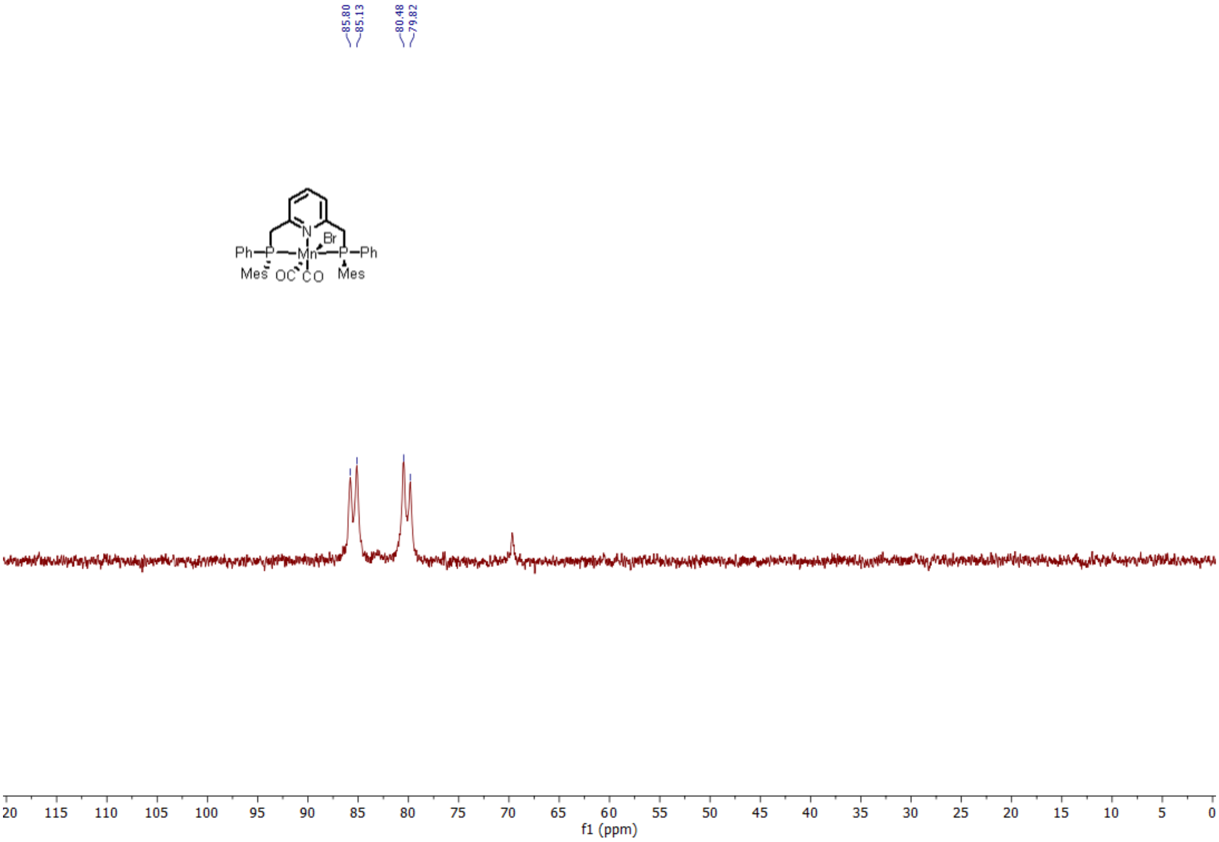

11. Cartesian coordinates and energy reports

Table 5. Energy report of the reported stationary points.[a]

ID	ImFreqs	Stable	SCF	SCF+ZPVE	H	G
**Cat**		Yes	-6825.557609	-6825.017601	-6824.980611	-6825.085098
**HPPhMes**		Yes	-922.6823714	-922.408895	-922.391989	-922.454846
**I**		Yes	-7748.275217	-7747.457237	-7747.403192	-7747.546041
**II**		Yes	-7748.270948	-7747.455423	-7747.400943	-7747.544878
**III**		Yes	-5173.723733	-5172.919922	-5172.868059	-5173.004646
**IV**		Yes	-5173.729266	-5172.925614	-5172.873688	-5173.01017
**TS-IV-V**	-331.801	Yes	-5904.566805	-5903.642199	-5903.582218	-5903.736283
**TS-III-VI**	-316.181	Yes	-5904.568832	-5903.643682	-5903.583278	-5903.739854
**V**		Yes	-5904.631796	-5903.701925	-5903.642259	-5903.796585
**VI**		Yes	-5904.628699	-5903.700283	-5903.640366	-5903.794122

[a] ImFreq correspond to the value of the imaginary frequencies. Stable corresponds to the result of the stability check of the wavefunction. SCF denotes the electronic energies. SCF+ZPVE indicates electronic energies with the zero point energy correction, H respresents the sum of electronic and thermal Enthalpies and G sum of electronic and thermal Free Energies. All energies are reported in Hartrees.

Cat

Fe -3.130818 -0.691436 -0.998224

Mn 1.263259 -0.640702 1.003826

P -0.192256 0.859765 0.131345

O -0.672767 -2.113610 2.660136

O 1.954331 1.160047 3.226003

N 2.815915 0.012546 -0.302992

N 0.970770 -1.938106 -0.744853

C 3.790290 0.865839 0.055529

H 3.694119 1.323574 1.041335

C 4.871540 1.167775 -0.767646

H	5.631991	1.874617	-0.427815
C	4.954255	0.546513	-2.016891
H	5.791150	0.751234	-2.690482
C	3.946327	-0.342754	-2.388799
H	3.970543	-0.849831	-3.356634
C	2.884133	-0.580081	-1.510781
H	1.475170	-2.740996	-0.352658
C	1.722252	-1.450352	-1.914429
H	1.038360	-0.837957	-2.527364
H	2.068616	-2.276050	-2.559333
C	-0.422938	-2.372574	-1.051132
H	-0.847363	-2.647168	-0.075289
C	-0.461913	-3.620661	-1.939644
H	-0.113066	-3.410018	-2.964841
H	-1.490464	-4.013381	-2.000920
H	0.173810	-4.412853	-1.506735
C	-1.239390	-1.225979	-1.619933
C	-2.153258	-1.322749	-2.716814
H	-2.390912	-2.229423	-3.269530
C	-2.747683	-0.045085	-2.935759
H	-3.510417	0.185541	-3.679287
C	-2.205129	0.863075	-1.985185
H	-2.464900	1.915678	-1.890524

C	-1.263540	0.150613	-1.162163
C	-3.562141	-1.185276	0.965346
H	-2.818855	-1.263630	1.756780
C	-3.966020	-2.240685	0.090416
H	-3.583200	-3.261859	0.099821
C	-4.925388	-1.710565	-0.828950
H	-5.402054	-2.256396	-1.643546
C	-5.114630	-0.327197	-0.519044
H	-5.759790	0.366651	-1.058629
C	-4.271167	-0.000912	0.587368
H	-4.157649	0.982492	1.042184
C	-1.350779	1.799597	1.220723
C	-1.641619	1.336935	2.512805
H	-1.150001	0.444716	2.901753
C	-2.570505	2.006923	3.317326
H	-2.788808	1.628761	4.320476
C	-3.215816	3.152247	2.841018
H	-3.944564	3.674492	3.468298
C	-2.916774	3.634386	1.560234
H	-3.407498	4.537014	1.183717
C	-1.986547	2.966877	0.759535
H	-1.749298	3.367598	-0.229374
C	0.699455	2.226174	-0.730087

C	1.339441	3.195941	0.063201
H	1.213648	3.181550	1.150447
C	2.145220	4.174052	-0.523325
H	2.634329	4.923514	0.106411
C	2.334119	4.189161	-1.911446
H	2.970404	4.951361	-2.371317
C	1.707087	3.224238	-2.705918
H	1.849604	3.228117	-3.790867
C	0.891822	2.248322	-2.119223
H	0.402767	1.506032	-2.755504
C	0.067417	-1.505848	2.000819
C	1.682582	0.438406	2.356730
Br	3.042413	-2.513590	1.694570

HPPhMes

P	-0.714807	1.193998	-1.235752
C	-2.008329	0.352821	-0.193291
C	-1.763935	-0.103045	1.114367
H	-0.771723	0.024509	1.558327
C	-2.775836	-0.727434	1.850435
H	-2.570810	-1.077949	2.867080
C	-4.048154	-0.903936	1.290739

H	-4.838868	-1.394094	1.867308
C	-4.301185	-0.456323	-0.010245
H	-5.290565	-0.595972	-0.457264
C	-3.284660	0.162113	-0.748969
H	-3.488087	0.495543	-1.772323
C	0.857095	0.497882	-0.540914
C	1.774940	1.247805	0.238675
C	2.961988	0.633074	0.668291
H	3.665564	1.214591	1.273915
C	3.275745	-0.695230	0.356497
C	2.356371	-1.419333	-0.411991
H	2.576218	-2.462072	-0.666167
C	1.160574	-0.849740	-0.869506
H	-0.726877	2.440728	-0.549954
C	1.531625	2.687222	0.642686
H	1.438276	3.354942	-0.232842
H	0.603568	2.795004	1.233447
H	2.364405	3.065273	1.258553
C	0.208024	-1.701833	-1.679120
H	-0.143657	-1.175438	-2.585695
H	0.686826	-2.643505	-1.995389
H	-0.694098	-1.962254	-1.094839
C	4.577264	-1.316104	0.805750

H	5.365609	-1.178089	0.040577
H	4.945093	-0.856379	1.739474
H	4.472059	-2.402442	0.971644

I

C	-3.289074	-2.626987	-1.919368
C	-3.524084	-1.949776	-0.695196
C	-4.497634	-2.466226	0.208288
C	-5.143709	-3.670926	-0.102943
C	-4.883751	-4.380533	-1.280448
C	-3.968470	-3.827157	-2.179885
P	-2.610862	-0.401546	-0.229706
C	-3.752481	0.979438	-0.640822
C	-4.147212	1.872287	0.370965
C	-4.927985	2.988816	0.054770
C	-5.324411	3.223464	-1.266291
C	-4.939679	2.332405	-2.275754
C	-4.155104	1.217504	-1.965920
Mn	-0.283657	0.035868	-0.538216
C	-0.374454	0.210861	-2.304560
O	-0.379714	0.393227	-3.451586
P	1.962048	0.598840	-0.689551

C	2.193510	2.403475	-0.992978
C	1.859752	2.907002	-2.263989
C	1.904789	4.279197	-2.517779
C	2.273236	5.170618	-1.501908
C	2.604077	4.678250	-0.235925
C	2.567486	3.301488	0.017725
N	-0.775988	2.087188	-0.123164
C	-1.284297	2.933965	-1.037590
C	-1.691568	4.226577	-0.727205
C	-1.586005	4.659156	0.596839
C	-1.058006	3.785280	1.545977
C	-0.643255	2.509338	1.151551
C	0.052774	1.590129	2.117952
N	-0.060102	0.176773	1.709699
C	0.965812	-0.722715	2.331745
C	2.379533	-0.239138	2.063543
C	3.442140	-0.214342	3.021781
C	4.619349	0.290283	2.394784
C	4.304245	0.593252	1.041824
C	2.917365	0.276065	0.820508
Fe	3.949514	-1.403013	1.396539
C	4.694045	-2.569608	-0.144147
C	3.412558	-3.054988	0.267904

C	3.495448	-3.407512	1.650111
C	4.829103	-3.142355	2.094391
C	5.569109	-2.627098	0.984164
C	-0.013806	-1.724599	-0.570689
O	0.100921	-2.880927	-0.556179
C	2.980060	-0.146926	-2.038295
C	2.575069	-1.334408	-2.666031
C	3.383888	-1.941917	-3.632540
C	4.607332	-1.365473	-3.987557
C	5.011814	-0.169511	-3.381199
C	4.201261	0.438630	-2.419121
C	0.711114	-0.949557	3.826303
Br	-2.844298	0.418285	3.805565
H	-1.373356	2.552686	-2.055130
H	-2.097079	4.869157	-1.511147
H	-1.912916	5.661712	0.886338
H	-0.955962	4.081803	2.592457
H	-0.968528	-0.131416	2.086285
H	1.119234	1.868869	2.136945
H	-0.353230	1.737637	3.131518
H	-2.804648	-0.412819	1.170890
H	0.829356	-1.688026	1.824336
H	0.907413	-0.038689	4.416909

H	1.365929	-1.754103	4.201024
H	-0.338029	-1.241668	3.993693
H	3.379642	-0.561529	4.050805
H	5.598804	0.390424	2.862079
H	4.994380	0.989323	0.299338
H	2.520629	-3.116948	-0.351903
H	2.676347	-3.780912	2.265719
H	5.205287	-3.276365	3.108878
H	6.608127	-2.297320	1.006190
H	4.943590	-2.190785	-1.134841
H	1.625984	-1.801172	-2.402211
H	3.053245	-2.870299	-4.107397
H	5.242021	-1.841577	-4.740976
H	5.961963	0.295287	-3.661016
H	4.522708	1.381910	-1.970083
H	1.558374	2.222883	-3.062543
H	1.644392	4.655095	-3.511852
H	2.302662	6.246541	-1.698581
H	2.896021	5.366364	0.563109
H	2.842940	2.934261	1.009423
H	-3.835049	1.700236	1.406902
H	-5.224242	3.681680	0.848428
H	-5.932608	4.099701	-1.510867

H	-5.247361	2.507794	-3.311153
H	-3.858277	0.534776	-2.767216
C	-4.906758	-1.764541	1.484472
H	-5.888333	-4.061895	0.598385
C	-5.565557	-5.696823	-1.563454
H	-3.778217	-4.340947	-3.127923
C	-2.354484	-2.121252	-2.994135
H	-5.767769	-2.277141	1.944449
H	-4.099713	-1.724894	2.236288
H	-5.200612	-0.716733	1.298386
H	-2.538994	-2.648793	-3.944018
H	-2.475967	-1.046159	-3.189156
H	-1.297970	-2.286782	-2.729067
H	-5.612570	-5.906005	-2.645909
H	-5.013877	-6.531833	-1.090176
H	-6.592545	-5.715864	-1.158973

II

C	2.324033	-2.294224	2.924660
C	3.024563	-2.144429	1.717696
C	4.159280	-2.941952	1.482843
C	4.585210	-3.867809	2.438906

C	3.881263	-4.009352	3.641708
C	2.751053	-3.221438	3.882227
P	2.482898	-0.968973	0.404664
C	3.903035	0.186173	0.124103
C	4.456525	0.303578	-1.182941
C	5.432180	1.278972	-1.419809
C	5.895154	2.140142	-0.416183
C	5.361237	1.987940	0.863929
C	4.367561	1.039062	1.153845
Mn	0.309355	-0.061024	0.440449
C	0.552402	0.624797	2.063569
O	0.635147	1.071570	3.132912
P	-1.716108	1.057551	0.418824
C	-1.442606	2.856392	0.129626
C	-1.013035	3.660035	1.200947
C	-0.645842	4.990995	0.986005
C	-0.688971	5.532393	-0.304261
C	-1.109929	4.737282	-1.375417
C	-1.488073	3.407590	-1.160376
N	1.141466	1.492319	-0.687750
C	1.799199	2.539706	-0.155989
C	2.463135	3.487248	-0.925964
C	2.460550	3.340206	-2.314581

C	1.768471	2.265353	-2.870065
C	1.107828	1.365102	-2.029379
C	0.261048	0.258361	-2.598795
N	-0.069448	-0.782757	-1.604124
C	-1.386720	-1.449686	-1.900604
C	-2.548158	-0.472192	-1.894342
C	-3.617230	-0.449997	-2.848113
C	-4.549204	0.562355	-2.474516
C	-4.069756	1.190441	-1.292775
C	-2.826934	0.565520	-0.924941
Fe	-4.337374	-0.826185	-0.939829
C	-5.218916	-1.185525	0.901035
C	-4.178308	-2.136670	0.653383
C	-4.506046	-2.849076	-0.541334
C	-5.748617	-2.339224	-1.033821
C	-6.188810	-1.313207	-0.140184
C	-0.342640	-1.586531	1.095737
O	-0.754956	-2.601627	1.480705
C	-2.781347	1.063133	1.927024
C	-2.635466	0.075756	2.912960
C	-3.493844	0.039078	4.017564
C	-4.506382	0.993583	4.153019
C	-4.649760	1.993936	3.183188

C	-3.790610	2.031356	2.082231
C	-1.343656	-2.253610	-3.205925
Br	1.888217	-3.779859	-2.130896
H	1.787521	2.617250	0.930300
H	2.983273	4.312520	-0.436317
H	2.987192	4.052954	-2.954927
H	1.732939	2.114918	-3.951722
H	0.621028	-1.545240	-1.698166
H	-0.682159	0.715743	-2.946422
H	0.741672	-0.174212	-3.492711
H	2.629872	-1.853950	-0.690770
H	-1.525069	-2.161417	-1.075454
H	-1.283738	-1.594761	-4.089223
H	-2.257681	-2.863968	-3.297256
H	-0.475124	-2.931340	-3.201718
H	-3.731033	-1.121867	-3.695748
H	-5.483316	0.790202	-2.987560
H	-4.558471	2.001987	-0.756839
H	-3.275739	-2.271304	1.247985
H	-3.896987	-3.620394	-1.014079
H	-6.252918	-2.653857	-1.947782
H	-7.087933	-0.707285	-0.255037
H	-5.246808	-0.469010	1.720778

H	-1.854739	-0.680001	2.827950
H	-3.367658	-0.741601	4.773398
H	-5.179368	0.963537	5.015167
H	-5.433091	2.750961	3.285005
H	-3.905867	2.828877	1.343958
H	-0.957869	3.243440	2.210918
H	-0.316754	5.604997	1.829791
H	-0.393435	6.572025	-0.474386
H	-1.144362	5.151455	-2.387527
H	-1.816631	2.799932	-2.007733
C	4.056167	-0.584428	-2.342249
H	5.848751	1.369863	-2.428432
C	6.911392	3.211793	-0.726700
H	5.713438	2.638543	1.671901
C	3.831768	1.035297	2.569061
H	4.713066	-2.841472	0.544197
H	5.468188	-4.483817	2.242860
H	4.214116	-4.735161	4.389994
H	2.196099	-3.326000	4.819410
H	1.444055	-1.681674	3.131769
H	4.581070	-0.276147	-3.261014
H	4.289161	-1.647329	-2.157209
H	2.976297	-0.547265	-2.556448

H	4.658441	1.142860	3.292654
H	3.149573	1.888401	2.732113
H	3.279985	0.125812	2.833490
H	7.412151	3.576015	0.186416
H	7.684107	2.846204	-1.425753
H	6.424320	4.081543	-1.208298

III

C	-3.704516	0.679447	2.469265
C	-2.460223	0.157704	2.069659
C	-1.873799	-0.853238	2.845719
C	-2.527645	-1.355091	3.977145
C	-3.775196	-0.847036	4.351666
C	-4.360089	0.177557	3.596440
P	-1.634452	0.751493	0.523367
C	-2.712156	0.131813	-0.813346
C	-2.252638	-0.549796	-2.007633
C	-3.394140	-0.752009	-2.846740
C	-4.545849	-0.223339	-2.191996
C	-4.132949	0.320420	-0.943976
C	-0.835124	-0.989638	-2.325468
C	-0.672374	-1.441129	-3.781368

Fe	-3.659527	-1.683648	-1.011418
C	-5.120336	-2.923877	-0.221142
C	-4.135607	-2.626696	0.771039
C	-2.870297	-3.089303	0.289055
C	-3.073658	-3.668506	-1.001532
C	-4.465074	-3.565872	-1.318598
Mn	0.582938	0.340566	0.170425
C	0.484969	-1.387740	0.555777
O	0.455823	-2.528260	0.798113
P	2.887776	-0.157281	-0.624768
C	4.232383	0.993931	-0.121868
C	4.180374	2.316690	-0.625989
C	5.168574	3.257676	-0.330269
C	6.271449	2.904881	0.459490
C	6.367714	1.590647	0.932078
C	5.368160	0.652826	0.648752
C	3.522901	-1.831873	-0.086990
C	3.679499	-2.240896	1.263387
C	4.140026	-3.535754	1.555761
C	4.434072	-4.467085	0.556158
C	4.264736	-4.061901	-0.773427
C	3.821717	-2.774334	-1.112249
C	3.323019	-1.359889	2.439001

C	3.681453	-2.447395	-2.586871
C	4.888141	-5.868121	0.893235
C	0.997271	0.761858	1.836472
O	1.237228	1.086630	2.930301
N	0.841463	2.280682	-0.553323
C	1.270255	3.322640	0.183871
C	1.498381	4.588294	-0.348542
C	1.301451	4.786004	-1.716651
C	0.852878	3.710193	-2.484809
C	0.608599	2.479751	-1.869201
C	-0.032604	1.348083	-2.627867
N	0.154558	0.058110	-1.944197
C	-2.021464	2.556052	0.582929
C	-1.653866	3.271280	1.737870
C	-1.802529	4.658733	1.793494
C	-2.308126	5.356522	0.689047
C	-2.666783	4.655106	-0.466318
C	-2.525567	3.262752	-0.519458
H	1.446179	3.127443	1.241691
H	1.841651	5.394006	0.304007
H	1.489102	5.759525	-2.177786
H	0.670406	3.819987	-3.556719
H	1.093286	-0.285002	-2.189102

H	-1.115615	1.555729	-2.679636
H	0.332511	1.326468	-3.669374
H	-0.588941	-1.847612	-1.684478
H	-0.962047	-0.651409	-4.495296
H	-1.296551	-2.328512	-3.978689
H	0.378088	-1.722260	-3.974207
H	-3.395893	-1.260984	-3.808554
H	-5.568151	-0.261338	-2.568006
H	-4.782906	0.792413	-0.209769
H	-1.913156	-2.985959	0.796738
H	-2.297943	-4.083592	-1.646085
H	-4.936727	-3.889335	-2.246873
H	-6.179519	-2.670400	-0.168483
H	-4.306494	-2.106882	1.713199
H	-0.902774	-1.263929	2.569288
H	-2.056452	-2.147690	4.566154
H	-4.288300	-1.241288	5.234106
H	-5.330519	0.590639	3.888186
H	-4.165889	1.492660	1.903211
H	-1.242145	2.738278	2.600683
H	-1.515403	5.199108	2.700791
H	-2.420174	6.444255	0.729655
H	-3.061571	5.191339	-1.334793

H	-2.820815	2.729856	-1.427158
H	3.364314	2.604974	-1.292487
H	5.080750	4.272652	-0.732983
H	7.050803	3.638459	0.688630
H	7.232216	1.287264	1.533004
H	5.484588	-0.366406	1.024401
H	4.494571	-4.771589	-1.576356
H	4.264743	-3.825815	2.605649
H	4.032204	-3.289989	-3.208330
H	2.631487	-2.240185	-2.862706
H	4.255518	-1.547124	-2.864528
H	3.943439	-1.607085	3.318438
H	3.435395	-0.289659	2.225359
H	2.268469	-1.519095	2.727160
H	5.372179	-5.911198	1.884473
H	4.030794	-6.568689	0.916810
H	5.600682	-6.255092	0.143366

IV

C	3.597317	-0.601976	2.705763
C	2.625297	0.169117	2.042374
C	2.523654	1.534618	2.348254

C	3.391110	2.124754	3.274577
C	4.368770	1.354651	3.912050
C	4.466374	-0.013580	3.628331
P	1.539421	-0.583142	0.748133
C	2.680132	-0.915829	-0.635668
C	2.426888	-0.576236	-2.022268
C	3.502362	-1.119249	-2.794837
C	4.416992	-1.771359	-1.915633
C	3.917170	-1.649486	-0.589279
C	1.254910	0.213931	-2.575930
C	1.175047	0.149651	-4.105434
Fe	4.217350	0.226835	-1.387203
C	6.083441	1.029753	-0.974616
C	5.125778	1.490041	-0.018984
C	4.095326	2.185667	-0.727383
C	4.415470	2.151712	-2.119762
C	5.644640	1.435943	-2.273987
Mn	-0.397634	0.456303	0.124810
C	0.353918	2.033369	-0.165060
O	0.819708	3.079120	-0.394859
P	-2.458162	1.425553	-0.819119
C	-3.944932	0.308115	-0.656673
C	-4.351504	-0.436683	-1.801462

C	-5.416174	-1.345028	-1.707683
C	-6.120253	-1.550123	-0.516664
C	-5.736369	-0.794459	0.594698
C	-4.668848	0.115522	0.554740
C	-3.684329	-0.285489	-3.153241
C	-7.221511	-2.579687	-0.425304
C	-4.348189	0.854428	1.833698
C	-3.071636	2.971652	-0.019654
C	-2.297499	3.762270	0.854282
C	-2.776675	4.976794	1.360136
C	-4.053573	5.436076	1.020002
C	-4.842985	4.662596	0.156810
C	-4.355343	3.459963	-0.358871
C	-0.763312	0.877902	1.800486
O	-0.963545	1.154288	2.916223
N	-1.400911	-1.375828	0.084970
C	-2.195366	-1.824699	1.072305
C	-2.973582	-2.972660	0.958132
C	-2.942378	-3.688531	-0.239189
C	-2.108107	-3.235013	-1.261623
C	-1.341832	-2.086179	-1.061424
C	-0.344608	-1.623089	-2.088571
N	-0.029125	-0.193261	-1.929360

C	1.219049	-2.253991	1.463300
C	0.657863	-2.318033	2.752345
C	0.262668	-3.541888	3.296299
C	0.408051	-4.721109	2.553831
C	0.956980	-4.665483	1.269006
C	1.362807	-3.439430	0.726962
H	-2.213273	-1.231246	1.986216
H	-3.606994	-3.279376	1.793006
H	-3.557569	-4.582014	-0.376422
H	-2.046130	-3.763363	-2.216173
H	-0.774950	0.363006	-2.364400
H	0.587031	-2.193081	-1.932917
H	-0.690826	-1.868704	-3.107147
H	1.386150	1.267837	-2.295683
H	1.113277	-0.888326	-4.474094
H	2.065463	0.620451	-4.554285
H	0.288330	0.702957	-4.461437
H	3.628506	-1.018256	-3.870943
H	5.352743	-2.248266	-2.206932
H	4.393165	-2.037919	0.309179
H	3.202665	2.629913	-0.291158
H	3.809744	2.567287	-2.925895
H	6.141130	1.210455	-3.218127

H	6.972792	0.438048	-0.755989
H	5.152219	1.313358	1.055731
H	1.769925	2.151970	1.859135
H	3.300175	3.192351	3.496075
H	5.049179	1.816182	4.634136
H	5.220973	-0.626914	4.130162
H	3.674482	-1.674585	2.509634
H	0.522801	-1.400702	3.334221
H	-0.168785	-3.574807	4.301480
H	0.092482	-5.679797	2.976574
H	1.074424	-5.581072	0.681193
H	1.799706	-3.416776	-0.274843
H	-5.707323	-1.911287	-2.599807
H	-6.273070	-0.933096	1.540510
H	-4.987537	2.875031	-1.035281
H	-5.846456	5.003006	-0.121434
H	-4.431535	6.381142	1.422738
H	-2.147263	5.561211	2.040232
H	-1.311407	3.419035	1.169588
H	-4.291543	-0.756704	-3.945561
H	-3.520633	0.774376	-3.410023
H	-2.692929	-0.769893	-3.181296
H	-4.711515	0.292697	2.711785

H	-3.271539	1.030017	1.952302
H	-4.826298	1.850846	1.849741
H	-7.963121	-2.316508	0.349239
H	-7.752078	-2.695325	-1.386975
H	-6.810868	-3.573681	-0.159506

TS-IV-V

C	0.848215	-3.934614	-1.249041
C	2.095673	-3.631913	-0.664497
C	2.454029	-4.284756	0.531935
C	1.579443	-5.192471	1.132486
C	0.338412	-5.472282	0.548237
C	-0.022072	-4.842607	-0.651090
C	3.004664	-2.700842	-1.320236
P	2.428317	-0.435506	0.248164
C	3.688295	0.909555	-0.065149
C	4.188927	1.741478	0.977956
C	5.092383	2.771655	0.674154
C	5.510415	3.044324	-0.630171
C	5.005883	2.230018	-1.648582
C	4.116439	1.176452	-1.395771
C	3.799198	1.593432	2.431992

C	6.423408	4.206407	-0.938875
C	3.683252	0.352761	-2.590715
C	3.162405	-1.310583	1.683904
C	2.386630	-1.836536	2.733514
C	2.962599	-2.622074	3.738696
C	4.332580	-2.900483	3.725011
C	5.123941	-2.376470	2.692674
C	4.546369	-1.597207	1.688791
Mn	0.121975	0.345451	0.442907
N	0.818289	2.121851	-0.415400
C	0.806317	2.198949	-1.762248
C	1.401318	3.258256	-2.448968
C	2.006527	4.287106	-1.726861
C	1.989020	4.220792	-0.333175
C	1.392125	3.121741	0.276612
C	0.052900	1.123184	-2.495104
N	-0.082357	-0.101766	-1.684081
C	-1.200386	-0.986487	-2.137228
C	-0.953405	-1.544367	-3.545099
C	-0.370884	-1.290176	0.936179
O	-0.690012	-2.354789	1.279373
C	0.308317	0.825435	2.135222
O	0.382084	1.135880	3.255534

P	-2.022477	1.159965	0.431102
C	-3.014479	0.588496	-0.984474
C	-4.351797	0.997266	-1.324889
C	-4.701889	0.398169	-2.566048
C	-3.594749	-0.384203	-3.007778
C	-2.543848	-0.286342	-2.040162
Fe	-4.241851	-1.054030	-1.155242
C	-5.997140	-1.945411	-0.509944
C	-5.130458	-1.703000	0.600332
C	-3.911738	-2.416385	0.371341
C	-4.023880	-3.094386	-0.881310
C	-5.313552	-2.803416	-1.427904
C	-2.044091	2.997429	0.274673
C	-1.677269	3.758956	1.399635
C	-1.545499	5.145958	1.306115
C	-1.764589	5.792018	0.082529
C	-2.122371	5.041464	-1.041747
C	-2.264069	3.651459	-0.946554
C	-3.134251	0.899780	1.885544
C	-2.874269	-0.119070	2.814439
C	-3.758444	-0.363824	3.871358
C	-4.912755	0.411462	4.016809
C	-5.171392	1.444836	3.107433

C	-4.285829	1.690726	2.055108
H	1.379554	3.028832	1.362482
H	2.447942	4.995867	0.283695
H	2.485959	5.123037	-2.243334
H	1.387805	3.266420	-3.541454
H	-0.963259	1.501869	-2.699229
H	0.515622	0.925599	-3.476630
H	-1.189754	-1.827663	-1.430129
H	-1.083014	-0.773099	-4.322232
H	-1.655099	-2.367669	-3.756366
H	0.069723	-1.947816	-3.632247
H	-3.575020	-0.987436	-3.912971
H	-5.661619	0.490238	-3.074166
H	-4.989718	1.647522	-0.729557
H	-5.341383	-1.060858	1.455044
H	-3.246993	-3.698119	-1.352216
H	-5.694599	-3.146513	-2.390060
H	-6.990310	-1.518665	-0.652595
H	-1.982271	-0.737319	2.720476
H	-3.540638	-1.166453	4.582206
H	-5.606023	0.217949	4.840956
H	-6.065490	2.065591	3.219486
H	-4.492790	2.514309	1.366994

H	-1.487609	3.263163	2.356907
H	-1.262742	5.724768	2.190681
H	-1.655321	6.878100	0.006881
H	-2.295835	5.537458	-2.001608
H	-2.554983	3.081316	-1.832728
H	5.313377	2.422294	-2.682428
H	5.461991	3.401237	1.491374
H	6.199520	-2.580086	2.667854
H	2.329813	-3.018405	4.539536
H	5.182945	-1.196770	0.893231
H	1.316867	-1.632315	2.778780
H	4.782549	-3.515398	4.510541
H	4.230111	-0.605681	-2.645009
H	2.611779	0.109447	-2.572253
H	3.877362	0.897432	-3.529569
H	4.431213	0.844139	2.941704
H	3.921340	2.552574	2.963857
H	2.762107	1.260047	2.558837
H	7.084058	3.987771	-1.796249
H	5.835896	5.107425	-1.202941
H	7.054860	4.469752	-0.072660
H	-3.037235	-2.418615	1.017968
H	0.774836	-0.652941	-1.774824

H	2.659826	-2.124281	-2.168821
H	0.575018	-3.464244	-2.196283
H	3.421552	-4.071656	0.992583
H	-0.988372	-5.059204	-1.117417
H	1.867985	-5.680886	2.068323
H	-0.346710	-6.180591	1.024221
H	3.951946	-2.436485	-0.863574
Cl	4.149932	-4.080626	-2.831042

TS-III-VI

C	5.687506	0.040277	1.946187
C	4.685323	-0.844909	1.499547
C	5.037815	-1.868380	0.595425
C	6.351127	-1.998132	0.147471
C	7.338098	-1.112058	0.597674
C	7.002080	-0.092288	1.499009
C	3.317104	-0.733464	1.969767
P	2.239773	0.748094	-0.240854
C	3.547352	0.235132	-1.415410
C	3.415234	-0.913229	-2.216270
C	4.458716	-1.352243	-3.038107
C	5.667980	-0.651532	-3.076936

C	5.820278	0.490699	-2.278871
C	4.778978	0.924175	-1.458075
C	2.360974	2.611020	-0.339029
C	2.274453	3.301420	-1.581314
C	2.332621	4.703008	-1.602605
C	2.452191	5.468062	-0.437918
C	2.518684	4.781053	0.778431
C	2.477202	3.380100	0.848833
C	2.111340	2.593701	-2.906368
C	2.590930	2.750893	2.219782
C	2.466251	6.977476	-0.489825
Mn	-0.090797	-0.080223	-0.579827
N	0.521382	-2.083784	-0.075664
C	0.535880	-2.422453	1.229421
C	0.999356	-3.669032	1.665586
C	1.410284	-4.611832	0.726481
C	1.333585	-4.281195	-0.629441
C	0.888147	-3.011538	-0.979611
C	-0.026411	-1.439865	2.224235
N	-0.181766	-0.085429	1.657629
C	-1.271295	0.717739	2.289336
C	-0.959426	1.063768	3.749345
C	0.061605	-0.268542	-2.333338

O	0.139113	-0.424844	-3.484954
C	-0.545927	1.629751	-0.724871
O	-0.820180	2.758223	-0.800613
P	-2.262053	-0.845103	-0.629470
C	-3.154665	-0.594898	0.941173
C	-2.616450	0.033833	2.132617
C	-3.605995	-0.089331	3.159695
C	-4.743247	-0.762740	2.625329
C	-4.471633	-1.076237	1.265371
Fe	-4.353075	0.965480	1.536909
C	-5.410000	2.606576	2.228013
C	-6.123467	1.977759	1.159790
C	-5.289341	1.993473	-0.000275
C	-4.059529	2.635976	0.349205
C	-4.134165	3.014400	1.725354
C	-3.431001	-0.230127	-1.924512
C	-4.637075	-0.908068	-2.182790
C	-5.551643	-0.407714	-3.112498
C	-5.269792	0.774181	-3.809762
C	-4.063288	1.441353	-3.578829
C	-3.148695	0.939597	-2.645254
C	-2.359420	-2.668164	-0.895533
C	-2.169453	-3.164897	-2.198036

C	-2.090685	-4.540206	-2.429586
C	-2.183745	-5.439079	-1.359961
C	-2.362361	-4.952740	-0.060953
C	-2.453001	-3.575408	0.170567
Cl	3.237664	-2.084803	3.881943
H	0.803103	-2.724558	-2.027665
H	1.615752	-4.990039	-1.410827
H	1.775467	-5.592099	1.045110
H	1.050030	-3.873686	2.735780
H	-1.023790	-1.804327	2.524620
H	0.603650	-1.438366	3.130795
H	-1.289857	1.656509	1.718046
H	-0.964820	0.168689	4.393933
H	-1.702842	1.777163	4.141462
H	0.031447	1.543579	3.825527
H	-3.526538	0.301944	4.171737
H	-5.671293	-0.969935	3.157791
H	-5.148241	-1.588282	0.584261
H	-3.202089	2.779731	-0.305977
H	-3.343474	3.498122	2.300030
H	-5.762235	2.723730	3.253148
H	-7.115080	1.529815	1.229487
H	-5.529151	1.561867	-0.971227

H	-2.213221	1.473758	-2.482173
H	-3.827157	2.358415	-4.126718
H	-5.986333	1.167480	-4.537189
H	-6.487303	-0.944715	-3.295329
H	-4.864378	-1.842278	-1.663577
H	-2.076968	-2.471130	-3.039270
H	-1.947235	-4.911083	-3.449063
H	-2.114103	-6.516235	-1.539102
H	-2.431571	-5.647410	0.781605
H	-2.597365	-3.211867	1.191377
H	4.921184	1.812665	-0.836152
H	6.765629	1.043005	-2.288387
H	6.487003	-0.992489	-3.717898
H	4.320251	-2.247146	-3.653903
H	2.477019	-1.464652	-2.211213
H	2.613234	5.353662	1.707707
H	2.267586	5.216094	-2.568832
H	3.522066	2.167613	2.313837
H	2.592728	3.520225	3.009548
H	1.755306	2.065262	2.437188
H	3.064996	2.157783	-3.252673
H	1.396395	1.761837	-2.838649
H	1.757513	3.293520	-3.682347

H	1.436765	7.384732	-0.463737
H	3.008901	7.408327	0.369629
H	2.937497	7.346889	-1.417505
H	0.692021	0.418295	1.820868
H	3.006891	0.108713	2.575158
H	4.266130	-2.557710	0.241108
H	5.428779	0.831333	2.656714
H	6.606680	-2.787153	-0.565407
H	7.770281	0.602764	1.851701
H	8.368192	-1.211675	0.241927
H	2.561253	-1.360079	1.517377

V

C	3.657555	-3.966303	-0.496183
C	3.770338	-2.632588	-0.925137
C	5.042013	-2.144297	-1.271778
C	6.173378	-2.960273	-1.175777
C	6.052282	-4.282324	-0.731013
C	4.788657	-4.783175	-0.396526
C	2.528910	-1.779352	-0.990375
P	2.328561	-0.366747	0.245846
C	3.517460	1.022469	-0.148941

C	3.990931	1.838004	0.919531
C	4.882413	2.886887	0.648134
C	5.300714	3.204934	-0.643623
C	4.749800	2.460718	-1.690129
C	3.861130	1.396879	-1.480828
C	3.573296	1.707241	2.371898
C	6.284377	4.317460	-0.908170
C	3.306998	0.762251	-2.740694
C	3.090165	-1.183726	1.724501
C	2.293470	-1.740943	2.735258
C	2.874428	-2.429146	3.807256
C	4.262789	-2.567169	3.886097
C	5.068826	-2.004230	2.889041
C	4.487778	-1.319072	1.820648
Mn	-0.013812	0.261252	0.404876
N	0.543693	2.137977	-0.480591
C	0.397939	2.260993	-1.816073
C	0.879019	3.375377	-2.507784
C	1.507717	4.400903	-1.801500
C	1.628401	4.282691	-0.415726
C	1.136171	3.135824	0.198475
C	-0.368416	1.175453	-2.526353
N	-0.310942	-0.110639	-1.804640

C	-1.372021	-1.090467	-2.208509
C	-1.145675	-1.611872	-3.629573
C	-0.407806	-1.410945	0.896130
O	-0.640795	-2.481731	1.271565
C	0.140805	0.769143	2.097949
O	0.183858	1.117507	3.206291
P	-2.243537	0.933046	0.457693
C	-3.252881	0.257736	-0.892745
C	-4.652026	0.515393	-1.122518
C	-5.019493	-0.088166	-2.356321
C	-3.864156	-0.722827	-2.900623
C	-2.763197	-0.530058	-2.007376
Fe	-4.293902	-1.508834	-1.027455
C	-5.883250	-2.711270	-0.481480
C	-5.162152	-2.290271	0.679916
C	-3.818921	-2.764448	0.553873
C	-3.701551	-3.469709	-0.682497
C	-4.980636	-3.436590	-1.323495
C	-2.397982	2.761465	0.269352
C	-1.948989	3.566388	1.332685
C	-1.939361	4.957128	1.214561
C	-2.368658	5.564460	0.026979
C	-2.815767	4.771488	-1.033884

C	-2.833804	3.375928	-0.913516
C	-3.260769	0.630263	1.970249
C	-2.903339	-0.365333	2.892081
C	-3.721248	-0.646156	3.991880
C	-4.905273	0.071014	4.188716
C	-5.259169	1.083603	3.288509
C	-4.439962	1.365491	2.192176
H	1.221743	3.003056	1.277229
H	2.109087	5.055260	0.187459
H	1.897520	5.277815	-2.325399
H	0.760499	3.427973	-3.592515
H	-1.425465	1.485786	-2.572200
H	-0.026032	1.083578	-3.570726
H	-1.217321	-1.951454	-1.543243
H	-1.294279	-0.832956	-4.397680
H	-1.838545	-2.443771	-3.832621
H	-0.130991	-2.034725	-3.706129
H	-3.838929	-1.296022	-3.824897
H	-6.017977	-0.095411	-2.793084
H	-5.315061	1.071546	-0.462898
H	-5.551155	-1.689972	1.502771
H	-2.778957	-3.909875	-1.070578
H	-5.218923	-3.858710	-2.300334

H	-6.926718	-2.487446	-0.705561
H	-1.983851	-0.935040	2.761945
H	-3.427286	-1.429684	4.696486
H	-5.546496	-0.150171	5.047298
H	-6.175898	1.660916	3.441985
H	-4.720383	2.173437	1.511522
H	-1.599950	3.102562	2.260588
H	-1.588091	5.569945	2.050168
H	-2.355035	6.654317	-0.068782
H	-3.156894	5.237403	-1.963289
H	-3.200802	2.772200	-1.747347
H	5.004441	2.730153	-2.720320
H	5.239636	3.493952	1.486715
H	6.157809	-2.097105	2.941886
H	2.232468	-2.854700	4.584412
H	5.133714	-0.872280	1.063643
H	1.210177	-1.640035	2.708345
H	4.717026	-3.105052	4.723871
H	3.831711	-0.167678	-3.019375
H	2.238526	0.529454	-2.658929
H	3.410212	1.458133	-3.587796
H	4.287443	1.093677	2.947702
H	3.542505	2.706995	2.837158

H	2.585870	1.252339	2.507614
H	7.309294	3.915191	-1.022154
H	6.041955	4.856821	-1.840531
H	6.306257	5.045700	-0.079548
H	-3.012368	-2.594220	1.260969
H	0.564961	-0.573349	-2.046333
H	1.642037	-2.411073	-0.802578
H	2.663862	-4.365106	-0.270853
H	5.160674	-1.104488	-1.586715
H	4.681535	-5.818744	-0.057762
H	7.155732	-2.557898	-1.442592
H	6.938288	-4.919868	-0.650090
H	2.391304	-1.386472	-2.004361
Cl	0.016542	-4.239400	-1.622376

VI

C	-3.756949	2.935056	-0.314801
C	-3.895065	3.546272	0.969479
C	-5.242455	3.351694	1.409051
C	-5.935909	2.621285	0.393595
C	-5.018536	2.361328	-0.670561
Fe	-4.321027	1.524662	1.092965

C	-3.825645	0.699590	2.930862
C	-2.748997	0.548237	1.998878
C	-3.260528	-0.222053	0.883407
C	-4.645001	-0.512019	1.149562
C	-4.985089	0.056518	2.407163
C	-1.354433	1.121628	2.171999
C	-1.162935	1.726852	3.566433
P	-2.279209	-0.826170	-0.518669
Mn	-0.018235	-0.301860	-0.379527
C	0.145151	-0.801698	-2.079360
O	0.216129	-1.141906	-3.187221
C	-3.243079	-0.322196	-2.013095
C	-4.486782	-0.920736	-2.289177
C	-5.257294	-0.495734	-3.373874
C	-4.791879	0.528195	-4.208726
C	-3.548021	1.113743	-3.956021
C	-2.778036	0.688443	-2.867323
C	-2.554013	-2.648704	-0.500415
C	-2.325219	-3.377229	-1.681659
C	-2.385232	-4.773058	-1.676959
C	-2.660339	-5.460256	-0.488635
C	-2.882862	-4.742534	0.691037
C	-2.833727	-3.344158	0.685634

N	0.281643	-2.230988	0.520065
C	0.579991	-3.350503	-0.165664
C	0.864170	-4.567269	0.443199
C	0.840436	-4.634546	1.839137
C	0.482327	-3.492948	2.552505
C	0.178818	-2.312816	1.863435
C	-0.383574	-1.133413	2.603558
N	-0.288231	0.118590	1.825438
P	2.354188	0.199701	-0.286147
C	2.714581	1.998907	-0.628692
C	2.851108	2.971692	0.397439
C	3.224879	4.280990	0.058341
C	3.419359	4.688501	-1.264400
C	3.131505	3.757383	-2.266384
C	2.747590	2.438293	-1.983837
C	2.508866	2.712016	1.844951
C	3.890573	6.082534	-1.597562
C	2.247999	1.626623	-3.162934
C	-0.281859	1.405458	-0.809074
O	-0.432723	2.531760	-1.046985
C	3.525942	-0.659768	-1.445696
C	3.244717	-1.960576	-1.880237
C	4.167465	-2.680524	-2.648266

C	5.391642	-2.102321	-2.993767
C	5.685762	-0.802539	-2.562334
C	4.765137	-0.089353	-1.792089
C	3.164537	-0.401398	1.299749
C	4.669819	-0.475267	1.334670
C	5.479174	0.672343	1.403222
C	6.873796	0.569587	1.411216
C	7.491051	-0.685145	1.350406
C	6.698171	-1.836545	1.284391
C	5.304153	-1.728454	1.275656
Cl	2.013199	0.456483	4.431561
H	0.564786	-3.268468	-1.251906
H	1.098303	-5.438578	-0.171781
H	1.076868	-5.566124	2.360622
H	0.416570	-3.504950	3.643009
H	-1.447281	-1.349199	2.803972
H	0.131960	-1.007649	3.571343
H	-1.221378	1.930577	1.440693
H	-1.357409	0.986852	4.360057
H	-1.853750	2.576510	3.699206
H	-0.128764	2.076916	3.701756
H	-3.785468	1.251798	3.866985
H	-5.969533	0.035789	2.874177

H	-5.317317	-1.067739	0.498891
H	-2.842078	2.886613	-0.902381
H	-3.102544	4.042506	1.530781
H	-5.657396	3.673018	2.364684
H	-6.972615	2.287020	0.441286
H	-5.228477	1.796579	-1.578051
H	-1.811660	1.159694	-2.692145
H	-3.169339	1.906855	-4.607352
H	-5.395462	0.862038	-5.058045
H	-6.224484	-0.968448	-3.569132
H	-4.857806	-1.733653	-1.660397
H	-2.093696	-2.852026	-2.613061
H	-2.208497	-5.326051	-2.604402
H	-2.699432	-6.553627	-0.482334
H	-3.096355	-5.271398	1.624692
H	-3.015095	-2.797963	1.614758
H	5.018342	0.918381	-1.458622
H	6.643867	-0.341145	-2.820587
H	6.115450	-2.660198	-3.595754
H	3.921742	-3.695055	-2.976379
H	2.297870	-2.425594	-1.620179
H	3.347047	5.012400	0.864144
H	3.179617	4.072575	-3.314231

H	3.254356	2.133236	2.409204
H	2.371102	3.664671	2.381551
H	1.564370	2.157772	1.937637
H	2.416464	0.546733	-3.087026
H	1.156608	1.775648	-3.262752
H	2.713605	1.971417	-4.101095
H	3.587051	6.809206	-0.824180
H	4.995306	6.116319	-1.662836
H	3.495989	6.422980	-2.570633
H	0.589893	0.543143	2.145973
H	2.760735	0.111104	2.191398
H	4.692440	-2.634115	1.209856
H	5.021110	1.662446	1.424886
H	7.166207	-2.824832	1.234107
H	7.481628	1.478624	1.460208
H	8.582603	-0.764827	1.353469
H	2.754762	-1.424168	1.353266
